# Nuclear proteome response to cell wall removal in rice (*Oryza sativa*)

**DOI:** 10.1186/1477-5956-11-26

**Published:** 2013-06-19

**Authors:** Hana Mujahid, Feng Tan, Jian Zhang, Babi Ramesh Reddy Nallamilli, Ken Pendarvis, Zhaohua Peng

**Affiliations:** 1 Department of Biochemistry, Molecular Biology, Entomology and Plant Pathology, Mississippi State University, Starkville, MS 39762, USA; 2 Institute for Genomics, Biocomputing and Biotechnology, Mississippi State University, Mississippi State, MS 39762, USA; 3 Present Address: College of Agriculture and Life Sciences, University of Arizona, P.O. Box 210036, Tucson, AZ 85721, USA

**Keywords:** Protoplast, Rice, Nuclear proteins, Cell wall, Comparative proteomics

## Abstract

Plant cells are routinely exposed to various pathogens and environmental stresses that cause cell wall perturbations. Little is known of the mechanisms that plant cells use to sense these disturbances and transduce corresponding signals to regulate cellular responses to maintain cell wall integrity. Previous studies in rice have shown that removal of the cell wall leads to substantial chromatin reorganization and histone modification changes concomitant with cell wall re-synthesis. But the genes and proteins that regulate these cellular responses are still largely unknown. Here we present an examination of the nuclear proteome differential expression in response to removal of the cell wall in rice suspension cells using multiple nuclear proteome extraction methods. A total of 382 nuclear proteins were identified with two or more peptides, including 26 transcription factors. Upon removal of the cell wall, 142 nuclear proteins were up regulated and 112 were down regulated. The differentially expressed proteins included transcription factors, histones, histone domain containing proteins, and histone modification enzymes. Gene ontology analysis of the differentially expressed proteins indicates that chromatin & nucleosome assembly, protein-DNA complex assembly, and DNA packaging are tightly associated with cell wall removal. Our results indicate that removal of the cell wall imposes a tremendous challenge to the cells. Consequently, plant cells respond to the removal of the cell wall in the nucleus at every level of the regulatory hierarchy.

## Background

The cell wall is a critical extracellular structure that provides protection and structural support in plant cells. It controls the cell shape and allows the turgor pressure to build up and maintain an upright position for plants. In addition, it glues the cell together and serves as a barrier for pathogen infection and insect and animal damage. Plant cells are routinely exposed to various pathogens and environmental stresses that cause cell wall perturbations. Insect and herbivore bites and wind are common factors contributing to cell wall damage. Little is known about the mechanisms that plants use to sense these disturbances and transduce the signals to stimulate responses to maintain cell wall integrity. It has been demonstrated in yeast cells that transient damage to cell wall leads to induction of cell wall-related genes as a compensatory response to maintain cell integrity [1]. However, in spite of clues from many stress-related studies, it is unknown if such a mechanism exists in plant cells.

Plant cells can rapidly re-synthesize the cell wall after the cell wall is removed [2]. The plant protoplast culture is an excellent experiment displaying the astonishing cell wall re-synthesis capability. Interestingly, the cell wall re-synthesis mechanism in protoplasts is probably different from the one used for new cell wall synthesis during cell division [3]. Tan et al. (2011) found that removal of cell wall leads to cell wall synthesis at multiple sites in protoplasts [3]. In contrast, new cell wall synthesis during cell division is limited to only one site- the cell plate derived from the phragmoplast. In addition, substantial chromatin reorganization was observed in protoplasts. The chromatin reorganization was associated with histone modification changes at multiple modification sites of histones as shown in Western blot studies with multiple histone modification specific antibodies. The histone acetylation changes at H3K18 and H3K23 following cell wall removal and regeneration were further verified and quantified using isotope labeling assisted mass spectrometry analysis. In addition, 136 up regulated and 94 down regulated proteins were identified using shot gun proteomics and label-free quantification analysis [3]. Sharma et al. (2011) examined the transcriptome response to enzymatic removal of cell wall [4]. They found that kinases, transcription factors and genes predicted to be involved in cell wall-related functions were enriched in the differentially regulated gene category. In addition, rice lines carrying Tos17 mutations in genes up-regulated during cell wall removal exhibited dwarf phenotypes. Many of the genes up-regulated during cell wall re-synthesis following cell wall removal are also up-regulated in response to infection and environmental perturbations, indicating that there is a coordinated response to diverse types of stress.

The nucleus is the most prominent organelle that contains majority of the genetic materials in eukaryotes. It is the site of DNA replication, RNA transcription, and ribosome preassembling. The nucleus is surrounded by a double membrane called the nuclear envelope. The nucleus contains several subcompartments [5], including nucleolus, euchromatin domains, heterochromatin domains, cajal bodies, speckles, and other domains. The nuclear matrix is a karyoskeletal, non-histone structure that serves as a support for the genome and nuclear activities [6]. The essential roles of the nuclear activities to the cell suggest that the nucleus is the most important control center of the cell.

The nuclear proteome is highly complicated, with proteins ranging from very low copy transcription factors to highly abundant core histone proteins and ribosomal proteins. In plants, the nuclear proteome has been examined by several laboratories in different organisms. The nuclear proteins were extracted using different methods for proteomics studies, including Trizol extraction [7], fractionation with differential ionic strength [8], high NaCl concentration [9], HEPES buffer [10], lysis buffer [11,12], and phenol extraction [13]. In rice, glucose-responsive nuclear proteins were extensively examined [9]. Nuclear enriched proteomes were also studied in different tissues in rice [9,12,13]. The nuclear proteome response to cold stress has been examined in *Arabidopsis* with several transcription factors shown to be differentially regulated under stress. Nucleolar, nuclear matrix, and nuclear pore complex proteomes were also examined in *Arabidopsis*[14-17]. Although many nuclear proteome studies have been reported, the number of low abundance transcription factors identified in each study was usually less than ten. When nuclei-enrichment was combined with a DNA binding affinity column, about a dozen transcription factors were identified [9], suggesting that improving the nuclear protein purification and extraction methods may lead to a better coverage of the nuclear proteome, particularly the low abundance proteins.

Although differential histone modifications and chromatin reorganization in response to cell wall removal and regeneration have been observed in rice, the regulatory network controlling the process is still largely unknown. No regulatory genes specifically involved in this process have been identified at the protein level. In this report, we used multiple nuclear proteome extraction methods to examine the nuclear proteome response to the removal of the cell wall. A large number of nuclear proteins including histone modification proteins, chromatin structure regulatory proteins, and transcription factor proteins were identified. Our studies substantially advanced our understanding of the plant nuclear proteome and cellular responses to cell wall removal.

## Results

### Cell wall removal stimulates active cell wall synthesis

**Figure 1:**
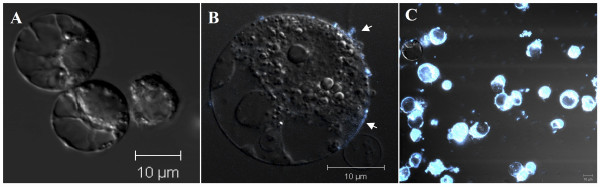
**Microscopy images of cultured rice protoplasts (from suspension cells) following the cell wall regeneration time course.** CLSM was used to observe the protoplasts stained by a fluorescent dye, Fluorescent Brightener 28, with polysaccharide specific binding activities. The excitation wavelength at 492 nm and emission at 520 nm were used. The protoplast culture times are 0hrs (**A**), 4 hrs (**B**), and 48 hrs (**C**). The arrows point at the positions of cell wall syntheses. The magnification is revealed by the scale bar.

To study how plant cells respond to the disturbance of cell wall, we examined cellular responses to the enzymatic removal of cell wall using rice suspension culture cells, the OC cell line [18,19]. Because of the unique cell wall structure of plants in the grass family, multiple hours of enzyme digestion are required to completely remove the rice cell wall [20-22]. After 9 hours of enzyme digestion, the cell wall was completely removed as revealed by the stain with Fluorescent Brightener 28, a fluorescent dye with specific polysaccharide binding activities (Figure 1A). Followed by 2 to 4 hours of culture of the protoplasts, new cell wall started to emerge (Figure 1B). After 48 hours of culture, the relatively spherical and smooth surface of protoplasts changed, suggesting the recovery of cell wall, which is verified by Fluorescent Brightener 28 stain (Figure 1C). We found that over 90% protoplasts could regrow their cell wall, suggesting that our protoplast isolation and culture is an excellent system to examine cellular response to the removal of cell wall.

### Nuclei enrichment and assessment

**Figure 2:**
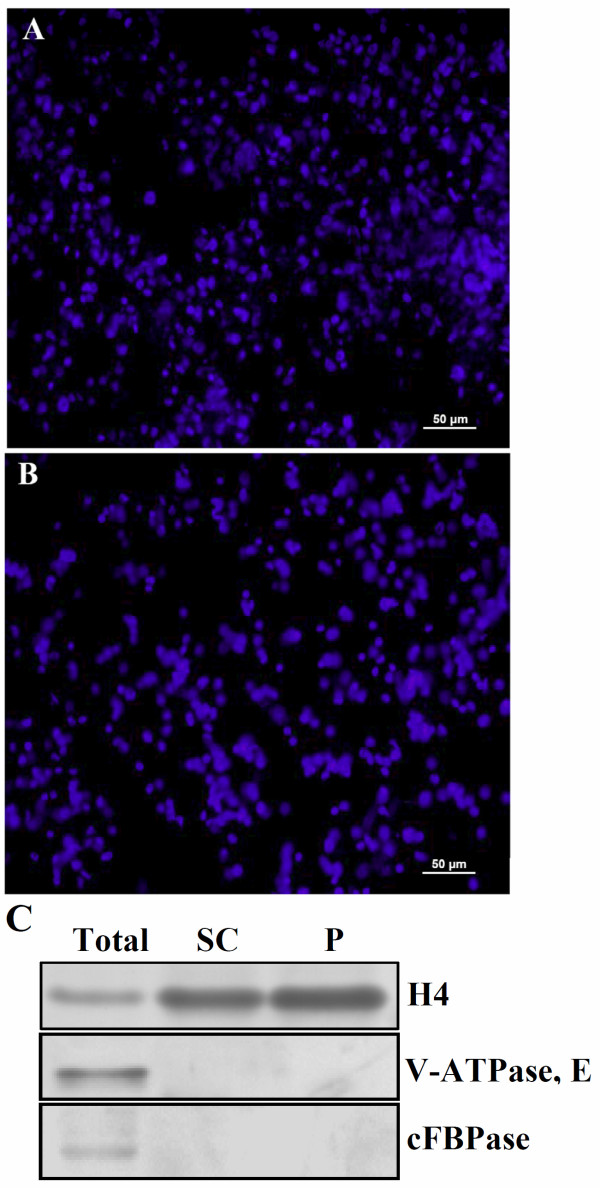
**Microscopy images of isolated rice (*O. sativa) *suspension cell and protoplast nuclei and Western blot analysis of purified nuclear proteins.** (**A**) Image of purified suspension cell nuclei after DAPI staining. (**B**) Image of protoplast with cell wall regeneration (4 hrs) nuclei after DAPI staining. A small volume of the purified nuclei was stained with DAPI (0.5 μg/ml) for 5 minutes and images were taken under a DAPI-filter. The magnification is revealed by the scale bar. (**C**) Nuclei enrichment revealed by Western blots. Antibodies against H4, V-ATPase, E, and cFBPase were used to assess the protein quantity in the total protein fraction and suspension cell and protoplast nuclei, respectively. 20 μg of proteins were loaded in each lane.

A high quality and large scale purification of nuclei is vital to nuclear subproteome analysis. We obtained nuclei from protoplasts and suspension cells, respectively. DAPI-staining of purified nuclei fractions from both suspension cells and protoplasts revealed that we obtained nuclei in a large scale from both suspension cells and protoplasts without any clear contamination from organelles such as chloroplasts and mitochondria as observed under the microscope (Figure 2A & B and data not shown). We validated the nuclear enrichment by Western blots with antibodies specific for known nuclear and cytosolic proteins. The Western blot results showed that histone H4 was highly enriched in the nuclear fraction compared to total protein extraction when equal amount of proteins were loaded (Figure 2C). In contrast, cytosolic fructose-1, 6-bisphosphatase (cFBPase) and vacuolar protein VHA-E were only detected in the total protein fraction (Figure 2C), indicating that the nuclear proteins were successfully enriched.

### Comparison of nuclear protein extraction methods

**Figure 3:**
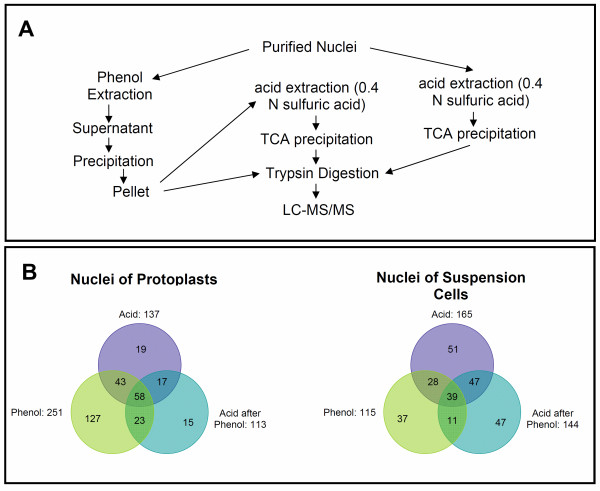
**Protein extraction methods utilized in this study and nuclear proteins identified in the various fractions.** (**A**) Suspension cell nuclei and protoplast nuclei were extracted with phenol alone, phenol and 0.4 N sulfuric acid (A & P), and 0.4 N sulfuric acid alone, respectively, followed by subsequent mass analysis. (**B**) Color-scheme Venn diagrams revealing identified nuclear proteins in each extraction procedure and the overlap among extraction procedures in suspension cell and protoplast nuclear samples. The numbers in circle areas equal the protein number identified. Purple: Acid extraction; Green: Phenol Extraction; Blue: Phenol-Acid double extraction.

Nuclear subproteomes have been studied with different protein extraction methods, including Trizol extraction [7], fractionation with differential ionic strength [8], high NaCl concentration [9], HEPES buffer [10], lysis buffer [11,12], and phenol extraction [13]. However, the low abundant nuclear proteins identified by mass spectrometry are still limited in plants. To optimize the method for nuclear protein identification, we tested different nuclear proteome extraction and fractionation methods as revealed in Figure 3A. To determine if a protein was localized in the nucleus, GO annotations were obtained from GORetriever, a tool available at AgBase [23,24]. We found that a combination of the phenol extraction with acid re-extraction could improve the nuclear subproteome coverage (Figure 3B). Phenol extraction of the nuclei derived from protoplasts and suspension cells followed by LC-MS/MS identified 251 and 115 nuclear proteins, respectively. Acid extraction followed by LC-MS/MS identified 137 and 165 nuclear proteins, respectively. When the phenol extracted samples were re-extracted by sulfuric acid and examined with LC-MS/MS, 113 and 144 nuclear proteins were identified in the nuclear samples of protoplasts and suspension cells, respectively. Among them, 15 and 47 proteins, respectively, were new proteins that were not identified by either the phenol or acid extraction method. The total nuclear proteins identified by each of the extraction methods are listed in Additional file 1: Table S1. Due to some overlap, overall we identified 382 nuclear proteins with two or more peptides. Among them, 26 were transcription factors. All proteins discussed and presented in this study met the criterion of two or more matched peptides. To verify our protein identification results, a reverse database of *O. sativa* was searched using the reverse database functionality in Bioworks 3.2 as previously reported (Additional file 1: Table S2) [3]. The peptide false discovery rate (FDR) for the entire dataset was 0.58%, while the protein FDR was 1.51%.

Analysis of the total identified peptides showed that about 31% of the peptides identified using phenol extraction were nuclear protein peptides. When the sample was re-extracted by acid, 67% of the identified peptides were nuclear protein peptides. Nine of the top 10 most abundant proteins (based on peptide counts) identified in the acid re-extraction samples were histones (Table 1). In contrast, none of the 10 most abundant proteins extracted by phenol alone were histones although the majority was nuclear proteins (Table 1), suggesting that acid re-extraction enriched nucleic acid associated proteins. Meanwhile, 47% of the peptides identified in samples directly extracted by acid were nuclear protein peptides. Of the 10 most abundant proteins identified by acid extraction, three were histones and three were nucleolar proteins.

**Table 1:** The most abundant proteins identified in phenol, acid, and phenol-acid extracted suspension cell and protoplast nuclear samples

**TIGR ID**	**Annotation**	**Peptide (Hits)**
Phenol-Extracted		
LOC_Os03g22740	Nucleolar protein NOP5-1, putative, expressed	108
LOC_Os08g04240	Cysteine-rich repeat secretory protein 55 precursor, putative, expressed	75
LOC_Os11g10480	Dehydrogenase, putative, expressed	73
LOC_Os03g22730	Nucleolar protein NOP5-1, putative, expressed	67
LOC_Os05g08360	rRNA 2-O-methyltransferase fibrillarin 2, putative, expressed	51
LOC_Os04g40950	Glyceraldehyde-3-phosphate dehydrogenase, putative, expressed	48
LOC_Os08g04250	Cysteine-rich repeat secretory protein 55 precursor, putative, expressed	47
LOC_Os03g22880	Nucleolar protein 5A, putative, expressed	46
LOC_Os02g57590	rRNA 2-O-methyltransferase fibrillarin 2, putative, expressed	44
LOC_Os02g38920	Glyceraldehyde-3-phosphate dehydrogenase, putative, expressed	44
Acid-Extracted		
LOC_Os08g04240	Cysteine-rich repeat secretory protein 55 precursor, putative, expressed	125
LOC_Os08g04250	Cysteine-rich repeat secretory protein 55 precursor, putative, expressed	107
LOC_Os08g04210	Cysteine-rich repeat secretory protein 55 precursor, putative, expressed	81
LOC_Os04g52960	Nucleolin, putative, expressed	70
LOC_Os03g22730	Nucleolar protein NOP5-1, putative, expressed	65
LOC_Os03g22740	Nucleolar protein NOP5-1, putative, expressed	63
LOC_Os07g44190	h/ACA ribonucleoprotein complex subunit 4, putative, expressed	51
LOC_Os01g61920	Histone H4	42
LOC_Os05g38640	Probable histone H2A.4	41
LOC_Os05g02300	Probable histone H2A.6	41
Phenol-Acid-Double Extracted		
LOC_Os02g56960	Ribosomal protein, putative, expressed	146
LOC_Os05g38640	Probable histone H2A.4	99
LOC_Os05g02300	Probable histone H2A.6	99
LOC_Os03g17100	Probable histone H2A.5	99
LOC_Os07g36500	Histone H4	70
LOC_Os01g61920	Histone H4	70
LOC_Os01g05900	Histone H2B.10	43
LOC_Os01g05630	Histone H2B.4	43
LOC_Os01g05610	Histone H2B.3	43
LOC_Os07g36140	Probable histone H2A.2	39

### Differentially expressed proteins in response to cell wall removal

Upon removal of cell wall, rice cells display substantial chromatin decondensation and reorganization [3]. To identify nuclear proteins that may be involved in chromatin decondensation and reorganization; we examined differentially expressed nuclear proteins upon the removal of cell wall. To reveal the differentially expressed proteins, we compared the suspension cell nuclear proteome with the protoplast nuclear proteome extracted by phenol extraction, acid re-extraction, and acid extraction, respectively. A non-labeling quantification method was used for differential regulation analysis. Previous reports and our studies have shown that the spectral count and ΣX_corr_ score methods generated identical results in all studies [3,25]. But the ΣX_corr_ score method provided values for direct comparison of protein fold-change. Therefore we used the ΣX_corr_ score method. Also, the sum of SEQUEST ΣX_corr_ has been shown to compare suitably with the concentrations of a known protein mixture in serial dilutions [26]. In the ΣX_corr_ method a preliminary list is built using all scans for peptides with an X_corr_ (generated by TurboSEQUEST^TM^ (Bioworks Browser 3.2, Thermo Electron)) above the threshold used for protein identification. Finally, the values of suspension cell control ΣX_corr_ versus protoplast treatment ΣX_corr_ for individual proteins identified are compared and statistically significant changes are used to assign regulation and fold-change [25,26]. The X_corr_ values generated from TurboSEQUEST were used for ΣX_corr_ quantification as reported by Nanduri and Bridges [26,27], in which three biological replicas of each sample treatment is required. The quantitative analysis criteria and procedure were identical to previously reported [3,25,27]. Differential expression was only considered for proteins with a p-value < 0.05.

**Figure 4:**
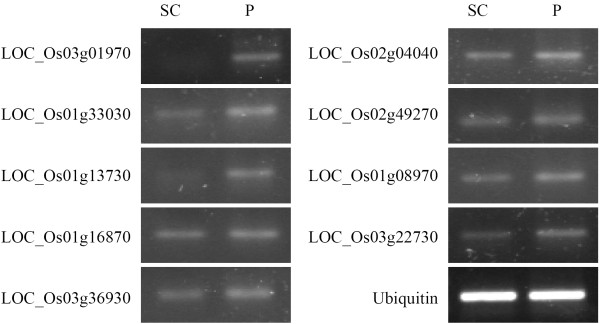
**RT-PCR analysis of differentially expressed genes in suspension cell and protoplast nuclei.** Rice ubiquitin gene was used as an internal control. Equal amount of cDNA template was used for suspension cell and protoplast nuclei cDNA samples. 35 cycles were used. Primers utilized in this study are provided in Additional file 4.

**Figure 5:**
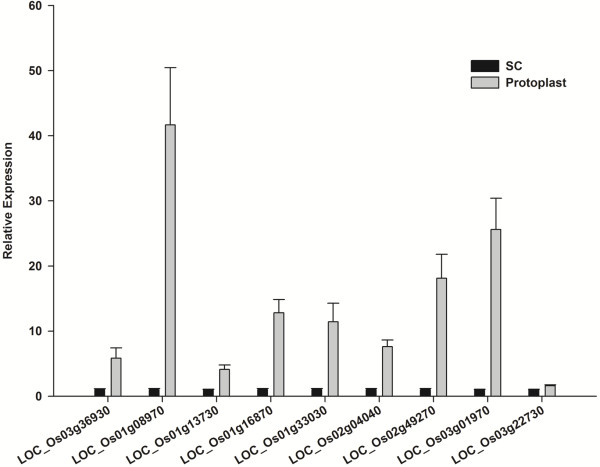
**Quantitative real-time PCR analyses of differentially expressed genes in suspension cell and protoplast nuclei.** Equivalent amount of cDNA template was used for each sample and rice ubiquitin gene was used as an internal control.

**Figure 6:**
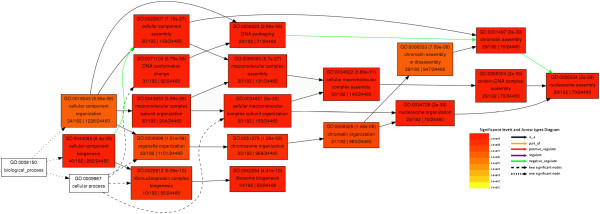
**Enriched GO biological processes of differentially expressed nuclear proteins.** Figure displays the significantly enriched biological processes revealed by GO annotation analysis for differentially expressed proteins. The top line in each box is the GO identifier of the term and statistical significance (multiple hypothesis corrected p-value, lower is more significant) of that annotation. The middle line in each box is a description of the GO term. The four numbers on the bottom line are the number of nuclear proteins that had this annotation, the number of nuclear proteins that had any annotation (192), the total number of proteins that had the annotation, and the total number of proteins that had any annotation (24460). The color of the box indicates the significance of the term as indicated by the legend on the bottom left corner. White boxes are not significant.

Following removal of cell wall, 142 nuclear proteins with a p-value < 0.05 displayed differential up regulation and 112 nuclear proteins with a p-value < 0.05 displayed differential down regulation (Additional file 1: Table S3). To validate the protein differential expression results generated by the ΣX_corr_ method between the suspension cells and protoplast at the transcriptional level, we randomly selected nine differentially expressed proteins for RT-PCR and real-time PCR analysis (Figures 4 and 5). The expression levels of these genes correlated with the non-labeled protein quantification results, providing further support for our protein quantification results. To further analyze the differentially regulated proteins, functional classification of the differentially expressed nuclear proteins was carried out according to the gene ontology (GO) rules using AgBase at http://www.agbase.msstate.edu/[24] and ortholog and Pfam domain information available for all proteins identified with two or more peptides was collected using the tools provided by the TIGR Rice Genome Annotation Project (http://rice.plantbiology.msu.edu/). Ortholog and Pfam domain information available for the identified proteins is presented in Additional files 2 and 3, respectively. Three independent gene ontologies were used to describe the function of gene products such as cellular component (CC), molecular function (MF) and biological process (BP) [28]. GO annotations were obtained from GORetriever, a tool available at AgBase [23,24]. GO classification was carried out using tools available at AgBase [23,24] and AgriGO [29,30]. Functional classification for differentially regulated proteins in categories as cellular component, molecular function, and biological process were found. The results are presented in Figure 6 and Additional file 1: Figure S1A and S1B, respectively.

Figure 6 shows the significantly enriched GO biological processes of differentially expressed nuclear proteins. The biological processes tightly associated with cell wall regeneration included chromatin assembly, nucleosome assembly, macromolecular complex subunit organization, protein-DNA complex assembly, and DNA packaging (Figure 6).

### Differential expression of transcriptional regulation proteins

Identifying regulatory proteins such as transcription factors controlling cellular response to cell wall removal is essential for revealing the cellular regulatory network. However, transcription factors are difficult to detect by mass spectrometry due to low copy numbers. We successfully identified 26 transcription factors and found that several of them were differentially regulated, including multiple zinc finger proteins. While zinc finger C-x8-C-x5-C-x3-H type family proteins LOC_Os02g06584 and LOC_Os06g46890 proteins were up regulated in response to cell wall removal, zinc finger family protein (LOC_Os04g57010) and ZOS3-23-C2H2 zinc finger protein (LOC_Os03g61640) were down regulated. Other differentially regulated transcription factors included Whirly transcription factor domain containing protein (LOC_Os06g05350), Helix-loop-helix DNA binding domain containing protein (LOC_Os02g39140), transcription factor TF2 (LOC_Os05g03740), and putative transcription factor (LOC_Os09g27850). Other proteins that might be involved in transcriptional regulation were also differentially regulated. The SKIP (SKI-interacting protein) is an essential spliceosomal component and transcriptional co-regulator, which may provide regulation by coupling transcription initiation with splicing [31]. A SKIP/SNW domain containing protein (LOC_Os02g52250) was down regulated. The BRCA1 C terminus domain containing protein (LOC_Os03g49210) was also up regulated. SSRP1-like FACT complex subunit (LOC_Os01g08970) was found to be up regulated. The FACT complex contains proteins such as SSRP1 and Spt16, which are connected with transcriptional elongation [32,33]. Finally, a putative DNA-directed RNA polymerase subunit (LOC_Os09g02284) was also up regulated. RNA polymerase II is a multi-subunit holoenzyme composed of ten to twelve protein subunits (RPB1-RPB12) [34-36]. LOC_Os09g02284 is an ortholog of the Arabidopsis protein DNA-directed RNA polymerase II subunit RPB3-B (At2g15400), which composes the core element of the RNA polymerase II protein.

### Differential expression of chromatin structure and modification proteins

A large number of genes regulating chromatin structure and function were differentially regulated, including core histone proteins, core histone domain containing proteins, HMG proteins, histone modification proteins, and nucleosome remodeling proteins. Interestingly, several core histone domain containing proteins were up regulated. The function of these genes remains to be further explored. Meanwhile, the H3 proteins were also up regulated. The HMG proteins are present in all tissues of eukaryotes, leading many to believe that HMG proteins are central for proper cellular function [37]. Johns et al. (1982) [38] estimated that HMG proteins bind to ≤10% of the nucleosomes, making them the second most abundant family of chromosomal proteins with probable structural function in the nucleus [37]. While HMG-Y-related protein (LOC_Os09g23730) was up regulated, the putative HMG1/2 (LOC_Os06g51220) was down regulated.

The histone modification proteins are believed to regulate the access of transcription factors, chromatin modifying enzymes, and chromatin remodeling factors to nucleosomal DNA by chemical modifications to histones. We found that while the putative histone deacetylase (LOC_Os07g06980) was up regulated, the histone methyltransferase (LOC_Os05g41172) was down regulated. A histone lysine N-methyltransferase H3 lysine-9 specific SUVHI (LOC_Os05g41172), which belongs to the SET family and contains an YDG_SRA domain, was found to be down regulated. The SRA (SET and RING finger associated) domain is believed to play a part in directing SUVH proteins to specific chromatin subdomains [39-42]. The YDG/SRA domain of KYP/SUVH4 has the ability to bind directly to methylated DNA, indicating that DNA methylation is necessary for SUVH targeting [43,44]. In Arabidopsis, loss of SUVH1 and SUVH4 causes weak reduction of heterochromatic histone H3K9 dimethylation [45].

In addition, a putative PHD finger protein (LOC_Os07g41740) and two RecF/RecN/SMC N terminal domain containing proteins (LOC_Os02g04040 (SMC3) and LOC_Os12g44390 (SMC1)) were up regulated.

### Differential expression of other important proteins

Many proteins with highly important biological roles were also shown to be differentially regulated. The differentially expressed proteins included: cleavage and polyadenylation specificity factor, CCAAT/enhancer-binding protein, RNA recognition motif containing proteins, OsTOP6B-Topoisomerase 6 subunit B protein, DEAD-box ATP-dependent RNA helicase, Nucleolar protein NOP5-1, 26S proteasome proteins, protease homologue, 14-3-3 proteins, importin subunit alpha, DNA topoisomerase 1, cell division control protein 48 homolog E, putative Argonaute protein.

## Discussion

### Nuclear proteome and comparison of nuclear protein extraction methods

Proteomic studies on biochemically isolated organelles require stringent protein categorization parameters that allow for distinction between valid and contaminating co-purifying components. In addition, many proteins shuttle between the nucleus and cytoplasm and are annotated in multiple cellular compartments. There are a variety of effective bioinformatic tools for predicting nuclear localization based on signal peptides and nuclear localization signals, however using these tools for sub nuclear domain categorization is not possible. Also, many of the entries in the datasets available through these tools rely heavily on Uniprot “subcellular localization” field keywords. In these circumstances, data available from the gene ontology project [28] can be utilized in conjunction allowing identified proteins to be classified on their cellular localization, biological process, and molecular function. Gene ontology is mainly based on available publications, which provides relevant evidence of cellular localization.

Recently, Aki and Yanagisawa (2009) [9] using nanoLC/ESI/MS/MS did extensive studies on the rice nuclear proteome. Aki and Yanagisawa (2009) [9] possibly identified the largest number of nuclear proteins in Rice thus far, using co-enrichment with nuclear purification as criteria for nuclear localization. In this study, cellular localization was classified based on prior publications and GO annotations. In recent years, electronic annotation has significantly improved in terms of specificity, reliability, and coverage [46]. Using this cellular localization criterion and two or more peptide match for protein identification, we successfully identified 382 nuclear proteins. Many of the proteins have not been reported in prior nuclear proteome studies.

We compared the nuclear proteomes extracted by phenol and sulfuric acid. The phenol extraction method identified 251 nuclear proteins in the nuclei derived from protoplasts and 115 proteins in the nuclei derived from suspension cells. In contrast, the acid extraction identified 137 nuclear proteins in protoplast nuclear sample and 165 nuclear proteins in suspension cell nuclear sample. The acid extracted proteins were mainly histones, nucleolar proteins, and ribosomal proteins. On the other hand, the proteins identified by phenol extraction were more diversified. Interestingly, we found that further fractionating the phenol extracted proteins by sulfuric acid uncovered nuclear proteins that were not identified by either method. Sulfuric acid re-extraction identified 113 nuclear proteins in protoplast nuclei and 144 proteins in suspension cell nuclei. Among them, 32 and 94 proteins were not identified by phenol extraction alone of the protoplast and suspension cell nuclei, respectively. Similarly, 38 and 58 of the proteins were not identified in acid extracted protoplast and suspension cell samples, respectively. The results suggested that the nuclear proteome is highly complex, further fractionation of the subproteome by acid can lead to a better coverage of the nuclear subproteome. Combining phenol, acid, and their double extraction, we identified 382 nuclear proteins with two or more peptides, including 26 transcription factors. The plant (rice) nuclear proteome has been studied extensively by many authors in tissues including rice seedlings, rice suspension cells, and rice seed endosperm and evolutionarily conserved and glucose responsive nuclear proteins have been identified among many other nuclear proteins [9,12,13]. Although the nuclear purification steps presented all appeared to be convincing, the coverage of nuclear proteins, particularly the low abundant nuclear proteins such as transcription factors, remains to be improved. Our results suggested that due to the complexity of the nuclear subproteome and the presence of high abundant proteins such as ribosomal proteins, further fractionation of the nuclear proteome is necessary to achieve a deeper coverage of the nuclear subproteome.

### Regulation of chromatin structure and histone modification change in response to cell wall removal

Previous studies find that removal of the cell wall is concomitant with substantial chromatin reorganization. Western blots and isotope-labeling assisted quantitative mass spectrometry analyses reveal that the chromatin reorganization is associated with substantial histone modification changes. Particularly, the H3K18 and H3K23 acetylation are substantially induced upon removal of the cell wall [3]. We are interested in identifying proteins involved in chromatin reorganization and histone modifications. In this study, we found that a histone deacetylase (LOC_Os07g06980) was up regulated and a histone-lysine N-methyltransferase (LOC_Os11g38900) was down regulated. Examining the relationship between the regulation of these two proteins in response to cell wall removal and the histone modification changes caused by cell wall removal is of interest [3]. To investigate whether there is any causal relationship between the two observed effects, we can use the mutants of these differentially regulated genes to examine their cellular response to cell wall removal and test acetyltransferase activity in response to cell wall removal. The structural maintenance of chromosomal (SMC) proteins function together with other proteins in a range of chromosomal transactions, including chromosome condensation, sister-chromatid cohesion, recombination, DNA repair and epigenetic silencing of gene expression [47]. The RecF/RecN proteins are required for DNA repair and homologous recombination. We found that two RecF/RecN/SMC N terminal domain containing proteins structural maintenance of chromosomes (SMC) SMC3 and SMC1, respectively (LOC_Os02g04040 and LOC_Os12g44390) were up regulated upon removal of cell wall. Their potential role in chromatin reorganization upon removal of cell wall is worthy of further examination. We found that several core histone domain containing proteins were up regulated in response to cell wall removal. Although the function of this group of genes is still unknown, it is a group of very interesting genes which should be further explored. A remarkable question is whether these proteins are directly involved in the chromatin re-organization induced by cell wall removal.

### Differentially expressed regulatory proteins and cellular process

To understand the cellular response to cell wall removal and the underlying regulatory mechanism, it is essential to elucidate the gene regulatory network. Transcription factors are the key regulators in gene expression control. We found that several transcription factors and transcriptional regulatory genes are differentially expressed in response to cell wall removal. These include two up regulated zinc finger proteins and two down regulated zinc finger proteins. Other differentially expressed transcription factors include Helix-loop-helix DNA binding protein, factor TF2 (LOC_Os05g03740) containing a myb-like family domain, and putative transcription factor (LOC_Os09g27850). Our study clearly demonstrated differential expression of transcription factors at the protein level in response to cell wall removal. In addition, we also observed protein level changes in putative DNA-directed RNA polymerase and other transcriptional regulators or co-regulators. Our results are consistent with the dramatic transcriptome change observed in response to cell wall removal revealed by oligo microarray studies in rice [4].

In addition to differential expression of proteins involved in the transcription process, we also observed protein differential expression in RNA binding proteins, RNA splicing proteins, ribosomal proteins, translational elongation factors, molecular chaperones, protein modification proteins, protein degradation proteins. The results suggested that the cells responded to cell wall at all levels. To further define the regulatory network, we carried out gene ontology analysis. GO analysis indicates that the biological processes tightly associated with cell wall removal includes chromatin assembly, nucleosome assembly, macromolecular complex subunit organization, protein-DNA complex assembly, and DNA packaging. Our results clearly indicate that removal of cell wall imposes a tremendous challenge to the cells. Consequently, plant cells respond to removal of cell wall in all major cellular components and biological processes.

## Materials

### Cell culture

The rice (*Oryza sativa*) suspension culture line OC was used for all experiments in this study [18,19]. Line OC was grown in the dark at 24°C in a gyratory shaker under a constant speed of 150 rpm in liquid B5 organic medium (pH 5.7) supplemented with 20 g/L sucrose, 0.5 g/L MES, 2.0 mg/L 2-4-dichlorophenoxyacetic acid (2,4-D) as previously reported [3,20,48]. Weekly subculture was performed at a dilution of 1:5 (cells:fresh medium).

## Methods

### Protoplast isolation and cell wall regeneration

OC cells were harvested five days after subculture for protoplast isolation. Protoplast isolation was performed as previously described [3,20]. Briefly, suspension cells were suspended in filter-sterilized enzyme solution containing 2.5% Cellulase RS (Onozuka RS), 1% Macroenzyme R10 (Research Products International), 0.4 M mannitol, 80 mM CaCl_2_, 0.125 mM MgCl_2_, 0.5 mM MES, and B5 organic medium with 2.0 mg/L 2,4-D (pH 5.6). After an incubation period in the dark for nine hours at 25°C, the protoplasts were collected by first filtering the enzyme solution through a 25 μm stainless steel sieve and then centrifuging the filtered solution at 120 × g for 5 min. The suspension cells were washed several times with protoplast suspension medium (0.4 M mannitol, 80 mM CaCl_2_, 0.125 mM MgCl_2_, 0.5 mM MES, and 2 g/L N-Z-Amine A in B5 organic medium plus 2.0 mg/L 2,4-D at pH 5.6). After protoplasts were washed, they were cultured in sealed petri dishes using protoplast suspension medium at a density of 5 × 10^5^ cells/ml in complete darkness at 25°C without agitation before being harvested for further study.

### Analysis of new cell wall formation

New cell wall formation was evaluated by monitoring the fluorescence of Fluorescent Brightener 28 (Calcofluor White M2R, Fluostain I, Sigma Aldrich, St. Louis, MO) using a confocal laser scanning microscope (CLSM) Zeiss Axiovert 200 M (Zeiss, Germany) as previously described [3].

### Nuclei isolation and purification

Rice suspension cells were suspended in nuclear isolation buffer (NIB: 10 mM Tris pH 8.0, 2 mM MgCl_2_, 1 mM CaCl_2_, 1 mM EDTA, 0.25 M sucrose, 0.1 mM spermidine, 0.5% Ficoll, 0.5% Triton-X 100 [added freshly], and 1 mM PMSF [added freshly], 1 mM DTT [added freshly]). The suspended cells were added to a pre-chilled blender and blended on high for 30 seconds. The homogenized slurry was first filtered through two layers of cheesecloth, and then filtered through a 25 μm stainless steel sieve to remove any unbroken cells. The filtered solution was centrifuged at 500 × g for 10 min at 4°C. The resulting pellet was re-suspended in NIB, under constant shaking at 4°C for 15 min, followed by centrifugation. Wash steps with NIB were repeated three times, followed by layering solution on a 2 M sucrose gradient, and centrifugation at 6000 × g for 30 min at 4°C to pellet purified nuclei. The resulting pellet was washed with NIB and used for further study. Protoplast nuclei were isolated the same way as previously described [3,20].

### Microscopic observation of purified nuclei

After purification, the integrity of isolated nuclei was assessed by staining with 4′, 6′ – diamidino-2-phenylindole hydrochloride (DAPI). A small volume of the purified nuclei was stained with DAPI (0.5 μg/ml) for 5 minutes and images were taken under a DAPI-filter.

### Nuclear protein extraction

The protein extraction method is a modification of our previous nuclear protein extraction procedure. The proteins for suspension cell nuclei and protoplast nuclei were extracted using phenol extraction as previously described [3,49,50]. Three biological replicates were extracted for both suspension cell nuclei and protoplast nuclei samples. The resulting pellets were further extracted using the acid extraction method or directly re-suspended in 8 M urea lysis buffer for trypsin digestion. Acid extraction for designated nuclear pellets was carried out as previously described [3,51]. To further fractionate the phenol extracted proteins, the phenol extracted pellet was suspended in 0.4 N sulfuric acid and incubated for 2 hours at 4°C with constant rotation. After incubation, the solution was centrifuged at 16,000 × g for 15 min at 4°C; the resulting supernatant was collected and precipitated with a final concentration of 33% trichloroacetic acid (TCA) for 30 min. The TCA precipitated pellet was washed with acetone and vacuum dried, followed by suspension in 8 M urea lysis buffer. Protein quantification was carried out for all samples using the RC DC™ Protein Assay Kit. Three replicates were performed for each nuclear protein extraction procedure (2 treatments × 3 methods × 3 replicates), resulting in a total of 18 mass spectrometric runs.

### Western blot analysis of purified nuclear proteins

Proteins were separated on a 12% SDS-PAGE gel and electrotransfer of gel proteins onto a PVDF membrane (Millipore) was carried out at 0.8 mA/cm^2^ gel area for 1.5 hours. Nonspecific binding sites on the membrane were blocked overnight with block solution (5% m/V non-fat milk, 0.05% v/v tween-20, and 1 X TBS). After blocking, the membrane was incubated with respective primary antibody for 2 hours at room temperature, followed by incubation with respective alkaline phosphatase conjugated secondary antibody for 90 minutes. Signal detection was carried out using NBT/BCIP detection system.

### RNA Isolation and RT-PCR analysis

Total RNA was extracted from suspension cell and protoplast nuclei using Trizol following manufacturer’s instructions provided by Invitrogen (Invitrogen, http://www.invitrogen.com). Reverse transcription of RNA was performed as previously described with minor modification [52,53]. Rice ubiquitin gene was used as an internal control. Resulting PCR products were examined using 1% agarose gel electrophoresis. PCR primers used in the study are supplied in Additional file 4.

### Quantitative real-time PCR Analysis

Real-time quantitative PCR analysis was performed as previously described [53]. The rice ubiquitin gene was used as an internal control. The 2^-ΔΔCT^ method was used to calculate relative transcript levels [54]. Primers used in the study are provided in Additional file 4.

### Protein digestion and shotgun proteomic analysis

Protein digestion was carried out as previously described [3,20,25]. Briefly, after dissolving proteins in 8 M Urea lysis buffer (pH 7.8), proteins were reduced with 10 mM DTT for 1 hour and alkylated with 50 mM IAA for 1 hour. Subsequently, the urea concentration was reduced to less than 0.6 M for trypsin digestion. Trypsin (Promega) was added at a final ratio of 1:50 (protease:protein) and digestion was carried out at 37°C overnight. Trypsin was inactivated by decreasing the pH to less than 2 by adding 2 μl of formic acid. Peptide mixtures were desalted with a Michrom Bioresources peptide desalting macrotrap following manufacturer’s instructions. The eluted peptides were vacuum-dried and resuspended in 20 μl 5% Acetonitrile, 0.1% formic acid for 1D liquid chromatography-electrospray ionization tandem MS (1D LC ESI MS/MS) using a Surveyor HPLC (Thermo) in-line with an ESI ion trap mass spectrometer (LCQ Deca XP Plus, ThermoElectron). A reverse-phase column (BioBasic C18 column (Thermo 72105–100266)) was used for peptide separation at a flow rate of 500 nl min-1. Peptides were loaded with 5% ACN, 0.1% formic acid for 20 min. The elution gradient (all solvents containing 0.1% formic acid) was as follows: 5-25% ACN in 450 min, followed by 25-50% in 130 min, followed by a 20 min wash with 95% ACN and then equilibration with 5% ACN for 55 min. The extended gradient time was used to compensate for the slow scan rate of the instrument. Data was collected over a total duration of 655 min using repetitive MS scans directly followed by three tandem MS/MS scans on the three most intense precursor masses from the full scan. Dynamic mass exclusion windows were 2 minutes long. The mass spectra and tandem mass spectra were searched against the *Oryza sativa* non-redundant protein database (TIGR, V7.0) downloaded on 1/19/2012 from TIGR Rice Genome Annotation (http://rice.plantbiology.msu.edu) by using TurboSEQUEST, Bioworks Browser 3.2 (Thermo Electron Corp). The database contained 66 338 protein entries. Criteria, parameters, and procedure used for protein identification were identical to what was previously reported [3]. The allowance for missed cleavages was one. The peptide (precursor) ion mass tolerance was 1.0 Da, and the fragment ion (MS2) tolerance was 0.5 Da. The requirement for protein identification was two peptides from a protein to meet the following criteria: X-correlation >1.9 (+1 charge), >2.2 (+2 charge), >3.75 (+3 charge); delta correlation value ≥0.08; probability <0.01. Using the reverse database functionality in Bioworks 3.2, the peptide and protein false discovery rates were estimated using the same search criteria as described above against the reverse *O. sativa* database.

### Protein quantification

TurboSEQUEST (Bioworks Browser 3.2, Thermo Electron Corporation), commercial software commonly used in mass data analysis was used to generate X_corr_ values. The ΣX_corr_ quantification method used was as reported by Nanduri and Bridges [26,27]. The ProtQuant software [27] was downloaded from AgBase [23] database tool box (http://www.agbase.msstate.edu/). Quantitative analysis criteria and procedure were identical to previously reported [3,25]. A peptide X_corr_ value was only considered if it passed the following protein identification criteria: X-correlation >1.9 (+1 charge), >2.2 (+2 charge), >3.75 (+3 charge); delta correlation value ≥0.08; probability <0.01). Using the library R statistical package http://www.r-project.org/, ProtQuant performed one-way ANOVA analysis for proteins identified with three or more peptide scans in comparative treatments to determine the statistical significance of differential expression (p-value). Differential regulation was only considered for proteins with a p-value < 0.05.

### Gene ontology annotation

In order to carry out protein functional categorization, the gene ontology (GO) rules provided with the GO browser at http://www.geneontology.org/[28] were followed. Gene ontologies can be classified into three independent groups: biological process (BP), molecular function (MF), and cellular component (CC). Using the GORetriever tool available at AgBase [23] (http://www.agbase.msstate.edu/), GO annotations were assigned. If GO annotations could not be retrieved using this tool, other websites including Uniprot, TIGR, NCBI, and Gramene were used to retrieve annotations. GOSlimViewer (available at AgBase) tool was used to retrieve GoSlim ids. Functional categorization of genes was also carried out according to the GO rules [28] at agriGO [29,30].

## Competing interests

The authors declare that they have no competing interests.

## Authors’ contributions

Conceived, designed, and implemented the study: ZP; Protein extraction and purification: HM; Assistant with mass spectrometry analysis: KP; Western blot analysis: HM BN; Staining and CLSM imaging of protoplasts: FT; Data analysis: HM JZ ZP; Reagents/materials/analysis tools: ZP; Drafted the manuscript: ZP HM; All authors edited the manuscript and approved the final version.

## Supplementary Material

Additional file 1: Table S1 Nuclear Proteins Identified with Two or More Matched Peptides. Table S2. Peptides Identified in Reverse Database Searches. Table S3. Differentially Regulated Nuclear Proteins. Figure S1. Enriched cellular component and molecular function of differentially expressed nuclear proteins revealed by GO analysis. Click here for file

Additional file 2 As Orthologous Proteins from Different Plant Species. Click here for file

Additional file 3 As Pfam Domain Assignment for Proteins Identified with two or more peptides. Click here for file

Additional file 4 As List of Primers Used in the Study. Click here for file

## References

[B1] Garcia R Bermejo C Grau C Perez R Rodriguez-Pena JM Francois J Nombela C Arroyo J The global transcriptional response to transient cell wall damage in Saccharomyces cerevisiae and its regulation by the cell integrity signaling pathway J Biol Chem 2004 279 15183 15195 10.1074/jbc.M312954200 14739279

[B2] Mishra AK Colvin JR The formation of wall-like envelopes by isolated tomato-fruit protoplasts Protoplasma 1969 67 295 305 10.1007/BF01254895

[B3] Tan F Zhang K Mujahid H Verma DP Peng Z Differential histone modification and protein expression associated with cell wall removal and regeneration in rice (Oryza sativa) J Proteome Res 2011 10 551 563 10.1021/pr100748e 20958091

[B4] Sharma R Tan F Jung KH Sharma MK Peng Z Ronald PC Transcriptional dynamics during cell wall removal and regeneration reveals key genes involved in cell wall development in rice Plant Mol Biol 2011 77 391 406 10.1007/s11103-011-9819-4 21887580

[B5] Shaw PJ Brown JW Plant nuclear bodies Curr Opin Plant Biol 2004 7 614 620 10.1016/j.pbi.2004.09.011 15491908

[B6] Fawcett DW On the occurrence of a fibrous lamina on the inner aspect of the nuclear envelope in certain cells of vertebrates Am J Anat 1966 119 129 145 10.1002/aja.1001190108 6007824

[B7] Abdalla KO Thomson JA Rafudeen MS Protocols for nuclei isolation and nuclear protein extraction from the resurrection plant Xerophyta viscosa for proteomic studies Anal Biochem 2009 384 365 367 10.1016/j.ab.2008.09.049 18938124

[B8] Gonzalez-Camacho F Medina FJ Extraction of nuclear proteins from root meristematic cells Methods Mol Biol 2007 355 63 72 17093303 10.1385/1-59745-227-0:63

[B9] Aki T Yanagisawa S Application of rice nuclear proteome analysis to the identification of evolutionarily conserved and glucose-responsive nuclear proteins J Proteome Res 2009 8 3912 3924 10.1021/pr900187e 19621931

[B10] Jones AM MacLean D Studholme DJ Serna-Sanz A Andreasson E Rathjen JP Peck SC Phosphoproteomic analysis of nuclei-enriched fractions from Arabidopsis thaliana J Proteomics 2009 72 439 451 10.1016/j.jprot.2009.02.004 19245862

[B11] O‘Farrell PH High resolution two-dimensional electrophoresis of proteins J Biol Chem 1975 250 4007 4021 236308 PMC2874754

[B12] Khan MM Komatsu S Rice proteomics: recent developments and analysis of nuclear proteins Phytochemistry 2004 65 1671 1681 10.1016/j.phytochem.2004.04.012 15276429

[B13] Li G Nallamilli BR Tan F Peng Z Removal of high-abundance proteins for nuclear subproteome studies in rice (Oryza sativa) endosperm Electrophoresis 2008 29 604 617 10.1002/elps.200700412 18203134

[B14] Calikowski TT Meulia T Meier I A proteomic study of the arabidopsis nuclear matrix J Cell Biochem 2003 90 361 378 10.1002/jcb.10624 14505352

[B15] Gonzalez-Camacho F Medina FJ Identification of specific plant nucleolar phosphoproteins in a functional proteomic analysis Proteomics 2004 4 407 417 10.1002/pmic.200300645 14760710

[B16] Pendle AF Clark GP Boon R Lewandowska D Lam YW Andersen J Mann M Lamond AI Brown JW Shaw PJ Proteomic analysis of the Arabidopsis nucleolus suggests novel nucleolar functions Mol Biol Cell 2005 16 260 269 15496452 10.1091/mbc.E04-09-0791 PMC539170

[B17] Tamura K Fukao Y Iwamoto M Haraguchi T Hara-Nishimura I Identification and characterization of nuclear pore complex components in Arabidopsis thaliana Plant Cell 2010 22 4084 4097 10.1105/tpc.110.079947 21189294 PMC3027183

[B18] Baba A Hasezawa S Syono K Cultivation of rice protoplasts and their transformation mediated by Agrobacterium spheroplasts Plant Cell Physiol 1986 27 463 472

[B19] Kyozuka J Fujimoto H Izawa T Shimamoto K Anaerobic induction and tissue-specific expression of maize Adh1 promoter in transgenic rice plants and their progeny Mol Gen Genet 1991 228 40 48 1715976 10.1007/BF00282445

[B20] Tan F Li G Chitteti BR Peng Z Proteome and phosphoproteome analysis of chromatin associated proteins in rice (Oryza sativa) Proteomics 2007 7 4511 4527 10.1002/pmic.200700580 18022940

[B21] Yamada Y Yang ZQ Tang DT Plant regeneration from protoplast-derived callus of rice (*Oryza sativa* L.) Plant Cell Rep 1986 5 85 88 10.1007/BF00269240 24248040

[B22] Vogel J Unique aspects of the grass cell wall Curr Opin Plant Biol 2008 11 301 307 10.1016/j.pbi.2008.03.002 18434239

[B23] McCarthy FM Bridges SM Wang N Magee GB Williams WP Luthe DS Burgess SC AgBase: a unified resource for functional analysis in agriculture Nucleic Acids Res 2007 35 D599 D603 10.1093/nar/gkl936 17135208 PMC1751552

[B24] McCarthy FM Wang N Magee GB Nanduri B Lawrence ML Camon EB Barrell DG Hill DP Dolan ME Williams WP AgBase: a functional genomics resource for agriculture BMC Genomics 2006 7 229 10.1186/1471-2164-7-229 16961921 PMC1618847

[B25] Chitteti BR Tan F Mujahid H Magee BG Bridges SM Peng Z Comparative analysis of proteome differential regulation during cell dedifferentiation in Arabidopsis Proteomics 2008 8 4303 4316 10.1002/pmic.200701149 18814325

[B26] Nanduri B Lawrence ML Vanguri S Pechan T Burgess SC Proteomic analysis using an unfinished bacterial genome: the effects of subminimum inhibitory concentrations of antibiotics on Mannheimia haemolytica virulence factor expression Proteomics 2005 5 4852 4863 10.1002/pmic.200500112 16247735

[B27] Bridges SM Magee GB Wang N Williams WP Burgess SC Nanduri B ProtQuant: a tool for the label-free quantification of MudPIT proteomics data BMC Bioinformatics 2007 8 Suppl 7 S24 10.1186/1471-2105-8-S7-S24 18047724 PMC2099493

[B28] Ashburner M Ball CA Blake JA Botstein D Butler H Cherry JM Davis AP Dolinski K Dwight SS Eppig JT Gene ontology: tool for the unification of biology. The Gene Ontology Consortium Nat Genet 2000 25 25 29 10.1038/75556 10802651 PMC3037419

[B29] Du Z Zhou X Ling Y Zhang Z Su Z AgriGO: a GO analysis toolkit for the agricultural community Nucleic Acids Res 2010 38 W64 W70 10.1093/nar/gkq310 20435677 PMC2896167

[B30] Wee CW Dinneny JR Tools for high-spatial and temporal-resolution analysis of environmental responses in plants Biotechnol Lett 2010 32 1361 1371 10.1007/s10529-010-0307-8 20502944

[B31] Folk P Puta F Skruzny M Transcriptional coregulator SNW/SKIP: the concealed tie of dissimilar pathways Cell Mol Life Sci 2004 61 629 640 10.1007/s00018-003-3215-4 15052407 PMC11138892

[B32] Perales M Mas P A functional link between rhythmic changes in chromatin structure and the Arabidopsis biological clock Plant Cell 2007 19 2111 2123 10.1105/tpc.107.050807 17616736 PMC1955692

[B33] Duroux M Houben A Ruzicka K Friml J Grasser KD The chromatin remodelling complex FACT associates with actively transcribed regions of the Arabidopsis genome Plant J 2004 40 660 671 10.1111/j.1365-313X.2004.02242.x 15546350

[B34] Malkus A Chang PF Zuzga SM Chung KR Shao J Cunfer BM Arseniuk E Ueng PP RNA polymerase II gene (RPB2) encoding the second largest protein subunit in Phaeosphaeria nodorum and P. avenaria Mycol Res 2006 110 1152 1164 10.1016/j.mycres.2006.07.015 17020806

[B35] Archambault J Friesen JD Genetics of eukaryotic RNA polymerases I, II, and III Microbiol Rev 1993 57 703 724 8246845 10.1128/mr.57.3.703-724.1993 PMC372932

[B36] Ishihama A Kimura M Mitsuzawa H Subunits of yeast RNA polymerases: structure and function Curr Opin Microbiol 1998 1 190 196 10.1016/S1369-5274(98)80010-6 10066472

[B37] Launholt D Merkle T Houben A Schulz A Grasser KD Arabidopsis chromatin-associated HMGA and HMGB use different nuclear targeting signals and display highly dynamic localization within the nucleus Plant Cell 2006 18 2904 2918 10.1105/tpc.106.047274 17114349 PMC1693932

[B38] Johns EW The HMG Chromosomal Proteins 1982 London: Academic

[B39] Baumbusch LO Thorstensen T Krauss V Fischer A Naumann K Assalkhou R Schulz I Reuter G Aalen RB The Arabidopsis thaliana genome contains at least 29 active genes encoding SET domain proteins that can be assigned to four evolutionarily conserved classes Nucleic Acids Res 2001 29 4319 4333 10.1093/nar/29.21.4319 11691919 PMC60187

[B40] Casas-Mollano JA Lao NT Kavanagh TA Intron-regulated expression of SUVH3, an Arabidopsis Su(var)3-9 homologue J Exp Bot 2006 57 3301 3311 10.1093/jxb/erl093 16928780

[B41] Citterio E Papait R Nicassio F Vecchi M Gomiero P Mantovani R Di Fiore PP Bonapace IM Np95 is a histone-binding protein endowed with ubiquitin ligase activity Mol Cell Biol 2004 24 2526 2535 10.1128/MCB.24.6.2526-2535.2004 14993289 PMC355858

[B42] Yu Y Dong A Shen WH Molecular characterization of the tobacco SET domain protein NtSET1 unravels its role in histone methylation, chromatin binding, and segregation Plant J 2004 40 699 711 10.1111/j.1365-313X.2004.02240.x 15546353

[B43] Johnson LM Bostick M Zhang X Kraft E Henderson I Callis J Jacobsen SE The SRA methyl-cytosine-binding domain links DNA and histone methylation Curr Biol 2007 17 379 384 10.1016/j.cub.2007.01.009 17239600 PMC1850948

[B44] Qin FJ Sun QW Huang LM Chen XS Zhou DX Rice SUVH histone methyltransferase genes display specific functions in chromatin modification and retrotransposon repression Mol Plant 2010 3 773 782 10.1093/mp/ssq030 20566579

[B45] Naumann K Fischer A Hofmann I Krauss V Phalke S Irmler K Hause G Aurich AC Dorn R Jenuwein T Reuter G Pivotal role of AtSUVH2 in heterochromatic histone methylation and gene silencing in Arabidopsis EMBO J 2005 24 1418 1429 10.1038/sj.emboj.7600604 15775980 PMC1142535

[B46] Skunca N Altenhoff A Dessimoz C Quality of computationally inferred gene ontology annotations PLoS Comput Biol 2012 8 e1002533 10.1371/journal.pcbi.1002533 22693439 PMC3364937

[B47] Haering CH Lowe J Hochwagen A Nasmyth K Molecular architecture of SMC proteins and the yeast cohesin complex Mol Cell 2002 9 773 788 10.1016/S1097-2765(02)00515-4 11983169

[B48] Lee TJ Shultz RW Hanley-Bowdoin L Thompson WF Establishment of rapidly proliferating rice cell suspension culture and its characterization by fluorescence-activated cell sorting analysis Plant Mol Biol Report 2004 22 259 267 10.1007/BF02773136

[B49] Chitteti BR Peng Z Proteome and phosphoproteome differential expression under salinity stress in rice (Oryza sativa) roots J Proteome Res 2007 6 1718 1727 10.1021/pr060678z 17385905

[B50] Hurkman WJ Tanaka CK Solubilization of plant membrane proteins for analysis by two-dimensional gel electrophoresis Plant Physiol 1986 81 802 806 10.1104/pp.81.3.802 16664906 PMC1075430

[B51] Shechter D Dormann HL Allis CD Hake SB Extraction, purification and analysis of histones Nat Protoc 2007 2 1445 1457 10.1038/nprot.2007.202 17545981

[B52] Zhang J Nallamilli BR Mujahid H Peng Z OsMADS6 plays an essential role in endosperm nutrient accumulation and is subject to epigenetic regulation in rice (Oryza sativa) Plant J 2010 64 604 617 10.1111/j.1365-313X.2010.04354.x 20822505

[B53] Nallamilli BR Zhang J Mujahid H Malone BM Bridges SM Peng Z Polycomb group gene OsFIE2 regulates rice (Oryza sativa) seed development and grain filling via a mechanism distinct from Arabidopsis PLoS Genet 2013 9 e1003322 10.1371/journal.pgen.1003322 23505380 PMC3591265

[B54] Livak KJ Schmittgen TD Analysis of relative gene expression data using real-time quantitative PCR and the 2(-Delta Delta C(T)) Method Methods 2001 25 402 408 10.1006/meth.2001.1262 11846609

