# Supplementary material for: Nuclear proteome response to cell wall removal in rice (Oryza sativa)
Source: Proteome Sci. 2013 Jun 19;11:26. doi: 10.1186/1477-5956-11-26 (PMC3695858; doi:10.1186/1477-5956-11-26)
Supplement: Additional file 1: Table S1 — Nuclear Proteins Identified with Two or More Matched Peptides. Table S2. Peptides Identified in Reverse Database Searches. Table S3. Differentially Regulated Nuclear Proteins. Figure S1. Enriched cellular component and molecular function of differentially expressed nuclear proteins revealed by GO analysis. [file 1477-5956-11-26-S1.pdf]

Supplement Table 1. Nuclear Proteins Identified with Two or More Matched Peptides

Locus ID	Name	P (pro)	Coverage (%)	MW (Da)	Peptides	Method		
						A	B	C
LOC_Os12g44390	RecF/RecN/SMC N terminal domain containing protein, expressed	5.54E-08	13.26	65674.3	5	*		
LOC_Os12g42150	WD domain, G-beta repeat domain containing protein, expressed	3.66E-07	6.12	34210.7	2	*		
LOC_Os12g41930	SRP40, C-terminal domain containing protein, expressed	2.52E-04	9.60	48089.0	4		*	
LOC_Os12g41715	DEAD-box ATP-dependent RNA helicase, putative, expressed	7.39E-09	21.34	65594.1	9	*	*	*
LOC_Os12g41620	WD domain, G-beta repeat domain containing protein, expressed	1.18E-06	13.17	86906.5	8	*		*
LOC_Os12g38180	heat shock cognate 70 kDa protein 2, putative, expressed	3.13E-05	5.58	24333.3	4		*	
LOC_Os12g38000	60S ribosomal protein L8, putative, expressed	7.67E-11	18.77	28249.3	5	*	*	*
LOC_Os12g34510	Probable histone H2AXb	7.02E-09	8.70	14339.3	3	*	*	*
LOC_Os12g25690	UDP-glucose 6-dehydrogenase, putative, expressed	1.38E-04	8.54	52854.4	2	*		
LOC_Os12g25120	Probable histone H2A.7	3.42E-12	17.04	14052.0	2	*	*	
LOC_Os12g21798	40S ribosomal protein S3a, putative, expressed	3.64E-10	18.46	29710.3	5	*		*
LOC_Os12g14070	DnaK family protein, putative, expressed	6.40E-04	2.01	74086.4	2			*
LOC_Os12g07010	ribosomal protein L3, putative, expressed	5.88E-10	14.65	44463.7	4	*		*
LOC_Os12g06910	nucleolar protein family 6, putative, expressed	1.69E-06	4.15	117149.9	4	*		
LOC_Os12g03880	60S acidic ribosomal protein P0, putative, expressed	5.47E-05	18.44	34466.5	4	*		
LOC_Os12g01430	ribosomal protein L10, putative, expressed	4.29E-08	17.41	28096.1	3	*		*
LOC_Os11g43900	translationally-controlled tumor protein, putative, expressed	2.45E-05	16.15	14684.5	2	*		
LOC_Os11g43890	WD domain, G-beta repeat domain containing protein, expressed	1.01E-07	13.62	33930.1	2	*		
LOC_Os11g40090	A49-like RNA polymerase I associated factor family protein, expressed	1.51E-04	8.68	52756.6	2		*	
LOC_Os11g38959	40S ribosomal protein S9-2, putative, expressed	6.32E-08	13.33	22673.0	2	*		*
LOC_Os11g38900	histone-lysine N-methyltransferase, H3 lysine-9 specific SUVH1, putative,	7.40E-06	8.00	89214.4	4	*	*	
LOC_Os11g37080	h/ACA ribonucleoprotein complex subunit 1-like protein 1, putative, expres	1.50E-07	38.78	20005.3	4	*	*	*
LOC_Os11g36390	RFC1 - Putative clamp loader of PCNA, replication factor C subunit 1, expr	8.04E-06	3.04	110899.6	2		*	
LOC_Os11g34450	14-3-3-like protein GF14-D	3.55E-07	29.43	29261.5	5	*		
LOC_Os11g29190	40S ribosomal protein S5, putative, expressed	7.37E-08	14.07	22206.5	2	*		*
LOC_Os11g19864	Transposon protein, putative, ping/pong/SNOOPY sub-class	4.18E-06	15.90	48160.7	5	*	*	*
LOC_Os11g11390	ribosomal protein, putative, expressed	2.34E-08	17.41	25346.5	4	*	*	*
LOC_Os11g07470	expressed protein	8.83E-08	6.76	127054.6	5	*		
LOC_Os11g06750	ribosomal protein L3, putative, expressed	5.88E-10	14.65	44490.7	4	*		*
LOC_Os11g05562	40S ribosomal protein S25, putative, expressed	1.43E-07	12.04	11918.8	2		*	
LOC_Os11g04070	60S acidic ribosomal protein P0, putative, expressed	5.47E-05	18.44	34422.4	4	*		
LOC_Os11g01420	ribosomal protein L10, putative, expressed	2.36E-08	32.62	26552.3	4	*		*
LOC_Os10g41470	60S ribosomal protein L27-3, putative, expressed	1.92E-05	8.09	15471.4	2		*	*

Supplement Table 1 cont. Nuclear Proteins Identified with Two or More Matched Peptides								
**Locus ID**	**Name**	**P (pro)**	**Coverage (%)**	**MW (Da)**	**Peptides**	**Method**		
						**A**	**B**	**C**
LOC_Os10g35290	DNA-directed RNA polymerase I subunit RPA2, putative, expressed	1.55E-07	6.47	115827.6	5	*		
LOC_Os10g35280	nucleolar complex protein 2, putative, expressed	7.75E-07	4.23	86352.9	3	*		*
LOC_Os10g32880	U-box domain-containing protein 72	1.26E-06	15.29	54400.2	4	*		
LOC_Os10g31520	ribosomal RNA assembly protein mis3, putative, expressed	3.38E-05	2.78	45187.8	2	*		
LOC_Os10g30580	cell division control protein 48 homolog E, putative, expressed	9.51E-08	14.73	89810.6	9	*		*
LOC_Os10g28230	Probable histone H2A variant 2	5.26E-06	23.74	14611.9	3	*	*	*
LOC_Os10g27190	40S ribosomal protein S17, putative, expressed	4.28E-06	16.31	16429.0	3		*	*
LOC_Os09g39540	40S ribosomal protein S25, putative, expressed	7.29E-10	17.20	10700.5	2		*	*
LOC_Os09g37860	NOL1/NOP2/sun family protein, putative, expressed	9.03E-06	12.45	78272.4	6	*		*
LOC_Os09g36160	LRP1, putative, expressed	1.50E-07	36.83	32021.3	5	*	*	
LOC_Os09g32532	60S ribosomal protein L32, putative, expressed	2.44E-11	32.33	15674.4	5	*	*	*
LOC_Os09g32520	60S ribosomal protein L32, putative, expressed	2.44E-11	42.11	15704.5	5		*	*
LOC_Os09g32500	60S ribosomal protein L32, putative, expressed	2.44E-11	42.11	15730.5	5		*	*
LOC_Os09g31180	ribosomal protein L6, putative, expressed	4.04E-09	41.58	21348.6	5	*		*
LOC_Os09g30418	Heat shock protein 81-2	4.65E-08	11.57	94194.8	9	*	*	
LOC_Os09g30412	heat shock protein, putative, expressed	4.65E-08	13.73	80199.4	9	*	*	
LOC_Os09g27850	transcription regulator, putative, expressed	1.30E-08	7.73	46193.8	3	*		
LOC_Os09g24820	ZF-HD protein dimerisation region containing protein, expressed	2.24E-12	14.96	35323.4	2			*
LOC_Os09g24260	WD domain, G-beta repeat domain containing protein, expressed	1.45E-06	5.64	109677.6	4	*		
LOC_Os09g23730	HMG-Y-related protein A, putative, expressed	1.29E-10	23.00	21752.6	5	*	*	*
LOC_Os09g18440	transposon protein, putative, Pong sub-class, expressed	4.18E-06	15.90	48160.7	5	*	*	*
LOC_Os09g15775	expressed protein	2.42E-06	9.33	48924.1	3	*	*	*
LOC_Os09g15770	CPuORF13 - conserved peptide uORF-containing transcript, expressed	2.42E-06	9.33	48924.1	3	*	*	*
LOC_Os09g10770	OsTOP6B - Topoisomerase 6 subunit B, expressed	3.70E-09	14.22	78014.8	7	*		
LOC_Os09g08430	ribosomal protein L22, putative, expressed	1.29E-07	15.79	19495.3	3		*	
LOC_Os09g02810	CCAAT-box-binding transcription factor-like protein	1.77E-07	7.47	110342.6	6	*		
LOC_Os09g02284	DNA-directed RNA polymerase subunit, putative, expressed	6.16E-06	21.52	36460.0	4	*		
LOC_Os08g44480	40S ribosomal protein S25, putative, expressed	7.29E-10	26.85	12068.0	3		*	*
LOC_Os08g44450	L1P family of ribosomal proteins domain containing protein, expressed	1.79E-07	6.94	24482.9	2			*
LOC_Os08g44380	L1P family of ribosomal proteins domain containing protein, expressed	1.79E-07	6.94	24592.0	2			*
LOC_Os08g42920	60S ribosomal protein L7, putative, expressed	2.50E-06	10.89	28985.9	3	*	*	*
LOC_Os08g41810	ribosomal protein L22, putative, expressed	5.34E-07	10.38	20836.9	2		*	
LOC_Os08g41300	60S ribosomal protein L32, putative, expressed	2.44E-11	32.33	15716.5	5	*	*	*
LOC_Os08g39140	Heat shock protein 81-1	2.10E-06	12.40	74243.8	8	*	*	
LOC_Os08g38410	RNA recognition motif containing	8.94E-08	10.22	38125.5	2	*		

Supplement Table 1 cont. Nuclear Proteins Identified with Two or More Matched Peptides

Locus ID	Name	P (pro)	Coverage (%)	MW (Da)	Peptides	Method		
						A	B	C
LOC_Os08g38300	Histone H2B.2	6.10E-10	10.00	16334.0	2		*	*
LOC_Os08g37490	14-3-3-like protein GF14-A	7.31E-08	16.29	29000.5	4	*	*	
LOC_Os08g36450	transcription regulator, putative, expressed	4.27E-05	2.64	48578.1	3	*		
LOC_Os08g33920	60S ribosomal protein L34, putative, expressed	1.56E-08	13.56	13634.0	2		*	
LOC_Os08g33370	14-3-3-like protein GF14-C	4.92E-08	29.69	28826.3	7	*	*	
LOC_Os08g33120	RNA recognition motif containing protein, putative, expressed	1.56E-09	25.17	30490.9	4	*	*	*
LOC_Os08g33100	Probable histone H2A.3	1.87E-10	21.64	13917.0	2	*	*	
LOC_Os08g32090	DEAD-box ATP-dependent RNA helicase, putative, expressed	1.73E-06	9.99	95531.7	5	*	*	
LOC_Os08g31810	RNA recognition motif containing protein, putative, expressed	3.06E-11	27.57	25103.9	3	*	*	*
LOC_Os08g31240	FACT complex subunit SPT16, putative, expressed	9.70E-08	3.54	114700.2	3			*
LOC_Os08g29650	RNA recognition motif containing protein, expressed	1.05E-07	12.19	32947.2	4	*	*	
LOC_Os08g23710	ribosomal protein L7Ae, putative, expressed	2.34E-08	5.43	29303.7	3			*
LOC_Os08g21840	50S ribosomal protein L15, putative, expressed	1.93E-06	14.58	31768.8	3		*	
LOC_Os08g21660	WD domain, G-beta repeat domain containing protein, expressed	1.10E-09	10.79	35241.4	3	*		
LOC_Os08g13690	60S ribosomal protein L7, putative, expressed	8.13E-08	5.31	28453.2	3			*
LOC_Os08g09350	gar2, putative, expressed	5.40E-07	4.90	60393.3	4			*
LOC_Os08g06040	60S ribosomal protein L34, putative, expressed	1.56E-08	13.56	13620.0	2		*	
LOC_Os08g05880	Putative U3 snoRNP protein IMP4	1.45E-09	20.14	33916.6	5	*	*	*
LOC_Os08g05840	DNA topoisomerase 1, putative, expressed	1.13E-05	1.53	102666.1	2	*		
LOC_Os08g04280	actin, putative, expressed	3.22E-06	13.91	54125.6	6	*		
LOC_Os08g03640	60S acidic ribosomal protein P0, putative, expressed	6.77E-10	24.76	34377.4	5	*		*
LOC_Os08g03520	retrotransposon protein, putative, Ty1-copia subclass, expressed	5.63E-08	20.81	18693.7	2		*	
LOC_Os08g02410	40S ribosomal protein S13, putative, expressed	4.83E-07	34.44	17145.2	4	*	*	*
LOC_Os08g02400	40S ribosomal protein S13, putative, expressed	8.46E-05	16.56	17115.2	4	*	*	*
LOC_Os07g49150	26S protease regulatory subunit 4, putative, expressed	4.17E-07	11.61	49586.7	3	*		
LOC_Os07g47420	60S ribosome subunit biogenesis protein NIP7, putative, expressed	1.27E-08	10.64	20878.9	2		*	*
LOC_Os07g46720	nucleolar protein 5A, putative, expressed	2.33E-04	5.43	34880.8	2	*		
LOC_Os07g46370	WD domain, G-beta repeat domain containing protein, expressed	3.89E-06	2.93	121745.7	2	*		
LOC_Os07g44190	h/ACA ribonucleoprotein complex subunit 4, putative, expressed	1.06E-09	27.67	65866.2	16	*	*	*
LOC_Os07g43980	DEAD-box ATP-dependent RNA helicase, putative, expressed	8.91E-06	7.98	55502.0	4	*		
LOC_Os07g42950	40S ribosomal protein S6, putative, expressed	7.42E-11	10.80	28472.0	2	*	*	*
LOC_Os07g42450	ribosomal protein S2, putative, expressed	1.02E-04	6.56	32972.0	2	*		*
LOC_Os07g42170	60S ribosomal protein, putative, expressed	2.12E-07	21.23	16016.7	2		*	*
LOC_Os07g41750	40S ribosomal protein S3-1, putative, expressed	4.40E-07	18.75	23148.9	3	*		*
LOC_Os07g41740	PHD finger protein, putative, expressed	2.32E-05	10.43	30414.7	2	*		
LOC_Os07g41260	PPR repeat domain containing protein, putative, expressed	2.18E-06	10.08	53714.4	3			*

Supplement Table 1 cont. Nuclear Proteins Identified with Two or More Matched Peptides

Locus ID	Name	P (pro)	Coverage (%)	MW (Da)	Peptides	Method		
						A	B	C
LOC_Os07g40930	WD repeat-containing protein 12, putative, expressed	2.68E-11	13.30	47659.4	4	*		
LOC_Os07g36500	Histone H4	8.02E-09	23.30	11409.3	7	*	*	*
LOC_Os07g36140	Probable histone H2A.2	2.65E-09	17.04	13976.1	2	*	*	*
LOC_Os07g36130	Probable histone H2A.1	3.42E-12	17.04	14044.1	2	*	*	
LOC_Os07g33340	DEAD-box ATP-dependent RNA helicase, putative, expressed	4.49E-07	12.18	86669.9	6	*	*	*
LOC_Os07g32350	WD domain, G-beta repeat domain containing protein, expressed	3.17E-10	14.09	46838.8	3	*		*
LOC_Os07g25440	WD domain, G-beta repeat domain containing protein, expressed	1.36E-08	4.57	77221.1	2	*		
LOC_Os07g20580	DEAD-box ATP-dependent RNA helicase, putative, expressed	1.37E-07	8.01	55622.5	3	*		*
LOC_Os07g19190	ribosomal protein L24, putative, expressed	7.76E-07	7.36	19761.8	2	*	*	*
LOC_Os07g12910	PHD finger protein, putative, expressed	4.88E-05	13.11	27404.5	3	*		
LOC_Os07g12650	ribosomal protein L7Ae, putative, expressed	3.96E-08	11.52	18353.7	2	*	*	
LOC_Os07g12320	WD domain, G-beta repeat domain containing protein, expressed	1.51E-07	15.38	58102.4	5	*		*
LOC_Os07g12250	ribosomal protein L24, putative, expressed	1.72E-07	14.91	18234.4	2		*	*
LOC_Os07g10660	ribosomal protein, putative, expressed	5.64E-05	8.33	30108.8	3	*		*
LOC_Os07g10350	S1 RNA binding domain containing protein, expressed	1.39E-07	13.82	99679.0	7	*		
LOC_Os07g08960	RNA recognition motif containing protein, expressed	4.24E-10	18.83	33250.4	5	*		
LOC_Os07g08880	ES43 protein, putative, expressed	5.45E-07	11.06	24794.0	2	*		
LOC_Os07g08660	40S ribosomal protein S15, putative, expressed	4.01E-06	26.62	17415.3	2	*		
LOC_Os07g08330	ribosomal protein L4, putative, expressed	8.55E-12	20.74	44738.6	9	*	*	*
LOC_Os07g07580	ROOT HAIRLESS 1, putative, expressed	1.13E-05	7.08	39623.1	2	*		*
LOC_Os07g07220	LSM domain containing protein, expressed	1.69E-05	12.26	27348.9	2	*	*	*
LOC_Os07g06980	histone deacetylase, putative, expressed	1.69E-06	7.22	67066.0	6	*		*
LOC_Os07g05580	ribosomal protein L7Ae, putative, expressed	3.96E-08	26.09	14799.0	3	*	*	*
LOC_Os07g03240	RNA recognition motif containing protein, putative, expressed	3.29E-03	7.96	41006.0	2	*		
LOC_Os07g01920	nucleolar GTP-binding protein 1, putative, expressed	1.91E-08	5.77	77067.4	3	*		*
LOC_Os07g01490	kinesin motor domain containing protein, putative, expressed	1.17E-03	8.38	43932.6	3	*		
LOC_Os06g48750	DEAD-box ATP-dependent RNA helicase, putative, expressed	7.44E-08	23.58	41913.1	9	*	*	
LOC_Os06g48355	expressed protein	2.42E-06	9.33	48869.0	3	*	*	*
LOC_Os06g48350	CPuORF14 - conserved peptide uORF-containing transcript, expressed	2.42E-06	9.33	48869.0	3	*	*	*
LOC_Os06g48230	DNA-directed RNA polymerases I, II, and III subunit RPABC3, putative, expr	8.48E-09	20.42	21551.7	2	*		
LOC_Os06g46890	zinc finger C-x8-C-x5-C-x3-H type family protein, expressed	2.08E-08	11.29	42478.6	6	*	*	
LOC_Os06g45710	phosphoglycerate kinase protein, putative, expressed	1.17E-08	21.70	42278.6	7	*	*	
LOC_Os06g43690	WD repeat-containing protein, putative, expressed	5.38E-08	19.05	100826.6	11	*		
LOC_Os06g41384	zinc finger C-x8-C-x5-C-x3-H type family protein, expressed	3.70E-05	4.07	30451.4	2		*	
LOC_Os06g40600	elongation factor, putative,	6.13E-08	15.75	110497.2	11	*		*

Supplement Table 1 cont. Nuclear Proteins Identified with Two or More Matched Peptides

Locus ID	Name	P (pro)	Coverage (%)	MW (Da)	Peptides	Method		
						A	B	C
LOC_Os06g40560	26S protease regulatory subunit S10B, putative, expressed	9.20E-06	6.48	44581.1	3	*		
LOC_Os06g36870	transposon protein, putative, Pong sub-class, expressed	4.18E-06	15.90	48160.7	5	*	*	*
LOC_Os06g36160	40S ribosomal protein S24, putative, expressed	1.72E-04	17.39	15708.4	2		*	
LOC_Os06g30320	NOC3 - Putative nucleolar complex subunit 3, expressed	1.31E-05	6.74	95710.7	3	*		
LOC_Os06g21516	transposon protein, putative, Pong sub-class, expressed	4.18E-06	15.90	48160.7	5	*	*	*
LOC_Os06g17840	expressed protein	1.49E-12	5.45	174766.2	6	*	*	*
LOC_Os06g16290	ribosomal protein L7Ae, putative, expressed	7.96E-05	36.67	16354.2	3	*	*	
LOC_Os06g14470	RNA recognition motif containing protein, putative, expressed	4.76E-07	10.88	31817.5	2	*	*	
LOC_Os06g12780	OsDegp10 - Putative Deg protease homologue, expressed	4.47E-09	14.01	68028.0	4	*		
LOC_Os06g10710	expressed protein	7.10E-08	8.45	31671.5	2	*		*
LOC_Os06g10430	protein of unknown function DUF1296 domain containing protein, expressed	1.04E-06	4.33	92197.7	2		*	
LOC_Os06g09570	nucleolar GTP-binding protein 1, putative, expressed	1.42E-06	9.17	77205.6	4	*		*
LOC_Os06g08770	ruvB-like 2, putative, expressed	2.18E-08	13.03	51843.1	4	*		
LOC_Os06g07580	expressed protein	4.62E-06	17.74	6931.1	2		*	
LOC_Os06g06880	Ser/Thr protein phosphatase family protein, putative, expressed	2.17E-05	10.87	36184.3	2	*		
LOC_Os06g06480	Histone H3	1.12E-13	12.80	27976.0	2		*	*
LOC_Os06g06460	Histone H3.2	1.12E-13	23.53	15267.8	2		*	*
LOC_Os06g05660	NAP domain containing protein, putative, expressed	1.73E-04	3.37	40511.1	2			*
LOC_Os06g05350	whirly transcription factor domain containing protein, expressed	1.98E-04	7.72	30161.1	2	*		
LOC_Os06g03780	NUC153 domain containing protein, expressed	4.70E-10	14.94	80926.8	5	*		
LOC_Os06g02510	ribosomal protein L13, putative, expressed	3.87E-08	24.52	24053.9	4	*	*	*
LOC_Os06g01700	CWC15 homolog A, putative, expressed	7.91E-06	17.39	26942.4	2		*	
LOC_Os05g51850	AT hook-containing DNA-binding protein, putative, expressed	1.89E-06	17.76	53755.3	7	*	*	
LOC_Os05g51830	ZOS5-12 - C2H2 zinc finger protein, expressed	1.11E-09	24.58	32546.9	6	*		*
LOC_Os05g51180	hyaluronan/mRNA binding family domain containing protein, expressed	6.06E-09	18.02	41028.5	3		*	
LOC_Os05g49890	GTP-binding nuclear protein Ran-2	7.53E-07	29.65	22737.1	4	*		
LOC_Os05g49860	Histone H2B.9	4.21E-11	9.87	16255.9	2		*	*
LOC_Os05g49230	ribosomal RNA large subunit methyltransferase J, putative, expressed	2.16E-07	7.08	92341.6	3			*
LOC_Os05g49030	60S ribosomal protein L18a, putative, expressed	5.27E-09	10.11	21459.0	4	*	*	*
LOC_Os05g48820	DDT, putative, expressed	1.08E-06	3.13	206399.0	4	*		
LOC_Os05g44320	WD domain, G-beta repeat domain containing protein, expressed	4.31E-12	10.47	98037.5	5	*		
LOC_Os05g41172	histone-lysine N-methyltransferase, H3 lysine-9 specific SUVH1, putative,	3.64E-07	10.12	73563.9	4	*	*	
LOC_Os05g40820	ribosomal protein L24, putative, expressed	1.24E-07	14.91	18859.2	2		*	
LOC_Os05g38640	Probable histone H2A.4	9.03E-12	18.40	16970.8	2	*	*	*
LOC_Os05g30880	nucleolar matrix protein-related, putative, expressed	8.69E-06	3.62	43583.6	2			*
LOC_Os05g30530	40S ribosomal protein S4, putative,	2.22E-09	12.55	29662.5	2			*

Supplement Table 1 cont. Nuclear Proteins Identified with Two or More Matched Peptides								
Locus ID	Name	P (pro)	Coverage (%)	MW (Da)	Peptides	Method		
						A	B	C
LOC_Os05g28280	peptidase, M24 family protein, putative, expressed	1.70E-07	14.25	43197.2	4	*		*
LOC_Os05g27940	40S ribosomal protein S7, putative, expressed	2.59E-07	26.04	22306.8	3	*	*	*
LOC_Os05g22920	digestive organ expansion factor, putative, expressed	8.95E-07	5.72	76118.0	3	*		
LOC_Os05g16660	WD domain, G-beta repeat domain containing protein, expressed	1.10E-09	4.42	35013.3	2	*		
LOC_Os05g11710	ribosomal protein L5, putative, expressed	3.01E-10	17.58	20836.0	2	*		
LOC_Os05g10620	no apical meristem protein, putative, expressed	7.67E-09	28.81	39354.9	4		*	
LOC_Os05g09620	SCC3, putative, expressed	9.17E-05	4.39	126855.5	3	*		
LOC_Os05g08360	rRNA 2-O-methyltransferase fibrillarin 2, putative, expressed	1.81E-12	33.33	32572.9	9	*	*	*
LOC_Os05g07700	ribosomal protein, putative, expressed	6.03E-08	22.81	19509.8	3	*	*	*
LOC_Os05g06770	40S ribosomal protein S27a, putative, expressed	8.08E-09	18.71	17682.5	2		*	
LOC_Os05g06350	Importin subunit alpha-1b	1.00E-08	14.23	58566.1	7	*		
LOC_Os05g06310	60S ribosomal protein L18-3, putative, expressed	6.08E-07	17.55	21310.9	5	*	*	*
LOC_Os05g04850	RNA recognition motif containing protein, putative, expressed	6.53E-10	8.33	22104.2	2	*		
LOC_Os05g03740	transcription factor TF2, putative, expressed	4.18E-06	12.91	36946.7	2	*	*	
LOC_Os05g02300	Probable histone H2A.6	9.03E-12	19.23	16500.4	2		*	*
LOC_Os05g01450	eukaryotic translation initiation factor 3 subunit F, putative, expressed	3.38E-04	14.00	21863.7	2	*		
LOC_Os04g58830	ribosome biogenesis regulatory protein, putative, expressed	2.12E-07	26.36	36850.0	6	*	*	*
LOC_Os04g57010	zinc finger C-x8-C-x5-C-x3-H type family protein, expressed	3.26E-06	19.09	31787.6	4		*	
LOC_Os04g56720	RCC2, putative, expressed	2.64E-08	12.75	58407.7	4	*		*
LOC_Os04g56590	ATP/GTP binding protein, putative, expressed	9.55E-09	17.34	40256.8	4	*		*
LOC_Os04g56350	nucleolar protein,Nop52 containing protein, expressed	6.49E-09	9.32	60254.9	3	*		*
LOC_Os04g52960	nucleolin, putative, expressed	9.86E-12	12.73	75252.9	9	*	*	*
LOC_Os04g52200	RNA recognition motif containing protein, putative, expressed	2.32E-08	1.55	101159.6	2	*		*
LOC_Os04g51630	60S ribosomal protein L7, putative, expressed	8.13E-08	5.33	28361.1	3			*
LOC_Os04g50990	L11 domain containing ribosomal protein, putative, expressed	1.03E-07	35.54	17747.6	4	*	*	*
LOC_Os04g50660	WD domain, G-beta repeat domain containing protein, expressed	5.11E-10	16.84	97560.2	10	*		
LOC_Os04g49580	nucleolar complex protein, putative, expressed	9.10E-10	13.39	65224.5	6	*		*
LOC_Os04g48060	RFC2 - Putative clamp loader of PCNA, replication factor C subunit 2, expr	1.36E-06	21.19	36900.3	5	*		
LOC_Os04g46920	zinc knuckle domain containing protein, expressed	1.76E-07	12.64	30593.7	2	*	*	
LOC_Os04g45940	transcription factor like protein, putative, expressed	4.21E-10	19.51	22366.4	3	*		*
LOC_Os04g42270	60S ribosomal protein L23A, putative, expressed	3.84E-05	5.26	16910.0	2		*	*
LOC_Os04g42140	eukaryotic initiation factor iso-4F subunit p82-34, putative, expressed	1.58E-04	5.17	87021.0	2	*		
LOC_Os04g41040	DNA-directed RNA polymerases I, II, and III subunit RPABC1, putative, expr	1.08E-04	15.57	19535.3	2	*		
LOC_Os04g39444	LSM domain containing protein, expressed	4.23E-07	27.19	12625.8	2		*	*

Supplement Table 1 cont. Nuclear Proteins Identified with Two or More Matched Peptides								
Locus ID	Name	P (pro)	Coverage (%)	MW (Da)	Peptides	Method		
						A	B	C
LOC_Os04g38870	14-3-3-like protein GF14-B	4.23E-06	17.18	29863.4	4	*		
LOC_Os04g38310	expressed protein	2.70E-06	12.04	31898.6	2		*	
LOC_Os04g36890	peptidyl-prolyl cis-trans isomerase, FKBP-type, putative, expressed	4.37E-08	17.90	57177.7	6	*	*	*
LOC_Os04g34100	expressed protein	3.55E-05	13.85	24583.1	2			*
LOC_Os04g31320	SWIB/MDM2 domain containing protein, expressed	9.92E-08	24.41	51481.7	8	*		
LOC_Os04g28180	ribosomal protein, putative, expressed	4.26E-09	25.34	24928.4	5	*	*	*
LOC_Os04g28090	MYB family transcription factor, putative, expressed	6.22E-04	3.40	109683.3	3	*	*	
LOC_Os04g25550	FACT complex subunit SPT16, putative, expressed	9.70E-08	3.50	118587.7	3			*
LOC_Os04g02820	elongation factor, putative, expressed	5.50E-11	14.00	93973.0	8	*	*	*
LOC_Os04g01740	heat shock protein, putative, expressed	8.76E-05	3.41	80249.6	2	*		
LOC_Os03g63690	BCCIP, putative, expressed	2.22E-04	6.98	40136.7	3	*		*
LOC_Os03g63670	expressed protein	2.47E-08	4.82	105582.2	2		*	
LOC_Os03g61990	glycine-rich RNA-binding protein 7, putative, expressed	1.23E-03	13.17	21823.2	2		*	
LOC_Os03g61640	ZOS3-23 - C2H2 zinc finger protein, expressed	1.85E-05	15.84	23655.6	2		*	
LOC_Os03g61560	expressed protein	4.07E-06	11.99	73325.6	6	*		*
LOC_Os03g59310	ribosomal protein, putative, expressed	1.47E-07	18.25	29975.6	7	*		*
LOC_Os03g58810	DEAD-box ATP-dependent RNA helicase 27	3.61E-06	14.41	66650.6	6	*		*
LOC_Os03g58530	ES43 protein, putative, expressed	2.11E-10	25.23	25047.4	3	*		
LOC_Os03g58430	40S ribosomal protein S15, putative, expressed	4.01E-06	23.43	19657.8	2	*		
LOC_Os03g58204	ribosomal protein L4, putative, expressed	1.60E-08	15.59	44488.4	5	*		*
LOC_Os03g55150	eukaryotic translation initiation factor 5A, putative, expressed	4.39E-09	7.45	17544.7	2	*		
LOC_Os03g55070	UDP-glucose 6-dehydrogenase, putative, expressed	1.38E-04	8.54	52898.4	2	*		
LOC_Os03g54890	ribosomal protein L13, putative, expressed	1.69E-07	13.81	20733.6	3	*	*	*
LOC_Os03g53220	U5 small nuclear ribonucleoprotein 200 kDa helicase, putative, expressed	3.79E-07	2.10	242550.6	3	*		
LOC_Os03g53190	Probable histone H2A variant 3	1.43E-04	17.52	14458.7	2	*	*	*
LOC_Os03g52470	WD domain, G-beta repeat domain containing protein, expressed	3.83E-07	10.78	89000.5	6	*		
LOC_Os03g51200	Probable histone H2AXa	7.05E-06	24.09	14271.3	3	*	*	
LOC_Os03g50480	phosphoglucomutase, putative, expressed	4.80E-06	6.93	54533.1	2	*		
LOC_Os03g50290	14-3-3-like protein GF14-F	5.82E-10	35.38	29177.6	7	*	*	
LOC_Os03g50090	transposon protein, putative, CACTA, En/Spm sub-class, expressed	1.10E-05	6.93	84520.5	3	*		
LOC_Os03g49210	BRCA1 C Terminus domain containing protein, expressed	4.29E-11	16.06	69342.5	6	*		*
LOC_Os03g47800	RNA recognition motif containing protein, expressed	5.13E-06	3.25	34036.3	2	*	*	
LOC_Os03g46770	RNA recognition motif containing protein, expressed	1.08E-07	17.90	15901.8	2	*		
LOC_Os03g46490	40S ribosomal protein S21, putative, expressed	3.04E-08	39.02	9283.3	2		*	
LOC_Os03g42770	Putative U3 small nucleolar ribonucleoprotein complex-associated protein	1.23E-06	16.24	57027.6	7	*		*
LOC_Os03g42110	semialdehyde dehydrogenase, NAD binding domain containing protein,	9.52E-06	17.35	44786.2	5	*		

Supplement Table 1 cont. Nuclear Proteins Identified with Two or More Matched Peptides

Locus ID	Name	P (pro)	Coverage (%)	MW (Da)	Peptides	Method		
						A	B	C
LOC_Os03g41612	ribosomal protein L25, putative, expressed	3.25E-09	5.44	25740.8	2		*	
LOC_Os03g38000	40S ribosomal protein S3-1, putative, expressed	1.03E-10	34.21	25434.6	5	*		*
LOC_Os03g37970	ribosomal protein L13, putative, expressed	3.13E-06	29.81	24013.9	5	*	*	*
LOC_Os03g36930	DEAD-box ATP-dependent RNA helicase 34	6.53E-09	17.57	45628.4	5	*		*
LOC_Os03g31134	retrotransposon protein, putative, Ty1-copia subclass, expressed	3.69E-07	0.72	170954.0	2		*	*
LOC_Os03g31090	40S ribosomal protein S19, putative, expressed	3.69E-07	7.53	16386.7	2		*	*
LOC_Os03g28410	ribosomal protein S2, putative, expressed	1.02E-04	7.33	29347.0	2	*		*
LOC_Os03g27260	40S ribosomal protein S6, putative, expressed	7.42E-11	11.02	28058.5	2	*	*	*
LOC_Os03g26630	SAP domain containing protein, expressed	2.86E-05	4.06	78116.7	2			*
LOC_Os03g25450	h/ACA ribonucleoprotein complex subunit 4, putative, expressed	5.18E-11	22.34	64808.0	12	*	*	*
LOC_Os03g22880	nucleolar protein 5A, putative, expressed	5.42E-10	21.38	61331.4	16	*	*	*
LOC_Os03g22740	nucleolar protein NOP5-1, putative, expressed	6.37E-13	39.08	62631.3	17	*	*	*
LOC_Os03g22730	nucleolar protein NOP5-1, putative, expressed	1.29E-12	37.15	62458.2	19	*	*	*
LOC_Os03g22460	expressed protein	3.04E-08	45.71	7780.9	2		*	
LOC_Os03g22320	utp14 protein, putative, expressed	1.37E-13	2.60	99679.6	4		*	*
LOC_Os03g22180	60S ribosomal protein L18-3, putative, expressed	2.47E-08	14.97	21073.7	2			*
LOC_Os03g21940	60S ribosomal protein L19-3, putative, expressed	1.83E-10	19.71	24141.4	2		*	*
LOC_Os03g21530	AARP2CN domain containing protein, expressed	1.60E-08	5.93	133700.0	5	*		
LOC_Os03g18840	NUC189 domain containing protein, expressed	7.86E-06	12.40	68764.7	4			*
LOC_Os03g18580	40S ribosomal protein S7, putative, expressed	2.66E-04	13.54	22228.7	2		*	*
LOC_Os03g18570	40S ribosomal protein S7, putative, expressed	1.24E-11	25.00	22182.6	2	*	*	*
LOC_Os03g18510	expressed protein	1.05E-11	10.03	38234.4	2	*		*
LOC_Os03g18410	RNA polymerase Rpb3/Rpb11 dimerisation domain containing protein, expressed	1.52E-08	27.97	42097.3	7	*		*
LOC_Os03g17100	Probable histone H2A.5	9.03E-12	18.87	16401.2	2	*	*	*
LOC_Os03g17084	Histone H2B.1	6.10E-10	3.60	43872.5	2		*	*
LOC_Os03g15900	SH3 domain containing protein, expressed	7.38E-05	3.55	113383.2	3	*		
LOC_Os03g14530	S10/S20 domain containing ribosomal protein, putative, expressed	7.49E-06	10.24	13958.2	2		*	
LOC_Os03g13800	ribosomal protein L7Ae, putative, expressed	1.74E-10	43.75	13932.1	4	*	*	*
LOC_Os03g17000	NAD dependent epimerase/dehydratase family domain containing protein	2.47E-4	5.33	76065.1	2	*		
LOC_Os03g10340	40S ribosomal protein S3a, putative, expressed	3.64E-10	24.05	29727.4	5	*		*
LOC_Os03g08810	expressed protein	6.42E-07	14.22	24622.0	2			*
LOC_Os03g08440	ribosomal protein S2, putative, expressed	1.02E-04	7.43	29856.7	2	*		*
LOC_Os03g08050	elongation factor Tu, putative, expressed	8.33E-08	23.71	49292.7	9	*	*	*
LOC_Os03g08020	elongation factor Tu, putative, expressed	8.33E-08	23.71	49292.7	9	*	*	*
LOC_Os03g08010	elongation factor Tu, putative,	8.33E-08	23.71	49292.7	9	*	*	*

Supplement Table 1 cont. Nuclear Proteins Identified with Two or More Matched Peptides

Locus ID	Name	P (pro)	Coverage (%)	MW (Da)	Peptides	Method		
						A	B	C
LOC_Os03g06670	Probable histone H2A variant 1	1.43E-04	17.39	14555.8	2	*	*	*
LOC_Os03g05980	40S ribosomal protein S9-2, putative, expressed	6.32E-08	13.33	22673.0	2	*		*
LOC_Os03g05730	cell division control protein 48 homolog E, putative, expressed	9.51E-08	16.56	89743.5	11	*	*	*
LOC_Os03g05720	WD domain, G-beta repeat domain containing protein, expressed	6.42E-08	10.74	103407.7	8	*		
LOC_Os03g01970	THO complex subunit 1, putative, expressed	2.21E-06	4.70	71523.3	4	*		
LOC_Os03g01530	tubulin/FtsZ domain containing protein, putative, expressed	1.96E-06	7.16	50182.2	7	*		
LOC_Os02g57590	rRNA 2-O-methyltransferase fibrillarin 2, putative, expressed	1.37E-12	40.52	32402.7	10	*	*	*
LOC_Os02g56960	ribosomal protein, putative, expressed	3.72E-07	27.14	15030.7	4	*	*	*
LOC_Os02g56014	40S ribosomal protein S30, putative, expressed	4.62E-06	17.74	6931.1	2		*	
LOC_Os02g55010	expressed protein	1.27E-06	4.42	127150.9	3	*		
LOC_Os02g54340	26S protease regulatory subunit 7, putative, expressed	5.90E-08	12.97	44955.6	3	*		
LOC_Os02g52950	expressed protein	9.60E-06	28.23	31156.1	6	*		*
LOC_Os02g52250	SKIP/SNW domain containing protein, expressed	2.15E-07	14.33	67479.0	5		*	
LOC_Os02g50880	OsDegp3 - Putative Deg protease homologue, expressed	1.63E-10	21.34	61641.7	6	*		*
LOC_Os02g49270	NOL1/NOP2/sun family protein, putative, expressed	1.34E-05	13.56	77358.5	6	*		*
LOC_Os11g38900	Histone-lysine N-methyltransferase, H3 lysine-9 specific SUVH1, putativ	7.40E-06	8.00	89214.4	4	*		
LOC_Os02g47140	L11 domain containing ribosomal protein, putative, expressed	1.03E-07	35.54	17716.6	4	*	*	*
LOC_Os02g43930	chaperone protein dnaJ, putative, expressed	1.32E-03	8.81	47133.0	2	*		
LOC_Os02g40514	h/ACA ribonucleoprotein complex subunit 3, putative, expressed	4.88E-06	15.63	7538.6	3		*	
LOC_Os02g39140	helix-loop-helix DNA-binding domain containing protein, expressed	1.61E-04	15.33	30721.9	2		*	
LOC_Os02g38210	elongation factor Tu, putative, expressed	6.82E-07	15.63	50413.4	6	*		
LOC_Os02g37862	60S ribosomal protein L6, putative, expressed	8.96E-09	10.50	24258.1	3	*	*	*
LOC_Os02g37430	LSM domain containing protein, expressed	4.23E-07	27.19	12661.7	2			*
LOC_Os02g36974	F-box protein GID2	1.02E-07	18.13	21553.1	3	*		
LOC_Os02g33470	transposon protein, putative, Pong sub-class, expressed	4.18E-06	15.90	48160.7	5	*	*	*
LOC_Os02g33140	ribosomal protein, putative, expressed	1.35E-06	20.53	16419.7	2	*	*	*
LOC_Os02g32030	elongation factor, putative, expressed	5.50E-11	14.00	94021.0	8	*	*	*
LOC_Os02g28810	ribosomal protein, putative, expressed	4.26E-09	23.53	26886.9	5	*	*	*
LOC_Os02g21660	L1P family of ribosomal proteins domain containing protein, expressed	1.79E-07	7.21	23592.7	2			*
LOC_Os02g18660	expressed protein	1.09E-08	10.21	61523.7	8	*	*	*
LOC_Os02g18550	40S ribosomal protein S3a, putative, expressed	3.64E-10	13.03	29759.4	3			*
LOC_Os02g18380	60S ribosomal protein L27-3, putative, expressed	3.01E-08	9.49	15578.5	3		*	
LOC_Os02g16640	proline-rich protein HaeIII subfamily 1 precursor, putative, expressed	9.35E-07	7.89	23883.2	2		*	*
LOC_Os02g13990	U2 small nuclear ribonucleoprotein A, putative, expressed	5.23E-08	22.18	31999.0	4	*		*
LOC_Os02g13530	40S ribosomal protein S24,	1.72E-04	17.39	15748.5	2		*	

Supplement Table 1 cont. Nuclear Proteins Identified with Two or More Matched Peptides

Locus ID	Name	P (pro)	Coverage (%)	MW (Da)	Peptides	Method		
						A	B	C
LOC_Os02g10640	26S protease regulatory subunit, putative, expressed	9.20E-06	2.75	44586.0	2	*		
LOC_Os02g10080	zinc finger C-x8-C-x5-C-x3-H type family protein, expressed	3.70E-05	5.19	23952.2	2		*	
LOC_Os02g08370	ubiquitin carboxyl-terminal hydrolase, family 1, putative, expressed	3.99E-04	9.12	37617.1	2	*		
LOC_Os02g07890	60S ribosomal protein L27a-3, putative, expressed	6.11E-10	19.44	15795.5	2		*	*
LOC_Os02g07260	phosphoglycerate kinase protein, putative, expressed	9.27E-11	30.19	39481.5	13	*	*	*
LOC_Os02g06700	ribosomal protein, putative, expressed	1.35E-06	20.67	16362.7	2	*	*	*
LOC_Os02g06584	zinc finger C-x8-C-x5-C-x3-H type family protein, expressed	5.63E-06	2.58	49077.2	2	*		
LOC_Os02g06370	whirly transcription factor domain containing protein, expressed	3.39E-04	6.58	25197.4	2	*		
LOC_Os02g05330	DEAD-box ATP-dependent RNA helicase, putative, expressed	7.44E-08	17.15	47137.6	7	*	*	
LOC_Os02g04480	cleavage and polyadenylation specificity factor, putative, expressed	3.80E-07	10.30	94281.8	5	*		*
LOC_Os02g04050	chromosome segregation protein, putative, expressed	6.32E-09	6.48	79786.0	3	*		
LOC_Os02g04040	RecF/RecN/SMC N terminal domain containing protein, expressed	1.83E-10	20.85	56694.9	7	*		
LOC_Os02g02410	DnaK family protein, putative, expressed	1.16E-08	19.70	73389.7	9	*	*	*
LOC_Os02g01740	U5 small nuclear ribonucleoprotein 200 kDa helicase, putative, expressed	2.25E-08	7.58	246612.2	10	*		
LOC_Os02g01560	40S ribosomal protein S4, putative, expressed	1.59E-13	17.36	29864.7	3	*	*	*
LOC_Os02g01332	ribosomal protein L6, putative, expressed	4.04E-09	34.38	21643.0	4	*		*
LOC_Os02g01250	LSM domain containing protein, expressed	1.01E-06	28.03	14280.6	2		*	
LOC_Os01g69970	WD domain, G-beta repeat domain containing protein, expressed	2.93E-04	10.27	53050.5	3			*
LOC_Os01g68950	ubiquitin family domain containing protein, expressed	1.62E-04	25.00	10928.2	2		*	
LOC_Os01g68320	DEAD-box ATP-dependent RNA helicase 30, putative, expressed	1.05E-05	15.92	72188.4	8	*		
LOC_Os01g67134	ribosomal L18p/L5e family protein, putative, expressed	2.11E-09	11.96	34326.5	4	*		*
LOC_Os01g64640	Histone H3.2	1.12E-13	23.53	15267.8	2		*	*
LOC_Os01g64090	L1P family of ribosomal proteins domain containing protein, expressed	1.79E-07	6.30	27101.1	2			*
LOC_Os01g62230	Histone H2B.11	6.10E-10	10.79	15365.9	2		*	*
LOC_Os01g62040	ruvB-like, putative, expressed	4.75E-09	29.67	49668.9	8	*		
LOC_Os01g61920	Histone H4	8.02E-09	24.27	11409.3	7	*	*	*
LOC_Os01g59990	ribosomal protein L24, putative, expressed	1.24E-07	14.81	18453.5	2		*	*
LOC_Os01g59500	U3 small nucleolar RNA-associated protein 11, putative, expressed	4.07E-08	19.65	27511.5	5	*	*	*
LOC_Os01g54870	60S ribosomal protein L18a, putative, expressed	5.27E-09	17.98	21400.0	4	*	*	*
LOC_Os01g53900	elongation factor, putative, expressed	4.47E-04	6.30	91999.7	4	*		
LOC_Os01g52490	40S ribosomal protein S24, putative, expressed	4.47E-06	26.09	15718.5	3		*	
LOC_Os01g51300	WD domain, G-beta repeat domain containing protein, expressed	1.44E-08	17.00	50110.7	8	*		
LOC_Os01g49290	Guanine nucleotide-binding protein subunit beta-like protein A	2.51E-06	8.98	36231.5	4	*		*

Supplement Table 1 cont. Nuclear Proteins Identified with Two or More Matched Peptides

Locus ID	Name	P (pro)	Coverage (%)	MW (Da)	Peptides	Method		
						A	B	C
LOC_Os01g47660	60S ribosomal protein L18a, putative, expressed	5.27E-09	17.98	21386.0	4	*	*	*
LOC_Os01g46060	NUC189 domain containing protein, expressed	4.96E-10	11.86	66545.8	4	*		*
LOC_Os01g45190	DEAD-box ATP-dependent RNA helicase 2	6.53E-09	17.57	45598.4	5	*		*
LOC_Os01g42820	RNA recognition motif containing protein, putative, expressed	1.48E-08	6.26	44269.4	2	*		
LOC_Os01g36890	DEAD-box ATP-dependent RNA helicase, putative, expressed	2.24E-07	12.50	39062.0	2	*		
LOC_Os01g36860	DEAD-box ATP-dependent RNA helicase 40, putative, expressed	7.35E-09	16.47	64797.5	8	*		
LOC_Os01g34200	AATF, putative, expressed	6.29E-05	10.26	40093.8	2			*
LOC_Os01g33050	ribosomal protein L24, putative, expressed	5.60E-08	25.00	19884.0	3	*	*	*
LOC_Os01g33030	Brix domain containing protein, putative, expressed	9.95E-04	4.52	35213.8	2	*		*
LOC_Os01g27730	GTPase of unknown function domain containing protein, putative, expressed	3.26E-06	5.54	82783.3	3	*	*	*
LOC_Os01g25610	40S ribosomal protein S4, putative, expressed	3.78E-07	10.19	29807.6	2	*	*	*
LOC_Os01g24690	60S ribosomal protein L23A, putative, expressed	3.84E-05	5.84	15511.4	2		*	*
LOC_Os01g21940	WD domain, G-beta repeat domain containing protein, expressed	5.16E-09	5.22	58198.9	2	*		
LOC_Os01g21590	homeodomain, putative, expressed	1.79E-07	12.61	57857.2	3	*	*	
LOC_Os01g16870	argonaute, putative, expressed	5.13E-05	3.98	100638.4	2	*		
LOC_Os01g16290	DNA gyrase subunit B	9.47E-06	4.94	80468.3	2	*		
LOC_Os01g16220	Sad1 / UNC-like C-terminal domain containing protein, putative, expressed	4.02E-06	7.91	48429.5	2			*
LOC_Os01g16010	BCAS2 protein, putative, expressed	6.17E-11	16.80	28832.6	2	*	*	
LOC_Os01g14950	Importin subunit alpha-1a	1.03E-05	15.02	57570.3	4	*		
LOC_Os01g13730	WD domain, G-beta repeat domain containing protein, expressed	2.21E-04	15.27	51980.9	4	*		
LOC_Os06g51220	HMG1/2, putative, expressed	4.29E-05	5.70	17099.7	4	*		
LOC_Os01g08970	SSRP1-like FACT complex subunit, putative, expressed	2.10E-09	4.84	71333.5	2			*
LOC_Os01g08770	WD domain, G-beta repeat domain containing protein, expressed	2.68E-07	22.10	60503.3	8	*		*
LOC_Os01g07810	protein of unknown function domain containing protein, expressed	1.74E-06	8.40	14091.7	3		*	*
LOC_Os01g06290	splicing factor, arginine/serine-rich, putative, expressed	1.73E-05	8.70	30987.5	2		*	
LOC_Os01g06010	Histone H2B.5	4.21E-11	9.68	16757.5	2		*	*
LOC_Os01g05970	OsFBO1 - F-box and other domain containing protein, expressed (Histone H2B.7/H2B.6)	6.10E-10	9.80	16472.2	2		*	*
LOC_Os01g05900	Histone H2B.10	1.15E-11	14.38	16548.3	3		*	*
LOC_Os01g05630	Histone H2B.4	1.15E-11	14.38	16502.2	3		*	*
LOC_Os01g05610	Histone H2B.3	1.15E-11	14.38	16530.2	3		*	*
LOC_Os01g01510	sas10/Utp3 family protein, expressed	2.96E-06	5.12	73289.0	2	*		
LOC_Os01g01060	40S ribosomal protein S5, putative, expressed	7.37E-08	14.00	22226.5	2	*		*
LOC_Os07g41190	WD domain, G-beta repeat domain	2.06E-09	19.57	61730.1	8	*		
LOC_Os06g40950	DNA-directed RNA polymerase I subunit RPA1, putative, expressed	1.22E-06	6.80	39332.1	2	*		

a) **Locus ID**: TIGR Locus ID Number
b) **Name**: Annotation
c) **P(pro)**: the probability of a random match for this protein in the *Oryza sativa* database
d) **Coverage (%)**: the percent of the amino acid sequence that has been verified with MS/MS
e) **MW (Da)**: predicted molecular mass
f) **Peptide**: Total Number of peptides assigned to this protein

g) **Method**: These three columns indicate that the protein was identified using which of the three protein extraction methods. (A) Phenol extraction; (B) Phenol-Acid double extraction; (C) Acid extraction. * indicates that this protein was identified with 2 or more peptides in the sample extracted by the corresponding procedure, respectively.

Supplement Table 2. Peptides Identified in Reverse Database Searches			
**Peptide**	**MH+**	**z**	**P (pro)**
K.AEQISTIENFNKTNPIGLCR.M	2249.53320	2	1.04E-04
K.AVC*GELCEPPMGGPMKALNR.V	2131.62185	2	2.39E-03
K.CVNLAFAVEVAAGAAVRVR.R	1917.26843	2	2.14E-03
K.DLSLLRQTGYGAIK.A	1535.76990	2	9.38E-03
K.EIATKLLELGEK.N	1344.57910	2	7.56E-06
K.ELLVDHHKALLADK.R	1602.85950	2	1.79E-04
K.EWKSFPLVGR.E	1219.41675	2	1.14E-03
K.FGC*AGGNDDPGAGNPAGYWTAR.A	2212.37224	2	2.05E-01
K.FSDIVSIGRNCK.G	1339.54614	2	4.31E-03
K.GDFIGILGLRVHAGGEEGR.K	1954.17896	2	5.33E-03
K.GDKDVFELTNTLVC*PAEFR.L	2212.53664	2	8.26E-03
K.GRAGGGGVGEGGAASAASLPR.H	1755.87415	2	7.30E-03
K.IDQLNGALR.S	1000.13422	2	7.93E-03
K.KDAEPSPIGGEDK.L	1343.42188	2	3.25E-03
K.KDSDVEELAYR.A	1325.40649	2	2.83E-03
K.KSLQDLIPQVEAK.T	1469.70825	2	6.77E-05
K.LNENEDLLLNEPK.L	1541.68494	2	5.72E-04
K.LTGAIFLLVAARLLK.N	1600.02820	2	3.40E-03
K.M#SCVTLLLPVLGMEVILWPSFVK.S	2609.25186	2	8.32E-03
K.MC*EKLAGDLHAAR.E	1472.78769	2	9.13E-03
K.MDLEANC*EQLQAMLDIK.R	2023.41029	2	4.41E-03
K.NENM#NTRENENVNTPENM#	2215.23888	2	7.51E-03
K.NRISGTVDGLAIVK.G	1443.67407	2	2.29E-03
K.QC*DEC*GEGIAVKAQDVSLCK.L	2211.62081	2	2.10E-03
K.RGVSPQSGSAEEEQNGEVDR.E	2132.14868	2	1.37E-03
K.SAGNWVELQISGAIRFALK.E	2061.37256	2	6.06E-03
K.SEASSLKIALNR.K	1289.46362	2	3.61E-03
K.SFNDLNITYAGSGGGGPK.A	1755.86621	2	3.64E-03
K.SMQSPPDSRTIVQIVR.R	1815.08813	2	3.40E-03
K.SSDEEAVAAAAAAR.K	1319.36035	2	2.64E-04
K.SYDGVVIGAIEGNSPLNYQVEDK.A	2468.65796	2	7.35E-03
K.TC*TLAIKVDGSITIPGGPSLMK.M	2260.77243	2	2.81E-03
K.TSTVDVGLGTDRGTAAELQAGGSGR.R	2377.51172	2	2.08E-03
K.VEEIERGIGHADAQLIR.E	1907.12061	2	8.58E-03
K.VFVQGNAGQASLLKALQEGAK.R	2130.43311	2	2.91E-03
K.VLEVWQDYDIDEPCLKYLASR.K	2556.87500	2	4.00E-04
K.VM#SEAFDRFFEAAYMGTAQR.R	2360.61238	2	1.98E-03
K.VVIDDVYAEVNRGLQNC*LVK.Y	2305.70835	2	2.87E-03
R.AAVEFRCAFYANMLATQTFLVSSYGLR.L	3031.50049	2	2.87E-04
R.AGVSPRVAEMVQEM	1504.75806	2	4.91E-03
R.AIGILVEPNKCVQDSFSSR.S	2064.35181	2	7.75E-03
R.AQDRELIEAHTVAGGGGR.P	1837.97522	2	5.82E-05
R.AQHLM#DDDNC*DIHRK.E	1901.10152	2	3.12E-03
R.ASETEVVELAR.E	1204.31238	2	9.12E-03
R.C*TYIEYLARYVEVLCGDGR.P	2281.66561	2	9.01E-03
R.DCGVLQWLLKDSDVENLGELR.Y	2403.69727	2	5.73E-04
R.DGSEGVSINLVLKQVK.I	1686.93164	2	1.34E-03
R.EELATARAELAVER.R	1558.71875	2	7.62E-03
R.EFPC*MERLFAPGC*LK.T	1856.36248	2	5.97E-03
R.EGEDVVAKAADR.F	1260.33606	1	6.28E-03
R.ELEVEDVGKGAAVEK.M	1573.72693	2	7.41E-03
R.ELISASIKEFEER.K	1551.72302	2	2.26E-03
R.FAGGKSILVQNGSLSK.G	1606.84814	2	3.84E-03
R.FGLTSSGVDYLVEFTR.Q	1791.98157	2	2.85E-03
R.GEVASVAASDGGAM#	1254.30809	2	8.36E-03
R.GLNGKTCADIVEGGNLAMFYFHK.Q	2486.85449	2	3.74E-04
R.GVELDIM#M#KLLQYISR.D	1974.33433	2	4.22E-05
R.HLFLLLLVLEETDLNEICR.V	2284.70361	2	7.70E-03
R.HPRVFAAILSPMSELFPVR.G	2168.59277	2	3.13E-03
R.IELNFGECIERLK.N	1564.83154	2	7.30E-03

Supplement Table 2 cont. Peptides Identified in Reverse Database Searches			
**Peptide**	**MH+**	**z**	**P (pro)**
R.IGEIENNNGQLIVSKDFTK.R	2120.34888	2	2.93E-03
R.INQTESVLLWALVIK.L	1728.06848	2	4.53E-05
R.KSASSEMVGLQNVTETTSAIR.H	2210.45215	2	3.08E-03
R.LC*C*KIAAPINTNVLYSSWNICR.T	2598.20197	2	1.22E-03
R.LDLTELCQLM#FK.G	1486.78050	2	1.44E-01
R.LGDSFTKGPLLWYVK.A	1725.02307	2	6.42E-03
R.LLRYVSEVNNNNK.G	1563.74023	2	3.19E-04
R.LMASIFASKGVNASGLIYVDR.G	2213.58569	2	3.64E-03
R.LMAVVQLMNPR.K	1272.60950	2	2.07E-03
R.LSVYEGNDEER.Y	1311.33667	2	2.63E-04
R.LTLLVSPLSVR.A	1198.48047	2	7.89E-03
R.NVAAEVRAVPGHEPLVGGCC*GGR.A	2306.68316	2	6.24E-03
R.PHRLLYLGAVLAVVR.P	1678.06067	2	1.78E-03
R.PTM#LFLPTAAAGAGAGDDEVERR.R	2378.60574	2	9.69E-03
R.QC*VVC*LAVFEAIER.K	1695.13761	2	9.76E-03
R.QRGSGSAVATALGGR.C	1388.51538	2	9.44E-03
R.RMTLQIER.D	1047.25757	2	1.62E-03
R.RQAAAGAGAAAGASSGK.E	1402.49890	2	8.12E-06
R.RVFWESTNPNSAEFM#TK.E	2077.26397	2	5.33E-03
R.SSPTTTAVLEEPKHK.R	1625.80505	2	1.45E-03
R.STDPDSLPARR.N	1215.29846	2	1.02E-02
R.TEIDKM#SSVFDK.A	1432.57992	2	6.27E-03
R.TGVPLILKWVLEGESFDK.P	2032.36792	2	4.35E-03
R.VAHQVAPDGGVGAGPVRVVHVAGR.V	2306.61572	2	8.12E-03
R.VDLAELNQLEKWK.E	1586.81372	2	4.46E-03
R.VIPIKELTIQNLR.S	1537.87207	2	2.52E-03
R.VVAAVVYQQAATWRR.R	1718.98340	2	6.07E-03
R.WHERSGANAPLVLLLR.R	1833.12964	2	7.96E-03
R.WQTENSAVLEK.M	1305.41846	1	4.96E-03

a)Sequence: Identified peptide sequence
b)MH+: Protonated Molecular Mass of the peptide
c) Z: Charge number
d) P(pro): The probability of a random match to the identified peptide
e) *:Cystine with carbamidomethylation
f) #: methionine with oxidation

Supplement Table 3. Differentially Regulated Nuclear Proteins					
**Locus ID**	**Annotation**	**Method(Fold-Change)**			**Regulation**
		**A**	**B**	**C**	
LOC_Os01g07810	protein protein of unknown function domain containing protein, expres			(*)	up
LOC_Os01g08970	protein SSRP1-like FACT complex subunit, putative, expressed			(*)	up
LOC_Os01g10820	ribosomal protein L5, putative, expressed	(2.03)			up
LOC_Os01g13730	WD domain, G-beta repeat domain containing protein, expressed	(*)			up
LOC_Os01g16870	argonaute, putative, expressed	(*)			up
LOC_Os01g21590	homeodomain, putative, expressed	(*)			up
LOC_Os01g21940	WD domain, G-beta repeat domain containing protein, expressed	(*)			up
LOC_Os01g27730	GTPase of unknown function domain containing protein, putative, expressed		(*)	(2.87)	up
LOC_Os01g33030	Brix domain containing protein, putative, expressed	(*)		(2.71)	up
LOC_Os01g36860	DEAD-box ATP-dependent RNA helicase 40, putative, expressed	(27.14)			up
LOC_Os01g45190	protein DEAD-box ATP-dependent RNA helicase, putative, expressed			(*)	up
LOC_Os01g46060	NUC189 domain containing protein, expressed	(*)		(1.97)	up
LOC_Os01g62040	ruvB-like, putative, expressed	(*)			up
LOC_Os01g64640	histone H3, putative, expressed		(28.44)		up
LOC_Os01g68320	DEAD-box ATP-dependent RNA helicase 30, putative, expressed	(6.27)			up
LOC_Os01g69970	protein WD domain, G-beta repeat domain containing protein, expressed			(4.17)	up
LOC_Os02g01740	U5 small nuclear ribonucleoprotein 200 kDa helicase, putative, expressed	(*)			up
LOC_Os02g04040	RecF/RecN/SMC N terminal domain containing protein, expressed	(*)			up
LOC_Os02g04050	chromosome segregation protein, putative, expressed	(*)			up
LOC_Os02g04480	cleavage and polyadenylation specificity factor, putative, expressed	(*)			up
LOC_Os02g06584	zinc finger C-x8-C-x5-C-x3-H type family protein, expressed	(*)			up
LOC_Os02g13990	U2 small nuclear ribonucleoprotein A, putative, expressed	(*)			up
LOC_Os02g16640	protein proline-rich protein HaeIII subfamily 1 precursor, putative,			(*)	up
LOC_Os02g18660	expressed protein	(*)	(14.32)		up
LOC_Os02g49270	NOL1/NOP2/sun family protein, putative, expressed	(*)			up
LOC_Os02g50880	OsDegp3 - Putative Deg protease homologue, expressed	(*)			up
LOC_Os09g23730	HMG-Y-related protein A, putative	(1.38)			up
LOC_Os05g03740	Transcription factor TF2, putative	(*)			up
LOC_Os02g57590	rRNA 2-O-methyltransferase fibrillarin 2, putative, expressed	(6.09)	(3.81)	(1.75)	up
LOC_Os03g01530	tubulin/FtsZ domain containing protein, putative, expressed	(33.65)			up
LOC_Os03g01970	THO complex subunit 1, putative, expressed	(*)			up
LOC_Os03g05720	WD domain, G-beta repeat domain containing protein, expressed	(*)			up
LOC_Os03g13800	ribosomal protein L7Ae, putative, expressed	(2.2)	(6.87)	(3.15)	up
LOC_Os03g15900	SH3 domain containing protein, expressed	(*)			up
LOC_Os03g18410	DNA-directed RNA polymerase subunit, putative, expressed	(5)			up
LOC_Os03g18510	expressed protein	(*)		(2.11)	up
LOC_Os03g18840	protein NUC189 domain containing protein, expressed			(1.58)	up
LOC_Os03g21530	AARP2CN domain containing protein, expressed	(*)			up
LOC_Os03g22320	utp14 protein, putative, expressed		(5.44)		up
LOC_Os03g22730	nucleolar protein NOP5-1, putative, expressed	(3.47)			up
LOC_Os03g22740	nucleolar protein NOP5-1, putative, expressed	(3.6)			up
LOC_Os03g25450	h/ACA ribonucleoprotein complex subunit 4, putative, expressed	(16.19)	(*)		up
LOC_Os03g36930	protein DEAD-box ATP-dependent RNA helicase, puta			(*)	up

Supplement Table 3 cont. Differentially Regulated Nuclear Proteins					
**Locus ID**	**Annotation**	**Method(Fold-Change)**			**Regulation**
		**A**	**B**	**C**	
LOC_Os03g49210	BRCA1 C Terminus domain containing protein, expressed	(*)			up
LOC_Os03g51200	Core histone H2A/H2B/H3/H4 domain containing protein, putative, expressed	(*)	(26.6)		up
LOC_Os03g52470	WD domain, G-beta repeat domain containing protein, expressed	(*)			up
LOC_Os03g53220	U5 small nuclear ribonucleoprotein 200 kDa helicase, putative, expressed	(*)			up
LOC_Os03g58530	ES43 protein, putative, expressed	(*)			up
LOC_Os03g58810	DEAD-box ATP-dependent RNA helicase, putative, expressed	(*)		(*)	up
LOC_Os03g63690	BCCIP, putative, expressed	(*)		(4.02)	up
LOC_Os04g25550	protein FACT complex subunit SPT16, putative, expressed			(*)	up
LOC_Os04g31320	SWIB/MDM2 domain containing protein, expressed	(29.35)			up
LOC_Os04g36890	peptidyl-prolyl cis-trans isomerase, FKBP-type, putative, expressed	(*)	(2.23)	(1.7)	up
LOC_Os04g41040	DNA-directed RNA polymerases I, II, and III subunit RPABC1, putative, expr	(*)			up
LOC_Os04g45940	transcription factor like protein, putative, expressed	(*)		(17.17)	up
LOC_Os04g48060	RFC2 - Putative clamp loader of PCNA, replication factor C subunit 2, expr	(*)			up
LOC_Os04g49580	nucleolar complex protein, putative, expressed	(*)		(1.72)	up
LOC_Os04g50660	WD domain, G-beta repeat domain containing protein, expressed	(*)			up
LOC_Os04g52200	RNA recognition motif containing protein, putative, expressed	(*)		(*)	up
LOC_Os04g52960	nucleolin, putative, expressed	(*)	(3.24)	(3.5)	up
LOC_Os04g56350	nucleolar protein,Nop52 containing protein, expressed	(*)			up
LOC_Os04g56590	ATP/GTP binding protein, putative, expressed	(8.55)			up
LOC_Os04g56720	RCC2, putative, expressed	(*)		(1.96)	up
LOC_Os04g58830	ribosome biogenesis regulatory protein, putative, expressed	(6.77)	(1.59)		up
LOC_Os05g04850	RNA recognition motif containing protein, putative, expressed	(*)			up
LOC_Os05g08360	rRNA 2-O-methyltransferase fibrillarin 2, putative, expressed	(4.63)	(4.21)	(1.92)	up
LOC_Os05g09620	SCC3, putative, expressed	(*)			up
LOC_Os05g11710	ribosomal protein L5, putative, expressed	(2.03)			up
LOC_Os05g22920	digestive organ expansion factor, putative, expressed	(*)			up
LOC_Os09g30412	heat shock protein, putative, expressed	(2.85)	(*)		up
LOC_Os05g44320	WD domain, G-beta repeat domain containing protein, expressed	(*)			up
LOC_Os05g48820	DDT, putative, expressed	(*)			up
LOC_Os05g51850	AT hook-containing DNA-binding protein, putative, expressed		(3.85)		up
LOC_Os06g03780	NUC153 domain containing protein, expressed	(*)			up
LOC_Os06g05350	whirly transcription factor domain containing protein, expressed	(*)			up
LOC_Os06g06480	Core histone H2A/H2B/H3/H4 domain containing protein, putative, expressed		(28.44)		up
LOC_Os06g06880	Ser/Thr protein phosphatase family protein, putative, expressed	(*)			up
LOC_Os06g08770	ruvB-like 2, putative, expressed	(*)			up
LOC_Os06g10710	expressed protein	(*)			up
LOC_Os06g12780	OsDegp10 - Putative Deg protease homologue, expressed	(*)			up
LOC_Os06g16290	ribosomal protein L7Ae, putative, expressed	(*)	(2.22)		up
LOC_Os06g17840	expressed protein	(*)	(6.49)	(*)	up
LOC_Os06g30320	NOC3 - Putative nucleolar complex subunit 3, expressed	(*)			up
LOC_Os06g43690	WD repeat-containing protein, putative, expressed	(*)			up
LOC_Os06g46890	zinc finger C-x8-C-x5-C-x3-H type family protein,	(*)	(2.61)		up

Supplement Table 3 cont. Differentially Regulated Nuclear Proteins					
**Locus ID**	**Annotation**	**Method(Fold-Change)**			**Regulation**
		**A**	**B**	**C**	
LOC_Os06g48230	DNA-directed RNA polymerases I, II, and III subunit RPABC3, putative, expr	(*)			up
LOC_Os06g48350	CPuORF14 - conserved peptide uORF-containing transcript, expressed	(*)	(*)	(*)	up
LOC_Os06g48355	expressed protein	(*)	(*)	(*)	up
LOC_Os07g03240	RNA recognition motif containing protein, putative, expressed	(*)			up
LOC_Os07g06980	histone deacetylase, putative, expressed	(29.53)		(3.68)	up
LOC_Os07g07580	ROOT HAIRLESS 1, putative, expressed	(*)		(*)	up
LOC_Os07g08880	ES43 protein, putative, expressed	(*)			up
LOC_Os07g08960	RNA recognition motif containing protein, expressed	(*)			up
LOC_Os07g10350	S1 RNA binding domain containing protein, expressed	(*)			up
LOC_Os07g12320	WD domain, G-beta repeat domain containing protein, expressed	(*)			up
LOC_Os07g12910	PHD finger protein, putative, expressed	(*)			up
LOC_Os07g20580	protein DEAD-box ATP-dependent RNA helicase, putative, expressed			(4.22)	up
LOC_Os07g32350	WD domain, G-beta repeat domain containing protein, expressed	(*)		(2.01)	up
LOC_Os07g33340	DEAD-box ATP-dependent RNA helicase, putative, expressed	(*)		(2.27)	up
LOC_Os07g36130	core histone H2A/H2B/H3/H4, putative, expressed	(*)	(2.03)		up
LOC_Os07g36140	core histone H2A/H2B/H3/H4, putative, expressed	(1.8)	(7.46)	(1.9)	up
LOC_Os07g40930	WD repeat-containing protein 12, putative, expressed	(*)			up
LOC_Os07g41190	WD domain, G-beta repeat domain containing protein, expressed	(7.76)			up
LOC_Os07g41740	PHD finger protein, putative, expressed	(*)			up
LOC_Os07g43980	DEAD-box ATP-dependent RNA helicase, putative, expressed	(*)			up
LOC_Os07g47420	protein 60S ribosome subunit biogenesis protein NIP7, putative, expre			(11.47)	up
LOC_Os08g04280	actin, putative, expressed	(*)			up
LOC_Os08g05840	DNA topoisomerase 1, putative, expressed	(*)			up
LOC_Os08g09350	protein gar2, putative, expressed			(*)	up
LOC_Os08g29650	RNA recognition motif containing protein, expressed	(*)	(3)		up
LOC_Os08g31240	protein FACT complex subunit SPT16, putative, expressed			(*)	up
LOC_Os08g31810	RNA recognition motif containing protein, putative, expressed	(19.25)	(2.44)	(1.57)	up
LOC_Os08g32090	DEAD-box ATP-dependent RNA helicase, putative, expressed	(*)	(*)		up
LOC_Os08g33100	core histone H2A/H2B/H3/H4, putative,	(*)	(2.39)		up
LOC_Os08g36450	transcription regulator, putative, expressed	(*)			up
LOC_Os08g42920	60S ribosomal protein L7, putative, expressed	(16.12)			up
LOC_Os09g02284	DNA-directed RNA polymerase subunit, putative, expressed	(4.17)			up
LOC_Os09g02810	CCAAT/enhancer-binding protein, putative, expressed	(*)			up
LOC_Os09g10770	OsTOP6B - Topoisomerase 6 subunit B, expressed	(*)			up
LOC_Os09g15770	CPuORF13 - conserved peptide uORF-containing transcript, expressed	(*)	(*)	(*)	up
LOC_Os09g15775	expressed protein	(*)	(*)	(*)	up
LOC_Os09g24260	WD domain, G-beta repeat domain containing protein, expressed	(*)			up
LOC_Os09g24820	protein ZF-HD protein dimerisation region containing protein, express			(*)	up
LOC_Os09g27850	transcription regulator, putative, expressed	(*)			up
LOC_Os09g36160	LRP1, putative, expressed	(14.85)	(5.02)		up
LOC_Os10g28230	Core histone H2A/H2B/H3/H4 domain containing protein, putative, expressed	(1.61)			up
LOC_Os10g31520	ribosomal RNA assembly protein mis3, putative, expressed	(*)			up
LOC_Os10g32880	WD domain, G-beta repeat domain containing protein, expressed	(3.32)			up

Supplement Table 3 cont. Differentially Regulated Nuclear Proteins					
**Locus ID**	**Annotation**	**Method(Fold-Change)**			**Regulation**
		**A**	**B**	**C**	
LOC_Os10g35290	DNA-directed RNA polymerase I subunit RPA2, putative, expressed	(*)			up
LOC_Os11g01420	ribosomal protein L10, putative, expressed	(24.86)			up
LOC_Os11g07470	expressed protein	(*)			up
LOC_Os11g37080	h/ACA ribonucleoprotein complex subunit 1-like protein 1, putative, expres	(2.77)	(2.87)		up
LOC_Os11g43890	WD domain, G-beta repeat domain containing protein, expressed	(*)			up
LOC_Os12g01430	ribosomal protein L10, putative, expressed	(21.47)			up
LOC_Os12g06910	nucleolar protein family 6, putative, expressed	(*)			up
LOC_Os12g25120	core histone H2A/H2B/H3/H4, putative, expressed	(*)	(2.03)		up
LOC_Os12g34510	Core histone H2A/H2B/H3/H4 domain containing protein, putative, expressed	(6.81)	(5.14)		up
LOC_Os12g41620	WD domain, G-beta repeat domain containing protein, expressed	(*)			up
LOC_Os12g41930	SRP40, C-terminal domain containing protein, expressed		(2.45)		up
LOC_Os12g42150	WD domain, G-beta repeat domain containing protein, expressed	(*)			up
LOC_Os12g44390	RecF/RecN/SMC N terminal domain containing protein, expressed	(*)			up
LOC_Os10g35280	nucleolar complex protein 2, putative, expressed	(*)			up
LOC_Os01g16220	protein Sad1 / UNC-like C-terminal domain containing protein, putativ			(*)	down
LOC_Os01g24690	protein 60S ribosomal protein L23A, putative, expressed			(2.68)	down
LOC_Os01g25610	protein 40S ribosomal protein S4, putative, expressed			(7.89)	down
LOC_Os01g42820	RNA recognition motif containing protein, putative, expressed	(*)			down
LOC_Os01g49290	protein WD repeat-containing protein, putative, expressed			(18.37)	down
LOC_Os01g53900	elongation factor, putative, expressed	(6.51)			down
LOC_Os01g59500	protein U3 small nucleolar RNA-associated protein 11, putative, expre			(*)	down
LOC_Os01g59990	ribosomal protein L24, putative, expressed		(7.95)	(24.46)	down
LOC_Os01g68950	ubiquitin family domain containing protein, expressed		(*)		down
LOC_Os02g01332	protein ribosomal protein L6, putative, expressed			(4.45)	down
LOC_Os02g05330	DEAD-box ATP-dependent RNA helicase, putative, expressed	(2.27)	(*)		down
LOC_Os02g07260	phosphoglycerate kinase protein, putative, expressed	(5.76)	(*)	(*)	down
LOC_Os02g07890	protein 60S ribosomal protein L27a-3, putative, expressed			(3.98)	down
LOC_Os02g10080	zinc finger C-x8-C-x5-C-x3-H type family protein, expressed		(*)		down
LOC_Os02g10640	26S protease regulatory subunit, putative, expressed	(*)			down
LOC_Os02g18380	60S ribosomal protein L27-3, putative, expressed		(2.1)		down
LOC_Os02g18550	protein 40S ribosomal protein S3a, putative, expressed			(4.85)	down
LOC_Os02g28810	protein ribosomal protein, putative, expressed			(3.69)	down
LOC_Os02g32030	elongation factor, putative, expressed		(*)	(*)	down
LOC_Os02g36974	14-3-3 protein, putative, expressed	(3.85)			down
LOC_Os02g37430	protein LSM domain containing protein, expressed			(2.37)	down
LOC_Os02g39140	helix-loop-helix DNA-binding domain containing protein, expressed		(*)		down
LOC_Os02g40514	h/ACA ribonucleoprotein complex subunit 3, putative, expressed		(3.72)		down
LOC_Os06g51220	HMG1/2, putative, expressed	(4.12)			down
LOC_Os02g52250	SKIP/SNW domain containing protein, expressed		(13.66)		down
LOC_Os02g54340	26S protease regulatory subunit 7, putative, expressed	(*)			down
LOC_Os02g56014	40S ribosomal protein S30, putative, expressed		(*)		down
LOC_Os03g05730	cell division control protein 48 homolog E, putative, expressed	(7.46)	(*)		down
LOC_Os03g08440	protein ribosomal protein S2, putative, expressed			(*)	down
LOC_Os03g08810	protein expressed protein			(3.06)	down

Supplement Table 3 cont. Differentially Regulated Nuclear Proteins					
**Locus ID**	**Annotation**	**Method(Fold-Change)**			**Regulation**
		**A**	**B**	**C**	
LOC_Os03g17000	NAD dependent epimerase/dehydratase family domain containing protein, expressed	(*)			down
LOC_Os03g18570	protein 40S ribosomal protein S7, putative, expressed			(5.11)	down
LOC_Os03g18580	40S ribosomal protein S7, putative, expressed		(2.43)	(3.1)	down
LOC_Os03g21940	60S ribosomal protein L19-3, putative, expressed		(2.24)	(*)	down
LOC_Os03g22180	protein 60S ribosomal protein L18-3, putative, expressed			(14.19)	down
LOC_Os03g22460	expressed protein		(*)		down
LOC_Os03g27260	protein 40S ribosomal protein S6, putative, expressed			(4.2)	down
LOC_Os03g31090	40S ribosomal protein S19, putative, expressed		(*)	(*)	down
LOC_Os03g31134	retrotransposon protein, putative, Ty1-copia subclass, expressed		(*)	(*)	down
LOC_Os03g38000	protein 40S ribosomal protein S3-1, putative, expressed			(*)	down
LOC_Os03g41612	ribosomal protein L25, putative, expressed		(5.85)		down
LOC_Os03g46490	40S ribosomal protein S21, putative, expressed		(*)		down
LOC_Os03g50090	transposon protein, putative, CACTA, En/Spm sub-class, expressed	(*)			down
LOC_Os03g50290	14-3-3 protein, putative, expressed	(3.02)	(*)		down
LOC_Os03g55150	eukaryotic translation initiation factor 5A, putative, expressed	(*)			down
LOC_Os03g59310	ribosomal protein, putative, expressed	(2.48)		(12.58)	down
LOC_Os03g61640	ZOS3-23 - C2H2 zinc finger protein, expressed		(*)		down
LOC_Os03g63670	expressed protein		(*)		down
LOC_Os04g01740	heat shock protein, putative, expressed	(*)			down
LOC_Os04g02820	elongation factor, putative, expressed	(2.81)	(*)	(*)	down
LOC_Os04g28180	protein ribosomal protein, putative, expressed			(2.75)	down
LOC_Os04g34100	protein expressed protein			(*)	down
LOC_Os04g38310	expressed protein		(*)		down
LOC_Os04g38870	14-3-3 protein, putative, expressed	(3.85)			down
LOC_Os04g42270	protein 60S ribosomal protein L23A, putative, expressed			(2.68)	down
LOC_Os04g51630	protein 60S ribosomal protein L7, putative, expressed			(5.04)	down
LOC_Os04g57010	zinc finger C-x8-C-x5-C-x3-H type family protein, expressed		(*)		down
LOC_Os05g01450	eukaryotic translation initiation factor 3 subunit F, putative, expressed	(*)			down
LOC_Os05g06350	importin subunit alpha, putative, expressed	(3.04)			down
LOC_Os05g06770	40S ribosomal protein S27a, putative, expressed		(*)		down
LOC_Os05g07700	ribosomal protein, putative, expressed		(16.28)	(*)	down
LOC_Os05g16660	WD domain, G-beta repeat domain containing protein, expressed	(*)			down
LOC_Os05g27940	40S ribosomal protein S7, putative, expressed	(3.01)	(1.9)	(12.69)	down
LOC_Os05g28280	protein peptidase, M24 family protein, putative, expressed			(3.6)	down
LOC_Os05g30530	protein 40S ribosomal protein S4, putative, expressed			(4.15)	down
LOC_Os05g40820	ribosomal protein L24, putative, expressed		(*)		down
LOC_Os05g49890	ras-related protein, putative, expressed	(2.56)			down
LOC_Os05g51180	hyaluronan/mRNA binding family domain containing protein, expressed		(*)		down
LOC_Os06g01700	CWC15 homolog A, putative, expressed		(*)		down
LOC_Os06g07580	expressed protein		(*)		down
LOC_Os06g10430	protein of unknown function DUF1296 domain containing protein, expressed		(*)		down
LOC_Os06g40560	26S protease regulatory subunit S10B, putative, expressed	(*)			down
LOC_Os06g41384	zinc finger C-x8-C-x5-C-x3-H type family protein, expressed		(*)		down
LOC_Os06g45710	phosphoglycerate kinase protein, putative, expressed	(2.5)	(*)		down
LOC_Os06g48750	DEAD-box ATP-dependent RNA helicase, putative, expressed	(2.37)	(*)		down
LOC_Os07g01490	kinesin motor domain containing protein, putative, expressed	(*)			down

Supplement Table 3 cont. Differentially Regulated Nuclear Proteins					
**Locus ID**	**Annotation**	**Method(Fold-Change)**			**Regulation**
		**A**	**B**	**C**	
LOC_Os07g10660	protein ribosomal protein, putative, expressed			(8.03)	down
LOC_Os11g38900	Histone-lysine N-methyltransferase, H3 lysine-9 specific SUVH1, putative,	(7.60)			down
LOC_Os07g41260	protein PPR repeat domain containing protein, putative, expressed			(*)	down
LOC_Os07g41750	protein 40S ribosomal protein S3-1, putative, expressed			(*)	down
LOC_Os07g42170	60S ribosomal protein, putative, expressed		(3.4)	(7.17)	down
LOC_Os07g42450	protein ribosomal protein S2, putative, expressed			(*)	down
LOC_Os07g42950	protein 40S ribosomal protein S6, putative,			(6.96)	down
LOC_Os07g49150	26S protease regulatory subunit 4, putative, expressed	(2.87)			down
LOC_Os08g03520	retrotransposon protein, putative, Ty1-copia subclass, expressed		(2.72)		down
LOC_Os08g03640	protein 60S acidic ribosomal protein P0, putative, expressed			(*)	down
LOC_Os08g13690	protein 60S ribosomal protein L7, putative, expressed			(4.78)	down
LOC_Os08g21660	WD domain, G-beta repeat domain containing protein, expressed	(*)			down
LOC_Os08g23710	protein ribosomal protein L7Ae, putative, expressed			(3.6)	down
LOC_Os08g33370	14-3-3 protein, putative, expressed	(3.58)	(*)		down
LOC_Os08g37490	14-3-3 protein, putative, expressed	(3.16)			down
LOC_Os08g39140	heat shock protein, putative, expressed	(2.66)	(*)		down
LOC_Os08g44480	40S ribosomal protein S25, putative, expressed		(*)	(*)	down
LOC_Os09g30418	heat shock protein, putative, expressed	(2.8)	(*)		down
LOC_Os09g31180	protein ribosomal protein L6, putative, expressed			(4.61)	down
LOC_Os09g32500	protein 60S ribosomal protein L32, putative, expressed			(*)	down
LOC_Os09g39540	40S ribosomal protein S25, putative, expressed		(*)	(*)	down
LOC_Os10g27190	40S ribosomal protein S17, putative, expressed		(*)	(*)	down
LOC_Os10g30580	cell division control protein 48 homolog E, putative, expressed	(7.29)			down
LOC_Os10g41470	protein 60S ribosomal protein L27-3, putative, expressed			(*)	down
LOC_Os11g05562	40S ribosomal protein S25, putative, expressed		(*)		down
LOC_Os11g06750	protein ribosomal protein L3, putative, expressed			(2.25)	down
LOC_Os11g11390	ribosomal protein, putative, expressed		(9.68)	(*)	down
LOC_Os11g34450	14-3-3 protein, putative, expressed	(9.21)			down
LOC_Os12g07010	protein ribosomal protein L3, putative, expressed			(2.04)	down
LOC_Os12g21798	protein 40S ribosomal protein S3a, putative, expressed			(7)	down
LOC_Os12g38000	60S ribosomal protein L8, putative, expressed		(4.35)	(2.67)	down
LOC_Os03g54890	protein ribosomal protein L13, putative,			(29.09)	down
LOC_Os01g14950	importin subunit alpha, putative, expressed	(2.6)			down
LOC_Os03g10340	protein 40S ribosomal protein S3a, putative, expressed			(6)	down
LOC_Os07g05580	ribosomal protein L7Ae, putative, expressed	(*)	(2.77)	(*)	down
LOC_Os05g41172	histone-lysine N-methyltransferase, H3 lysine-9 specific SUVH1, putative,	(*)	(*)		down

a) **Locus ID**: TIGR Locus ID
b) **Annotation**: Protein Name
c) **Method**: Protein extraction method in which the protein differentially expression was examined. The three columns indicate the following extraction procedures: (A) Phenol extraction; (B) Phenol-Acid double extraction; (C) Acid extraction.
d) **Fold-Change**: Relative expression level compared with suspension cell nuclear protein. Xcorr generated by ProtQuant was used for quantification analysis. * indicates that the fold change is not available because the protein is not detected either in the control or in the treatment.
e) **Regulation**: type of differential expression. Down: down regulation compared with suspension cell nuclear protein. Up: up regulation compared with suspension cell nuclear protein.

A

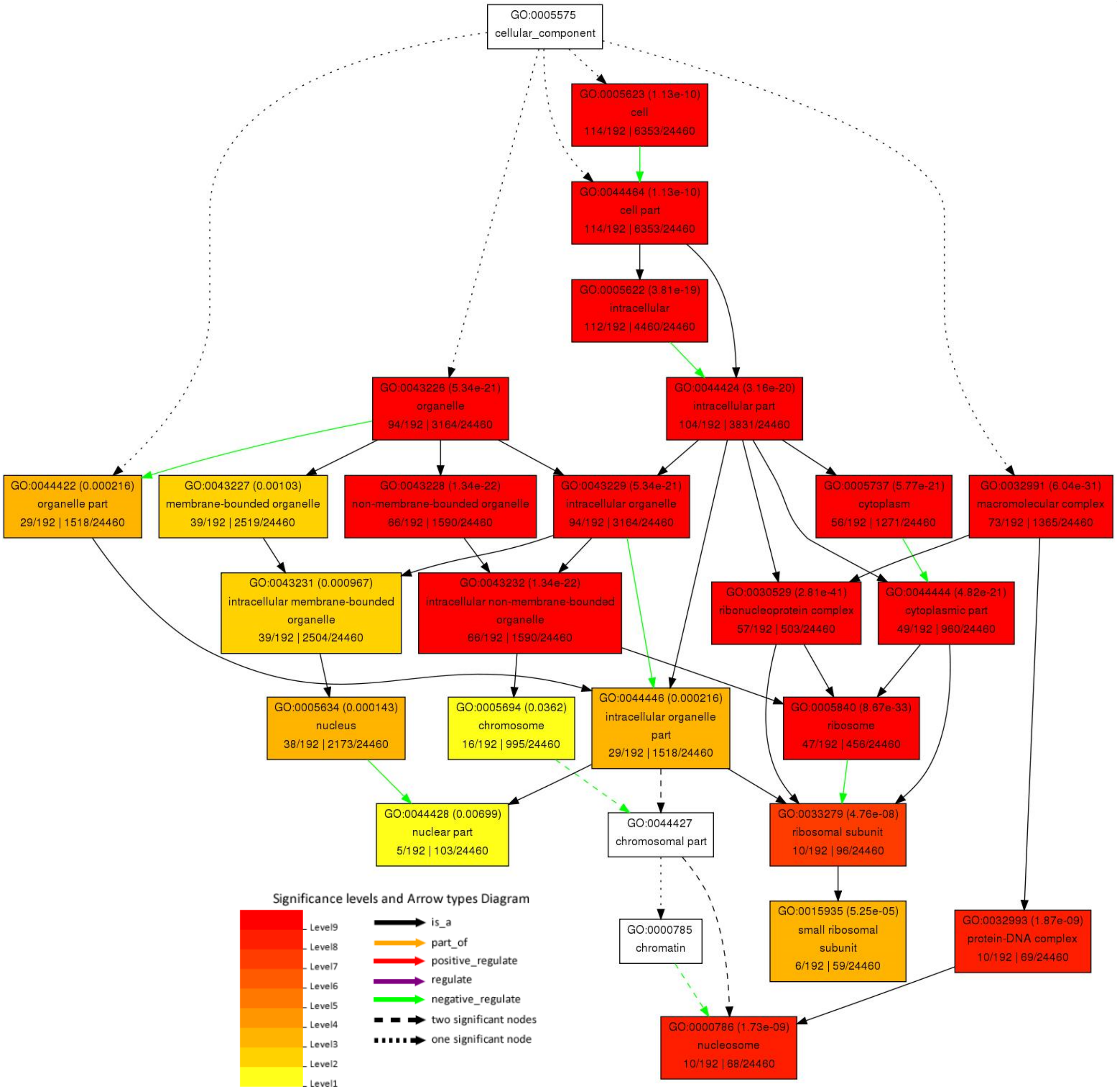
GO:0005575
cellular_component
GO:0005623 (1.13e-10)
cell
114/192 | 6353/24460
GO:0044464 (1.13e-10)
cell part
114/192 | 6353/24460
GO:0005622 (3.81e-19)
intracellular
112/192 | 4460/24460
GO:0043226 (5.34e-21)
organelle
94/192 | 3164/24460
GO:0044424 (3.16e-20)
intracellular part
104/192 | 3831/24460
GO:0044422 (0.000216)
organelle part
29/192 | 1518/24460
GO:0043227 (0.00103)
membrane-bounded organelle
39/192 | 2519/24460
GO:0043228 (1.34e-22)
non-membrane-bounded organelle
66/192 | 1590/24460
GO:0043229 (5.34e-21)
intracellular organelle
94/192 | 3164/24460
GO:0005737 (5.77e-21)
cytoplasm
56/192 | 1271/24460
GO:0032991 (6.04e-31)
macromolecular complex
73/192 | 1365/24460
GO:0043231 (0.000967)
intracellular membrane-bounded organelle
39/192 | 2504/24460
GO:0043232 (1.34e-22)
intracellular non-membrane-bounded organelle
66/192 | 1590/24460
GO:0030529 (2.81e-41)
ribonucleoprotein complex
57/192 | 503/24460
GO:0044444 (4.82e-21)
cytoplasmic part
49/192 | 960/24460
GO:0005634 (0.000143)
nucleus
38/192 | 2173/24460
GO:0005694 (0.0362)
chromosome
16/192 | 995/24460
GO:0044446 (0.000216)
intracellular organelle part
29/192 | 1518/24460
GO:0005840 (8.67e-33)
ribosome
47/192 | 456/24460
GO:0044428 (0.00699)
nuclear part
5/192 | 103/24460
GO:0044427
chromosomal part
GO:0033279 (4.76e-08)
ribosomal subunit
10/192 | 96/24460
GO:0000785
chromatin
GO:0015935 (5.25e-05)
small ribosomal subunit
6/192 | 59/24460
GO:0032993 (1.87e-09)
protein-DNA complex
10/192 | 69/24460
GO:0000786 (1.73e-09)
nucleosome
10/192 | 68/24460
Significance levels and Arrow types Diagram
Level9
Level8
Level7
Level6
Level5
Level4
Level3
Level2
Level1
is_a
part_of
positive_regulate
regulate
negative_regulate
two significant nodes
one significant node

B

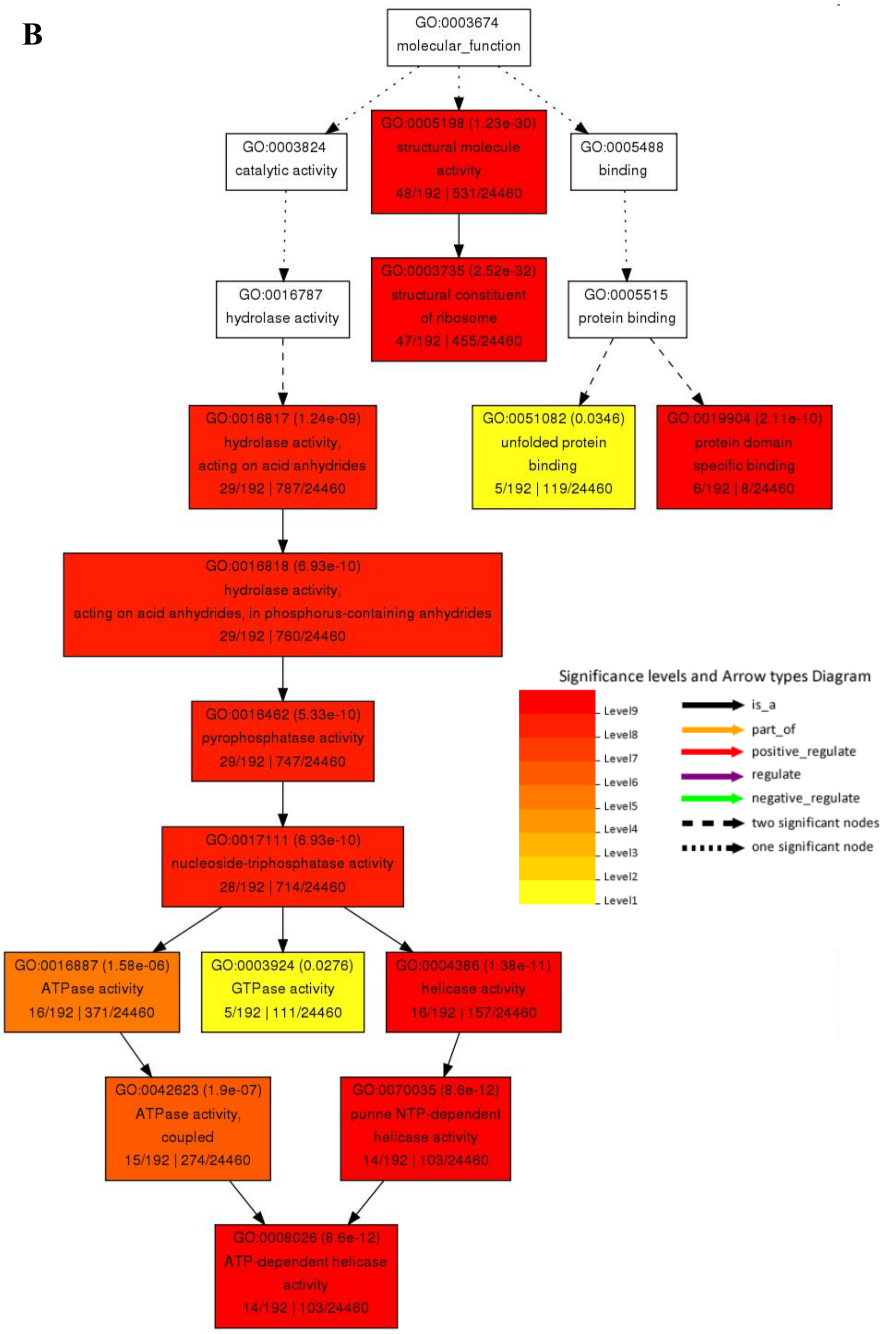

Supplement Figure 1. Enriched cellular component and molecular function of differentially expressed nuclear proteins revealed by GO analysis. (A) Cellular Component. (B) Molecular Function. Figure displays the significantly enriched biological process and cellular component GO annotation for differentially expressed nuclear proteins. The top line in each box is the GO identifier of the term and statistical significance (multiple hypothesis corrected p-value, lower is more significant) of that annotation. The middle line in each box is a description of the GO term. The four numbers on the bottom line are the number of nuclear proteins with this annotation, the number of nuclear proteins that had any annotation (192), the total number of proteins that had the annotation, and the total number of proteins that had any annotation (24460). The color of the box indicates the significance of the term as indicated by the legend on the bottom left corner. White boxes are not significant.
