# Supplementary material for: Nuclear proteome response to cell wall removal in rice (Oryza sativa)
Source: Proteome Sci. 2013 Jun 19;11:26. doi: 10.1186/1477-5956-11-26 (PMC3695858; doi:10.1186/1477-5956-11-26)
Supplement: Additional file 2 — As Orthologous Proteins from Different Plant Species. [file 1477-5956-11-26-S2.pdf]

Additional File Two: Orthologous Proteins from Different Plant Species

Species	Orthologous genes	Putative function
Rice	LOC_Os12g44390	RecF/RecN/SMC N terminal domain containing protein, expressed
Arabidopsis	AT3G54670	Structural maintenance of chromosomes (SMC) family protein
Poplar	POPTR_0005s24640	TTN8 (TITAN8); ATP binding / transporter
Grapevine	GSVIVG00028343001	Structural maintenance of chromosome 1 protein
Sorghum	Sb07g023430	Condensin complex components subunit
Maize	GRMZM2G416069	Structural maintenance of chromosome 1 protein
	GRMZM5G899800	RecF/RecN/SMC N terminal domain containing protein
Brachypodium	Bradi3g12830	Structural maintenance of chromosome 1 protein

Species	Orthologous genes	Putative function
Rice	LOC_Os12g42150	WD domain, G-beta repeat domain containing protein, expressed
Arabidopsis	AT1G65030	Transducin/WD40 repeat-like superfamily protein
Poplar	POPTR_0012s10930	transducin family protein / WD-40 repeat family protein
Grapevine	GSVIVG00034157001	Pak inhibitor skb15
Sorghum	Sb08g021660	Nucleotide binding protein
Maize	GRMZM2G327655	Nucleotide binding protein
Brachypodium	Bradi4g01590	Transducin family protein

Species	Orthologous genes	Putative function
Rice	LOC_Os12g41930	SRP40, C-terminal domain containing protein, expressed
Arabidopsis	AT5G57120	NA
Poplar	POPTR_0006s07280	unknown protein
	POPTR_0018s13690	unknown protein
Grapevine	GSVIVG00029286001	Cylicin-2
Sorghum	Sb08g021440	BRI1-KD interacting protein 132
Maize	GRMZM2G164868	BRI1-KD interacting protein 132
Brachypodium	Bradi4g01760	SRP40, C-terminal domain containing protein

Species	Orthologous genes	Putative function
Rice	LOC_Os12g41620	WD domain, G-beta repeat domain containing protein, expressed
Arabidopsis	AT2G18900	Transducin/WD40 repeat-like superfamily protein
Poplar	POPTR_0006s18020	transducin family protein / WD-40 repeat family protein
	POPTR_0018s09840	transducin family protein / WD-40 repeat family protein
Grapevine	GSVIVG00030738001	Wd40 protein
Sorghum	Sb08g020980	Wd40 protein
Maize	GRMZM2G049201	Wd40 protein
Brachypodium	Bradi4g02100	Wd40 protein

Species	Orthologous genes	Putative function
Rice	LOC_Os12g38000	60S ribosomal protein L8, putative, expressed
Arabidopsis	AT2G18020	Ribosomal protein L2 family
	AT4G36130	Ribosomal protein L2 family

Poplar	POPTR_0005s11780	60S ribosomal protein L8 (RPL8C)
	POPTR_0789s00200	60S ribosomal protein L8 (RPL8C)
Grapevine	GSVIVG00014552001	60S ribosomal protein L8
Sorghum	Sb02g009810	60S ribosomal protein L2
	Sb08g018650	60S ribosomal protein L2
Maize	GRMZM2G067456	60S ribosomal protein L2
	GRMZM2G072729	60S ribosomal protein L2
	GRMZM2G077851	60S ribosomal protein L2
Brachypodium	Bradi4g04120	60S ribosomal protein L2

**Species**	**Orthologous genes**	**Putative function**
Rice	LOC_Os03g51200	Core histone H2A/H2B/H3/H4 domain containing protein, putative, expressed
	LOC_Os12g34510	Core histone H2A/H2B/H3/H4 domain containing protein, putative, expressed
Arabidopsis	AT1G08880	Histone superfamily protein
	AT1G54690	gamma histone variant H2AX
Poplar	POPTR_0005s04260	GAMMA-H2AX (GAMMA HISTONE VARIANT H2AX); DNA binding
	POPTR_0013s02990	GAMMA-H2AX (GAMMA HISTONE VARIANT H2AX); DNA binding
	POPTR_0369s00210	GAMMA-H2AX (GAMMA HISTONE VARIANT H2AX); DNA binding
Grapevine	GSVIVG00011033001	Histone H2A
Sorghum	Sb01g009820	Histone H2A
	Sb01g028960	Histone H2A
	Sb08g016830	Histone H2A
Maize	GRMZM2G046055	Histone H2A
Brachypodium	Bradi1g10390	Histone H2A
	Bradi4g06010	histone H2AXb

**Species**	**Orthologous genes**	**Putative function**
Rice	LOC_Os03g31210	UDP-glucose 6-dehydrogenase, putative, expressed
	LOC_Os03g55070	UDP-glucose 6-dehydrogenase, putative, expressed
	LOC_Os12g25690	UDP-glucose 6-dehydrogenase, putative, expressed
	LOC_Os12g25700	UDP-glucose 6-dehydrogenase, putative, expressed
Arabidopsis	AT3G29360	UDP-glucose 6-dehydrogenase family protein
	AT5G15490	UDP-glucose 6-dehydrogenase family protein
	AT5G39320	UDP-glucose 6-dehydrogenase family protein
Poplar	POPTR_0004s11760	UDP-glucose 6-dehydrogenase, putative
	POPTR_0008s09390	UDP-glucose 6-dehydrogenase, putative
	POPTR_0010s16730	UDP-glucose 6-dehydrogenase, putative
	POPTR_0017s12760	UDP-glucose 6-dehydrogenase, putative
Grapevine	GSVIVG00007910001	UDP-glucose dehydrogenase
	GSVIVG00012198001	UDP-glucose 6-dehydrogenase
Sorghum	Sb01g007580	UDP-glucose 6-dehydrogenase
Maize	GRMZM2G328500	UDP-glucose 6-dehydrogenase

Additional File 2 cont.: Orthologous Proteins from Different Plant Species

	GRMZM5G862540	UDP-glucose 6-dehydrogenase
Brachypodium	Bradi1g08120	UDP-glucose 6-dehydrogenase
	Bradi4g25140	UDP-glucose 6-dehydrogenase

**Species**	**Orthologous genes**	**Putative function**
Rice	LOC_Os07g36130	core histone H2A/H2B/H3/H4, putative, expressed
	LOC_Os12g25120	core histone H2A/H2B/H3/H4, putative, expressed
Sorghum	Sb02g035640	Histone H2A
Maize	GRMZM2G041381	Histone H2A
	GRMZM2G151826	histone H2A.1
Brachypodium	Bradi1g25400	histone H2A.7

**Species**	**Orthologous genes**	**Putative function**
Rice	LOC_Os02g18550	40S ribosomal protein S3a, putative, expressed
	LOC_Os03g10340	40S ribosomal protein S3a, putative, expressed
	LOC_Os12g21798	40S ribosomal protein S3a, putative, expressed
Arabidopsis	AT3G04840	Ribosomal protein S3Ae
	AT4G34670	Ribosomal protein S3Ae
Poplar	POPTR_0005s05280	40S ribosomal protein S3A (RPS3aB)
	POPTR_0008s15610	40S ribosomal protein S3A (RPS3aB)
	POPTR_0010s09330	40S ribosomal protein S3A (RPS3aB)
Grapevine	GSVIVG00018024001	40S ribosomal protein S3a-1
Sorghum	Sb02g038365	40S ribosomal protein S3a
	Sb02g038370	40S ribosomal protein S3a
Maize	GRMZM2G030915	40S ribosomal protein S3a
	GRMZM2G145258	40S ribosomal protein S3a
Brachypodium	Bradi1g71200	40S ribosomal protein S3a
	Bradi1g78170	40S ribosomal protein S3a
	Bradi3g01210	40S ribosomal protein S3a

**Species**	**Orthologous genes**	**Putative function**
Rice	LOC_Os05g23740	DnaK family protein, putative, expressed
	LOC_Os12g14070	DnaK family protein, putative, expressed
Arabidopsis	AT4G24280	chloroplast heat shock protein 70-1
	AT5G49910	chloroplast heat shock protein 70-2
Poplar	POPTR_0004s23310	CPHSC70-2EAT SHOCK PROTEIN 70-2 (CHLOROPLAST HEAT SHOCK PROTEIN 70-2); ATP binding / unfolded protein binding
	POPTR_0022s00530	CPHSC70-2EAT SHOCK PROTEIN 70-2 (CHLOROPLAST HEAT SHOCK PROTEIN 70-2); ATP binding / unfolded protein binding
Grapevine	GSVIVG00026014001	Heat shock protein
Sorghum	Sb08g009580	70 kDa heat shock protein
Maize	GRMZM2G001500	70 kDa heat shock protein
	GRMZM2G079668	Chloroplast heat shock protein 70
	GRMZM2G111475	Chloroplast heat shock protein 70

Additional File 2 cont.: Orthologous Proteins from Different Plant Species

Brachypodium	Bradi2g30560	Chloroplast heat shock protein 70
	Bradi4g39470	70 kDa heat shock protein

**Species**	**Orthologous genes**	**Putative function**
Rice	LOC_Os11g06750	ribosomal protein L3, putative, expressed
	LOC_Os12g07010	ribosomal protein L3, putative, expressed
Arabidopsis	AT1G43170	ribosomal protein 1
	AT1G61580	R-protein L3 B
Poplar	POPTR_0002s06680	ARP1 (ARABIDOPSIS RIBOSOMAL PROTEIN 1); structural constituent of ribosome
	POPTR_0005s21640	ARP1 (ARABIDOPSIS RIBOSOMAL PROTEIN 1); structural constituent of ribosome
	POPTR_0022s00670	RPL3B (R-PROTEIN L3 B); structural constituent of ribosome
Grapevine	GSVIVG00023241001	60S ribosomal protein L3
	GSVIVG00034555001	60S ribosomal protein L3
Sorghum	Sb05g004130	60S ribosomal protein L3
	Sb06g028650	60S ribosomal protein L3
Maize	GRMZM2G132968	60S ribosomal protein L3
	GRMZM2G135727	60S ribosomal protein L3
	GRMZM2G324314	60S ribosomal protein L3
	GRMZM5G801409	60S ribosomal protein L3
Brachypodium	Bradi4g24610	Ribosomal protein L3

**Species**	**Orthologous genes**	**Putative function**
Rice	LOC_Os12g06910	nucleolar protein family 6, putative, expressed
Arabidopsis	AT1G63810	NA
Poplar	POPTR_0007s12920	unknown protein
Grapevine	GSVIVG00018952001	Nucleolar RNA-associated protein
Sorghum	Sb08g004180	Nrap protein
Maize	GRMZM2G036019	Nrap protein
Brachypodium	Bradi4g41720	Nrap protein

**Species**	**Orthologous genes**	**Putative function**
Rice	LOC_Os08g03640	60S acidic ribosomal protein P0, putative, expressed
	LOC_Os11g04070	60S acidic ribosomal protein P0, putative, expressed
	LOC_Os12g03880	60S acidic ribosomal protein P0, putative, expressed
Arabidopsis	AT2G40010	Ribosomal protein L10 family protein
	AT3G09200	Ribosomal protein L10 family protein
	AT3G11250	Ribosomal protein L10 family protein
Poplar	POPTR_0008s06620	60S acidic ribosomal protein P0 (RPP0A)
	POPTR_0010s19860	60S acidic ribosomal protein P0 (RPP0A)
Grapevine	GSVIVG00016313001	60S acidic ribosomal protein P0
	GSVIVG00032857001	60S acidic ribosomal protein P0
Sorghum	Sb07g002560	60S acidic ribosomal protein P0

Additional File 2 cont.: Orthologous Proteins from Different Plant Species

	Sb09g028230	60S acidic ribosomal protein P0
Maize	GRMZM2G066460	60S acidic ribosomal protein P0
	GRMZM2G179976	60S acidic ribosomal protein P0
Brachypodium	Bradi3g14340	60S acidic ribosomal protein P0

**Species**	**Orthologous genes**	**Putative function**
Rice	LOC_Os11g01420	ribosomal protein L10, putative, expressed
	LOC_Os12g01430	ribosomal protein L10, putative, expressed
Arabidopsis	AT1G25260	Ribosomal protein L10 family protein
Poplar	POPTR_0001s46440	acidic ribosomal protein P0-related
	POPTR_0011s15980	acidic ribosomal protein P0-related
Grapevine	GSVIVG00023018001	Ribosomal protein L10
Sorghum	Sb05g000460	mRNA turnover protein 4
	Sb08g000490	mRNA turnover protein 4
Maize	GRMZM2G155437	mRNA turnover protein 4
	GRMZM2G467086	mRNA turnover protein 4
Brachypodium	Bradi4g44960	mRNA turnover protein 4

**Species**	**Orthologous genes**	**Putative function**
Rice	LOC_Os11g43900	translationally-controlled tumor protein, putative, expressed
Arabidopsis	AT3G05540	Methionine sulfoxide reductase (MSS4-like) family protein
	AT3G16640	translationally controlled tumor protein
Poplar	POPTR_0005s02470	TCTP (TRANSLATIONALLY CONTROLLED TUMOR PROTEIN)
	POPTR_0008s22310	TCTP (TRANSLATIONALLY CONTROLLED TUMOR PROTEIN)
	POPTR_0010s01700	TCTP (TRANSLATIONALLY CONTROLLED TUMOR PROTEIN)
Grapevine	GSVIVG00017723001	Translationally-controlled tumor protein
	GSVIVG00031135001	Translationally-controlled tumor protein
Sorghum	Sb04g000750	Translationally controlled tumor protein
Maize	GRMZM2G075624	Translationally-controlled tumor protein
	GRMZM2G108474	Translationally-controlled tumor protein
Brachypodium	Bradi4g10920	Translationally-controlled tumor protein homolog

**Species**	**Orthologous genes**	**Putative function**
Rice	LOC_Os11g43890	WD domain, G-beta repeat domain containing protein, expressed
Arabidopsis	AT4G29830	Transducin/WD40 repeat-like superfamily protein
Poplar	POPTR_0006s14800	VIP3 (vernalization independence 3); nucleotide binding / protein binding
Grapevine	GSVIVG00015149001	Meiotic recombination protein
Sorghum	Sb05g026120	Meiotic recombination protein
Maize	GRMZM2G126552	Meiotic recombination protein
Brachypodium	Bradi4g10930	Meiotic recombination protein

**Species**	**Orthologous genes**	**Putative function**

Additional File 2 cont.: Orthologous Proteins from Different Plant Species

Rice	LOC_Os11g40090	A49-like RNA polymerase I associated factor family protein, expressed
Arabidopsis	AT3G13940	DNA binding;DNA-directed RNA polymerases
Poplar	POPTR_0001s20580	DNA binding / DNA-directed RNA polymerase
Grapevine	GSVIVG00016817001	DNA-directed RNA polymerase I 49 kDa polypeptide
Sorghum	Sb05g024500	A49-like RNA polymerase I associated factor family protein
Maize	GRMZM2G453684	A49-like RNA polymerase I associated factor family protein
Brachypodium	Bradi4g13720	A49-like RNA polymerase I associated factor family protein

**Species**	**Orthologous genes**	**Putative function**
Rice	LOC_Os03g05980	40S ribosomal protein S9-2, putative, expressed
	LOC_Os11g38959	40S ribosomal protein S9-2, putative, expressed
Arabidopsis	AT5G39850	Ribosomal protein S4
Poplar	POPTR_0006s22630	40S ribosomal protein S9 (RPS9C)
	POPTR_0007s09750	40S ribosomal protein S9 (RPS9C)
	POPTR_0011s09670	40S ribosomal protein S9 (RPS9C)
	POPTR_0016s07730	40S ribosomal protein S9 (RPS9C)
	POPTR_0016s07770	40S ribosomal protein S9 (RPS9C)
	POPTR_0018s07520	40S ribosomal protein S9 (RPS9C)
Grapevine	GSVIVG00008028001	40S ribosomal protein S9
	GSVIVG00011384001	40S ribosomal protein S9
	GSVIVG00020020001	40S ribosomal protein S9
Sorghum	Sb02g040080	40S ribosomal protein S9
	Sb02g040120	40S ribosomal protein S9
	Sb08g015270	40S ribosomal protein S9
Maize	GRMZM2G108348	40S ribosomal protein S9
	GRMZM5G832108	40S ribosomal protein S9
Brachypodium	Bradi1g20580	40S ribosomal protein S9
	Bradi1g61090	40S ribosomal protein S9
	Bradi4g07120	40S ribosomal protein S9
	Bradi4g14160	40S ribosomal protein S9

**Species**	**Orthologous genes**	**Putative function**
Rice	LOC_Os11g38900	histone-lysine N-methyltransferase, H3 lysine-9 specific SUVH1, putative, expressed
Arabidopsis	AT5G04940	SU(VAR)3-9 homolog 1
Poplar	POPTR_0001s07390	SUVH3 (SU(VAR)3-9 HOMOLOG 3); histone methyltransferase
	POPTR_0003s18740	SUVH3 (SU(VAR)3-9 HOMOLOG 3); histone methyltransferase
Sorghum	Sb02g006620	SET1
	Sb06g001340	SET1
Maize	AC233961.1_FG001	SET1
Brachypodium	Bradi1g53840	SET1

**Species**	**Orthologous genes**	**Putative function**

Additional File 2 cont.: Orthologous Proteins from Different Plant Species

Rice	LOC_Os11g37080	h/ACA ribonucleoprotein complex subunit 1-like protein 1, putative, expressed
Arabidopsis	AT3G03920	"H/ACA ribonucleoprotein complex, subunit Gar1/Naf1 protein"
Poplar	POPTR_0013s05490	Gar1 RNA-binding region family protein
	POPTR_0019s05040	Gar1 RNA-binding region family protein
Grapevine	GSVIVG00033401001	H/ACA ribonucleoprotein complex subunit
Sorghum	Sb05g022540	H/ACA ribonucleoprotein complex subunit 1 1
Maize	GRMZM2G032419	H/ACA ribonucleoprotein complex subunit 1 1
Brachypodium	Bradi4g15350	H/ACA ribonucleoprotein complex subunit 1 1

**Species**	**Orthologous genes**	**Putative function**
Rice	LOC_Os11g36390	RFC1 - Putative clamp loader of PCNA, replication factor C subunit 1, expressed
Arabidopsis	AT5G22010	replication factor C1
Poplar	POPTR_0001s22140	AtRFC1 (replication factor C 1); ATP binding / DNA binding / DNA clamp loader/ nucleoside-triphosphatase/ nucleotide binding
Grapevine	GSVIVG00037357001	Replication factor C large subunit
Sorghum	Sb02g030910	Replication factor C 110 kDa subunit
	Sb05g022200	Replication factor C 110 kDa subunit
Maize	GRMZM2G457381	Replication factor C 110 kDa subunit
Brachypodium	Bradi4g16040	Replication factor C 110 kDa subunit

**Species**	**Orthologous genes**	**Putative function**
Rice	LOC_Os11g34450	14-3-3 protein, putative, expressed
Sorghum	Sb05g021020	14-3-3
Maize	GRMZM2G408768	14-3-3
Brachypodium	Bradi4g16640	14-3-3E

**Species**	**Orthologous genes**	**Putative function**
Rice	LOC_Os05g07700	ribosomal protein, putative, expressed
	LOC_Os11g11390	ribosomal protein, putative, expressed
Arabidopsis	AT1G14320	Ribosomal protein L16p/L10e family protein
	AT1G26910	Ribosomal protein L16p/L10e family protein
	AT1G66580	senescence associated gene 24
Poplar	POPTR_0013s15560	60S ribosomal protein L10 (RPL10B)
	POPTR_0019s15250	60S ribosomal protein L10 (RPL10B)
Grapevine	GSVIVG00024537001	60S ribosomal protein L10
Sorghum	Sb01g015470	60S ribosomal protein L10-3
	Sb04g008500	60S ribosomal protein L10-1
Maize	GRMZM2G087233	60S ribosomal protein L10-3
Brachypodium	Bradi4g22070	60S ribosomal protein L10-3

**Species**	**Orthologous genes**	**Putative function**
Rice	LOC_Os02g55010	expressed protein
	LOC_Os11g07470	expressed protein

Additional File 2 cont.: Orthologous Proteins from Different Plant Species

Arabidopsis	AT3G01780	ARM repeat superfamily protein
Poplar	POPTR_0001s34190	TPLATE; binding
Grapevine	GSVIVG00001825001	Armadillo-like helical domain-containing protein
Sorghum	Sb05g004840	Armadillo-like helical domain-containing protein
Maize	GRMZM2G162286	Conserved gene of unknown function
	GRMZM2G472770	Conserved gene of unknown function
Brachypodium	Bradi4g24200	Armadillo-like helical domain-containing protein

Species	Orthologous genes	Putative function
Rice	LOC_Os11g06750	ribosomal protein L3, putative, expressed
	LOC_Os12g07010	ribosomal protein L3, putative, expressed
Arabidopsis	AT1G43170	ribosomal protein 1
	AT1G61580	R-protein L3 B
Poplar	POPTR_0002s06680	ARP1 (ARABIDOPSIS RIBOSOMAL PROTEIN 1); structural constituent of ribosome
	POPTR_0005s21640	ARP1 (ARABIDOPSIS RIBOSOMAL PROTEIN 1); structural constituent of ribosome
	POPTR_0022s00670	RPL3B (R-PROTEIN L3 B); structural constituent of ribosome
Grapevine	GSVIVG00023241001	60S ribosomal protein L3
	GSVIVG00034555001	60S ribosomal protein L3
Sorghum	Sb05g004130	60S ribosomal protein L3
	Sb06g028650	60S ribosomal protein L3
Maize	GRMZM2G132968	60S ribosomal protein L3
	GRMZM2G135727	60S ribosomal protein L3
	GRMZM2G324314	60S ribosomal protein L3
	GRMZM5G801409	60S ribosomal protein L3
Brachypodium	Bradi4g24610	Ribosomal protein L3

Species	Orthologous genes	Putative function
Rice	LOC_Os08g03640	60S acidic ribosomal protein P0, putative, expressed
	LOC_Os11g04070	60S acidic ribosomal protein P0, putative, expressed
	LOC_Os12g03880	60S acidic ribosomal protein P0, putative, expressed
Arabidopsis	AT2G40010	Ribosomal protein L10 family protein
	AT3G09200	Ribosomal protein L10 family protein
	AT3G11250	Ribosomal protein L10 family protein
Poplar	POPTR_0008s06620	60S acidic ribosomal protein P0 (RPP0A)
	POPTR_0010s19860	60S acidic ribosomal protein P0 (RPP0A)
Grapevine	GSVIVG00016313001	60S acidic ribosomal protein P0
	GSVIVG00032857001	60S acidic ribosomal protein P0
Sorghum	Sb07g002560	60S acidic ribosomal protein P0
	Sb09g028230	60S acidic ribosomal protein P0
Maize	GRMZM2G066460	60S acidic ribosomal protein P0
	GRMZM2G179976	60S acidic ribosomal protein P0
Brachypodium	Bradi3g14340	60S acidic ribosomal protein P0

Additional File 2 cont.: Orthologous Proteins from Different Plant Species

Species	Orthologous genes	Putative function
Rice	LOC_Os11g01420	ribosomal protein L10, putative, expressed
	LOC_Os12g01430	ribosomal protein L10, putative, expressed
Arabidopsis	AT1G25260	Ribosomal protein L10 family protein
Poplar	POPTR_0001s46440	acidic ribosomal protein P0-related
	POPTR_0011s15980	acidic ribosomal protein P0-related
Grapevine	GSVIVG00023018001	Ribosomal protein L10
Sorghum	Sb05g000460	mRNA turnover protein 4
	Sb08g000490	mRNA turnover protein 4
Maize	GRMZM2G155437	mRNA turnover protein 4
	GRMZM2G467086	mRNA turnover protein 4
Brachypodium	Bradi4g44960	mRNA turnover protein 4

Species	Orthologous genes	Putative function
Rice	LOC_Os02g18380	60S ribosomal protein L27-3, putative, expressed
	LOC_Os10g41470	60S ribosomal protein L27-3, putative, expressed
Arabidopsis	AT3G22230	Ribosomal L27e protein family
	AT4G15000	Ribosomal L27e protein family
Poplar	POPTR_0001s35630	60S ribosomal protein L27 (RPL27C)
	POPTR_0006s02230	60S ribosomal protein L27 (RPL27C)
	POPTR_0016s02040	60S ribosomal protein L27 (RPL27C)
Grapevine	GSVIVG00012080001	60S ribosomal protein L27
	GSVIVG00020410001	60S ribosomal protein L27
Sorghum	Sb06g029070	60S ribosomal protein L27
	Sb10g027330	60S ribosomal protein L27
Maize	GRMZM2G016250	60S ribosomal protein L27
	GRMZM2G302712	60S ribosomal protein L27
	GRMZM2G326066	60S ribosomal protein L27
	GRMZM2G366077	60S ribosomal protein L27
Brachypodium	Bradi1g30210	60S ribosomal protein L27

Species	Orthologous genes	Putative function
Rice	LOC_Os10g35290	DNA-directed RNA polymerase I subunit RPA2, putative, expressed
Arabidopsis	AT1G29940	nuclear RNA polymerase A2
Poplar	POPTR_0001s06810	NRPA2; DNA binding / DNA-directed RNA polymerase/ ribonucleoside binding
Grapevine	GSVIVG00036642001	DNA-directed RNA polymerase
Sorghum	Sb04g001790	DNA-directed RNA polymerase
Maize	GRMZM2G388892	DNA-directed RNA polymerase
Brachypodium	Bradi3g29920	DNA-directed RNA polymerase

Species	Orthologous genes	Putative function

Additional File 2 cont.: Orthologous Proteins from Different Plant Species

Rice	LOC_Os10g35280	nucleolar complex protein 2, putative, expressed
Arabidopsis	AT2G18220	Noc2p family
Poplar	POPTR_0001s05030	unknown protein
	POPTR_0003s21740	unknown protein
Grapevine	GSVIVG00037396001	Peroxidase 31
Sorghum	Sb01g018260	Nucleolar complex protein 2 homolog
Maize	GRMZM2G374385	Nucleolar complex protein 2 homolog
Brachypodium	Bradi3g29890	Nucleolar complex protein 2 homolog

**Species**	**Orthologous genes**	**Putative function**
Rice	LOC_Os10g32880	WD domain, G-beta repeat domain containing protein, expressed
Arabidopsis	AT1G04510	MOS4-associated complex 3A
	AT2G33340	MOS4-associated complex 3B
Poplar	POPTR_0008s17140	nucleotide binding / ubiquitin-protein ligase
	POPTR_0010s07660	nucleotide binding / ubiquitin-protein ligase
Grapevine	GSVIVG00027911001	Pre-mRNA-splicing factor
Sorghum	Sb01g019790	Pre-mRNA-splicing factor 19
Maize	GRMZM2G037698	Pre-mRNA-splicing factor 19
	GRMZM2G324540	Pre-mRNA-splicing factor 19
Brachypodium	Bradi1g75750	Pre-mRNA-splicing factor 19
	Bradi3g28280	Pre-mRNA-splicing factor 19

**Species**	**Orthologous genes**	**Putative function**
Rice	LOC_Os01g51620	KRR1 small subunit processome component, putative, expressed
	LOC_Os10g31520	ribosomal RNA assembly protein mis3, putative, expressed
Arabidopsis	AT5G08420	RNA-binding KH domain-containing protein
Poplar	POPTR_0007s08410	RNA binding
Grapevine	GSVIVG00037891001	Ribosomal RNA assembly protein mis3
Sorghum	Sb03g032750	Rev interacting protein mis3
Maize	GRMZM2G167809	Ribosomal RNA assembly protein mis3
Brachypodium	Bradi2g48070	Ribosomal RNA assembly protein mis3

**Species**	**Orthologous genes**	**Putative function**
Rice	LOC_Os03g05730	cell division control protein 48 homolog E, putative, expressed
	LOC_Os10g30580	cell division control protein 48 homolog E, putative, expressed
Arabidopsis	AT3G09840	cell division cycle 48
	AT3G53230	"ATPase, AAA-type, CDC48 protein"
	AT5G03340	"ATPase, AAA-type, CDC48 protein"
Poplar	POPTR_0006s12740	cell division cycle protein 48, putative / CDC48, putative
	POPTR_0012s09000	cell division cycle protein 48, putative / CDC48, putative
	POPTR_0015s09220	cell division cycle protein 48, putative / CDC48, putative
	POPTR_0016s09280	cell division cycle protein 48, putative / CDC48, putative

Additional File 2 cont.: Orthologous Proteins from Different Plant Species

	POPTR_0017s14340	cell division cycle protein 48, putative / CDC48, putative
Grapevine	GSVIVG00007689001	Cell division cycle protein 48 homolog
	GSVIVG00025723001	Transitional endoplasmic reticulum ATPase
	GSVIVG00031517001	similar to Cell division cycle protein 48 homolog
Sorghum	Sb01g020910	Transitional endoplasmic reticulum ATPase
	Sb01g046840	Transitional endoplasmic reticulum ATPase
	Sb01g047410	Cell division cycle protein 48 homolog
	Sb01g047440	Cell division cycle protein 48 homolog
Maize	AC233949.1_FG004	Cell division cycle protein 48
	GRMZM2G036765	Cell division cycle protein 48
	GRMZM2G063060	Cell division cycle protein 48
Brachypodium	Bradi1g74920	Transitional endoplasmic reticulum ATPase
	Bradi1g75570	Cell division cycle protein 48 homolog

**Species**	**Orthologous genes**	**Putative function**
Rice	LOC_Os03g06670	Core histone H2A/H2B/H3/H4 domain containing protein, putative, expressed
	LOC_Os03g53190	Core histone H2A/H2B/H3/H4 domain containing protein, putative, expressed
	LOC_Os10g28230	Core histone H2A/H2B/H3/H4 domain containing protein, putative, expressed
Arabidopsis	AT1G52740	histone H2A protein 9
	AT3G54560	histone H2A 11
Poplar	POPTR_0002s04720	HTA11; DNA binding
	POPTR_0005s23810	HTA11; DNA binding
	POPTR_0006s26540	HTA9 (HISTONE H2A PROTEIN 9); DNA binding
	POPTR_0006s26550	HTA9 (HISTONE H2A PROTEIN 9); DNA binding
	POPTR_0018s01310	HTA9 (HISTONE H2A PROTEIN 9); DNA binding
	POPTR_0018s01320	HTA9 (HISTONE H2A PROTEIN 9); DNA binding
Grapevine	GSVIVG00003139001	Histone H2A
	GSVIVG00017218001	Histone H2A
	GSVIVG00035768001	Histone H2A
Sorghum	Sb04g025140	Histone H2A
Maize	GRMZM2G050833	Histone H2A
	GRMZM2G056231	Histone H2A
	GRMZM2G149775	Histone H2A
Brachypodium	Bradi1g09060	histone H2A variant 3
	Bradi3g26880	histone H2A variant 2

**Species**	**Orthologous genes**	**Putative function**
Rice	LOC_Os03g01900	40S ribosomal protein S17, putative, expressed
	LOC_Os10g27190	40S ribosomal protein S17, putative, expressed
Arabidopsis	AT2G04390	Ribosomal S17 family protein
	AT2G05220	Ribosomal S17 family protein
	AT3G10610	Ribosomal S17 family protein

Additional File 2 cont.: Orthologous Proteins from Different Plant Species

	AT5G04800	Ribosomal S17 family protein
Poplar	POPTR_0008s01820	40S ribosomal protein S17 (RPS17D)
	POPTR_0010s24760	40S ribosomal protein S17 (RPS17D)
Grapevine	GSVIVG00033920001	40S ribosomal protein S17
	GSVIVG00034653001	40S ribosomal protein S17
Sorghum	Sb10g006620	40S ribosomal protein S17-4
Maize	GRMZM2G073150	40S ribosomal protein S17-4
	GRMZM2G086906	40S ribosomal protein S17-4
Brachypodium	Bradi3g29060	40S ribosomal protein S17-4

**Species**	**Orthologous genes**	**Putative function**
Rice	LOC_Os08g44480	40S ribosomal protein S25, putative, expressed
	LOC_Os09g39540	40S ribosomal protein S25, putative, expressed
Arabidopsis	AT2G21580	Ribosomal protein S25 family protein
	AT4G34555	Ribosomal protein S25 family protein
	AT4G39200	Ribosomal protein S25 family protein
Poplar	POPTR_0004s16450	40S ribosomal protein S25 (RPS25E)
	POPTR_0009s12150	40S ribosomal protein S25 (RPS25E)
	POPTR_0010s24580	40S ribosomal protein S25 (RPS25E)
Grapevine	GSVIVG00000974001	40S ribosomal protein S25
	GSVIVG00010721001	40S ribosomal protein S25
	GSVIVG00023806001	40S ribosomal protein S25-1
Sorghum	Sb03g036900	40S ribosomal protein S25-1
	Sb03g036910	40S ribosomal protein S25-1
	Sb03g038310	40S ribosomal protein S25-1
Maize	GRMZM2G057608	40S ribosomal protein S25-1
	GRMZM2G139349	40S ribosomal protein S25-1
	GRMZM5G867518	40S ribosomal protein S25-1
Brachypodium	Bradi2g00800	40S ribosomal protein S25-1
	Bradi3g42900	40S ribosomal protein S25-1
	Bradi4g38580	40S ribosomal protein S25-1

**Species**	**Orthologous genes**	**Putative function**
Rice	LOC_Os02g49270	NOL1/NOP2/sun family protein, putative, expressed
	LOC_Os09g37860	NOL1/NOP2/sun family protein, putative, expressed
Arabidopsis	AT4G26600	S-adenosyl-L-methionine-dependent methyltransferases superfamily protein
	AT5G55920	S-adenosyl-L-methionine-dependent methyltransferases superfamily protein
Poplar	POPTR_0001s37830	nucleolar protein, putative
Grapevine	GSVIVG00014717001	Proliferating-cell nucleolar antigen p120
Sorghum	Sb04g029465	proliferating cell nuclear protein P120
Maize	GRMZM2G005256	proliferating cell nuclear protein P120
Brachypodium	Bradi3g56470	proliferating cell nuclear protein P120

Additional File 2 cont.: Orthologous Proteins from Different Plant Species

Species	Orthologous genes	Putative function
Rice	LOC_Os06g49830	SHI, putative, expressed
	LOC_Os09g36160	LRP1, putative, expressed
Arabidopsis	AT3G51060	Lateral root primordium (LRP) protein-related
Poplar	POPTR_0002s03000	SRS5 (SHI-RELATED SEQUENCE 5)
	POPTR_0005s12020	STY1 (STYLISH 1); protein heterodimerization/ transcription factor
	POPTR_0005s25560	SRS5 (SHI-RELATED SEQUENCE 5)
	POPTR_0007s13610	STY1 (STYLISH 1); protein heterodimerization/ transcription factor
Sorghum	Sb10g029800	Stylish
Maize	GRMZM2G080295	LRP1
	GRMZM2G097683	LRP1
	GRMZM2G108798	LRP1
Brachypodium	Bradi1g34990	Transcription factor
	Bradi4g36030	lateral root primordia (LRP1)

Species	Orthologous genes	Putative function
Rice	LOC_Os08g41300	60S ribosomal protein L32, putative, expressed
	LOC_Os09g32500	60S ribosomal protein L32, putative, expressed
	LOC_Os09g32520	60S ribosomal protein L32, putative, expressed
	LOC_Os09g32532	60S ribosomal protein L32, putative, expressed
Arabidopsis	AT4G18100	Ribosomal protein L32e
	AT5G46430	Ribosomal protein L32e
Poplar	POPTR_0001s34370	60S ribosomal protein L32 (RPL32A)
	POPTR_0002s25050	60S ribosomal protein L32 (RPL32A)
	POPTR_0011s02900	60S ribosomal protein L32 (RPL32A)
	POPTR_0014s18940	60S ribosomal protein L32 (RPL32A)
Grapevine	GSVIVG00021377001	60S ribosomal protein L32
Sorghum	Sb02g029030	60S ribosomal protein L32
Maize	GRMZM2G115901	60S ribosomal protein L32
Brachypodium	Bradi3g40840	60S ribosomal protein L32
	Bradi3g40870	60S ribosomal protein L32
	Bradi4g34340	60S ribosomal protein L32
	Bradi4g34360	60S ribosomal protein L32

Species	Orthologous genes	Putative function
Rice	LOC_Os08g41300	60S ribosomal protein L32, putative, expressed
	LOC_Os09g32500	60S ribosomal protein L32, putative, expressed
	LOC_Os09g32520	60S ribosomal protein L32, putative, expressed
	LOC_Os09g32532	60S ribosomal protein L32, putative, expressed
Arabidopsis	AT4G18100	Ribosomal protein L32e
	AT5G46430	Ribosomal protein L32e
Poplar	POPTR_0001s34370	60S ribosomal protein L32 (RPL32A)

Additional File 2 cont.: Orthologous Proteins from Different Plant Species

	POPTR_0002s25050	60S ribosomal protein L32 (RPL32A)
	POPTR_0011s02900	60S ribosomal protein L32 (RPL32A)
	POPTR_0014s18940	60S ribosomal protein L32 (RPL32A)
Grapevine	GSVIVG00021377001	60S ribosomal protein L32
Sorghum	Sb02g029030	60S ribosomal protein L32
Maize	GRMZM2G115901	60S ribosomal protein L32
Brachypodium	Bradi3g40840	60S ribosomal protein L32
	Bradi3g40870	60S ribosomal protein L32
	Bradi4g34340	60S ribosomal protein L32
	Bradi4g34360	60S ribosomal protein L32

Species	Orthologous genes	Putative function
Rice	LOC_Os08g41300	60S ribosomal protein L32, putative, expressed
	LOC_Os09g32500	60S ribosomal protein L32, putative, expressed
	LOC_Os09g32520	60S ribosomal protein L32, putative, expressed
	LOC_Os09g32532	60S ribosomal protein L32, putative, expressed
Arabidopsis	AT4G18100	Ribosomal protein L32e
	AT5G46430	Ribosomal protein L32e
Poplar	POPTR_0001s34370	60S ribosomal protein L32 (RPL32A)
	POPTR_0002s25050	60S ribosomal protein L32 (RPL32A)
	POPTR_0011s02900	60S ribosomal protein L32 (RPL32A)
	POPTR_0014s18940	60S ribosomal protein L32 (RPL32A)
Grapevine	GSVIVG00021377001	60S ribosomal protein L32
Sorghum	Sb02g029030	60S ribosomal protein L32
Maize	GRMZM2G115901	60S ribosomal protein L32
Brachypodium	Bradi3g40840	60S ribosomal protein L32
	Bradi3g40870	60S ribosomal protein L32
	Bradi4g34340	60S ribosomal protein L32
	Bradi4g34360	60S ribosomal protein L32

Species	Orthologous genes	Putative function
Rice	LOC_Os02g01332	ribosomal protein L6, putative, expressed
	LOC_Os09g31180	ribosomal protein L6, putative, expressed
Arabidopsis	AT1G33120	Ribosomal protein L6 family
	AT1G33140	Ribosomal protein L6 family
	AT4G10450	Ribosomal protein L6 family
Poplar	POPTR_0001s45810	PGY2 (PIGGYBACK2); structural constituent of ribosome
	POPTR_0001s45820	PGY2 (PIGGYBACK2); structural constituent of ribosome
	POPTR_0011s15080	PGY2 (PIGGYBACK2); structural constituent of ribosome
	POPTR_0011s15170	PGY2 (PIGGYBACK2); structural constituent of ribosome
Grapevine	GSVIVG00016795001	60S ribosomal protein L9
	GSVIVG00038670001	60S ribosomal protein L9

Additional File 2 cont.: Orthologous Proteins from Different Plant Species

Sorghum	Sb10g000700	60S ribosomal protein L9
Maize	GRMZM2G084739	60S ribosomal protein L9
	GRMZM2G385287	60S ribosomal protein L9
Brachypodium	Bradi1g21850	60S ribosomal protein L9
	Bradi1g52040	60S ribosomal protein L9
	Bradi3g00640	60S ribosomal protein L9

**Species**	**Orthologous genes**	**Putative function**
Rice	LOC_Os08g39140	heat shock protein, putative, expressed
	LOC_Os09g30412	heat shock protein, putative, expressed
	LOC_Os09g30418	heat shock protein, putative
	LOC_Os09g30439	heat shock protein, putative
Arabidopsis	AT5G56000	HEAT SHOCK PROTEIN 81.4
	AT5G56010	heat shock protein 81-3
Poplar	POPTR_0001s29350	heat shock protein 81-4 (HSP81-4)
	POPTR_0001s47020	heat shock protein 81-4 (HSP81-4)
	POPTR_0006s00470	heat shock protein 81-4 (HSP81-4)
	POPTR_0016s00510	heat shock protein 81-4 (HSP81-4)
Grapevine	GSVIVG00003469001	Heat shock protein 90-1
	GSVIVG00028856001	Heat shock protein
Sorghum	Sb02g028020	Heat shock protein 81-2
	Sb02g028050	Heat shock protein 81-2
	Sb07g028270	Heat shock protein 90
Maize	GRMZM2G012631	HSP protein
	GRMZM2G069651	Heat shock protein 81-1
	GRMZM2G112165	Heat shock protein 81-1
Brachypodium	Bradi3g39590	Cytosolic heat shock protein 90.2
	Bradi3g39620	Cytosolic heat shock protein 90.2
	Bradi3g39630	Cytosolic heat shock protein 90.2

**Species**	**Orthologous genes**	**Putative function**
Rice	LOC_Os08g39140	heat shock protein, putative, expressed
	LOC_Os09g30412	heat shock protein, putative, expressed
	LOC_Os09g30418	heat shock protein, putative
	LOC_Os09g30439	heat shock protein, putative
Arabidopsis	AT5G56000	HEAT SHOCK PROTEIN 81.4
	AT5G56010	heat shock protein 81-3
Poplar	POPTR_0001s29350	heat shock protein 81-4 (HSP81-4)
	POPTR_0001s47020	heat shock protein 81-4 (HSP81-4)
	POPTR_0006s00470	heat shock protein 81-4 (HSP81-4)
	POPTR_0016s00510	heat shock protein 81-4 (HSP81-4)
Grapevine	GSVIVG00003469001	Heat shock protein 90-1
	GSVIVG0002885600	Heat shock protein

	1	
Sorghum	Sb02g028020<br>Sb02g028050<br>Sb07g028270	Heat shock protein 81-2<br>Heat shock protein 81-2<br>Heat shock protein 90
Maize	GRMZM2G012631<br>GRMZM2G069651<br>GRMZM2G112165	HSP protein<br>Heat shock protein 81-1<br>Heat shock protein 81-1
Brachypodium	Bradi3g39590<br>Bradi3g39620<br>Bradi3g39630	Cytosolic heat shock protein 90.2<br>Cytosolic heat shock protein 90.2<br>Cytosolic heat shock protein 90.2

**Species**	**Orthologous genes**	**Putative function**
Rice	LOC_Os09g27850	transcription regulator, putative, expressed
Sorghum	Sb02g026310	storekeeper protein
Maize	GRMZM2G134866	storekeeper protein
Brachypodium	Bradi4g31840	storekeeper protein

**Species**	**Orthologous genes**	**Putative function**
Rice	LOC_Os09g24820	ZF-HD protein dimerisation region containing protein, expressed
Arabidopsis	AT3G28920<br>AT5G39760	homeobox protein 34<br>homeobox protein 23
Poplar	POPTR_0004s12500<br>POPTR_0017s11920	ATHB30 (ARABIDOPSIS THALIANA HOMEOBOX PROTEIN 30); DNA binding / transcription factor<br>AtHB34 (ARABIDOPSIS THALIANA HOMEOBOX PROTEIN 34); DNA binding / transcription factor
Grapevine	GSVIVG0001141300<br>1	Zinc finger-homeodomain protein 1
Sorghum	Sb02g024650	ZF-HD homeobox protein
Maize	GRMZM2G051955<br>GRMZM2G161315	ZF-HD homeobox protein<br>ZF-HD homeobox protein
Brachypodium	Bradi4g30240	ZF-HD homeobox protein

**Species**	**Orthologous genes**	**Putative function**
Rice	LOC_Os09g24260	WD domain, G-beta repeat domain containing protein, expressed
Arabidopsis	AT3G50590	Transducin/WD40 repeat-like superfamily protein
Poplar	POPTR_0005s17990<br>POPTR_0007s11290	nucleotide binding<br>nucleotide binding
Grapevine	GSVIVG0000657500<br>1	Nucleotide binding protein
Sorghum	Sb01g014880	Nucleotide binding protein
Maize	GRMZM2G150772	nucleotide binding
Brachypodium	Bradi1g51890	nucleotide binding

**Species**	**Orthologous genes**	**Putative function**
Rice	LOC_Os06g48350<br>LOC_Os06g48355<br>LOC_Os09g15770	CPuORF14 - conserved peptide uORF-containing transcript, expressed<br>expressed protein<br>CPuORF13 - conserved peptide uORF-containing transcript, expressed

	LOC_Os09g15775	expressed protein
Arabidopsis	AT1G36730	Translation initiation factor IF2/IF5
	AT1G77840	Translation initiation factor IF2/IF5
Poplar	POPTR_0004s09250	eukaryotic translation initiation factor 5, putative / eIF-5, putative
	POPTR_0004s11110	eukaryotic translation initiation factor 5, putative / eIF-5, putative
	POPTR_0005s14880	eukaryotic translation initiation factor 5, putative / eIF-5, putative
Grapevine	GSVIVG00008949001	Eukaryotic translation initiation factor
Sorghum	Sb01g038080	Eukaryotic translation initiation factor 5
	Sb04g003550	Eukaryotic translation initiation factor 5
Maize	GRMZM2G369939	Eukaryotic translation initiation factor 5
Brachypodium	Bradi3g03430	Translation initiation factor eIF5
	Bradi3g03850	Translation initiation factor eIF5

Species	Orthologous genes	Putative function
Rice	LOC_Os06g48350	CPuORF14 - conserved peptide uORF-containing transcript, expressed
	LOC_Os06g48355	expressed protein
	LOC_Os09g15770	CPuORF13 - conserved peptide uORF-containing transcript, expressed
	LOC_Os09g15775	expressed protein
Arabidopsis	AT1G36730	Translation initiation factor IF2/IF5
	AT1G77840	Translation initiation factor IF2/IF5
Poplar	POPTR_0004s09250	eukaryotic translation initiation factor 5, putative / eIF-5, putative
	POPTR_0004s11110	eukaryotic translation initiation factor 5, putative / eIF-5, putative
	POPTR_0005s14880	eukaryotic translation initiation factor 5, putative / eIF-5, putative
Grapevine	GSVIVG00008949001	Eukaryotic translation initiation factor
Sorghum	Sb01g038080	Eukaryotic translation initiation factor 5
	Sb04g003550	Eukaryotic translation initiation factor 5
Maize	GRMZM2G369939	Eukaryotic translation initiation factor 5
Brachypodium	Bradi3g03430	Translation initiation factor eIF5
	Bradi3g03850	Translation initiation factor eIF5

Species	Orthologous genes	Putative function
Rice	LOC_Os09g10770	OsTOP6B - Topoisomerase 6 subunit B, expressed
Arabidopsis	AT3G20780	topoisomerase 6 subunit B
Poplar	POPTR_0003s22040	ATTOP6B (topoisomerase 6 subunit B); DNA topoisomerase (ATP-hydrolyzing)/ identical protein binding / protein binding
Grapevine	GSVIVG00025336001	Type II DNA topoisomerase VI subunit B
Sorghum	Sb02g018660	topoisomerase VI subunit B
Maize	GRMZM2G015011	Topoisomerase 6 subunit B
Brachypodium	Bradi4g27060	topoisomerase VI subunit B

Species	Orthologous genes	Putative function
Rice	LOC_Os09g08430	ribosomal protein L22, putative, expressed
Arabidopsis	AT1G27400	Ribosomal protein L22p/L17e family protein

Additional File 2 cont.: Orthologous Proteins from Different Plant Species

	AT1G67430	Ribosomal protein L22p/L17e family protein
Poplar	POPTR_0008s17560	60S ribosomal protein L17 (RPL17B)
	POPTR_0010s07020	60S ribosomal protein L17 (RPL17B)
	POPTR_0012s09860	60S ribosomal protein L17 (RPL17A)
	POPTR_0015s10650	60S ribosomal protein L17 (RPL17A)
Grapevine	GSVIVG00008528001	Ribosomal protein
	GSVIVG00010449001	Ribosomal protein
Sorghum	Sb01g001600	60S ribosomal protein L17
	Sb01g001610	60S ribosomal protein L17
	Sb09g006420	60S ribosomal protein L17
Maize	GRMZM2G126594	60S ribosomal protein L17
	GRMZM2G148744	60S ribosomal protein L17
	GRMZM2G702426	60S ribosomal protein L17
Brachypodium	Bradi3g41150	60S ribosomal protein L17-2
	Bradi4g08670	Ribosomal protein L17-1

Species	Orthologous genes	Putative function
Rice	LOC_Os09g02810	CCAAT/enhancer-binding protein, putative, expressed
Arabidopsis	AT1G72440	CCAAT-binding factor
Poplar	POPTR_0001s16440	EDA25 (embryo sac development arrest 25)
Grapevine	GSVIVG00016696001	Slow walker 2
Sorghum	Sb01g042620	Slow walker 2
Maize	GRMZM2G407287	CCAAT-box-binding transcription factor
Brachypodium	Bradi3g47770	EDA25 (embryo sac development arrest 25)

Species	Orthologous genes	Putative function
Rice	LOC_Os09g02284	DNA-directed RNA polymerase subunit, putative, expressed
Arabidopsis	AT2G15400	DNA-directed RNA polymerase family protein
	AT2G15430	DNA-directed RNA polymerase family protein
Poplar	POPTR_0009s10050	NRPB3; DNA binding / DNA-directed RNA polymerase/ protein dimerization
Grapevine	GSVIVG00003174001	DNA-directed RNA polymerase II subunit
Sorghum	Sb02g014460	DNA-directed RNA polymerase II 36 kDa polypeptide A
Maize	GRMZM5G874167	DNA-directed RNA polymerase II 36 kDa polypeptide A
Brachypodium	Bradi4g08350	DNA-directed RNA polymerase II 36 kDa polypeptide A

Species	Orthologous genes	Putative function
Rice	LOC_Os08g44480	40S ribosomal protein S25, putative, expressed
	LOC_Os09g39540	40S ribosomal protein S25, putative, expressed
Arabidopsis	AT2G21580	Ribosomal protein S25 family protein
	AT4G34555	Ribosomal protein S25 family protein
	AT4G39200	Ribosomal protein S25 family protein
Poplar	POPTR_0004s16450	40S ribosomal protein S25 (RPS25E)

Additional File 2 cont.: Orthologous Proteins from Different Plant Species

	POPTR_0009s12150	40S ribosomal protein S25 (RPS25E)
	POPTR_0010s24580	40S ribosomal protein S25 (RPS25E)
Grapevine	GSVIVG00000974001	40S ribosomal protein S25
	GSVIVG00010721001	40S ribosomal protein S25
	GSVIVG00023806001	40S ribosomal protein S25-1
Sorghum	Sb03g036900	40S ribosomal protein S25-1
	Sb03g036910	40S ribosomal protein S25-1
	Sb03g038310	40S ribosomal protein S25-1
Maize	GRMZM2G057608	40S ribosomal protein S25-1
	GRMZM2G139349	40S ribosomal protein S25-1
	GRMZM5G867518	40S ribosomal protein S25-1
Brachypodium	Bradi2g00800	40S ribosomal protein S25-1
	Bradi3g42900	40S ribosomal protein S25-1
	Bradi4g38580	40S ribosomal protein S25-1

**Species**	**Orthologous genes**	**Putative function**
Rice	LOC_Os01g64090	L1P family of ribosomal proteins domain containing protein, expressed
	LOC_Os02g21660	L1P family of ribosomal proteins domain containing protein, expressed
	LOC_Os08g44380	L1P family of ribosomal proteins domain containing protein, expressed
Arabidopsis	AT1G08360	Ribosomal protein L1p/L10e family
	AT2G27530	Ribosomal protein L1p/L10e family
	AT5G22440	Ribosomal protein L1p/L10e family
Poplar	POPTR_0004s21290	60S ribosomal protein L10A (RPL10aA)
	POPTR_0004s21300	PGY1 (PIGGYBACK1); RNA binding / structural constituent of ribosome
	POPTR_0007s11880	PGY1 (PIGGYBACK1); RNA binding / structural constituent of ribosome
	POPTR_0007s11960	PGY1 (PIGGYBACK1); RNA binding / structural constituent of ribosome
Grapevine	GSVIVG00033841001	Ribosomal protein L1
	GSVIVG00036128001	similar to ribosomal protein L10a
Sorghum	Sb03g040550	Ribosomal protein L1
	Sb07g024200	Ribosomal protein L1
	Sb07g024210	Ribosomal protein L1
Maize	GRMZM2G023748	Ribosomal protein
	GRMZM2G144387	Ribosomal protein
Brachypodium	Bradi4g38510	Ribosomal protein L1

**Species**	**Orthologous genes**	**Putative function**
Rice	LOC_Os08g42920	60S ribosomal protein L7, putative, expressed
Arabidopsis	AT1G80750	Ribosomal protein L30/L7 family protein
Poplar	POPTR_0001s14790	60S ribosomal protein L7 (RPL7A)
	POPTR_0003s17910	60S ribosomal protein L7 (RPL7A)
Grapevine	GSVIVG00034230001	60S ribosomal protein L7
Sorghum	Sb07g025450	60S ribosomal protein L7

Additional File 2 cont.: Orthologous Proteins from Different Plant Species

Maize	GRMZM2G107336	60S ribosomal protein L7
Brachypodium	Bradi3g41850	60S ribosomal protein L7

Species	Orthologous genes	Putative function
Rice	LOC_Os08g41300	60S ribosomal protein L32, putative, expressed
	LOC_Os09g32500	60S ribosomal protein L32, putative, expressed
	LOC_Os09g32520	60S ribosomal protein L32, putative, expressed
	LOC_Os09g32532	60S ribosomal protein L32, putative, expressed
Arabidopsis	AT4G18100	Ribosomal protein L32e
	AT5G46430	Ribosomal protein L32e
Poplar	POPTR_0001s34370	60S ribosomal protein L32 (RPL32A)
	POPTR_0002s25050	60S ribosomal protein L32 (RPL32A)
	POPTR_0011s02900	60S ribosomal protein L32 (RPL32A)
	POPTR_0014s18940	60S ribosomal protein L32 (RPL32A)
Grapevine	GSVIVG00021377001	60S ribosomal protein L32
Sorghum	Sb02g029030	60S ribosomal protein L32
Maize	GRMZM2G115901	60S ribosomal protein L32
Brachypodium	Bradi3g40840	60S ribosomal protein L32
	Bradi3g40870	60S ribosomal protein L32
	Bradi4g34340	60S ribosomal protein L32
	Bradi4g34360	60S ribosomal protein L32

Species	Orthologous genes	Putative function
Rice	LOC_Os08g39140	heat shock protein, putative, expressed
	LOC_Os09g30412	heat shock protein, putative, expressed
	LOC_Os09g30418	heat shock protein, putative
	LOC_Os09g30439	heat shock protein, putative
Arabidopsis	AT5G56000	HEAT SHOCK PROTEIN 81.4
	AT5G56010	heat shock protein 81-3
Poplar	POPTR_0001s29350	heat shock protein 81-4 (HSP81-4)
	POPTR_0001s47020	heat shock protein 81-4 (HSP81-4)
	POPTR_0006s00470	heat shock protein 81-4 (HSP81-4)
	POPTR_0016s00510	heat shock protein 81-4 (HSP81-4)
Grapevine	GSVIVG00003469001	Heat shock protein 90-1
	GSVIVG00028856001	Heat shock protein
Sorghum	Sb02g028020	Heat shock protein 81-2
	Sb02g028050	Heat shock protein 81-2
	Sb07g028270	Heat shock protein 90
Maize	GRMZM2G012631	HSP protein
	GRMZM2G069651	Heat shock protein 81-1
	GRMZM2G112165	Heat shock protein 81-1
Brachypodium	Bradi3g39590	Cytosolic heat shock protein 90.2
	Bradi3g39620	Cytosolic heat shock protein 90.2

	Bradi3g39630	Cytosolic heat shock protein 90.2

Species	Orthologous genes	Putative function
Rice	LOC_Os08g23120	RNA recognition motif containing protein, putative, expressed
	LOC_Os08g38410	RNA recognition motif containing protein, putative, expressed
Arabidopsis	AT3G13224	RNA-binding (RRM/RBD/RNP motifs) family protein
Poplar	POPTR_0001s37920	RNA recognition motif (RRM)-containing protein
Grapevine	GSVIVG00014729001	Heterogeneous nuclear ribonucleoprotein A1
Sorghum	Sb03g027920	Heterogeneous nuclear ribonucleoprotein A1
Maize	GRMZM2G050218	Heterogeneous nuclear ribonucleoprotein A1
	GRMZM2G152526	Heterogeneous nuclear ribonucleoprotein A1
Brachypodium	Bradi3g20700	Heterogeneous nuclear ribonucleoprotein A1
	Bradi3g39090	Heterogeneous nuclear ribonucleoprotein A1

Species	Orthologous genes	Putative function
Rice	LOC_Os01g05610	Core histone H2A/H2B/H3/H4 domain containing protein, putative, expressed
	LOC_Os01g05630	Core histone H2A/H2B/H3/H4 domain containing protein, putative, expressed
	LOC_Os01g05900	Core histone H2A/H2B/H3/H4 domain containing protein, putative
	LOC_Os01g05970	OsFBO1 - F-box and other domain containing protein, expressed
	LOC_Os01g06010	Core histone H2A/H2B/H3/H4 domain containing protein, putative, expressed
	LOC_Os01g62230	Core histone H2A/H2B/H3/H4 domain containing protein, putative, expressed
	LOC_Os05g49860	Core histone H2A/H2B/H3/H4 domain containing protein, putative, expressed
	LOC_Os08g38300	Core histone H2A/H2B/H3/H4 domain containing protein, putative, expressed
Arabidopsis	AT1G07790	Histone superfamily protein
	AT2G28720	Histone superfamily protein
	AT3G45980	Histone superfamily protein
	AT3G46030	Histone superfamily protein
	AT5G22880	histone B2
	AT5G59910	Histone superfamily protein
Poplar	POPTR_0008s02990	HTB1; DNA binding
	POPTR_0008s03040	histone H2B, putative
	POPTR_0008s03050	histone H2B, putative
	POPTR_0008s03060	HTB9; DNA binding
	POPTR_0010s23720	histone H2B, putative
	POPTR_0010s23730	histone H2B, putative
	POPTR_0010s23770	HTB9; DNA binding
Grapevine	GSVIVG00025021001	Histone H2B
	GSVIVG00025023001	Histone H2B
	GSVIVG00025025001	Histone H2B
Sorghum	Sb02g025410	Histone H2B
	Sb02g041800	Histone H2B
	Sb03g005720	Histone H2B
	Sb03g005730	Histone H2B

Additional File 2 cont.: Orthologous Proteins from Different Plant Species

	Sb03g007700	Histone H2B
	Sb03g026260	Histone H2B
	Sb03g039310	Histone H2B
	Sb04g030340	Histone H2B
	Sb07g022370	Histone H2B
	Sb07g028760	Histone H2B
	Sb09g022610	Histone H2B
Maize	GRMZM2G071959	Histone H2B.1
	GRMZM2G112912	Histone H2B
	GRMZM2G119071	Histone H2B.2
	GRMZM2G141432	Histone H2B
	GRMZM2G163939	Histone H2B
	GRMZM2G304575	Histone H2B
	GRMZM2G306258	Histone H2B.4
	GRMZM2G342515	Histone H2B.5
	GRMZM2G401147	Histone H2B
	GRMZM2G472696	Histone H2B
Brachypodium	Bradi1g08860	Histone H2B.1
	Bradi1g47980	Histone H2B
	Bradi1g56060	Histone H2B.1
	Bradi2g00510	Histone H2B
	Bradi2g00530	Histone H2B
	Bradi2g23230	Histone H2B
	Bradi2g27710	Histone H2B.1
	Bradi2g27760	Histone H2B
	Bradi2g54540	Histone H2B.11
	Bradi3g54520	Histone H2B

**Species**	**Orthologous genes**	**Putative function**
Rice	LOC_Os08g37490	14-3-3 protein, putative, expressed
Sorghum	Sb07g029110	14-3-3 A
Maize	GRMZM2G140545	14-3-3 A
Brachypodium	Bradi3g38640	14-3-3D

**Species**	**Orthologous genes**	**Putative function**
Rice	LOC_Os08g36450	transcription regulator, putative, expressed
Sorghum	Sb07g022740	storekeeper protein
Maize	GRMZM2G423292	storekeeper protein
Brachypodium	Bradi3g37930	storekeeper protein

**Species**	**Orthologous genes**	**Putative function**
Rice	LOC_Os08g06040	60S ribosomal protein L34, putative, expressed
	LOC_Os08g33920	60S ribosomal protein L34, putative, expressed
Sorghum	Sb07g003770	60S ribosomal protein L34

Additional File 2 cont.: Orthologous Proteins from Different Plant Species

Maize	GRMZM2G032564	60S ribosomal protein L34

Species	Orthologous genes	Putative function
Rice	LOC_Os04g38870	14-3-3 protein, putative, expressed
	LOC_Os08g33370	14-3-3 protein, putative, expressed
Arabidopsis	AT3G02520	general regulatory factor 7
	AT5G16050	general regulatory factor 5
Poplar	POPTR_0004s10120	GRF7 (GENERAL REGULATORY FACTOR 7); protein binding / protein phosphorylated amino acid binding
	POPTR_0017s13840	GRF7 (GENERAL REGULATORY FACTOR 7); protein binding / protein phosphorylated amino acid binding
Grapevine	GSVIVG00014210001	14-3-3 protein
Sorghum	Sb06g019100	14-3-3 GF14-6
	Sb07g020990	14-3-3
	Sb07g025680	14-3-3
Maize	AC217050.4_FG006	14-3-3
	GRMZM2G091155	14-3-3
	GRMZM2G102499	14-3-3 GF14-6
Brachypodium	Bradi3g36480	14-3-3 GF14-C
	Bradi5g12510	14-3-3 GF14-B

Species	Orthologous genes	Putative function
Rice	LOC_Os08g33120	RNA recognition motif containing protein, putative, expressed
Arabidopsis	AT1G66260	RNA-binding (RRM/RBD/RNP motifs) family protein
	AT5G37720	ALWAYS EARLY 4
Poplar	POPTR_0004s08540	RNA and export factor-binding protein, putative
	POPTR_0017s02890	RNA and export factor-binding protein, putative
Grapevine	GSVIVG00030785001	RNA and export factor binding protein
Sorghum	Sb07g020860	THO complex subunit 4
Maize	GRMZM2G171921	THO complex subunit 4
Brachypodium	Bradi3g36320	RNA and export factor binding protein

Species	Orthologous genes	Putative function
Rice	LOC_Os07g36140	core histone H2A/H2B/H3/H4, putative, expressed
	LOC_Os08g33100	core histone H2A/H2B/H3/H4, putative, expressed
Arabidopsis	AT1G51060	histone H2A 10
	AT4G27230	histone H2A 2
	AT5G54640	Histone superfamily protein
Poplar	POPTR_0001s44140	HTA10; DNA binding
	POPTR_0011s13490	HTA10; DNA binding
Grapevine	GSVIVG00002735001	Histone H2A
	GSVIVG00002746001	Histone H2A
	GSVIVG00014580001	Histone H2A
Sorghum	Sb02g035650	Histone H2A

Additional File 2 cont.: Orthologous Proteins from Different Plant Species

Maize	GRMZM2G042047	Histone H2A
	GRMZM2G151726	Histone H2A
Brachypodium	Bradi1g25390	histone H2A.2

Species	Orthologous genes	Putative function
Rice	LOC_Os08g31810	RNA recognition motif containing protein, putative, expressed
Arabidopsis	AT5G04600	RNA-binding (RRM/RBD/RNP motifs) family protein
Poplar	POPTR_0010s24120	RNA recognition motif (RRM)-containing protein
Grapevine	GSVIVG00021851001	similar to RNA binding protein
Sorghum	Sb03g045720	MKI67 FHA domain-interacting nucleolar phosphoprotein
Maize	GRMZM2G056350	MKI67 FHA domain-interacting nucleolar phosphoprotein
Brachypodium	Bradi3g35650	RBP

Species	Orthologous genes	Putative function
Rice	LOC_Os04g25550	FACT complex subunit SPT16, putative, expressed
	LOC_Os08g31240	FACT complex subunit SPT16, putative, expressed
Arabidopsis	AT4G10710	global transcription factor C
Poplar	POPTR_0005s00690	SPT16 (global transcription factor C)
	POPTR_0005s00700	SPT16 (global transcription factor C)
	POPTR_0013s00590	SPT16 (global transcription factor C)
	POPTR_0013s00600	SPT16 (global transcription factor C)
Grapevine	GSVIVG00031308001	Global transcription factor group
Sorghum	Sb01g002390	FACT complex subunit SPT16
	Sb07g026150	FACT complex subunit SPT16
Maize	GRMZM5G806358	FACT complex subunit SPT16
Brachypodium	Bradi1g59920	FACT complex subunit SPT16
	Bradi1g59940	FACT complex subunit SPT16

Species	Orthologous genes	Putative function
Rice	LOC_Os08g29650	RNA recognition motif containing protein, expressed
Poplar	POPTR_0003s13030	nucleic acid binding / nucleotide binding
Grapevine	GSVIVG00019390001	Arginine/serine-rich-splicing factor
Sorghum	Sb07g019390	Arginine/serine-rich splicing factor 10
Maize	GRMZM2G031846	Arginine/serine-rich splicing factor 10
Brachypodium	Bradi1g29180	Alternative splicing regulator

Species	Orthologous genes	Putative function
Rice	LOC_Os08g23710	ribosomal protein L7Ae, putative, expressed
	LOC_Os09g32976	ribosomal protein L7Ae, putative, expressed
Arabidopsis	AT2G47610	Ribosomal protein L7Ae/L30e/S12e/Gadd45 family protein
	AT3G62870	Ribosomal protein L7Ae/L30e/S12e/Gadd45 family protein
Poplar	POPTR_0001s33400	60S ribosomal protein L7A (RPL7aB)

Additional File 2 cont.: Orthologous Proteins from Different Plant Species

	POPTR_0004s11250	60S ribosomal protein L7A (RPL7aB)
	POPTR_0004s11260	60S ribosomal protein L7A (RPL7aB)
	POPTR_0017s13510	60S ribosomal protein L7A (RPL7aB)
	POPTR_0017s13530	60S ribosomal protein L7A (RPL7aB)
Grapevine	GSVIVG00008585001	60S ribosomal protein L7a
	GSVIVG00033050001	60S ribosomal protein L7a
	GSVIVG00036540001	Structural constituent of ribosome
Sorghum	Sb02g029380	60S ribosomal protein L7a
	Sb02g029400	60S ribosomal protein L7a
Maize	GRMZM2G135654	60S ribosomal protein L7a
	GRMZM2G178968	60S ribosomal protein L7a
	GRMZM5G870752	60S ribosomal protein L7a
Brachypodium	Bradi4g34750	60S ribosomal protein L7a
	Bradi5g24680	60S ribosomal protein L7a

**Species**	**Orthologous genes**	**Putative function**
Rice	LOC_Os08g21840	50S ribosomal protein L15, putative, expressed
Arabidopsis	AT5G64670	Ribosomal protein L18e/L15 superfamily protein
Poplar	POPTR_0001s33180	ribosomal protein L15 family protein
	POPTR_0016s00420	ribosomal protein L15 family protein
Grapevine	GSVIVG00016205001	60S ribosomal protein L10, mitochondrial
Sorghum	Sb03g012270	60S ribosomal protein L10, mitochondrial
Maize	GRMZM2G058923	50S ribosomal protein L15
Brachypodium	Bradi3g20440	60S ribosomal protein L10, mitochondrial

**Species**	**Orthologous genes**	**Putative function**
Rice	LOC_Os05g16660	WD domain, G-beta repeat domain containing protein, expressed
	LOC_Os08g21660	WD domain, G-beta repeat domain containing protein, expressed
Arabidopsis	AT2G46280	TGF-beta receptor interacting protein 1
	AT2G46290	Transducin/WD40 repeat-like superfamily protein
Poplar	POPTR_0008s14090	eukaryotic translation initiation factor 3 subunit 2, putative / eIF-3 beta, putative / eIF3i, putative
	POPTR_0010s11040	eukaryotic translation initiation factor 3 subunit 2, putative / eIF-3 beta, putative / eIF3i, putative
Grapevine	GSVIVG00011562001	TGF-beta receptor-interacting protein 1
Sorghum	Sb02g003760	Eukaryotic translation initiation factor 3 subunit 2
Maize	GRMZM2G028834	Eukaryotic translation initiation factor 3 subunit 2
	GRMZM2G143330	Eukaryotic translation initiation factor 3 subunit 2
Brachypodium	Bradi4g14100	Eukaryotic translation initiation factor 3 subunit 2

**Species**	**Orthologous genes**	**Putative function**
Rice	LOC_Os04g51630	60S ribosomal protein L7, putative, expressed
	LOC_Os08g13690	60S ribosomal protein L7, putative, expressed
Arabidopsis	AT2G01250	Ribosomal protein L30/L7 family protein

Additional File 2 cont.: Orthologous Proteins from Different Plant Species

	AT2G44120	Ribosomal protein L30/L7 family protein
	AT3G13580	Ribosomal protein L30/L7 family protein
Poplar	POPTR_0006s07290	60S ribosomal protein L7 (RPL7C)
	POPTR_0008s00840	60S ribosomal protein L7 (RPL7D)
	POPTR_0010s25740	60S ribosomal protein L7 (RPL7D)
	POPTR_0018s13700	60S ribosomal protein L7 (RPL7D)
Grapevine	GSVIVG00029655001	60S ribosomal protein L7
Sorghum	Sb06g027740	60S ribosomal protein L7-2
	Sb07g006970	60S ribosomal protein L7-1
Maize	GRMZM2G094051	60S ribosomal protein L7-2
	GRMZM2G100225	60S ribosomal protein L7-1
	GRMZM2G178807	60S ribosomal protein L7-1
	GRMZM5G868433	60S ribosomal protein L7-2
Brachypodium	Bradi1g04950	60S ribosomal protein L7-2
	Bradi5g20900	60S ribosomal protein L7-2

**Species**	**Orthologous genes**	**Putative function**
Rice	LOC_Os04g52960	nucleolin, putative, expressed
	LOC_Os08g09350	gar2, putative, expressed
Arabidopsis	AT1G48920	nucleolin like 1
	AT3G18610	nucleolin like 2
Poplar	POPTR_0005s09600	ATRANGAP1 (RAN GTPASE-ACTIVATING PROTEIN 1); nucleic acid binding / nucleotide binding
	POPTR_0007s07800	ATRANGAP1 (RAN GTPASE-ACTIVATING PROTEIN 1); nucleic acid binding / nucleotide binding
Grapevine	GSVIVG00012042001	Nucleic acid binding protein
Sorghum	Sb01g019710	nucleolin
	Sb07g005510	Nucleic acid binding protein
Maize	GRMZM2G001850	NuM1 protein
	GRMZM2G131943	nucleolin
Brachypodium	Bradi3g14910	L. (clone na-481-5)
	Bradi5g22120	Nucleolin

**Species**	**Orthologous genes**	**Putative function**
Rice	LOC_Os08g06040	60S ribosomal protein L34, putative, expressed
	LOC_Os08g33920	60S ribosomal protein L34, putative, expressed
Sorghum	Sb07g003770	60S ribosomal protein L34
Maize	GRMZM2G032564	60S ribosomal protein L34

**Species**	**Orthologous genes**	**Putative function**
Rice	LOC_Os08g05880	Brix domain containing protein, putative, expressed
Arabidopsis	AT1G63780	Ribosomal RNA processing Brix domain protein
Poplar	POPTR_0001s06190	IMP4
	POPTR_0003s20010	IMP4
Grapevine	GSVIVG00000834001	U3 small nucleolar ribonucleoprotein protein imp4

Additional File 2 cont.: Orthologous Proteins from Different Plant Species

Sorghum	Sb10g028620	U3 small nucleolar ribonucleoprotein protein imp4
Maize	GRMZM2G066555	U3 snoRNP protein IMP4
Brachypodium	Bradi3g16690	U3 small nucleolar ribonucleoprotein protein imp4

**Species**	**Orthologous genes**	**Putative function**
Rice	LOC_Os08g05840	DNA topoisomerase 1, putative, expressed
Arabidopsis	AT5G55300	DNA topoisomerase I alpha
	AT5G55310	DNA topoisomerase 1 beta
Poplar	POPTR_0001s37040	TOP1BETA (DNA TOPOISOMERASE 1 BETA); DNA topoisomerase type I
	POPTR_0011s09520	TOP1BETA (DNA TOPOISOMERASE 1 BETA); DNA topoisomerase type I
Grapevine	GSVIVG00020301001	Topoisomerase I
	GSVIVG00030405001	Topoisomerase I
Sorghum	Sb07g003610	DNA topoisomerase I
Maize	GRMZM2G092451	DNA topoisomerase I
Brachypodium	Bradi3g16920	DNA topoisomerase I

**Species**	**Orthologous genes**	**Putative function**
Rice	LOC_Os08g04280	actin, putative, expressed
Arabidopsis	AT1G18450	actin-related protein 4
Poplar	POPTR_0012s05840	ATARP4; structural constituent of cytoskeleton
Grapevine	GSVIVG00008254001	Protein binding protein
Sorghum	Sb01g049880	Actin 6A
Maize	GRMZM2G015384	Actin 6A
Brachypodium	Bradi1g77930	Actin 6A

**Species**	**Orthologous genes**	**Putative function**
Rice	LOC_Os08g03640	60S acidic ribosomal protein P0, putative, expressed
	LOC_Os11g04070	60S acidic ribosomal protein P0, putative, expressed
	LOC_Os12g03880	60S acidic ribosomal protein P0, putative, expressed
Arabidopsis	AT2G40010	Ribosomal protein L10 family protein
	AT3G09200	Ribosomal protein L10 family protein
	AT3G11250	Ribosomal protein L10 family protein
Poplar	POPTR_0008s06620	60S acidic ribosomal protein P0 (RPP0A)
	POPTR_0010s19860	60S acidic ribosomal protein P0 (RPP0A)
Grapevine	GSVIVG00016313001	60S acidic ribosomal protein P0
	GSVIVG00032857001	60S acidic ribosomal protein P0
Sorghum	Sb07g002560	60S acidic ribosomal protein P0
	Sb09g028230	60S acidic ribosomal protein P0
Maize	GRMZM2G066460	60S acidic ribosomal protein P0
	GRMZM2G179976	60S acidic ribosomal protein P0
Brachypodium	Bradi3g14340	60S acidic ribosomal protein P0

Additional File 2 cont.: Orthologous Proteins from Different Plant Species

Species	Orthologous genes	Putative function
Rice	LOC_Os08g02400	40S ribosomal protein S13, putative, expressed
	LOC_Os08g02410	40S ribosomal protein S13, putative, expressed
Arabidopsis	AT3G60770	Ribosomal protein S13/S15
	AT4G00100	ribosomal protein S13A
Poplar	POPTR_0002s14780	40S ribosomal protein S13 (RPS13A)
	POPTR_0012s14440	ATRPS13A (ARABIDOPSIS THALIANA RIBOSOMAL PROTEIN S13A); structural constituent of ribosome
	POPTR_0014s06420	40S ribosomal protein S13 (RPS13A)
	POPTR_0015s14490	ATRPS13A (ARABIDOPSIS THALIANA RIBOSOMAL PROTEIN S13A); structural constituent of ribosome
Grapevine	GSVIVG00018537001	40S ribosomal protein S13
	GSVIVG00027637001	40S ribosomal protein S13
Sorghum	Sb07g001620	40S ribosomal protein S13
Maize	GRMZM2G158034	40S ribosomal protein S13
	GRMZM5G852185	40S ribosomal protein S13
Brachypodium	Bradi3g13800	40S ribosomal protein S13
	Bradi3g13810	40S ribosomal protein S13

Species	Orthologous genes	Putative function
Rice	LOC_Os08g02400	40S ribosomal protein S13, putative, expressed
	LOC_Os08g02410	40S ribosomal protein S13, putative, expressed
Arabidopsis	AT3G60770	Ribosomal protein S13/S15
	AT4G00100	ribosomal protein S13A
Poplar	POPTR_0002s14780	40S ribosomal protein S13 (RPS13A)
	POPTR_0012s14440	ATRPS13A (ARABIDOPSIS THALIANA RIBOSOMAL PROTEIN S13A); structural constituent of ribosome
	POPTR_0014s06420	40S ribosomal protein S13 (RPS13A)
	POPTR_0015s14490	ATRPS13A (ARABIDOPSIS THALIANA RIBOSOMAL PROTEIN S13A); structural constituent of ribosome
Grapevine	GSVIVG00018537001	40S ribosomal protein S13
	GSVIVG00027637001	40S ribosomal protein S13
Sorghum	Sb07g001620	40S ribosomal protein S13
Maize	GRMZM2G158034	40S ribosomal protein S13
	GRMZM5G852185	40S ribosomal protein S13
Brachypodium	Bradi3g13800	40S ribosomal protein S13
	Bradi3g13810	40S ribosomal protein S13

Species	Orthologous genes	Putative function
Rice	LOC_Os03g18690	26S protease regulatory subunit 4, putative, expressed
	LOC_Os07g49150	26S protease regulatory subunit 4, putative, expressed
Arabidopsis	AT2G20140	AAA-type ATPase family protein
	AT4G29040	regulatory particle AAA-ATPase 2A
Poplar	POPTR_0002s25390	RPT2a (regulatory particle AAA-ATPase 2a); ATPase
Grapevine	GSVIVG00028520001	26S protease regulatory subunit
Sorghum	Sb02g043840	26S protease regulatory subunit 4 homolog

Additional File 2 cont.: Orthologous Proteins from Different Plant Species

Maize	GRMZM2G056569	26S protease regulatory subunit 4 homolog
	GRMZM2G104373	26S protease regulatory subunit 4
Brachypodium	Bradi1g16750	26S protease regulatory subunit 4 homolog
	Bradi1g65070	26S protease regulatory subunit 4 homolog

**Species**	**Orthologous genes**	**Putative function**
Rice	LOC_Os07g47420	60S ribosome subunit biogenesis protein NIP7, putative, expressed
Arabidopsis	AT4G15770	RNA binding
Poplar	POPTR_0010s02520	RNA binding / protein binding
	POPTR_0034s00260	RNA binding / protein binding
Grapevine	GSVIVG00017749001	60S ribosome subunit biogenesis protein NIP7
	GSVIVG00038238001	60S ribosome subunit biogenesis protein NIP7
Sorghum	Sb04g035510	60S ribosome subunit biogenesis protein NIP7
Maize	GRMZM2G468932	60S ribosome subunit biogenesis protein NIP7
Brachypodium	Bradi1g18250	60S ribosome subunit biogenesis protein NIP7

**Species**	**Orthologous genes**	**Putative function**
Rice	LOC_Os07g46370	WD domain, G-beta repeat domain containing protein, expressed
Arabidopsis	AT1G18830	Transducin/WD40 repeat-like superfamily protein
	AT3G63460	transducin family protein / WD-40 repeat family protein
Poplar	POPTR_0001s26820	WD-40 repeat family protein
	POPTR_0009s06020	WD-40 repeat family protein
Grapevine	GSVIVG00001441001	transducin family protein / WD-40 repeat family protein
Sorghum	Sb02g041640	transducin family protein / WD-40 repeat family protein
Maize	GRMZM2G035985	EMB2221
	GRMZM2G152963	EMB2221
Brachypodium	Bradi1g19210	Nucleotide binding protein

**Species**	**Orthologous genes**	**Putative function**
Rice	LOC_Os07g44190	h/ACA ribonucleoprotein complex subunit 4, putative, expressed
Arabidopsis	AT3G57150	homologue of NAP57
Poplar	POPTR_0006s04270	NAP57 (Arabidopsis thaliana homologue of NAP57); pseudouridine synthase
	POPTR_0016s04070	NAP57 (Arabidopsis thaliana homologue of NAP57); pseudouridine synthase
Grapevine	GSVIVG00034188001	Centromere/microtubule binding protein cbf5
Sorghum	Sb03g009870	Centromere/microtubule binding protein cbf5
Maize	GRMZM2G044128	H/ACA ribonucleoprotein complex subunit 4
	GRMZM2G172956	H/ACA ribonucleoprotein complex subunit 4
Brachypodium	Bradi2g04950	H/ACA ribonucleoprotein complex subunit 4
	Bradi4g38770	H/ACA ribonucleoprotein complex subunit 4

**Species**	**Orthologous genes**	**Putative function**
Rice	LOC_Os03g27260	40S ribosomal protein S6, putative, expressed

Additional File 2 cont.: Orthologous Proteins from Different Plant Species

	LOC_Os07g42950	40S ribosomal protein S6, putative, expressed
Arabidopsis	AT4G31700	ribosomal protein S6
	AT5G10360	Ribosomal protein S6e
Poplar	POPTR_0001s31850	EMB3010 (embryo defective 3010); structural constituent of ribosome
	POPTR_0002s09970	EMB3010 (embryo defective 3010); structural constituent of ribosome
	POPTR_0005s07380	EMB3010 (embryo defective 3010); structural constituent of ribosome
	POPTR_0005s17280	EMB3010 (embryo defective 3010); structural constituent of ribosome
	POPTR_0007s05090	EMB3010 (embryo defective 3010); structural constituent of ribosome
Grapevine	GSVIVG00003418001	S6 ribosomal protein
	GSVIVG00009072001	S6 ribosomal protein
	GSVIVG00015776001	S6 ribosomal protein
Sorghum	Sb02g039650	40S ribosomal protein S6
	Sb08g015010	40S ribosomal protein S6
Maize	GRMZM2G054136	40S ribosomal protein S6
	GRMZM5G851698	40S ribosomal protein S6
Brachypodium	Bradi1g60730	40S ribosomal protein S6

**Species**	**Orthologous genes**	**Putative function**
Rice	LOC_Os03g08440	ribosomal protein S2, putative, expressed
	LOC_Os07g42450	ribosomal protein S2, putative, expressed
Arabidopsis	AT1G72370	40s ribosomal protein SA
Poplar	POPTR_0001s16430	P40; structural constituent of ribosome
	POPTR_0003s06880	RPSAb (40S ribosomal protein SA B); structural constituent of ribosome
	POPTR_0012s11830	RPSAb (40S ribosomal protein SA B); structural constituent of ribosome
	POPTR_0015s12460	P40; structural constituent of ribosome
Grapevine	GSVIVG00016695001	40S ribosomal protein SA
Sorghum	Sb02g039260	40S ribosomal protein SA
	Sb02g039270	40S ribosomal protein SA
	Sb05g027730	40S ribosomal protein SA
Maize	GRMZM2G092719	40S ribosomal protein SA
	GRMZM2G099657	40S ribosomal protein SA
	GRMZM2G126821	40S ribosomal protein SA
	GRMZM2G145308	40S ribosomal protein SA
	GRMZM2G159237	40S ribosomal protein SA
Brachypodium	Bradi1g20880	40S ribosomal protein SA
	Bradi1g23780	40S ribosomal protein SA
	Bradi1g72480	40S ribosomal protein

**Species**	**Orthologous genes**	**Putative function**
Rice	LOC_Os07g42170	60S ribosomal protein, putative, expressed
Sorghum	Sb04g004680	60S ribosomal protein L27a-2
Maize	GRMZM2G427468	60S ribosomal protein L27a-2

Additional File 2 cont.: Orthologous Proteins from Different Plant Species

Species	Orthologous genes	Putative function
Rice	LOC_Os07g41750	40S ribosomal protein S3-1, putative, expressed
Sorghum	Sb02g038990	40S ribosomal protein S3
Maize	GRMZM2G093902	40S ribosomal protein S3
Brachypodium	Bradi1g21730	40S ribosomal protein S3

Species	Orthologous genes	Putative function
Rice	LOC_Os07g41740	PHD finger protein, putative, expressed
Arabidopsis	AT5G20510	alfin-like 5
Sorghum	Sb02g038980	nucleic acid binding protein
Maize	GRMZM2G038066	nucleic acid binding protein
Brachypodium	Bradi1g21740	PHD zinc finger protein

Species	Orthologous genes	Putative function
Rice	LOC_Os07g41260	PPR repeat domain containing protein, putative, expressed
Arabidopsis	AT5G60960	Pentatricopeptide repeat (PPR) superfamily protein
Poplar	POPTR_0017s02600	pentatricopeptide (PPR) repeat-containing protein
Grapevine	GSVIVG00008125001	Pentatricopeptide repeat-containing protein
Sorghum	Sb02g038690	Pentatricopeptide repeat-containing protein
Maize	GRMZM2G021567	Pentatricopeptide repeat-containing protein, mitochondrial
Brachypodium	Bradi1g22060	Pentatricopeptide repeat-containing protein, mitochondrial

Species	Orthologous genes	Putative function
Rice	LOC_Os07g40930	WD repeat-containing protein 12, putative, expressed
Arabidopsis	AT5G15550	Transducin/WD40 repeat-like superfamily protein
Poplar	POPTR_0017s12880	transducin family protein / WD-40 repeat family protein
Grapevine	GSVIVG00032921001	WD-repeat protein
Sorghum	Sb02g038500	WD-repeat protein
Maize	GRMZM2G050501	Transducin family protein
Brachypodium	Bradi1g22300	WD-repeat protein

Species	Orthologous genes	Putative function
Rice	LOC_Os01g61920	Core histone H2A/H2B/H3/H4 domain containing protein, putative, expressed
	LOC_Os02g45940	Core histone H2A/H2B/H3/H4 domain containing protein, putative, expressed
	LOC_Os03g02780	Core histone H2A/H2B/H3/H4 domain containing protein, putative, expressed
	LOC_Os04g49420	Core histone H2A/H2B/H3/H4 domain containing protein, putative, expressed
	LOC_Os05g38740	Core histone H2A/H2B/H3/H4 domain containing protein, putative, expressed
	LOC_Os05g39050	Core histone H2A/H2B/H3/H4 domain containing protein, putative, expressed
	LOC_Os07g36500	Core histone H2A/H2B/H3/H4 domain containing protein, putative, expressed
	LOC_Os09g26340	Core histone H2A/H2B/H3/H4 domain containing protein, putative, expressed
	LOC_Os09g38020	Core histone H2A/H2B/H3/H4 domain containing protein, putative, expressed
	LOC_Os10g39410	Core histone H2A/H2B/H3/H4 domain containing protein, putative, expressed

Additional File 2 cont.: Orthologous Proteins from Different Plant Species

Arabidopsis	AT1G07660	Histone superfamily protein
	AT1G07820	Histone superfamily protein
	AT2G28740	histone H4
	AT3G45930	Histone superfamily protein
	AT3G46320	Histone superfamily protein
	AT3G53730	Histone superfamily protein
	AT5G59690	Histone superfamily protein
	AT5G59970	Histone superfamily protein
Poplar	POPTR_0005s11740	histone H4
	POPTR_0005s11770	histone H4
	POPTR_0006s18360	histone H4
	POPTR_0007s14000	histone H4
	POPTR_0007s14020	histone H4
	POPTR_0007s14090	histone H4
	POPTR_0008s04720	histone H4
	POPTR_0010s22080	histone H4
	POPTR_0010s22090	histone H4
	POPTR_0018s10040	histone H4
	POPTR_0018s10070	histone H4
	POPTR_0018s10080	histone H4
	POPTR_0168s00200	histone H4
Grapevine	GSVIVG00016576001	Histone H4
Sorghum	Sb01g030460	Histone H4
	Sb01g049250	Histone H4
	Sb02g025440	Histone H4
	Sb02g032240	Histone H4
	Sb03g004840	Histone H4
	Sb03g004870	Histone H4
	Sb03g004890	Histone H4
	Sb03g039090	Histone H4
	Sb04g031620	Histone H4
	Sb06g026490	Histone H4
	Sb09g022920	Histone H4
Maize	AC196961.2_FG003	Histone H4
	AC212565.3_FG001	similar to germinal histone H4 gene
	AC233865.1_FG001	Histone H4
	GRMZM2G016232	Histone H4
	GRMZM2G063896	Histone H4
	GRMZM2G072855	Histone H4
	GRMZM2G073275	Histone H4
	GRMZM2G084195	Histone H4
	GRMZM2G143780	Histone H4
	GRMZM2G149178	Histone H4
	GRMZM2G181153	Histone H4

	GRMZM2G332838	similar to germinal histone H4 gene
	GRMZM2G349651	Histone H4
	GRMZM2G421279	Histone H4
	GRMZM2G479684	Histone H4
Brachypodium	Bradi1g05980	Histone H4
	Bradi1g06000	Histone H4
	Bradi1g68190	Histone H4
	Bradi1g77230	Histone H4
	Bradi2g22790	Histone H4
	Bradi2g22990	Histone H4
	Bradi2g23010	Histone H4
	Bradi3g51930	Histone H4
	Bradi4g06040	Histone H4
	Bradi4g30960	Histone H4
	Bradi4g37340	Histone H4
	Bradi5g19350	Histone H4
	Bradi5g19360	Histone H4

Species	Orthologous genes	Putative function
Rice	LOC_Os07g36140	core histone H2A/H2B/H3/H4, putative, expressed
	LOC_Os08g33100	core histone H2A/H2B/H3/H4, putative, expressed
Arabidopsis	AT1G51060	histone H2A 10
	AT4G27230	histone H2A 2
	AT5G54640	Histone superfamily protein
Poplar	POPTR_0001s44140	HTA10; DNA binding
	POPTR_0011s13490	HTA10; DNA binding
Grapevine	GSVIVG00002735001	Histone H2A
	GSVIVG00002746001	Histone H2A
	GSVIVG00014580001	Histone H2A
Sorghum	Sb02g035650	Histone H2A
Maize	GRMZM2G042047	Histone H2A
	GRMZM2G151726	Histone H2A
Brachypodium	Bradi1g25390	histone H2A.2

Species	Orthologous genes	Putative function
Rice	LOC_Os07g36130	core histone H2A/H2B/H3/H4, putative, expressed
	LOC_Os12g25120	core histone H2A/H2B/H3/H4, putative, expressed
Sorghum	Sb02g035640	Histone H2A
Maize	GRMZM2G041381	Histone H2A
	GRMZM2G151826	histone H2A.1
Brachypodium	Bradi1g25400	histone H2A.7

Species	Orthologous genes	Putative function

Additional File 2 cont.: Orthologous Proteins from Different Plant Species

Rice	LOC_Os07g32350	WD domain, G-beta repeat domain containing protein, expressed
Arabidopsis	AT1G29320	Transducin/WD40 repeat-like superfamily protein
Poplar	POPTR_0012s09680	transducin family protein / WD-40 repeat family protein
	POPTR_0015s10440	transducin family protein / WD-40 repeat family protein
Grapevine	GSVIVG00013111001	WD-repeat protein
Sorghum	Sb02g034230	WD-repeat protein
Maize	GRMZM2G028887	WD-repeat protein
	GRMZM2G154864	WD-repeat protein
Brachypodium	Bradi1g26700	WD-repeat protein

**Species**	**Orthologous genes**	**Putative function**
Rice	LOC_Os07g25440	WD domain, G-beta repeat domain containing protein, expressed
Arabidopsis	AT2G40360	Transducin/WD40 repeat-like superfamily protein
Poplar	POPTR_0004s23120	transducin family protein / WD-40 repeat family protein
	POPTR_0022s00810	transducin family protein / WD-40 repeat family protein
Grapevine	GSVIVG00004116001	Ribosome biogenesis protein bop1
Sorghum	Sb03g013640	transducin / WD-40 repeat protein
Brachypodium	Bradi3g39580	transducin / WD-40 repeat protein
	Bradi3g60760	transducin / WD-40 repeat protein

**Species**	**Orthologous genes**	**Putative function**
Rice	LOC_Os01g33050	ribosomal protein L24, putative, expressed
	LOC_Os07g19190	ribosomal protein L24, putative, expressed
Arabidopsis	AT2G44860	Ribosomal protein L24e family protein
Poplar	POPTR_0004s19930	60S ribosomal protein L24, putative
	POPTR_0009s15070	60S ribosomal protein L24, putative
Grapevine	GSVIVG00031394001	60S ribosomal protein L24
	GSVIVG00033788001	60S ribosomal protein L24
Sorghum	Sb09g028470	60S ribosomal protein L24
Brachypodium	Bradi2g03070	60S ribosomal protein L24
	Bradi4g01560	60S ribosomal protein L24

**Species**	**Orthologous genes**	**Putative function**
Rice	LOC_Os03g60390	PHD finger protein, putative, expressed
	LOC_Os07g12910	PHD finger protein, putative, expressed
Arabidopsis	AT3G11200	alfin-like 2
	AT5G05610	alfin-like 1
Poplar	POPTR_0008s06920	AL1 (ALFIN-LIKE 1); DNA binding / methylated histone residue binding
	POPTR_0010s19550	AL1 (ALFIN-LIKE 1); DNA binding / methylated histone residue binding
Grapevine	GSVIVG00011162001	PHD4
Sorghum	Sb01g003420	PHD finger protein
	Sb02g006980	PHD finger protein

Additional File 2 cont.: Orthologous Proteins from Different Plant Species

Maize	AC225147.4_FG003	PHD finger protein
	GRMZM2G047316	PHD finger protein
Brachypodium	Bradi1g03900	PHD finger protein
	Bradi1g53660	PHD finger protein

**Species**	**Orthologous genes**	**Putative function**
Rice	LOC_Os07g12320	WD domain, G-beta repeat domain containing protein, expressed
Arabidopsis	AT2G47990	transducin family protein / WD-40 repeat family protein
Poplar	POPTR_0002s22790	SWA1 (SLOW WALKER1); nucleotide binding
	POPTR_0014s13130	SWA1 (SLOW WALKER1); nucleotide binding
Grapevine	GSVIVG00011022001	U3 small nucleolar RNA-associated protein
Maize	AC212570.3_FG006	Small nucleolar ribonucleoprotein complex subunit
Brachypodium	Bradi1g53790	Small nucleolar ribonucleoprotein complex subunit

**Species**	**Orthologous genes**	**Putative function**
Rice	LOC_Os01g59990	ribosomal protein L24, putative, expressed
	LOC_Os05g40820	ribosomal protein L24, putative, expressed
	LOC_Os07g12250	ribosomal protein L24, putative, expressed
Arabidopsis	AT2G36620	ribosomal protein L24
	AT3G53020	Ribosomal protein L24e family protein
Poplar	POPTR_0003s12330	RPL24A (ribosomal protein L24); structural constituent of ribosome
	POPTR_0004s08370	RPL24A (ribosomal protein L24); structural constituent of ribosome
	POPTR_0012s13380	RPL24A (ribosomal protein L24); structural constituent of ribosome
	POPTR_0015s13330	RPL24A (ribosomal protein L24); structural constituent of ribosome
Grapevine	GSVIVG00010601001	60S ribosomal protein L24
	GSVIVG00033743001	60S ribosomal protein L24
	GSVIVG00034676001	60S ribosomal protein L24
Sorghum	Sb01g015240	60S ribosomal protein L24
	Sb09g023800	60S ribosomal protein L24
Maize	GRMZM2G074898	60S ribosomal protein L24
	GRMZM2G110328	60S ribosomal protein L24
	GRMZM2G142640	60S ribosomal protein L24
Brachypodium	Bradi2g53220	60S ribosomal protein L24

**Species**	**Orthologous genes**	**Putative function**
Rice	LOC_Os03g59310	ribosomal protein, putative, expressed
	LOC_Os07g10660	ribosomal protein, putative, expressed
Arabidopsis	AT1G58380	Ribosomal protein S5 family protein
	AT1G58684	Ribosomal protein S5 family protein
	AT1G58983	Ribosomal protein S5 family protein
	AT1G59359	Ribosomal protein S5 family protein
	AT2G41840	Ribosomal protein S5 family protein

Additional File 2 cont.: Orthologous Proteins from Different Plant Species

	AT3G57490	Ribosomal protein S5 family protein
Poplar	POPTR_0001s26400	XW6; structural constituent of ribosome
	POPTR_0006s05090	40S ribosomal protein S2 (RPS2D)
	POPTR_0016s05530	40S ribosomal protein S2 (RPS2C)
Grapevine	GSVIVG00033299001	40S ribosomal protein S2
Sorghum	Sb01g004250	40S ribosomal protein S2
	Sb01g004260	40S ribosomal protein S2
	Sb02g006200	40S ribosomal protein S2
Maize	AC210013.4_FG019	40S ribosomal protein S2
	GRMZM2G092663	40S ribosomal protein S2
	GRMZM2G168149	40S ribosomal protein S2
Brachypodium	Bradi1g04660	40S ribosomal protein S2
	Bradi1g54270	40S ribosomal protein S2

**Species**	**Orthologous genes**	**Putative function**
Rice	LOC_Os07g10350	S1 RNA binding domain containing protein, expressed
Arabidopsis	AT3G11964	RNA binding;RNA binding
Poplar	POPTR_0016s06250	RNA binding
Grapevine	GSVIVG00033430001	rRNA biogenesis protein
Sorghum	Sb02g006000	pre-rRNA processing protein RRP5
Maize	GRMZM2G039746	pre-rRNA processing protein RRP5
	GRMZM2G398527	pre-rRNA processing protein RRP5
Brachypodium	Bradi1g38230	pre-rRNA processing protein RRP5

**Species**	**Orthologous genes**	**Putative function**
Rice	LOC_Os03g47800	RNA recognition motif containing protein, expressed
	LOC_Os07g08960	RNA recognition motif containing protein, expressed
Arabidopsis	AT1G60650	RNA-binding (RRM/RBD/RNP motifs) family protein with retrovirus zinc finger-like domain
	AT5G04280	RNA-binding (RRM/RBD/RNP motifs) family protein with retrovirus zinc finger-like domain
Poplar	POPTR_0008s03290	glycine-rich RNA-binding protein
	POPTR_0010s23550	glycine-rich RNA-binding protein
Grapevine	GSVIVG00016195001	Glycine-rich RNA-binding protein
	GSVIVG00033666001	Glycine-rich RNA-binding protein
Sorghum	Sb01g011910	Glycine-rich RNA-binding protein 7
	Sb02g004930	Glycine-rich RNA-binding protein 7
Maize	GRMZM2G082931	Glycine-rich RNA-binding protein 7
	GRMZM2G083783	Glycine-rich RNA-binding protein
	GRMZM2G161242	Glycine-rich RNA-binding protein
Brachypodium	Bradi1g12460	Glycine-rich RNA-binding protein
	Bradi1g55020	Glycine-rich RNA-binding protein 7

**Species**	**Orthologous genes**	**Putative function**

Additional File 2 cont.: Orthologous Proteins from Different Plant Species

Rice	LOC_Os07g08880	ES43 protein, putative, expressed
Arabidopsis	AT4G39100	PHD finger family protein / bromo-adjacent homology (BAH) domain-containing protein
Poplar	POPTR_0004s16700	SHL1 (short life); transcription factor/ zinc-mediated transcriptional activator
	POPTR_0009s12380	SHL1 (short life); transcription factor/ zinc-mediated transcriptional activator
Grapevine	GSVIVG00024220001	Ebs-bah-phd domain-containing protein
Sorghum	Sb02g004890	SHL1
Maize	GRMZM2G097726	SHL1
Brachypodium	Bradi1g55090	SHL1

**Species**	**Orthologous genes**	**Putative function**
Rice	LOC_Os07g08660	40S ribosomal protein S15, putative, expressed
Arabidopsis	AT1G04270	cytosolic ribosomal protein S15
	AT5G09510	Ribosomal protein S19 family protein
Poplar	POPTR_0002s04420	40S ribosomal protein S15 (RPS15C)
	POPTR_0005s24120	40S ribosomal protein S15 (RPS15C)
	POPTR_0010s08750	40S ribosomal protein S15 (RPS15D)
Grapevine	GSVIVG00000754001	40S ribosomal protein S15
Sorghum	Sb02g004720	40S ribosomal protein S15
	Sb09g025000	40S ribosomal protein S15
Maize	GRMZM2G080222	40S ribosomal protein S15
	GRMZM2G111172	40S ribosomal protein S15
	GRMZM2G156110	40S ribosomal protein S15
Brachypodium	Bradi1g55280	40S ribosomal protein S15

**Species**	**Orthologous genes**	**Putative function**
Rice	LOC_Os03g58204	ribosomal protein L4, putative, expressed
	LOC_Os07g08330	ribosomal protein L4, putative, expressed
Arabidopsis	AT3G09630	Ribosomal protein L4/L1 family
	AT5G02870	Ribosomal protein L4/L1 family
Poplar	POPTR_0006s13470	60S ribosomal protein L4/L1 (RPL4A)
	POPTR_0006s13480	60S ribosomal protein L4/L1 (RPL4A)
	POPTR_0016s08520	60S ribosomal protein L4/L1 (RPL4A)
Grapevine	GSVIVG00025547001	60S ribosomal protein L4
Sorghum	Sb01g005210	60S ribosomal protein L4
	Sb02g004510	60S ribosomal protein L4
Maize	GRMZM2G007695	60S ribosomal protein L4
	GRMZM2G018197	60S ribosomal protein L4
	GRMZM2G068952	60S ribosomal protein L4
Brachypodium	Bradi1g05510	60S ribosomal protein L4
	Bradi1g55510	60S ribosomal protein L4

**Species**	**Orthologous genes**	**Putative function**

Additional File 2 cont.: Orthologous Proteins from Different Plant Species

Rice	LOC_Os06g51380	ROOT HAIRLESS 1, putative, expressed
	LOC_Os07g07580	ROOT HAIRLESS 1, putative, expressed
Arabidopsis	AT1G48380	root hair initiation protein root hairless 1 (RHL1)
Poplar	POPTR_0004s06800	RHL1 (ROOT HAIRLESS 1); DNA binding / protein binding
Grapevine	GSVIVG00027119001	DNA-binding protein RHL1
Sorghum	Sb10g031135	ROOT HAIRLESS 1
Maize	GRMZM2G406101	ROOT HAIRLESS 1

**Species**	**Orthologous genes**	**Putative function**
Rice	LOC_Os07g07220	LSM domain containing protein, expressed
Arabidopsis	AT4G20440	small nuclear ribonucleoprotein associated protein B
	AT5G44500	Small nuclear ribonucleoprotein family protein
Poplar	POPTR_0001s43060	small nuclear ribonucleoprotein associated protein B, putative / snRNP-B, putative / Sm protein B, putative
	POPTR_0011s15920	small nuclear ribonucleoprotein associated protein B, putative / snRNP-B, putative / Sm protein B, putative
Grapevine	GSVIVG00034611001	Small nuclear ribonucleoprotein-associated protein
Sorghum	Sb02g003850	Small nuclear ribonucleoprotein-associated protein B
Maize	GRMZM2G021149	Small nuclear ribonucleoprotein-associated protein B
	GRMZM2G022041	Small nuclear ribonucleoprotein-associated protein B

**Species**	**Orthologous genes**	**Putative function**
Rice	LOC_Os07g06980	histone deacetylase, putative, expressed
Arabidopsis	AT3G18520	histone deacetylase 15
Poplar	POPTR_0012s05730	HDA15; histone deacetylase
Grapevine	GSVIVG00008242001	Histone deacetylase hda1
Sorghum	Sb02g003690	Histone deacetylase HDA110 isoform 2
Maize	GRMZM2G107309	Histone deacetylase HDA110 isoform 1
Brachypodium	Bradi1g56740	Histone deacetylase RPD3/HDA1 class II

**Species**	**Orthologous genes**	**Putative function**
Rice	LOC_Os07g05580	ribosomal protein L7Ae, putative, expressed
Arabidopsis	AT1G15930	Ribosomal protein L7Ae/L30e/S12e/Gadd45 family protein
	AT2G32060	Ribosomal protein L7Ae/L30e/S12e/Gadd45 family protein
Poplar	POPTR_0001s14870	40S ribosomal protein S12 (RPS12C)
	POPTR_0002s05710	40S ribosomal protein S12 (RPS12C)
	POPTR_0003s17960	40S ribosomal protein S12 (RPS12C)
	POPTR_0005s22790	40S ribosomal protein S12 (RPS12C)
Grapevine	GSVIVG00023591001	40S ribosomal protein S12
	GSVIVG00034219001	40S ribosomal protein S12
Sorghum	Sb02g006810	40S ribosomal protein S12
	Sb09g020940	40S ribosomal protein S12
	Sb09g029400	40S ribosomal protein S12

Additional File 2 cont.: Orthologous Proteins from Different Plant Species

Maize	GRMZM2G063340	40S ribosomal protein S12
	GRMZM2G139900	40S ribosomal protein S12
Brachypodium	Bradi1g06520	40S ribosomal protein S12
	Bradi1g53740	40S ribosomal protein S12

**Species**	**Orthologous genes**	**Putative function**
Rice	LOC_Os07g03240	RNA recognition motif containing protein, putative, expressed
Sorghum	Sb02g002070	RNA-binding protein
Maize	GRMZM2G006071	RNA-binding protein
	GRMZM2G042343	Ribonucleoprotein 1
Brachypodium	Bradi1g57570	RNA-binding protein

**Species**	**Orthologous genes**	**Putative function**
Rice	LOC_Os06g09570	nucleolar GTP-binding protein 1, putative, expressed
	LOC_Os07g01920	nucleolar GTP-binding protein 1, putative, expressed
Arabidopsis	AT1G50920	Nucleolar GTP-binding protein
Poplar	POPTR_0001s26610	GTP-binding protein-related
	POPTR_0009s05870	GTP-binding protein-related
Grapevine	GSVIVG00034588001	GTP binding protein
Sorghum	Sb10g006410	GTP binding protein
Maize	GRMZM2G013318	GTP binding protein
	GRMZM2G156986	nucleolar GTP-binding protein
Brachypodium	Bradi1g46650	GTP binding protein

**Species**	**Orthologous genes**	**Putative function**
Rice	LOC_Os07g01490	kinesin motor domain containing protein, putative, expressed
Arabidopsis	AT4G05190	kinesin 5
	AT4G21270	kinesin 1
Poplar	POPTR_0004s03170	ATK1 (ARABIDOPSIS THALIANA KINESIN 1); microtubule motor/ minus-end-directed microtubule motor
	POPTR_0011s03380	ATK1 (ARABIDOPSIS THALIANA KINESIN 1); microtubule motor/ minus-end-directed microtubule motor
Grapevine	GSVIVG00007336001	Kinesin
Sorghum	Sb02g000560	Kinesin-1
Maize	GRMZM2G436981	Kinesin
Brachypodium	Bradi1g59490	Kinesin

**Species**	**Orthologous genes**	**Putative function**
Rice	LOC_Os06g48350	CPuORF14 - conserved peptide uORF-containing transcript, expressed
	LOC_Os06g48355	expressed protein
	LOC_Os09g15770	CPuORF13 - conserved peptide uORF-containing transcript, expressed
	LOC_Os09g15775	expressed protein
Arabidopsis	AT1G36730	Translation initiation factor IF2/IF5
	AT1G77840	Translation initiation factor IF2/IF5
Poplar	POPTR_0004s09250	eukaryotic translation initiation factor 5, putative / eIF-5, putative

Additional File 2 cont.: Orthologous Proteins from Different Plant Species

	POPTR_0004s11110	eukaryotic translation initiation factor 5, putative / eIF-5, putative
	POPTR_0005s14880	eukaryotic translation initiation factor 5, putative / eIF-5, putative
Grapevine	GSVIVG00008949001	Eukaryotic translation initiation factor
Sorghum	Sb01g038080	Eukaryotic translation initiation factor 5
	Sb04g003550	Eukaryotic translation initiation factor 5
Maize	GRMZM2G369939	Eukaryotic translation initiation factor 5
Brachypodium	Bradi3g03430	Translation initiation factor eIF5
	Bradi3g03850	Translation initiation factor eIF5

**Species**	**Orthologous genes**	**Putative function**
Rice	LOC_Os06g48350	CPuORF14 - conserved peptide uORF-containing transcript, expressed
	LOC_Os06g48355	expressed protein
	LOC_Os09g15770	CPuORF13 - conserved peptide uORF-containing transcript, expressed
	LOC_Os09g15775	expressed protein
Arabidopsis	AT1G36730	Translation initiation factor IF2/IF5
	AT1G77840	Translation initiation factor IF2/IF5
Poplar	POPTR_0004s09250	eukaryotic translation initiation factor 5, putative / eIF-5, putative
	POPTR_0004s11110	eukaryotic translation initiation factor 5, putative / eIF-5, putative
	POPTR_0005s14880	eukaryotic translation initiation factor 5, putative / eIF-5, putative
Grapevine	GSVIVG00008949001	Eukaryotic translation initiation factor
Sorghum	Sb01g038080	Eukaryotic translation initiation factor 5
	Sb04g003550	Eukaryotic translation initiation factor 5
Maize	GRMZM2G369939	Eukaryotic translation initiation factor 5
Brachypodium	Bradi3g03430	Translation initiation factor eIF5
	Bradi3g03850	Translation initiation factor eIF5

**Species**	**Orthologous genes**	**Putative function**
Rice	LOC_Os06g48230	DNA-directed RNA polymerases I, II, and III subunit RPABC3, putative, expressed
Arabidopsis	AT1G54250	RNA polymerase Rpb8
	AT3G59600	RNA polymerase Rpb8
Poplar	POPTR_0013s12480	NRPB8B; DNA-directed RNA polymerase
Grapevine	GSVIVG00038099001	RNA polymerase
Sorghum	Sb01g001330	DNA-directed RNA polymerases I, II, and III 17.1 kDa polypeptide
	Sb01g001420	DNA-directed RNA polymerases I, II, and III 17.1 kDa polypeptide
Maize	GRMZM2G034326	DNA-directed RNA polymerases I, II, and III 17.1 kDa polypeptide
Brachypodium	Bradi1g33850	RNA polymerase I, II and III 16.5 kDa subunit
	Bradi5g20710	RNA polymerase I, II and III 16.5 kDa subunit

**Species**	**Orthologous genes**	**Putative function**
Rice	LOC_Os06g46890	zinc finger C-x8-C-x5-C-x3-H type family protein, expressed
Sorghum	Sb10g027520	Zinc finger C-x8-C-x5-C-x3-H type family protein
Maize	GRMZM2G044398	Zinc finger C-x8-C-x5-C-x3-H type family protein

Additional File 2 cont.: Orthologous Proteins from Different Plant Species

Species	Orthologous genes	Putative function
Rice	LOC_Os02g07260	phosphoglycerate kinase protein, putative, expressed
	LOC_Os06g45710	phosphoglycerate kinase protein, putative, expressed
Arabidopsis	AT1G79550	phosphoglycerate kinase
Poplar	POPTR_0008s08400	PGK (PHOSPHOGLYCERATE KINASE); phosphoglycerate kinase
	POPTR_0010s17870	PGK (PHOSPHOGLYCERATE KINASE); phosphoglycerate kinase
Sorghum	Sb04g004690	Phosphoglycerate kinase
	Sb10g026710	Phosphoglycerate kinase
Maize	GRMZM2G382914	Phosphoglycerate kinase
Brachypodium	Bradi3g05220	Phosphoglycerate kinase, cytosolic

Species	Orthologous genes	Putative function
Rice	LOC_Os06g43690	WD repeat-containing protein, putative, expressed
Arabidopsis	AT4G04940	transducin family protein / WD-40 repeat family protein
Poplar	POPTR_0006s07850	transducin family protein / WD-40 repeat family protein
Grapevine	GSVIVG00034133001	WD-repeat protein
Sorghum	Sb10g025320	WD-repeat protein
Maize	GRMZM2G087712	WD-repeat protein
Brachypodium	Bradi1g30580	WD-repeat protein

Species	Orthologous genes	Putative function
Rice	LOC_Os02g10080	zinc finger C-x8-C-x5-C-x3-H type family protein, expressed
	LOC_Os06g41384	zinc finger C-x8-C-x5-C-x3-H type family protein, expressed
Arabidopsis	AT3G12130	KH domain-containing protein / zinc finger (CCCH type) family protein
	AT5G06770	KH domain-containing protein / zinc finger (CCCH type) family protein
Poplar	POPTR_0001s05070	KH domain-containing protein / zinc finger (CCCH type) family protein
	POPTR_0003s21780	KH domain-containing protein / zinc finger (CCCH type) family protein
	POPTR_0016s04590	KH domain-containing protein / zinc finger (CCCH type) family protein
	POPTR_0016s04690	KH domain-containing protein / zinc finger (CCCH type) family protein
Grapevine	GSVIVG00001369001	Zinc finger CCCH domain-containing protein 52
	GSVIVG00025360001	Zinc finger CCCH domain-containing protein 36
	GSVIVG00033508001	Zinc finger CCCH domain-containing protein 36
Sorghum	Sb04g006450	Zinc finger CCCH domain-containing protein 14
	Sb10g024330	Zinc finger C-x8-C-x5-C-x3-H type family protein
Maize	GRMZM2G056920	Zinc finger CCCH domain-containing protein 44
	GRMZM2G110402	Zinc finger C-x8-C-x5-C-x3-H type family protein
	GRMZM2G151689	Zinc finger C-x8-C-x5-C-x3-H type family protein
Brachypodium	Bradi1g35920	Zinc finger C-x8-C-x5-C-x3-H type family protein
	Bradi3g06940	Zinc finger CCCH domain-containing protein 14

Species	Orthologous genes	Putative function

Additional File 2 cont.: Orthologous Proteins from Different Plant Species

Rice	LOC_Os06g40600	elongation factor, putative, expressed
Arabidopsis	AT1G06220	Ribosomal protein S5/Elongation factor G/III/V family protein
	AT5G25230	Ribosomal protein S5/Elongation factor G/III/V family protein
Poplar	POPTR_0006s20500	MEE5 (MATERNAL EFFECT EMBRYO ARREST 5); GTP binding / GTPase/ translation elongation factor/ translation factor, nucleic acid binding
	POPTR_0018s12280	MEE5 (MATERNAL EFFECT EMBRYO ARREST 5); GTP binding / GTPase/ translation elongation factor/ translation factor, nucleic acid binding
Grapevine	GSVIVG00012881001	116 kD U5 small nuclear ribonucleoprotein component
Sorghum	Sb10g023820	U5 small nuclear ribonucleoprotein component
Maize	GRMZM2G093987	U5 small nuclear ribonucleoprotein component
	GRMZM2G316232	U5 small nuclear ribonucleoprotein component
Brachypodium	Bradi1g36340	U5 small nuclear ribonucleoprotein component

Species	Orthologous genes	Putative function
Rice	LOC_Os02g10640	26S protease regulatory subunit, putative, expressed
	LOC_Os06g40560	26S protease regulatory subunit S10B, putative, expressed
Arabidopsis	AT1G45000	AAA-type ATPase family protein
	AT5G43010	regulatory particle triple-A ATPase 4A
Poplar	POPTR_0002s03280	26S proteasome regulatory complex subunit p42D, putative
	POPTR_0004s16600	26S proteasome regulatory complex subunit p42D, putative
	POPTR_0005s25320	26S proteasome regulatory complex subunit p42D, putative
	POPTR_0009s12330	RPT4A; ATPase
Grapevine	GSVIVG00009774001	26S protease regulatory subunit S10b
	GSVIVG00023828001	26S protease regulatory subunit S10b
Sorghum	Sb04g006830	26S protease regulatory subunit S10B
Maize	GRMZM2G165817	26S protease regulatory subunit S10B
Brachypodium	Bradi1g36400	26S protease regulatory subunit S10B
	Bradi3g07370	26S protease regulatory subunit S10B

Species	Orthologous genes	Putative function
Rice	LOC_Os01g52490	40S ribosomal protein S24, putative, expressed
	LOC_Os02g13530	40S ribosomal protein S24, putative, expressed
	LOC_Os06g36160	40S ribosomal protein S24, putative, expressed
Arabidopsis	AT3G04920	Ribosomal protein S24e family protein
	AT5G28060	Ribosomal protein S24e family protein
Poplar	POPTR_0005s05120	40S ribosomal protein S24 (RPS24B)
	POPTR_0008s15190	40S ribosomal protein S24 (RPS24B)
	POPTR_0010s09820	40S ribosomal protein S24 (RPS24B)
	POPTR_0030s00470	40S ribosomal protein S24 (RPS24B)
Grapevine	GSVIVG00001167001	40S ribosomal protein S24
Sorghum	Sb03g033230	40S ribosomal protein S24
	Sb04g008130	40S ribosomal protein S24
	Sb10g021950	40S ribosomal protein S24
Maize	GRMZM2G029685	40S ribosomal protein S24

	GRMZM2G091383	40S ribosomal protein S24
	GRMZM2G171426	40S ribosomal protein S24
Brachypodium	Bradi1g38010	40S ribosomal protein S24
	Bradi3g08680	40S ribosomal protein S24

**Species**	**Orthologous genes**	**Putative function**
Rice	LOC_Os06g30320	NOC3 - Putative nucleolar complex subunit 3, expressed
Arabidopsis	AT1G79150	binding
Poplar	POPTR_0005s09970	binding
Grapevine	GSVIVG00036806001	Nucleolar complex-associated protein
Sorghum	Sb01g032360	Nucleolar complex-associated protein
Maize	GRMZM2G104983	Nucleolar complex-associated protein
Brachypodium	Bradi1g59970	Nucleolar complex-associated protein

**Species**	**Orthologous genes**	**Putative function**
Rice	LOC_Os06g17840	expressed protein
Arabidopsis	AT5G47690	binding
Poplar	POPTR_0006s00670	binding
	POPTR_0016s00720	binding
Grapevine	GSVIVG00002731001	Androgen induced inhibitor of proliferation (As3) / pds5
Sorghum	Sb10g010710	Androgen induced inhibitor of proliferation (As3) / pds5
Maize	GRMZM2G010637	Androgen induced inhibitor of proliferation (As3) / pds5
	GRMZM2G368678	Androgen induced inhibitor of proliferation (As3) / pds5
Brachypodium	Bradi1g43440	Androgen induced inhibitor of proliferation (As3) / pds5

**Species**	**Orthologous genes**	**Putative function**
Rice	LOC_Os02g49610	ribosomal protein L7Ae, putative, expressed
	LOC_Os06g16290	ribosomal protein L7Ae, putative, expressed
Arabidopsis	AT5G08180	Ribosomal protein L7Ae/L30e/S12e/Gadd45 family protein
Poplar	POPTR_0008s10960	ribosomal protein L7Ae/L30e/S12e/Gadd45 family protein
Grapevine	GSVIVG00008187001	H/ACA ribonucleoprotein complex subunit
Sorghum	Sb04g029160	H/ACA ribonucleoprotein complex subunit 2
	Sb10g010000	H/ACA ribonucleoprotein complex subunit 2
Maize	GRMZM2G122811	H/ACA ribonucleoprotein complex subunit 2
Brachypodium	Bradi3g56240	H/ACA ribonucleoprotein complex subunit 2

**Species**	**Orthologous genes**	**Putative function**
Rice	LOC_Os06g14470	RNA recognition motif containing protein, putative, expressed
Sorghum	Sb10g009240	THO complex subunit 4
Maize	GRMZM2G054468	THO complex subunit 4
Brachypodium	Bradi1g44150	DIP2 protein

Additional File 2 cont.: Orthologous Proteins from Different Plant Species

Species	Orthologous genes	Putative function
Rice	LOC_Os06g12780	OsDegp10 - Putative Deg protease homologue, expressed
Sorghum	Sb10g008370	DegP2 protease
Maize	GRMZM2G165969	DegP2 protease

Species	Orthologous genes	Putative function
Rice	LOC_Os06g10710	expressed protein
Sorghum	Sb10g006920	Conserved gene of unknown function
Maize	GRMZM2G008558	DUF573 domain containing protein
	GRMZM2G066373	DUF573 domain containing protein
Brachypodium	Bradi1g46160	storekeeper protein

Species	Orthologous genes	Putative function
Rice	LOC_Os03g29750	expressed protein
	LOC_Os06g10430	protein of unknown function DUF1296 domain containing protein, expressed
Arabidopsis	AT3G13990	Kinase-related protein of unknown function (DUF1296)
Poplar	POPTR_0001s17070	unknown protein
	POPTR_0003s06200	unknown protein
Grapevine	GSVIVG00016781001	Gb protein
Sorghum	Sb10g006740	Gb protein
Maize	GRMZM2G004736	Hydroxyproline-rich glycoprotein
	GRMZM2G033130	Gb protein
	GRMZM2G152768	Hydroxyproline-rich glycoprotein
Brachypodium	Bradi1g46360	Gb protein
	Bradi1g60080	Gb protein

Species	Orthologous genes	Putative function
Rice	LOC_Os06g09570	nucleolar GTP-binding protein 1, putative, expressed
	LOC_Os07g01920	nucleolar GTP-binding protein 1, putative, expressed
Arabidopsis	AT1G50920	Nucleolar GTP-binding protein
Poplar	POPTR_0001s26610	GTP-binding protein-related
	POPTR_0009s05870	GTP-binding protein-related
Grapevine	GSVIVG00034588001	GTP binding protein
Sorghum	Sb10g006410	GTP binding protein
Maize	GRMZM2G013318	GTP binding protein
	GRMZM2G156986	nucleolar GTP-binding protein
Brachypodium	Bradi1g46650	GTP binding protein

Species	Orthologous genes	Putative function
Rice	LOC_Os06g08770	ruvB-like 2, putative, expressed
Arabidopsis	AT5G67630	P-loop containing nucleoside triphosphate hydrolases superfamily protein
Poplar	POPTR_0006s12040	DNA helicase, putative
	POPTR_0016s10950	DNA helicase, putative

Additional File 2 cont.: Orthologous Proteins from Different Plant Species

Sorghum	Sb02g037530	RuvB-like 2
Maize	AC194970.5_FG009	RuvB-like 2
Brachypodium	Bradi1g23490	RuvB-like 2

Species	Orthologous genes	Putative function
Rice	LOC_Os02g56014	40S ribosomal protein S30, putative, expressed
	LOC_Os06g07580	expressed protein
Arabidopsis	AT2G19750	Ribosomal protein S30 family protein
	AT4G29390	Ribosomal protein S30 family protein
	AT5G56670	Ribosomal protein S30 family protein
Poplar	POPTR_0012s08850	40S ribosomal protein S30 (RPS30B)
	POPTR_0015s09620	40S ribosomal protein S30 (RPS30C)
Grapevine	GSVIVG0001690200 1	40S ribosomal protein S30
Sorghum	Sb04g036360	40S ribosomal protein S30
	Sb10g004940	40S ribosomal protein S30
Maize	GRMZM2G096690	40S ribosomal protein S30
	GRMZM5G805526	40S ribosomal protein S30
Brachypodium	Bradi1g48060	40S ribosomal protein S30

Species	Orthologous genes	Putative function
Rice	LOC_Os06g06880	Ser/Thr protein phosphatase family protein, putative, expressed
Arabidopsis	AT1G64040	type one serine/threonine protein phosphatase 3
	AT4G11240	Calcineurin-like metallo-phosphoesterase superfamily protein
Poplar	POPTR_0001s09930	TOPP7; protein serine/threonine phosphatase
Grapevine	GSVIVG0001942500 1	Serine/threonine-protein phosphatase
Sorghum	Sb10g004490	Serine/threonine-protein phosphatase
Maize	GRMZM2G016930	Serine/threonine-protein phosphatase
	GRMZM2G041822	Serine/threonine-protein phosphatase
Brachypodium	Bradi1g48410	Serine/threonine-protein phosphatase

Species	Orthologous genes	Putative function
Rice	LOC_Os06g06480	Core histone H2A/H2B/H3/H4 domain containing protein, putative, expressed
Brachypodium	Bradi5g09840	Histone H3

Species	Orthologous genes	Putative function
Rice	LOC_Os01g64640	histone H3, putative, expressed
	LOC_Os04g34240	histone H3, putative, expressed
	LOC_Os05g36280	histone H3, putative, expressed
	LOC_Os06g06460	histone H3, putative, expressed
	LOC_Os06g06510	histone H3, putative, expressed
	LOC_Os11g05730	histone H3, putative, expressed
Arabidopsis	AT1G09200	Histone superfamily protein
	AT3G27360	Histone superfamily protein

Additional File 2 cont.: Orthologous Proteins from Different Plant Species

	AT5G10390	Histone superfamily protein
	AT5G10400	Histone superfamily protein
	AT5G65360	Histone superfamily protein
Poplar	POPTR_0001s05450	histone H3
	POPTR_0001s05470	histone H3
	POPTR_0002s03030	histone H3
	POPTR_0003s22120	histone H3
	POPTR_0003s22240	histone H3
	POPTR_0005s25530	histone H3
	POPTR_0014s09260	histone H3
Sorghum	Sb03g005550	Histone H3.2
	Sb04g022160	Histone H3.2
	Sb06g016330	Histone H3.2
	Sb06g016850	Histone H3.2
	Sb09g021650	Histone H3.2
	Sb10g004100	Histone H3.2
	Sb10g004110	Histone H3.2
Maize	GRMZM2G130079	Histone H3.2
	GRMZM2G179005	Histone H3.2
	GRMZM2G355773	Histone H3.2
	GRMZM2G376957	Histone H3.2
	GRMZM2G401581	Histone H3.2
	GRMZM2G418258	Histone H3.2
	GRMZM2G447984	histone cluster 1, H3f
	GRMZM2G451254	Histone H3.2
	GRMZM2G475899	Histone H3
	GRMZM5G864735	Histone H3.2
Brachypodium	Bradi1g48660	Histone H3.2
	Bradi1g50820	Histone H3.2
	Bradi2g18410	Histone H3.2
	Bradi2g24080	Histone H3.2
	Bradi2g27720	Histone H3.2
	Bradi3g45290	Histone H3.2
	Bradi4g07840	Histone H3.2

**Species**	**Orthologous genes**	**Putative function**
Rice	LOC_Os05g46230	NAP domain containing protein, putative, expressed
	LOC_Os06g05660	NAP domain containing protein, putative, expressed
Arabidopsis	AT2G19480	nucleosome assembly protein 1;2
Poplar	POPTR_0006s15090	NAP1;3 (NUCLEOSOME ASSEMBLY PROTEIN 1;3); DNA binding
	POPTR_0018s06890	NAP1;2 (NUCLEOSOME ASSEMBLY PROTEIN 1;2); DNA binding / binding
Grapevine	GSVIVG00016870001	Nucleosome assembly protein
Sorghum	Sb09g026910	Nucleosome/chromatin assembly factor group A
	Sb10g003450	Nucleosome/chromatin assembly factor group A

Additional File 2 cont.: Orthologous Proteins from Different Plant Species

Maize	GRMZM2G140051	Nucleosome/chromatin assembly factor group A
	GRMZM2G176707	Nucleosome/chromatin assembly factor group A
Brachypodium	Bradi1g50860	Nucleosome assembly protein 1 1
	Bradi2g18330	Nucleosome assembly protein 1 2

**Species**	**Orthologous genes**	**Putative function**
Rice	LOC_Os06g05350	whirly transcription factor domain containing protein, expressed
Arabidopsis	AT1G14410	ssDNA-binding transcriptional regulator
	AT2G02740	ssDNA-binding transcriptional regulator
Poplar	POPTR_0008s14840	WHY1 (WHIRLY 1); DNA binding / telomeric DNA binding
	POPTR_0010s10260	WHY1 (WHIRLY 1); DNA binding / telomeric DNA binding
Grapevine	GSVIVG0002006100 1	DNA-binding protein p24
Sorghum	Sb10g003170	DNA-binding protein p24
Maize	GRMZM2G155662	DNA-binding protein p24
Brachypodium	Bradi1g51040	DNA-binding protein p24

**Species**	**Orthologous genes**	**Putative function**
Rice	LOC_Os06g03780	NUC153 domain containing protein, expressed
Arabidopsis	AT3G56990	embryo sac development arrest 7
Poplar	POPTR_0016s03690	EDA7 (embryo sac development arrest 7)
Grapevine	GSVIVG0003361500 1	glycine-rich protein
Sorghum	Sb10g001730	Nucleolar protein 10
Maize	GRMZM2G087196	Nucleolar protein 10
	GRMZM2G092797	Nucleolar protein 10
Brachypodium	Bradi1g49660	glycine-rich protein

**Species**	**Orthologous genes**	**Putative function**
Rice	LOC_Os03g37970	ribosomal protein L13, putative, expressed
	LOC_Os06g02510	ribosomal protein L13, putative, expressed
Arabidopsis	AT3G49010	breast basic conserved 1
	AT5G23900	Ribosomal protein L13e family protein
Poplar	POPTR_0001s01350	ATBBC1 (ARABIDOPSIS THALIANA BREAST BASIC CONSERVED 1); structural constituent of ribosome
	POPTR_0003s10180	ATBBC1 (ARABIDOPSIS THALIANA BREAST BASIC CONSERVED 1); structural constituent of ribosome
	POPTR_0013s02860	ATBBC1 (ARABIDOPSIS THALIANA BREAST BASIC CONSERVED 1); structural constituent of ribosome
	POPTR_0016s08310	ATBBC1 (ARABIDOPSIS THALIANA BREAST BASIC CONSERVED 1); structural constituent of ribosome
Grapevine	GSVIVG0002721200 1	60S ribosomal protein L13
	GSVIVG0003076700 1	60S ribosomal protein L13
Sorghum	Sb03g044290	60S ribosomal protein L13
	Sb09g005460	60S ribosomal protein L13
	Sb09g028960	60S ribosomal protein L13
Maize	GRMZM2G145280	60S ribosomal protein L13
	GRMZM2G409407	60S ribosomal protein L13

Additional File 2 cont.: Orthologous Proteins from Different Plant Species

Brachypodium	Bradi3g01600	60S ribosomal protein L13
	Bradi3g16170	60S ribosomal protein L13

Species	Orthologous genes	Putative function
Rice	LOC_Os06g01700	CWC15 homolog A, putative, expressed
Arabidopsis	AT3G13200	Cwf15 / Cwc15 cell cycle control family protein
Poplar	POPTR_0001s37930	EMB2769 (EMBRYO DEFECTIVE 2769)
Grapevine	GSVIVG0001473500 1	Pre-mRNA-splicing factor cwc15
Sorghum	Sb03g001570	Pre-mRNA-splicing factor cwc15
	Sb10g000650	Pre-mRNA-splicing factor cwc15
Maize	GRMZM2G062076	Pre-mRNA-splicing factor cwc15
	GRMZM2G154278	Pre-mRNA-splicing factor cwc15
Brachypodium	Bradi1g52090	Pre-mRNA-splicing factor cwc15

Species	Orthologous genes	Putative function
Rice	LOC_Os05g51850	AT hook-containing DNA-binding protein, putative, expressed
Sorghum	Sb10g006940	DNA-binding protein
Maize	GRMZM2G008456	DNA-binding protein
Brachypodium	Bradi2g14100	AT hook-containing DNA-binding proteins

Species	Orthologous genes	Putative function
Rice	LOC_Os05g51830	ZOS5-12 - C2H2 zinc finger protein, expressed
Arabidopsis	AT5G03740	histone deacetylase 2C
Poplar	POPTR_0006s11770	HDA3 (HISTONE DEACETYLASE 3); histone deacetylase/ nucleic acid binding / zinc ion binding
Grapevine	GSVIVG0003140400 1	Histone deacetylase 2a
Sorghum	Sb09g026730	Histone deacetylase HDT2
	Sb09g030950	Histone deacetylase HDT2
Maize	GRMZM2G100146	Histone deacetylase HDT2
Brachypodium	Bradi2g14120	Histone deacetylase HDAC2

Species	Orthologous genes	Putative function
Rice	LOC_Os05g51180	hyaluronan/mRNA binding family domain containing protein, expressed
Sorghum	Sb09g030400	Plasminogen activator inhibitor 1 RNA-binding protein
Maize	GRMZM2G464401	Plasminogen activator inhibitor 1 RNA-binding protein
Brachypodium	Bradi2g14540	Plasminogen activator inhibitor 1 RNA-binding protein

Species	Orthologous genes	Putative function
Rice	LOC_Os05g49890	ras-related protein, putative, expressed
Arabidopsis	AT5G20010	RAS-related nuclear protein-1
	AT5G20020	RAS-related GTP-binding nuclear protein 2
	AT5G55190	RAN GTPase 3
Poplar	POPTR_0006s26650	RAN3 (RAN GTPASE 3); GTP binding / GTPase/ protein binding
	POPTR_0006s26660	RAN3 (RAN GTPASE 3); GTP binding / GTPase/ protein binding

Additional File 2 cont.: Orthologous Proteins from Different Plant Species

	POPTR_0018s01400	RAN3 (RAN GTPASE 3); GTP binding / GTPase/ protein binding
	POPTR_0018s01420	RAN3 (RAN GTPASE 3); GTP binding / GTPase/ protein binding
Grapevine	GSVIVG00015482001	GTP-binding protein
	GSVIVG00017234001	GTP-binding protein
	GSVIVG00035804001	GTP-binding protein
	GSVIVG00035805001	GTP-binding nuclear protein Ran-3
Sorghum	Sb03g029530	GTP-binding nuclear protein Ran-A1
	Sb09g029250	GTP-binding nuclear protein Ran-A1
Maize	GRMZM2G157334	GTP-binding nuclear protein Ran-A1
	GRMZM2G354604	GTP-binding nuclear protein Ran-A1
Brachypodium	Bradi2g15730	GTP-binding nuclear protein Ran-2

**Species**	**Orthologous genes**	**Putative function**
Rice	LOC_Os01g05610	Core histone H2A/H2B/H3/H4 domain containing protein, putative, expressed
	LOC_Os01g05630	Core histone H2A/H2B/H3/H4 domain containing protein, putative, expressed
	LOC_Os01g05900	Core histone H2A/H2B/H3/H4 domain containing protein, putative
	LOC_Os01g05970	OsFBO1 - F-box and other domain containing protein, expressed
	LOC_Os01g06010	Core histone H2A/H2B/H3/H4 domain containing protein, putative, expressed
	LOC_Os01g62230	Core histone H2A/H2B/H3/H4 domain containing protein, putative, expressed
	LOC_Os05g49860	Core histone H2A/H2B/H3/H4 domain containing protein, putative, expressed
	LOC_Os08g38300	Core histone H2A/H2B/H3/H4 domain containing protein, putative, expressed
Arabidopsis	AT1G07790	Histone superfamily protein
	AT2G28720	Histone superfamily protein
	AT3G45980	Histone superfamily protein
	AT3G46030	Histone superfamily protein
	AT5G22880	histone B2
	AT5G59910	Histone superfamily protein
Poplar	POPTR_0008s02990	HTB1; DNA binding
	POPTR_0008s03040	histone H2B, putative
	POPTR_0008s03050	histone H2B, putative
	POPTR_0008s03060	HTB9; DNA binding
	POPTR_0010s23720	histone H2B, putative
	POPTR_0010s23730	histone H2B, putative
	POPTR_0010s23770	HTB9; DNA binding
Grapevine	GSVIVG00025021001	Histone H2B
	GSVIVG00025023001	Histone H2B
	GSVIVG00025025001	Histone H2B
Sorghum	Sb02g025410	Histone H2B
	Sb02g041800	Histone H2B
	Sb03g005720	Histone H2B
	Sb03g005730	Histone H2B
	Sb03g007700	Histone H2B

Additional File 2 cont.: Orthologous Proteins from Different Plant Species

	Sb03g026260	Histone H2B
	Sb03g039310	Histone H2B
	Sb04g030340	Histone H2B
	Sb07g022370	Histone H2B
	Sb07g028760	Histone H2B
	Sb09g022610	Histone H2B
Maize	GRMZM2G071959	Histone H2B.1
	GRMZM2G112912	Histone H2B
	GRMZM2G119071	Histone H2B.2
	GRMZM2G141432	Histone H2B
	GRMZM2G163939	Histone H2B
	GRMZM2G304575	Histone H2B
	GRMZM2G306258	Histone H2B.4
	GRMZM2G342515	Histone H2B.5
	GRMZM2G401147	Histone H2B
	GRMZM2G472696	Histone H2B
Brachypodium	Bradi1g08860	Histone H2B.1
	Bradi1g47980	Histone H2B
	Bradi1g56060	Histone H2B.1
	Bradi2g00510	Histone H2B
	Bradi2g00530	Histone H2B
	Bradi2g23230	Histone H2B
	Bradi2g27710	Histone H2B.1
	Bradi2g27760	Histone H2B
	Bradi2g54540	Histone H2B.11
	Bradi3g54520	Histone H2B

**Species**	**Orthologous genes**	**Putative function**
Rice	LOC_Os05g49230	ribosomal RNA large subunit methyltransferase J, putative, expressed
Arabidopsis	AT4G25730	FtsJ-like methyltransferase family protein
Poplar	POPTR_0009s12100	FtsJ-like methyltransferase family protein
Grapevine	GSVIVG00023801001	Ribosomal RNA methyltransferase
Sorghum	Sb09g028780	FtsJ-like methyltransferase family protein
Maize	GRMZM2G128579	FtsJ-like methyltransferase family protein
	GRMZM2G158091	Ribosomal RNA methyltransferase
Brachypodium	Bradi2g16130	FtsJ-like methyltransferase family protein

**Species**	**Orthologous genes**	**Putative function**
Rice	LOC_Os01g47660	60S ribosomal protein L18a, putative, expressed
	LOC_Os05g49030	60S ribosomal protein L18a, putative, expressed
Arabidopsis	AT2G34480	Ribosomal protein L18ae/LX family protein
Poplar	POPTR_0002s05870	60S ribosomal protein L18A (RPL18aB)
	POPTR_0011s06730	60S ribosomal protein L18A (RPL18aB)

Additional File 2 cont.: Orthologous Proteins from Different Plant Species

	POPTR_1064s00200	60S ribosomal protein L18A (RPL18aB)
Grapevine	GSVIVG00000485001	60S ribosomal protein L18a
	GSVIVG00021430001	similar to RPL18AA (60S RIBOSOMAL PROTEIN L18A-1)
Sorghum	Sb03g030600	60S ribosomal protein L18a
	Sb05g007540	60S ribosomal protein L18a
	Sb09g028590	60S ribosomal protein L18a
Maize	AC230013.2_FG007	60S ribosomal protein L18a
	GRMZM2G113720	60S ribosomal protein L18a
Brachypodium	Bradi2g16430	60S ribosomal protein L18a
	Bradi2g46010	60S ribosomal protein L18a

Species	Orthologous genes	Putative function
Rice	LOC_Os01g48180	DDT domain containing protein, putative, expressed
	LOC_Os05g48820	DDT, putative, expressed
Arabidopsis	AT1G28420	homeobox-1
	AT5G44180	Homeodomain-like transcriptional regulator
Poplar	POPTR_0004s04840	HB-1 (homeobox-1); transcription factor
	POPTR_0007s01330	homeobox transcription factor, putative
	POPTR_0011s05660	HB-1 (homeobox-1); transcription factor
	POPTR_0017s04760	homeobox transcription factor, putative
Grapevine	GSVIVG00020605001	Homeobox protein
	GSVIVG00021113001	Homeobox protein
Sorghum	Sb03g030770	Homeobox protein
	Sb03g030785	Homeobox protein
Maize	AC196465.3_FG006	Homeobox protein
	GRMZM2G122750	Homeobox protein
	GRMZM2G125294	Homeobox protein
	GRMZM2G159357	Homeobox protein
Brachypodium	Bradi2g16650	Homeobox protein
	Bradi2g46230	Homeobox protein

Species	Orthologous genes	Putative function
Rice	LOC_Os05g44320	WD domain, G-beta repeat domain containing protein, expressed
Arabidopsis	AT1G15440	periodic tryptophan protein 2
Poplar	POPTR_0001s17340	transducin family protein / WD-40 repeat family protein
Grapevine	GSVIVG00017243001	WD-repeat protein
Sorghum	Sb09g025880	WD-repeat protein
Maize	GRMZM2G060817	JHL25H03.15 protein
Brachypodium	Bradi2g19560	Periodic tryptophan protein-associated region; WD40

Species	Orthologous genes	Putative function
Rice	LOC_Os05g41172	histone-lysine N-methyltransferase, H3 lysine-9 specific SUVH1, putative, expressed

Additional File 2 cont.: Orthologous Proteins from Different Plant Species

Arabidopsis	AT1G73100	SU(VAR)3-9 homolog 3
Sorghum	Sb09g024010	Histone-lysine N-methyltransferase, H3 lysine-9 specific SUVH1
Maize	GRMZM2G117458	Histone-lysine N-methyltransferase, H3 lysine-9 specific SUVH1
	GRMZM2G140577	SET domain protein 105
Brachypodium	Bradi4g25940	Histone-lysine N-methyltransferase, H3 lysine-9 specific SUVH1

Species	Orthologous genes	Putative function
Rice	LOC_Os01g59990	ribosomal protein L24, putative, expressed
	LOC_Os05g40820	ribosomal protein L24, putative, expressed
	LOC_Os07g12250	ribosomal protein L24, putative, expressed
Arabidopsis	AT2G36620	ribosomal protein L24
	AT3G53020	Ribosomal protein L24e family protein
Poplar	POPTR_0003s12330	RPL24A (ribosomal protein L24); structural constituent of ribosome
	POPTR_0004s08370	RPL24A (ribosomal protein L24); structural constituent of ribosome
	POPTR_0012s13380	RPL24A (ribosomal protein L24); structural constituent of ribosome
	POPTR_0015s13330	RPL24A (ribosomal protein L24); structural constituent of ribosome
Grapevine	GSVIVG00010601001	60S ribosomal protein L24
	GSVIVG00033743001	60S ribosomal protein L24
	GSVIVG00034676001	60S ribosomal protein L24
Sorghum	Sb01g015240	60S ribosomal protein L24
	Sb09g023800	60S ribosomal protein L24
Maize	GRMZM2G074898	60S ribosomal protein L24
	GRMZM2G110328	60S ribosomal protein L24
	GRMZM2G142640	60S ribosomal protein L24
Brachypodium	Bradi2g53220	60S ribosomal protein L24

Species	Orthologous genes	Putative function
Rice	LOC_Os05g02300	Core histone H2A/H2B/H3/H4 domain containing protein, putative, expressed
	LOC_Os05g38640	Core histone H2A/H2B/H3/H4 domain containing protein, putative, expressed
Arabidopsis	AT5G02560	histone H2A 12
Poplar	POPTR_0006s08230	HTA12; DNA binding
Grapevine	GSVIVG00025019001	Histone H2A
Sorghum	Sb01g039240	Histone H2A
	Sb01g039250	Histone H2A
	Sb02g030950	Histone H2A
	Sb09g022690	Histone H2A
Maize	GRMZM2G003306	Histone H2A
	GRMZM2G047813	Histone H2A
	GRMZM2G109448	Histone H2A
	GRMZM2G130746	Histone H2A
	GRMZM2G448458	Histone H2A
Brachypodium	Bradi1g66370	Histone H2A

	Bradi2g23090	histone H2A.4

**Species**	**Orthologous genes**	**Putative function**
Rice	LOC_Os05g30880	nucleolar matrix protein-related, putative, expressed
Arabidopsis	AT5G05210	Surfeit locus protein 6
Poplar	POPTR_0008s17340	nucleolar matrix protein-related
	POPTR_0010s07460	nucleolar matrix protein-related
Grapevine	GSVIVG00027946001	Caldesmon
Sorghum	Sb04g026820	Caldesmon
Maize	GRMZM2G141411	Caldesmon
Brachypodium	Bradi1g10600	Caldesmon
	Bradi3g49500	Caldesmon

**Species**	**Orthologous genes**	**Putative function**
Rice	LOC_Os05g30530	40S ribosomal protein S4, putative, expressed
Sorghum	Sb09g018320	40S ribosomal protein S4

**Species**	**Orthologous genes**	**Putative function**
Rice	LOC_Os05g28280	peptidase, M24 family protein, putative, expressed
Arabidopsis	AT3G51800	metallopeptidase M24 family protein
Poplar	POPTR_0006s10320	ATG2; aminopeptidase/ metalloexopeptidase
	POPTR_0016s12720	ATG2; aminopeptidase/ metalloexopeptidase
Grapevine	GSVIVG00003934001	Proliferation-associated 2g4
Sorghum	Sb09g016610	Proliferation-associated protein 2G4
Maize	AC233895.1_FG001	Proliferation-associated protein 2G4
Brachypodium	Bradi2g28680	Proliferation-associated protein 2G4

**Species**	**Orthologous genes**	**Putative function**
Rice	LOC_Os03g18570	40S ribosomal protein S7, putative, expressed
	LOC_Os03g18580	40S ribosomal protein S7, putative
	LOC_Os05g27940	40S ribosomal protein S7, putative, expressed
Arabidopsis	AT1G48830	Ribosomal protein S7e family protein
	AT3G02560	Ribosomal protein S7e family protein
	AT5G16130	Ribosomal protein S7e family protein
Poplar	POPTR_0004s09830	40S ribosomal protein S7 (RPS7B)
	POPTR_0005s15580	40S ribosomal protein S7 (RPS7B)
	POPTR_0006s08820	40S ribosomal protein S7 (RPS7B)
	POPTR_0016s10560	40S ribosomal protein S7 (RPS7B)
Grapevine	GSVIVG00008307001	40S ribosomal protein S7
	GSVIVG00031018001	40S ribosomal protein S7
Sorghum	Sb01g038170	40S ribosomal protein S7
	Sb09g016170	40S ribosomal protein S7

Additional File 2 cont.: Orthologous Proteins from Different Plant Species

Maize	GRMZM2G030016	40S ribosomal protein S7
	GRMZM2G053652	40S ribosomal protein S7
Brachypodium	Bradi1g65150	Ribosomal protein S7

**Species**	**Orthologous genes**	**Putative function**
Rice	LOC_Os05g22920	digestive organ expansion factor, putative, expressed
Arabidopsis	AT1G17690	NA
Poplar	POPTR_0022s00850	unknown protein
Grapevine	GSVIVG00010908001	conserved hypothetical protein
Sorghum	Sb08g002830	Digestive organ expansion factor homolog
Maize	GRMZM2G003732	LOC100037097 protein
Brachypodium	Bradi2g05440	conserved hypothetical protein

**Species**	**Orthologous genes**	**Putative function**
Rice	LOC_Os05g16660	WD domain, G-beta repeat domain containing protein, expressed
	LOC_Os08g21660	WD domain, G-beta repeat domain containing protein, expressed
Arabidopsis	AT2G46280	TGF-beta receptor interacting protein 1
	AT2G46290	Transducin/WD40 repeat-like superfamily protein
Poplar	POPTR_0008s14090	eukaryotic translation initiation factor 3 subunit 2, putative / eIF-3 beta, putative / eIF3i, putative
	POPTR_0010s11040	eukaryotic translation initiation factor 3 subunit 2, putative / eIF-3 beta, putative / eIF3i, putative
Grapevine	GSVIVG00011562001	TGF-beta receptor-interacting protein 1
Sorghum	Sb02g003760	Eukaryotic translation initiation factor 3 subunit 2
Maize	GRMZM2G028834	Eukaryotic translation initiation factor 3 subunit 2
	GRMZM2G143330	Eukaryotic translation initiation factor 3 subunit 2
Brachypodium	Bradi4g14100	Eukaryotic translation initiation factor 3 subunit 2

**Species**	**Orthologous genes**	**Putative function**
Rice	LOC_Os01g10820	ribosomal protein L5, putative, expressed
	LOC_Os02g14059	ribosomal protein L5, putative, expressed
	LOC_Os05g11710	ribosomal protein L5, putative, expressed
	LOC_Os06g35730	ribosomal protein L5, putative, expressed
Arabidopsis	AT2G42740	ribosomal protein large subunit 16A
	AT3G58700	Ribosomal L5P family protein
	AT4G18730	ribosomal protein L16B
	AT5G45775	Ribosomal L5P family protein
Poplar	POPTR_0006s19530	60S ribosomal protein L11 (RPL11B)
	POPTR_0006s19540	60S ribosomal protein L11 (RPL11B)
	POPTR_0011s07080	60S ribosomal protein L11 (RPL11B)
	POPTR_0011s07100	60S ribosomal protein L11 (RPL11B)
Grapevine	GSVIVG00020743001	60S ribosomal protein L11
	GSVIVG00021244001	60S ribosomal protein L11-1
Sorghum	Sb03g002420	60S ribosomal protein L11

Additional File 2 cont.: Orthologous Proteins from Different Plant Species

	Sb04g008530	60S ribosomal protein L11-1
	Sb04g008540	60S ribosomal protein L11-1
Maize	GRMZM2G001816	60S ribosomal protein L11-1
	GRMZM2G014444	60S ribosomal protein L11-1
	GRMZM2G018770	60S ribosomal protein L11-1
	GRMZM5G813584	60S ribosomal protein L11-1
Brachypodium	Bradi2g06470	Ribosomal protein L11
	Bradi2g32640	Ribosomal protein L11
	Bradi3g09030	Ribosomal protein L11

**Species**	**Orthologous genes**	**Putative function**
Rice	LOC_Os05g09620	SCC3, putative, expressed
Arabidopsis	AT2G47980	sister-chromatid cohesion protein 3
Poplar	POPTR_0002s23150	SCC3 (SISTER-CHROMATID COHESION PROTEIN 3); binding
	POPTR_0014s14940	SCC3 (SISTER-CHROMATID COHESION PROTEIN 3); binding
Grapevine	GSVIVG00011020001	Stromal antigen
Sorghum	Sb05g025690	SCC3
Maize	GRMZM2G131443	SCC3
Brachypodium	Bradi4g11510	SCC3

**Species**	**Orthologous genes**	**Putative function**
Rice	LOC_Os02g57590	rRNA 2-O-methyltransferase fibrillarin 2, putative, expressed
	LOC_Os05g08360	rRNA 2-O-methyltransferase fibrillarin 2, putative, expressed
Arabidopsis	AT4G25630	fibrillarin 2
	AT5G52470	fibrillarin 1
Poplar	POPTR_0012s12860	FIB2 (FIBRILLARIN 2); snoRNA binding
	POPTR_0015s12790	FIB1 (FIBRILLARIN 1); snoRNA binding
Grapevine	GSVIVG00028887001	fibrillarin homolog
Sorghum	Sb04g037640	Fibrillarin-2
Maize	GRMZM2G363678	Fibrillarin-2
Brachypodium	Bradi1g32010	Fibrillarin-2
	Bradi3g55810	Fibrillarin-2

**Species**	**Orthologous genes**	**Putative function**
Rice	LOC_Os05g07700	ribosomal protein, putative, expressed
	LOC_Os11g11390	ribosomal protein, putative, expressed
Arabidopsis	AT1G14320	Ribosomal protein L16p/L10e family protein
	AT1G26910	Ribosomal protein L16p/L10e family protein
	AT1G66580	senescence associated gene 24
Poplar	POPTR_0013s15560	60S ribosomal protein L10 (RPL10B)
	POPTR_0019s15250	60S ribosomal protein L10 (RPL10B)
Grapevine	GSVIVG00024537001	60S ribosomal protein L10

Additional File 2 cont.: Orthologous Proteins from Different Plant Species

Sorghum	Sb01g015470	60S ribosomal protein L10-3
	Sb04g008500	60S ribosomal protein L10-1
Maize	GRMZM2G087233	60S ribosomal protein L10-3
Brachypodium	Bradi4g22070	60S ribosomal protein L10-3

**Species**	**Orthologous genes**	**Putative function**
Rice	LOC_Os01g22490	40S ribosomal protein S27a, putative, expressed
	LOC_Os05g06770	40S ribosomal protein S27a, putative, expressed
Arabidopsis	AT2G47110	ubiquitin 6
	AT3G62250	ubiquitin 5
Poplar	POPTR_0001s06260	UBQ6; protein binding
	POPTR_0002s19070	UBQ6; protein binding
	POPTR_0012s11550	UBQ6; protein binding
	POPTR_0014s11040	UBQ6; protein binding
	POPTR_0015s12320	UBQ6; protein binding
Sorghum	Sb03g013260	40S ribosomal protein S27a
	Sb09g004630	40S ribosomal protein S27a
Maize	GRMZM2G047732	Ubiquitin-40S ribosomal protein S27a
	GRMZM2G357296	40S ribosomal protein S27a
	GRMZM2G431821	Ubiquitin-40S ribosomal protein S27a
Brachypodium	Bradi2g12280	Ubiquitin
	Bradi2g34750	Ubiquitin

**Species**	**Orthologous genes**	**Putative function**
Rice	LOC_Os01g14950	importin subunit alpha, putative, expressed
	LOC_Os05g06350	importin subunit alpha, putative, expressed
Arabidopsis	AT3G06720	importin alpha isoform 1
	AT4G16143	importin alpha isoform 2
Poplar	POPTR_0005s02030	IMPA-1 (IMPORTIN ALPHA ISOFORM 1); binding / protein transporter
	POPTR_0008s22930	IMPA-1 (IMPORTIN ALPHA ISOFORM 1); binding / protein transporter
	POPTR_0013s01220	IMPA-1 (IMPORTIN ALPHA ISOFORM 1); binding / protein transporter
Grapevine	GSVIVG00031205001	Importin alpha
	GSVIVG00035047001	Impa2
Sorghum	Sb03g009700	Importin alpha-1b subunit
	Sb09g004320	Importin subunit alpha-1b
Maize	GRMZM2G009845	Importin subunit alpha-1b
	GRMZM2G088088	Importin subunit alpha-1b
	GRMZM2G091119	Importin alpha-1b subunit
Brachypodium	Bradi2g08960	Importin subunit alpha-1a
	Bradi2g35050	Importin subunit alpha-1b

**Species**	**Orthologous genes**	**Putative function**
Rice	LOC_Os05g06310	60S ribosomal protein L18-3, putative, expressed

Additional File 2 cont.: Orthologous Proteins from Different Plant Species

Sorghum	Sb09g004290	Ribosomal protein L18
Maize	GRMZM2G104025	Ribosomal protein L18
	GRMZM5G803952	Ribosomal protein L18
Brachypodium	Bradi2g35090	Ribosomal protein L18

**Species**	**Orthologous genes**	**Putative function**
Rice	LOC_Os05g04850	RNA recognition motif containing protein, putative, expressed
Arabidopsis	AT1G51510	RNA-binding (RRM/RBD/RNP motifs) family protein
Poplar	POPTR_0001s26260	Y14; RNA binding / protein binding
	POPTR_0009s05550	Y14; RNA binding / protein binding
Grapevine	GSVIVG00026504001	Polyadenylate-binding protein
Sorghum	Sb09g003130	RNA-binding protein 8A
Maize	GRMZM5G803275	RNA-binding protein 8A
	GRMZM5G817255	RNA-binding protein 8A
Brachypodium	Bradi2g36710	MRNA transport factor

**Species**	**Orthologous genes**	**Putative function**
Rice	LOC_Os05g03740	transcription factor TF2, putative, expressed
Arabidopsis	AT2G38250	Homeodomain-like superfamily protein
	AT5G01380	Homeodomain-like superfamily protein
Sorghum	Sb09g002370	Transcription factor
Maize	GRMZM2G016637	Trihelix transcription factor GT-3a
Brachypodium	Bradi2g38230	Transcription factor GT-3a

**Species**	**Orthologous genes**	**Putative function**
Rice	LOC_Os05g02300	Core histone H2A/H2B/H3/H4 domain containing protein, putative, expressed
	LOC_Os05g38640	Core histone H2A/H2B/H3/H4 domain containing protein, putative, expressed
Arabidopsis	AT5G02560	histone H2A 12
Poplar	POPTR_0006s08230	HTA12; DNA binding
Grapevine	GSVIVG00025019001	Histone H2A
Sorghum	Sb01g039240	Histone H2A
	Sb01g039250	Histone H2A
	Sb02g030950	Histone H2A
	Sb09g022690	Histone H2A
Maize	GRMZM2G003306	Histone H2A
	GRMZM2G047813	Histone H2A
	GRMZM2G109448	Histone H2A
	GRMZM2G130746	Histone H2A
	GRMZM2G448458	Histone H2A
Brachypodium	Bradi1g66370	Histone H2A
	Bradi2g23090	histone H2A.4

**Species**	**Orthologous genes**	**Putative function**

Additional File 2 cont.: Orthologous Proteins from Different Plant Species

Rice	LOC_Os05g01450	eukaryotic translation initiation factor 3 subunit F, putative, expressed
Arabidopsis	AT2G39990	eukaryotic translation initiation factor 2
Poplar	POPTR_0010s19940	EIF2; translation initiation factor
Grapevine	GSVIVG00016359001	Eukaryotic translation initiation factor 3f, eif3f
Sorghum	Sb09g026780	Eukaryotic translation initiation factor 3 subunit 5
Maize	GRMZM2G098153	Eukaryotic translation initiation factor 3 subunit 5
Brachypodium	Bradi2g39810	Eukaryotic translation initiation factor 3 subunit 5

Species	Orthologous genes	Putative function
Rice	LOC_Os04g58830	ribosome biogenesis regulatory protein, putative, expressed
Arabidopsis	AT2G37990	ribosome biogenesis regulatory protein (RRS1) family protein
Poplar	POPTR_0005s25260	ribosome biogenesis regulatory protein (RRS1) family protein
Grapevine	GSVIVG00027072001	Ribosome biogenesis regulatory protein
	GSVIVG00027422001	Ribosome biogenesis regulatory protein
Sorghum	Sb03g003790	Ribosome biogenesis regulatory protein
Maize	GRMZM2G127312	Ribosome biogenesis regulatory protein
	GRMZM2G132519	Ribosome biogenesis regulatory protein
Brachypodium	Bradi2g37930	Ribosome biogenesis regulatory protein

Species	Orthologous genes	Putative function
Rice	LOC_Os04g57010	zinc finger C-x8-C-x5-C-x3-H type family protein, expressed
Sorghum	Sb06g032000	Zinc finger C-x8-C-x5-C-x3-H type family protein
Maize	GRMZM2G071034	Zinc finger C-x8-C-x5-C-x3-H type family protein
Brachypodium	Bradi5g25340	Zinc finger CCCH domain-containing protein 31

Species	Orthologous genes	Putative function
Rice	LOC_Os04g56720	RCC2, putative, expressed
Arabidopsis	AT1G19880	Regulator of chromosome condensation (RCC1) family protein
Poplar	POPTR_0002s02810	regulator of chromosome condensation (RCC1) family protein
	POPTR_0005s25730	regulator of chromosome condensation (RCC1) family protein
Grapevine	GSVIVG00009847001	Protein RCC2
Sorghum	Sb06g031820	Protein RCC2
Maize	GRMZM5G866734	Protein RCC2
Brachypodium	Bradi5g25080	Protein RCC2

Species	Orthologous genes	Putative function
Rice	LOC_Os02g31960	ATP/GTP binding protein, putative, expressed
	LOC_Os04g56590	ATP/GTP binding protein, putative, expressed
Arabidopsis	AT5G10010	NA
Poplar	POPTR_0007s06500	unknown protein
Grapevine	GSVIVG00022216001	117M18_4
Sorghum	Sb01g018160	ATP/GTP binding protein

	Sb06g020900	ATP/GTP binding protein
Maize	GRMZM2G021219	ATP/GTP binding protein
Brachypodium	Bradi5g24900	ATP/GTP binding protein

**Species**	**Orthologous genes**	**Putative function**
Rice	LOC_Os02g26400	nuclease, EndA/NucM family protein, expressed
	LOC_Os04g56350	nucleolar protein,Nop52 containing protein, expressed
Arabidopsis	AT5G20600	NA
Poplar	POPTR_0012s03330	unknown protein
	POPTR_0012s03340	unknown protein
Grapevine	GSVIVG00029258001	Nnp-1 protein
Sorghum	Sb06g031410	Nucleolar protein,Nop52 containing protein
Maize	GRMZM2G029113	Nucleolar protein,Nop52 containing protein
	GRMZM2G101179	Nucleolar protein,Nop52 containing protein
Brachypodium	Bradi5g24580	H. sapiens NNP-1 / Nop52 AP001752 (Score = 92; E= 9e-18)

**Species**	**Orthologous genes**	**Putative function**
Rice	LOC_Os04g52960	nucleolin, putative, expressed
	LOC_Os08g09350	gar2, putative, expressed
Arabidopsis	AT1G48920	nucleolin like 1
	AT3G18610	nucleolin like 2
Poplar	POPTR_0005s09600	ATRANGAP1 (RAN GTPASE-ACTIVATING PROTEIN 1); nucleic acid binding / nucleotide binding
	POPTR_0007s07800	ATRANGAP1 (RAN GTPASE-ACTIVATING PROTEIN 1); nucleic acid binding / nucleotide binding
Grapevine	GSVIVG00012042001	Nucleic acid binding protein
Sorghum	Sb01g019710	nucleolin
	Sb07g005510	Nucleic acid binding protein
Maize	GRMZM2G001850	NuM1 protein
	GRMZM2G131943	nucleolin
Brachypodium	Bradi3g14910	L. (clone na-481-5)
	Bradi5g22120	Nucleolin

**Species**	**Orthologous genes**	**Putative function**
Rice	LOC_Os04g52200	RNA recognition motif containing protein, putative, expressed
Arabidopsis	AT4G19610	nucleotide binding;nucleic acid binding;RNA binding
Poplar	POPTR_0003s14940	RNA binding / nucleic acid binding / nucleotide binding
Grapevine	GSVIVG00012163001	RNA binding / nucleic acid binding / nucleotide binding
Sorghum	Sb06g028190	RNA binding / nucleic acid binding / nucleotide binding
Maize	GRMZM2G031529	RNA binding / nucleic acid binding / nucleotide binding
Brachypodium	Bradi5g21380	RNA binding / nucleic acid binding / nucleotide binding

**Species**	**Orthologous genes**	**Putative function**
Rice	LOC_Os04g51630	60S ribosomal protein L7, putative, expressed

Additional File 2 cont.: Orthologous Proteins from Different Plant Species

	LOC_Os08g13690	60S ribosomal protein L7, putative, expressed
Arabidopsis	AT2G01250	Ribosomal protein L30/L7 family protein
	AT2G44120	Ribosomal protein L30/L7 family protein
	AT3G13580	Ribosomal protein L30/L7 family protein
Poplar	POPTR_0006s07290	60S ribosomal protein L7 (RPL7C)
	POPTR_0008s00840	60S ribosomal protein L7 (RPL7D)
	POPTR_0010s25740	60S ribosomal protein L7 (RPL7D)
	POPTR_0018s13700	60S ribosomal protein L7 (RPL7D)
Grapevine	GSVIVG00029655001	60S ribosomal protein L7
Sorghum	Sb06g027740	60S ribosomal protein L7-2
	Sb07g006970	60S ribosomal protein L7-1
Maize	GRMZM2G094051	60S ribosomal protein L7-2
	GRMZM2G100225	60S ribosomal protein L7-1
	GRMZM2G178807	60S ribosomal protein L7-1
	GRMZM5G868433	60S ribosomal protein L7-2
Brachypodium	Bradi1g04950	60S ribosomal protein L7-2
	Bradi5g20900	60S ribosomal protein L7-2

**Species**	**Orthologous genes**	**Putative function**
Rice	LOC_Os02g47140	L11 domain containing ribosomal protein, putative, expressed
	LOC_Os04g50990	L11 domain containing ribosomal protein, putative, expressed
Arabidopsis	AT2G37190	Ribosomal protein L11 family protein
	AT3G53430	Ribosomal protein L11 family protein
	AT5G60670	Ribosomal protein L11 family protein
Poplar	POPTR_0006s07680	60S ribosomal protein L12 (RPL12B)
	POPTR_0018s14210	60S ribosomal protein L12 (RPL12B)
	POPTR_0018s14220	60S ribosomal protein L12 (RPL12B)
	POPTR_0516s00210	60S ribosomal protein L12 (RPL12B)
Grapevine	GSVIVG00025683001	60S ribosomal protein L12
Sorghum	Sb04g030890	60S ribosomal protein L12
	Sb06g014530	60S ribosomal protein L12
	Sb06g027330	60S ribosomal protein L12
Maize	AC196489.3_FG002	60S ribosomal protein L12
	GRMZM2G121075	60S ribosomal protein L12
	GRMZM2G149649	60S ribosomal protein L12
Brachypodium	Bradi3g52460	60S ribosomal protein L12
	Bradi5g20330	60S ribosomal protein L12

**Species**	**Orthologous genes**	**Putative function**
Rice	LOC_Os04g50660	WD domain, G-beta repeat domain containing protein, expressed
Arabidopsis	AT5G16750	Transducin family protein / WD-40 repeat family protein
Poplar	POPTR_0013s07710	TOZ (TORMOZEMBRYO DEFECTIVE); nucleotide binding
	POPTR_0019s07170	TOZ (TORMOZEMBRYO DEFECTIVE); nucleotide binding

Additional File 2 cont.: Orthologous Proteins from Different Plant Species

Grapevine	GSVIVG00024351001	U3 small nucleolar RNA-associated protein
Sorghum	Sb06g027070	U3 small nucleolar RNA-associated protein
Maize	GRMZM2G014276	U3 small nucleolar RNA-associated protein
	GRMZM2G128092	U3 small nucleolar RNA-associated protein
Brachypodium	Bradi5g20040	U3 small nucleolar RNA-associated protein

**Species**	**Orthologous genes**	**Putative function**
Rice	LOC_Os04g49580	nucleolar complex protein, putative, expressed
Arabidopsis	AT2G17250	CCAAT-binding factor
Poplar	POPTR_0009s16930	EMB2762 (EMBRYO DEFECTIVE 2762)
Grapevine	GSVIVG00004114001	similar to EMB2762 (EMBRYO DEFECTIVE 2762), partial
	GSVIVG00004442001	similar to EMB2762 (EMBRYO DEFECTIVE 2762), partial
Sorghum	Sb06g026607	Nucleolar complex protein 4
Maize	GRMZM2G031326	Nucleolar complex protein 4
Brachypodium	Bradi5g19570	Nucleolar complex protein 4

**Species**	**Orthologous genes**	**Putative function**
Rice	LOC_Os04g48060	RFC2 - Putative clamp loader of PCNA, replication factor C subunit 2, expressed
Arabidopsis	AT1G63160	replication factor C 2
Poplar	POPTR_0003s12340	replication factor C 40 kDa, putative
Grapevine	GSVIVG00019825001	Replication factor C / DNA polymerase III gamma-tau subunit
Sorghum	Sb06g025740	Replication factor C subunit 4
Maize	AC235534.1_FG001	Replication factor C subunit 4
Brachypodium	Bradi5g18710	Replication factor C 40kDa subunit

**Species**	**Orthologous genes**	**Putative function**
Rice	LOC_Os04g46920	zinc knuckle domain containing protein, expressed
Arabidopsis	AT5G52380	VASCULAR-RELATED NAC-DOMAIN 6
Poplar	POPTR_0012s13145	zinc knuckle (CCHC-type) family protein
	POPTR_0015s13070	zinc knuckle (CCHC-type) family protein
Grapevine	GSVIVG00028962001	Actin depolymerizing factor
Sorghum	Sb06g024880	actin depolymerizing factor
Maize	GRMZM2G071253	Actin depolymerizing factor
Brachypodium	Bradi5g17970	Actin depolymerizing factor

**Species**	**Orthologous genes**	**Putative function**
Rice	LOC_Os04g45940	transcription factor like protein, putative, expressed
Sorghum	Sb06g024110	MADF; Homeodomain
Maize	GRMZM2G320827	MADF; Homeodomain
	GRMZM2G427087	MADF; Homeodomain
Brachypodium	Bradi5g17280	MADF; Homeodomain

Additional File 2 cont.: Orthologous Proteins from Different Plant Species

Species	Orthologous genes	Putative function
Rice	LOC_Os01g24690	60S ribosomal protein L23A, putative, expressed
	LOC_Os04g42270	60S ribosomal protein L23A, putative, expressed
Arabidopsis	AT2G39460	ribosomal protein L23AA
	AT3G55280	ribosomal protein L23AB
Poplar	POPTR_0006s22980	RPL23AB (RIBOSOMAL PROTEIN L23AB); RNA binding / nucleotide binding / structural constituent of ribosome
	POPTR_0008s05000	RPL23AB (RIBOSOMAL PROTEIN L23AB); RNA binding / nucleotide binding / structural constituent of ribosome
	POPTR_0009s03210	RPL23AB (RIBOSOMAL PROTEIN L23AB); RNA binding / nucleotide binding / structural constituent of ribosome
	POPTR_0010s21740	RPL23AB (RIBOSOMAL PROTEIN L23AB); RNA binding / nucleotide binding / structural constituent of ribosome
	POPTR_0016s08070	RPL23AB (RIBOSOMAL PROTEIN L23AB); RNA binding / nucleotide binding / structural constituent of ribosome
Grapevine	GSVIVG00025041001	60S ribosomal protein L23a
Sorghum	Sb06g021660	60S ribosomal protein L23a
Maize	GRMZM2G083253	60S ribosomal protein L23a
	GRMZM2G166659	60S ribosomal protein L23a
Brachypodium	Bradi1g06220	60S ribosomal protein L23a
	Bradi5g14750	60S ribosomal protein L23a

Species	Orthologous genes	Putative function
Rice	LOC_Os02g39840	eukaryotic initiation factor iso-4F subunit p82-34, putative, expressed
	LOC_Os04g42140	eukaryotic initiation factor iso-4F subunit p82-34, putative, expressed
Arabidopsis	AT5G57870	MIF4G domain-containing protein / MA3 domain-containing protein
Poplar	POPTR_0006s19600	eukaryotic translation initiation factor 4F, putative / eIF-4F, putative
	POPTR_0006s28110	eukaryotic translation initiation factor 4F, putative / eIF-4F, putative
	POPTR_0018s02700	eukaryotic translation initiation factor 4F, putative / eIF-4F, putative
	POPTR_0018s11310	eukaryotic translation initiation factor 4F, putative / eIF-4F, putative
Grapevine	GSVIVG00023638001	eukaryotic translation initiation factor 4F / eIF-4F, putative
	GSVIVG00035980001	eukaryotic translation initiation factor 4 gamma
Sorghum	Sb06g021600	eukaryotic translation initiation factor 4 gamma
Maize	GRMZM2G098577	eukaryotic translation initiation factor 4 gamma
	GRMZM2G157061	eukaryotic translation initiation factor 4 gamma
Brachypodium	Bradi5g14690	Eukaryotic initiation factor iso-4F subunit p82-34

Species	Orthologous genes	Putative function
Rice	LOC_Os01g59140	DNA-directed RNA polymerases I, II, and III subunit RPABC1, putative, expressed
	LOC_Os04g41040	DNA-directed RNA polymerases I, II, and III subunit RPABC1, putative, expressed
Arabidopsis	AT3G22320	Eukaryotic rpb5 RNA polymerase subunit family protein
Poplar	POPTR_0006s16660	NRPB5; DNA binding / DNA-directed RNA polymerase
Grapevine	GSVIVG00015349001	RNA polymerase I, II and III 24.3 kDa subunit
Sorghum	Sb03g037480	DNA-directed RNA polymerases II 24 kDa polypeptide
	Sb06g020760	OJ991113_30.8 protein
Maize	GRMZM2G099183	OJ991113_30.8 protein

	GRMZM2G476009	DNA-directed RNA polymerases II 24 kDa polypeptide
Brachypodium	Bradi2g52780	DNA-directed RNA polymerases II 24 kDa polypeptide

**Species**	**Orthologous genes**	**Putative function**
Rice	LOC_Os02g37430	LSM domain containing protein, expressed
	LOC_Os04g39444	LSM domain containing protein, expressed
Arabidopsis	AT3G07590	Small nuclear ribonucleoprotein family protein
Poplar	POPTR_0002s05570	small nuclear ribonucleoprotein D1, putative / snRNP core protein D1, putative / Sm protein D1, putative
	POPTR_0005s22940	small nuclear ribonucleoprotein D1, putative / snRNP core protein D1, putative / Sm protein D1, putative
Grapevine	GSVIVG00011117001	Small nuclear ribonucleoprotein sm d1
Sorghum	Sb04g024330	Small nuclear ribonucleoprotein Sm D1
	Sb06g019630	Small nuclear ribonucleoprotein Sm D1
Maize	GRMZM2G027571	Small nuclear ribonucleoprotein Sm D1
	GRMZM2G416061	Small nuclear ribonucleoprotein Sm D1
Brachypodium	Bradi3g47250	Small nuclear ribonucleoprotein Sm D1
	Bradi5g13020	Small nuclear ribonucleoprotein Sm D1

**Species**	**Orthologous genes**	**Putative function**
Rice	LOC_Os04g38870	14-3-3 protein, putative, expressed
	LOC_Os08g33370	14-3-3 protein, putative, expressed
Arabidopsis	AT3G02520	general regulatory factor 7
	AT5G16050	general regulatory factor 5
Poplar	POPTR_0004s10120	GRF7 (GENERAL REGULATORY FACTOR 7); protein binding / protein phosphorylated amino acid binding
	POPTR_0017s13840	GRF7 (GENERAL REGULATORY FACTOR 7); protein binding / protein phosphorylated amino acid binding
Grapevine	GSVIVG00014210001	14-3-3 protein
Sorghum	Sb06g019100	14-3-3 GF14-6
	Sb07g020990	14-3-3
	Sb07g025680	14-3-3
Maize	AC217050.4_FG006	14-3-3
	GRMZM2G091155	14-3-3
	GRMZM2G102499	14-3-3 GF14-6
Brachypodium	Bradi3g36480	14-3-3 GF14-C
	Bradi5g12510	14-3-3 GF14-B

**Species**	**Orthologous genes**	**Putative function**
Rice	LOC_Os02g36500	expressed protein
	LOC_Os04g38310	expressed protein
Arabidopsis	AT4G39860	NA
Poplar	POPTR_0005s07750	unknown protein
	POPTR_0007s05500	unknown protein
Grapevine	GSVIVG00009018001	Conserved gene of unknown function
Sorghum	Sb06g018640	Conserved gene of unknown function

Additional File 2 cont.: Orthologous Proteins from Different Plant Species

Maize	GRMZM2G092107	Conserved gene of unknown function
Brachypodium	Bradi5g12080	Conserved gene of unknown function

Species	Orthologous genes	Putative function
Rice	LOC_Os04g36890	peptidyl-prolyl cis-trans isomerase, FKBP-type, putative, expressed
Arabidopsis	AT4G25340	FK506 BINDING PROTEIN 53
Poplar	POPTR_0012s14380	immunophilin-related / FKBP-type peptidyl-prolyl cis-trans isomerase-related
	POPTR_0015s14390	immunophilin-related / FKBP-type peptidyl-prolyl cis-trans isomerase-related
Grapevine	GSVIVG00018518001	FK506-binding protein
Sorghum	Sb06g018020	FK506-binding protein
Maize	GRMZM2G024811	FK506-binding protein
Brachypodium	Bradi5g10980	FK506-binding protein

Species	Orthologous genes	Putative function
Rice	LOC_Os04g34100	expressed protein
Arabidopsis	AT1G47420	succinate dehydrogenase 5
Poplar	POPTR_0014s03200	unknown protein
Grapevine	GSVIVG00009527001	Conserved gene of unknown function
Sorghum	Sb01g011580	Conserved gene of unknown function
Maize	GRMZM2G076524	Conserved gene of unknown function
	GRMZM2G146965	Conserved gene of unknown function
Brachypodium	Bradi1g50100	Conserved gene of unknown function
	Bradi5g09750	Conserved gene of unknown function

Species	Orthologous genes	Putative function
Rice	LOC_Os04g31320	SWIB/MDM2 domain containing protein, expressed
Arabidopsis	AT5G14170	SWIB/MDM2 domain superfamily protein
Poplar	POPTR_0001s33890	CHC1
	POPTR_0017s09890	CHC1
Grapevine	GSVIVG00008530001	Chromatin remodeling complex subunit
	GSVIVG00033102001	SWI/SNF complex component SNF12 homolog
Sorghum	Sb02g041010	SWI/SNF complex component SNF12 homolog
Maize	GRMZM2G052416	SWIB complex BAF60b domain-containing protein
Brachypodium	Bradi5g07730	Brg-1 associated factor

Species	Orthologous genes	Putative function
Rice	LOC_Os02g28810	ribosomal protein, putative, expressed
	LOC_Os04g28180	ribosomal protein, putative, expressed
Arabidopsis	AT5G20290	Ribosomal protein S8e family protein
	AT5G59240	Ribosomal protein S8e family protein
Poplar	POPTR_0001s26950	40S ribosomal protein S8 (RPS8B)
	POPTR_0009s06180	40S ribosomal protein S8 (RPS8B)

Additional File 2 cont.: Orthologous Proteins from Different Plant Species

	POPTR_0017s14180	40S ribosomal protein S8 (RPS8B)
Grapevine	GSVIVG00031566001	Ribosomal protein S8
Sorghum	Sb04g028530	Ribosomal protein S8
	Sb06g004770	Ribosomal protein S8
Maize	GRMZM2G030228	40S ribosomal protein S8
	GRMZM2G051848	40S ribosomal protein S8
	GRMZM2G063700	40S ribosomal protein S8
	GRMZM2G336875	40S ribosomal protein S8
	GRMZM2G360677	40S ribosomal protein S8
Brachypodium	Bradi1g51360	Ribosomal protein S8
	Bradi3g43560	Ribosomal protein S8

**Species**	**Orthologous genes**	**Putative function**
Rice	LOC_Os04g28090	MYB family transcription factor, putative, expressed
Arabidopsis	AT1G09770	cell division cycle 5
Poplar	POPTR_0013s04340	ATCDC5 (ARABIDOPSIS THALIANA CELL DIVISION CYCLE 5); DNA binding / transcription factor
	POPTR_0019s03520	ATCDC5 (ARABIDOPSIS THALIANA CELL DIVISION CYCLE 5); DNA binding / transcription factor
Grapevine	GSVIVG00023526001	CDC5
	GSVIVG00023527001	Cell division cycle 5
Sorghum	Sb03g044450	CDC5 protein
	Sb08g015280	CDC5 protein
Maize	AC203535.4_FG001	CDC5 protein
Brachypodium	Bradi4g07110	CDC5 protein

**Species**	**Orthologous genes**	**Putative function**
Rice	LOC_Os04g25550	FACT complex subunit SPT16, putative, expressed
	LOC_Os08g31240	FACT complex subunit SPT16, putative, expressed
Arabidopsis	AT4G10710	global transcription factor C
Poplar	POPTR_0005s00690	SPT16 (global transcription factor C)
	POPTR_0005s00700	SPT16 (global transcription factor C)
	POPTR_0013s00590	SPT16 (global transcription factor C)
	POPTR_0013s00600	SPT16 (global transcription factor C)
Grapevine	GSVIVG00031308001	Global transcription factor group
Sorghum	Sb01g002390	FACT complex subunit SPT16
	Sb07g026150	FACT complex subunit SPT16
Maize	GRMZM5G806358	FACT complex subunit SPT16
Brachypodium	Bradi1g59920	FACT complex subunit SPT16
	Bradi1g59940	FACT complex subunit SPT16

**Species**	**Orthologous genes**	**Putative function**
Rice	LOC_Os01g52470	elongation factor, putative, expressed
	LOC_Os01g53900	elongation factor, putative, expressed

Additional File 2 cont.: Orthologous Proteins from Different Plant Species

	LOC_Os02g32030	elongation factor, putative, expressed
	LOC_Os04g02820	elongation factor, putative, expressed
Arabidopsis	AT1G56070	Ribosomal protein S5/Elongation factor G/III/V family protein
	AT3G12915	Ribosomal protein S5/Elongation factor G/III/V family protein
Poplar	POPTR_0005s10090	LOS1; copper ion binding / translation elongation factor/ translation factor, nucleic acid binding
	POPTR_0007s08390	LOS1; copper ion binding / translation elongation factor/ translation factor, nucleic acid binding
	POPTR_0007s08400	LOS1; copper ion binding / translation elongation factor/ translation factor, nucleic acid binding
Sorghum	Sb01g002040	Elongation factor 2
	Sb03g033210	Elongation factor 2
	Sb03g034200	Elongation factor 2
Maize	AC203173.3_FG004	Elongation factor 2
	GRMZM2G040369	Elongation factor 2
	GRMZM2G095851	Elongation factor 2
	GRMZM2G113250	Elongation factor 2
Brachypodium	Bradi2g45070	Elongation factor 2
	Bradi3g44160	Elongation factor 2
	Bradi3g44480	Elongation factor 2

**Species**	**Orthologous genes**	**Putative function**
Rice	LOC_Os04g01740	heat shock protein, putative, expressed
Arabidopsis	AT5G52640	heat shock protein 90.1
Poplar	POPTR_0004s07190	ATHSP90.1 (HEAT SHOCK PROTEIN 90.1); ATP binding / unfolded protein binding
	POPTR_0017s01160	ATHSP90.1 (HEAT SHOCK PROTEIN 90.1); ATP binding / unfolded protein binding
Sorghum	Sb06g000660	Heat shock protein 82
Maize	GRMZM5G833699	Heat shock protein 82

**Species**	**Orthologous genes**	**Putative function**
Rice	LOC_Os03g63690	BCCIP, putative, expressed
Arabidopsis	AT2G44510	CDK inhibitor P21 binding protein
Poplar	POPTR_0001s46540	p21Cip1-binding protein-related
Grapevine	GSVIVG00011709001	Protein BCCIP homolog
	GSVIVG00023834001	Protein BCCIP homolog
Sorghum	Sb01g000790	p21 C-terminal-binding protein (Alternative splicing products)
Maize	GRMZM2G083410	p21 C-terminal-binding protein (Alternative splicing products)
Brachypodium	Bradi1g01070	p21 C-terminal-binding protein (Alternative splicing products)

**Species**	**Orthologous genes**	**Putative function**
Rice	LOC_Os03g63670	expressed protein
Arabidopsis	AT5G08550	GC-rich sequence DNA-binding factor-like protein
Poplar	POPTR_0008s00320	ILP1 (increased level of polyploidy1-1D); translation repressor
Grapevine	GSVIVG00011956001	Gc-rich sequence DNA-binding factor
Sorghum	Sb01g000820	Gc-rich sequence DNA-binding factor

Additional File 2 cont.: Orthologous Proteins from Different Plant Species

Maize	GRMZM5G832651	Gc-rich sequence DNA-binding factor
Brachypodium	Bradi2g26910	Gc-rich sequence DNA-binding factor

Species	Orthologous genes	Putative function
Rice	LOC_Os03g61990	glycine-rich RNA-binding protein 7, putative, expressed
Arabidopsis	AT3G26420	RNA-binding (RRM/RBD/RNP motifs) family protein with retrovirus zinc finger-like domain
Poplar	POPTR_0001s21460	ATRZ-1A; RNA binding / nucleotide binding
Grapevine	GSVIVG00001672001	Dc50
Sorghum	Sb01g002150	Glycine-rich RNA-binding protein 8
	Sb07g000400	Glycine-rich RNA-binding protein 8
Maize	GRMZM5G874478	Glycine-rich RNA-binding protein 8
Brachypodium	Bradi1g02570	Glycine-rich RNA-binding protein 8
	Bradi3g13040	RNA-binding protein

Species	Orthologous genes	Putative function
Rice	LOC_Os03g61640	ZOS3-23 - C2H2 zinc finger protein, expressed
Arabidopsis	AT3G05760	C2H2 and C2HC zinc fingers superfamily protein
Poplar	POPTR_0013s01160	nucleic acid binding / zinc ion binding
Grapevine	GSVIVG00031220001	Zinc finger protein
Sorghum	Sb03g003400	Zinc finger protein
Maize	GRMZM2G051458	Zinc finger protein
Brachypodium	Bradi1g02950	Zinc finger protein

Species	Orthologous genes	Putative function
Rice	LOC_Os03g61560	expressed protein
Arabidopsis	AT3G16810	pumilio 24
Poplar	POPTR_0008s23200	APUM24 (Arabidopsis Pumilio 24); RNA binding / binding
	POPTR_0010s00780	APUM24 (Arabidopsis Pumilio 24); RNA binding / binding
Grapevine	GSVIVG00035090001	Protein penguin
Sorghum	Sb01g002280	Protein penguin
Maize	GRMZM2G114692	Pumilio homolog 24
Brachypodium	Bradi1g03030	Protein penguin

Species	Orthologous genes	Putative function
Rice	LOC_Os03g59310	ribosomal protein, putative, expressed
	LOC_Os07g10660	ribosomal protein, putative, expressed
Arabidopsis	AT1G58380	Ribosomal protein S5 family protein
	AT1G58684	Ribosomal protein S5 family protein
	AT1G58983	Ribosomal protein S5 family protein
	AT1G59359	Ribosomal protein S5 family protein
	AT2G41840	Ribosomal protein S5 family protein
	AT3G57490	Ribosomal protein S5 family protein

Additional File 2 cont.: Orthologous Proteins from Different Plant Species

Poplar	POPTR_0001s26400	XW6; structural constituent of ribosome
	POPTR_0006s05090	40S ribosomal protein S2 (RPS2D)
	POPTR_0016s05530	40S ribosomal protein S2 (RPS2C)
Grapevine	GSVIVG00033299001	40S ribosomal protein S2
Sorghum	Sb01g004250	40S ribosomal protein S2
	Sb01g004260	40S ribosomal protein S2
	Sb02g006200	40S ribosomal protein S2
Maize	AC210013.4_FG019	40S ribosomal protein S2
	GRMZM2G092663	40S ribosomal protein S2
	GRMZM2G168149	40S ribosomal protein S2
Brachypodium	Bradi1g04660	40S ribosomal protein S2
	Bradi1g54270	40S ribosomal protein S2

Species	Orthologous genes	Putative function
Rice	LOC_Os03g58204	ribosomal protein L4, putative, expressed
	LOC_Os07g08330	ribosomal protein L4, putative, expressed
Arabidopsis	AT3G09630	Ribosomal protein L4/L1 family
	AT5G02870	Ribosomal protein L4/L1 family
Poplar	POPTR_0006s13470	60S ribosomal protein L4/L1 (RPL4A)
	POPTR_0006s13480	60S ribosomal protein L4/L1 (RPL4A)
	POPTR_0016s08520	60S ribosomal protein L4/L1 (RPL4A)
Grapevine	GSVIVG00025547001	60S ribosomal protein L4
Sorghum	Sb01g005210	60S ribosomal protein L4
	Sb02g004510	60S ribosomal protein L4
Maize	GRMZM2G007695	60S ribosomal protein L4
	GRMZM2G018197	60S ribosomal protein L4
	GRMZM2G068952	60S ribosomal protein L4
Brachypodium	Bradi1g05510	60S ribosomal protein L4
	Bradi1g55510	60S ribosomal protein L4

Species	Orthologous genes	Putative function
Rice	LOC_Os03g31210	UDP-glucose 6-dehydrogenase, putative, expressed
	LOC_Os03g55070	UDP-glucose 6-dehydrogenase, putative, expressed
	LOC_Os12g25690	UDP-glucose 6-dehydrogenase, putative, expressed
	LOC_Os12g25700	UDP-glucose 6-dehydrogenase, putative, expressed
Arabidopsis	AT3G29360	UDP-glucose 6-dehydrogenase family protein
	AT5G15490	UDP-glucose 6-dehydrogenase family protein
	AT5G39320	UDP-glucose 6-dehydrogenase family protein
Poplar	POPTR_0004s11760	UDP-glucose 6-dehydrogenase, putative
	POPTR_0008s09390	UDP-glucose 6-dehydrogenase, putative
	POPTR_0010s16730	UDP-glucose 6-dehydrogenase, putative
	POPTR_0017s12760	UDP-glucose 6-dehydrogenase, putative
Grapevine	GSVIVG00007910001	UDP-glucose dehydrogenase

	GSVIVG00012198001	UDP-glucose 6-dehydrogenase
Sorghum	Sb01g007580	UDP-glucose 6-dehydrogenase
Maize	GRMZM2G328500	UDP-glucose 6-dehydrogenase
	GRMZM5G862540	UDP-glucose 6-dehydrogenase
Brachypodium	Bradi1g08120	UDP-glucose 6-dehydrogenase
	Bradi4g25140	UDP-glucose 6-dehydrogenase

**Species**	**Orthologous genes**	**Putative function**
Rice	LOC_Os03g54890	ribosomal protein L13, putative, expressed
	LOC_Os07g01870	ribosomal protein L13, putative, expressed
Arabidopsis	AT3G07110	Ribosomal protein L13 family protein
	AT3G24830	Ribosomal protein L13 family protein
	AT4G13170	Ribosomal protein L13 family protein
	AT5G48760	Ribosomal protein L13 family protein
Poplar	POPTR_0001s32170	60S ribosomal protein L13A (RPL13aD)
	POPTR_0017s08170	60S ribosomal protein L13A (RPL13aD)
Grapevine	GSVIVG00021080001	60S ribosomal protein L13a
	GSVIVG00028661001	60S ribosomal protein L13a
Sorghum	Sb01g007690	60S ribosomal protein L13a
	Sb09g001930	60S ribosomal protein L13a
Maize	GRMZM2G090422	60S ribosomal protein L13a
Brachypodium	Bradi1g08260	Ribosomal protein L13a
	Bradi1g59280	Ribosomal protein L13a

**Species**	**Orthologous genes**	**Putative function**
Rice	LOC_Os03g06670	Core histone H2A/H2B/H3/H4 domain containing protein, putative, expressed
	LOC_Os03g53190	Core histone H2A/H2B/H3/H4 domain containing protein, putative, expressed
	LOC_Os10g28230	Core histone H2A/H2B/H3/H4 domain containing protein, putative, expressed
Arabidopsis	AT1G52740	histone H2A protein 9
	AT3G54560	histone H2A 11
Poplar	POPTR_0002s04720	HTA11; DNA binding
	POPTR_0005s23810	HTA11; DNA binding
	POPTR_0006s26540	HTA9 (HISTONE H2A PROTEIN 9); DNA binding
	POPTR_0006s26550	HTA9 (HISTONE H2A PROTEIN 9); DNA binding
	POPTR_0018s01310	HTA9 (HISTONE H2A PROTEIN 9); DNA binding
	POPTR_0018s01320	HTA9 (HISTONE H2A PROTEIN 9); DNA binding
Grapevine	GSVIVG00003139001	Histone H2A
	GSVIVG00017218001	Histone H2A
	GSVIVG00035768001	Histone H2A
Sorghum	Sb04g025140	Histone H2A
Maize	GRMZM2G050833	Histone H2A
	GRMZM2G056231	Histone H2A

Additional File 2 cont.: Orthologous Proteins from Different Plant Species

	GRMZM2G149775	Histone H2A
Brachypodium	Bradi1g09060	histone H2A variant 3
	Bradi3g26880	histone H2A variant 2

**Species**	**Orthologous genes**	**Putative function**
Rice	LOC_Os03g52470	WD domain, G-beta repeat domain containing protein, expressed
Arabidopsis	AT1G27470	transducin family protein / WD-40 repeat family protein
	AT4G07410	Transducin family protein / WD-40 repeat family protein
Poplar	POPTR_0002s11830	transducin family protein / WD-40 repeat family protein
	POPTR_0014s01520	transducin family protein / WD-40 repeat family protein
Grapevine	GSVIVG00009321001	Nucleotide binding protein
Sorghum	Sb01g008930	Nucleotide binding protein
Maize	GRMZM2G065822	Nucleotide binding protein
	GRMZM2G164085	Nucleotide binding protein
Brachypodium	Bradi1g09530	Nucleotide binding protein

**Species**	**Orthologous genes**	**Putative function**
Rice	LOC_Os03g51200	Core histone H2A/H2B/H3/H4 domain containing protein, putative, expressed
	LOC_Os12g34510	Core histone H2A/H2B/H3/H4 domain containing protein, putative, expressed
Arabidopsis	AT1G08880	Histone superfamily protein
	AT1G54690	gamma histone variant H2AX
Poplar	POPTR_0005s04260	GAMMA-H2AX (GAMMA HISTONE VARIANT H2AX); DNA binding
	POPTR_0013s02990	GAMMA-H2AX (GAMMA HISTONE VARIANT H2AX); DNA binding
	POPTR_0369s00210	GAMMA-H2AX (GAMMA HISTONE VARIANT H2AX); DNA binding
Grapevine	GSVIVG00011033001	Histone H2A
Sorghum	Sb01g009820	Histone H2A
	Sb01g028960	Histone H2A
	Sb08g016830	Histone H2A
Maize	GRMZM2G046055	Histone H2A
Brachypodium	Bradi1g10390	Histone H2A
	Bradi4g06010	histone H2AXb

**Species**	**Orthologous genes**	**Putative function**
Rice	LOC_Os03g50480	phosphoglucomutase, putative, expressed
Arabidopsis	AT1G23190	Phosphoglucomutase/phosphomannomutase family protein
	AT1G70730	Phosphoglucomutase/phosphomannomutase family protein
Poplar	POPTR_0010s11970	phosphoglucomutase, cytoplasmic, putative / glucose phosphomutase, putative
Grapevine	GSVIVG00011700001	Phosphoglucomutase
Sorghum	Sb01g010280	Phosphoglucomutase, cytoplasmic 2
Maize	GRMZM2G023289	Phosphoglucomutase, cytoplasmic 2
	GRMZM2G109383	Phosphoglucomutase, cytoplasmic 1
Brachypodium	Bradi1g11440	Phosphoglucomutase

Additional File 2 cont.: Orthologous Proteins from Different Plant Species

Species	Orthologous genes	Putative function
Rice	LOC_Os03g50290	14-3-3 protein, putative, expressed
Arabidopsis	AT1G22300	general regulatory factor 10
	AT5G38480	general regulatory factor 3
Poplar	POPTR_0002s09770	GRF12 (GENERAL REGULATORY FACTOR 12); protein binding / protein phosphorylated amino acid binding
Grapevine	GSVIVG00009037001	14-3-3
Brachypodium	Bradi1g11290	14-3-3 A

Species	Orthologous genes	Putative function
Rice	LOC_Os03g49210	BRCA1 C Terminus domain containing protein, expressed
Arabidopsis	AT5G14520	pescadillo-related
Poplar	POPTR_0004s12080	pescadillo-related
Grapevine	GSVIVG00032412001	Pescadillo
Sorghum	Sb01g011100	Pescadillo
Maize	GRMZM2G068471	Pescadillo
Brachypodium	Bradi1g11730	Pescadillo

Species	Orthologous genes	Putative function
Rice	LOC_Os03g47800	RNA recognition motif containing protein, expressed
	LOC_Os07g08960	RNA recognition motif containing protein, expressed
Arabidopsis	AT1G60650	RNA-binding (RRM/RBD/RNP motifs) family protein with retrovirus zinc finger-like domain
	AT5G04280	RNA-binding (RRM/RBD/RNP motifs) family protein with retrovirus zinc finger-like domain
Poplar	POPTR_0008s03290	glycine-rich RNA-binding protein
	POPTR_0010s23550	glycine-rich RNA-binding protein
Grapevine	GSVIVG00016195001	Glycine-rich RNA-binding protein
	GSVIVG00033666001	Glycine-rich RNA-binding protein
Sorghum	Sb01g011910	Glycine-rich RNA-binding protein 7
	Sb02g004930	Glycine-rich RNA-binding protein 7
Maize	GRMZM2G082931	Glycine-rich RNA-binding protein 7
	GRMZM2G083783	Glycine-rich RNA-binding protein
	GRMZM2G161242	Glycine-rich RNA-binding protein
Brachypodium	Bradi1g12460	Glycine-rich RNA-binding protein
	Bradi1g55020	Glycine-rich RNA-binding protein 7

Species	Orthologous genes	Putative function
Rice	LOC_Os03g46770	RNA recognition motif containing protein, expressed
	LOC_Os12g43600	RNA recognition motif containing protein, expressed
Arabidopsis	AT2G21660	"cold, circadian rhythm, and rna binding 2"
Poplar	POPTR_0004s16260	GR-RBP8; RNA binding / nucleic acid binding / nucleotide binding
	POPTR_0009s11920	GR-RBP8; RNA binding / nucleic acid binding / nucleotide binding
Grapevine	GSVIVG0003197300	Glycine-rich RNA-binding protein

	1	
Sorghum	Sb01g012300	Glycine-rich RNA-binding protein
	Sb08g022740	Glycine-rich RNA-binding protein 1
Maize	GRMZM2G080603	Glycine-rich RNA-binding protein 2
	GRMZM2G165901	Glycine-rich RNA-binding, abscisic acid-inducible protein
Brachypodium	Bradi1g12790	glycine-rich RNA binding protein
	Bradi4g00940	Glycine rich protein, RNA binding protein

**Species**	**Orthologous genes**	**Putative function**
Rice	LOC_Os03g46490	40S ribosomal protein S21, putative, expressed
Arabidopsis	AT3G53890	Ribosomal protein S21e
	AT5G27700	Ribosomal protein S21e
Poplar	POPTR_0013s01830	structural constituent of ribosome
Grapevine	GSVIVG0003115100 1	40S ribosomal protein S21e
Sorghum	Sb01g011430	40S ribosomal protein S21
	Sb01g035580	40S ribosomal protein S21
Maize	GRMZM2G125300	40S ribosomal protein S21
	GRMZM2G134109	40S ribosomal protein S21
Brachypodium	Bradi1g18190	40S ribosomal protein S21

**Species**	**Orthologous genes**	**Putative function**
Rice	LOC_Os03g42770	expressed protein
Arabidopsis	AT4G05410	Transducin/WD40 repeat-like superfamily protein
	AT4G21130	Transducin/WD40 repeat-like superfamily protein
Poplar	POPTR_0005s06230	transducin family protein / WD-40 repeat family protein
	POPTR_0007s03960	transducin family protein / WD-40 repeat family protein
Grapevine	GSVIVG0002161800 1	U3 small nucleolar RNA-interacting protein
Sorghum	Sb01g013700	U3 snoRNP-associated
Maize	GRMZM2G015005	Transducin family protein
Brachypodium	Bradi4g01810	U3 snoRNP-associated

**Species**	**Orthologous genes**	**Putative function**
Rice	LOC_Os03g42110	semialdehyde dehydrogenase, NAD binding domain containing protein, putative, expressed
	LOC_Os10g35170	semialdehyde dehydrogenase, NAD binding domain containing protein, putative, expressed
Arabidopsis	AT2G19940	"oxidoreductases, acting on the aldehyde or oxo group of donors, NAD or NADP as acceptor;copper ion binding"
Poplar	POPTR_0004s15290	N-acetyl-gamma-glutamyl-phosphate reductase/ NAD or NADH binding / binding / catalytic/ oxidoreductase
Grapevine	GSVIVG0003180800 1	N-acetyl-gamma-glutamyl-phosphate reductase
Sorghum	Sb01g014570	N-acetyl-gamma-glutamyl-phosphate reductase
Maize	GRMZM2G038848	N-acetyl-gamma-glutamyl-phosphate reductase
Brachypodium	Bradi1g14590	N-acetyl-gamma-glutamyl-phosphate reductase, chloroplastic

**Species**	**Orthologous genes**	**Putative function**

Additional File 2 cont.: Orthologous Proteins from Different Plant Species

Rice	LOC_Os03g41612	ribosomal protein L25, putative, expressed
Arabidopsis	AT5G66860	"Ribosomal protein L25/Gln-tRNA synthetase, anti-codon-binding domain"
Poplar	POPTR_0005s14160	unknown protein
	POPTR_0007s11440	unknown protein
	POPTR_0684s00200	unknown protein
Grapevine	GSVIVG00003545001	50S ribosomal protein L25
	GSVIVG00004324001	50S ribosomal protein L25
Sorghum	Sb01g014640	5S rRNA binding protein
Maize	GRMZM2G067306	5S rRNA binding protein
Brachypodium	Bradi1g14670	Ribosomal 5S rRNA E-loop binding protein Ctc/L25/TL5 containing protein

**Species**	**Orthologous genes**	**Putative function**
Rice	LOC_Os03g38000	40S ribosomal protein S3-1, putative, expressed
Arabidopsis	AT2G31610	Ribosomal protein S3 family protein
	AT3G53870	Ribosomal protein S3 family protein
	AT5G35530	Ribosomal protein S3 family protein
Poplar	POPTR_0006s23870	40S ribosomal protein S3 (RPS3C)
	POPTR_0015s08310	40S ribosomal protein S3 (RPS3C)
	POPTR_0018s08510	40S ribosomal protein S3 (RPS3C)
Grapevine	GSVIVG00015110001	Ribosomal protein
	GSVIVG00016775001	Ribosomal protein
	GSVIVG00035451001	40S ribosomal protein S3
Sorghum	Sb07g004290	40S ribosomal protein S3
Maize	GRMZM2G074300	40S ribosomal protein S3
	GRMZM2G099352	40S ribosomal protein S3
Brachypodium	Bradi3g16150	40S ribosomal protein S3

**Species**	**Orthologous genes**	**Putative function**
Rice	LOC_Os03g37970	ribosomal protein L13, putative, expressed
	LOC_Os06g02510	ribosomal protein L13, putative, expressed
Arabidopsis	AT3G49010	breast basic conserved 1
	AT5G23900	Ribosomal protein L13e family protein
Poplar	POPTR_0001s01350	ATBBC1 (ARABIDOPSIS THALIANA BREAST BASIC CONSERVED 1); structural constituent of ribosome
	POPTR_0003s10180	ATBBC1 (ARABIDOPSIS THALIANA BREAST BASIC CONSERVED 1); structural constituent of ribosome
	POPTR_0013s02860	ATBBC1 (ARABIDOPSIS THALIANA BREAST BASIC CONSERVED 1); structural constituent of ribosome
	POPTR_0016s08310	ATBBC1 (ARABIDOPSIS THALIANA BREAST BASIC CONSERVED 1); structural constituent of ribosome
Grapevine	GSVIVG00027212001	60S ribosomal protein L13
	GSVIVG00030767001	60S ribosomal protein L13
Sorghum	Sb03g044290	60S ribosomal protein L13
	Sb09g005460	60S ribosomal protein L13
	Sb09g028960	60S ribosomal protein L13

Maize	GRMZM2G145280	60S ribosomal protein L13
	GRMZM2G409407	60S ribosomal protein L13
Brachypodium	Bradi3g01600	60S ribosomal protein L13
	Bradi3g16170	60S ribosomal protein L13

**Species**	**Orthologous genes**	**Putative function**
Rice	LOC_Os03g27260	40S ribosomal protein S6, putative, expressed
	LOC_Os07g42950	40S ribosomal protein S6, putative, expressed
Arabidopsis	AT4G31700	ribosomal protein S6
	AT5G10360	Ribosomal protein S6e
Poplar	POPTR_0001s31850	EMB3010 (embryo defective 3010); structural constituent of ribosome
	POPTR_0002s09970	EMB3010 (embryo defective 3010); structural constituent of ribosome
	POPTR_0005s07380	EMB3010 (embryo defective 3010); structural constituent of ribosome
	POPTR_0005s17280	EMB3010 (embryo defective 3010); structural constituent of ribosome
	POPTR_0007s05090	EMB3010 (embryo defective 3010); structural constituent of ribosome
Grapevine	GSVIVG00003418001	S6 ribosomal protein
	GSVIVG00009072001	S6 ribosomal protein
	GSVIVG00015776001	S6 ribosomal protein
Sorghum	Sb02g039650	40S ribosomal protein S6
	Sb08g015010	40S ribosomal protein S6
Maize	GRMZM2G054136	40S ribosomal protein S6
	GRMZM5G851698	40S ribosomal protein S6
Brachypodium	Bradi1g60730	40S ribosomal protein S6

**Species**	**Orthologous genes**	**Putative function**
Rice	LOC_Os03g26630	SAP domain containing protein, expressed
Arabidopsis	AT4G39680	SAP domain-containing protein
Poplar	POPTR_0005s08230	SAP domain-containing protein
	POPTR_0007s06040	SAP domain-containing protein
Grapevine	GSVIVG00008977001	SAP domain containing protein
	GSVIVG00022190001	SAP domain containing protein
Sorghum	Sb01g033900	SAP domain containing protein
Maize	GRMZM2G113139	SAP domain containing protein
	GRMZM2G151387	SAP domain containing protein
Brachypodium	Bradi1g61000	SAP domain containing protein

**Species**	**Orthologous genes**	**Putative function**
Rice	LOC_Os03g22880	nucleolar protein 5A, putative, expressed
Arabidopsis	AT1G56110	homolog of nucleolar protein NOP56
	AT3G12860	NOP56-like pre RNA processing ribonucleoprotein
Poplar	POPTR_0012s09730	NOP56 (Arabidopsis homolog of nucleolar protein Nop56)
	POPTR_0015s10500	NOP56 (Arabidopsis homolog of nucleolar protein Nop56)

Additional File 2 cont.: Orthologous Proteins from Different Plant Species

Grapevine	GSVIVG00008506001	Nucleolar protein nop56
Sorghum	Sb01g035290	Nucleolar protein Nop56
	Sb02g042030	Nucleolar protein Nop56
Maize	GRMZM2G110233	Nucleolar protein Nop56
	GRMZM2G144995	Nucleolar protein Nop56
Brachypodium	Bradi1g18730	Nucleolar protein Nop56
	Bradi1g32180	Nucleolar protein Nop56

**Species**	**Orthologous genes**	**Putative function**
Rice	LOC_Os03g22730	nucleolar protein NOP5-1, putative
	LOC_Os03g22740	nucleolar protein NOP5-1, putative
Arabidopsis	AT3G05060	NOP56-like pre RNA processing ribonucleoprotein
	AT5G27120	NOP56-like pre RNA processing ribonucleoprotein
Poplar	POPTR_0005s04710	SAR DNA-binding protein, putative
	POPTR_0013s03260	SAR DNA-binding protein, putative
Grapevine	GSVIVG00021905001	Matrix attachment region binding protein
Sorghum	Sb01g035410	SAR DNA binding protein
	Sb03g044260	SAR DNA binding protein
Maize	GRMZM2G124576	Nucleolar protein NOP5
	GRMZM2G125148	Nucleolar protein NOP5
Brachypodium	Bradi1g18440	SAR DNA binding protein
	Bradi1g62430	SAR DNA binding protein

**Species**	**Orthologous genes**	**Putative function**
Rice	LOC_Os03g22730	nucleolar protein NOP5-1, putative
	LOC_Os03g22740	nucleolar protein NOP5-1, putative
Arabidopsis	AT3G05060	NOP56-like pre RNA processing ribonucleoprotein
	AT5G27120	NOP56-like pre RNA processing ribonucleoprotein
Poplar	POPTR_0005s04710	SAR DNA-binding protein, putative
	POPTR_0013s03260	SAR DNA-binding protein, putative
Grapevine	GSVIVG00021905001	Matrix attachment region binding protein
Sorghum	Sb01g035410	SAR DNA binding protein
	Sb03g044260	SAR DNA binding protein
Maize	GRMZM2G124576	Nucleolar protein NOP5
	GRMZM2G125148	Nucleolar protein NOP5
Brachypodium	Bradi1g18440	SAR DNA binding protein
	Bradi1g62430	SAR DNA binding protein

**Species**	**Orthologous genes**	**Putative function**
Rice	LOC_Os03g22320	utp14 protein, putative, expressed
Arabidopsis	AT4G02400	U3 ribonucleoprotein (Utp) family protein
	AT5G08600	U3 ribonucleoprotein (Utp) family protein
Poplar	POPTR_0002s20500	unknown protein

Additional File 2 cont.: Orthologous Proteins from Different Plant Species

	POPTR_0014s12300	unknown protein
Grapevine	GSVIVG00028028001	U3 small nucleolar RNA-associated protein
Sorghum	Sb01g035770	U3 small nucleolar RNA-associated protein
Maize	GRMZM2G158676	U3 small nucleolar RNA-associated protein
Brachypodium	Bradi1g62850	U3 small nucleolar RNA-associated protein

**Species**	**Orthologous genes**	**Putative function**
Rice	LOC_Os03g22180	60S ribosomal protein L18-3, putative, expressed
	LOC_Os07g47780	60S ribosomal protein L18-3, putative, expressed
Arabidopsis	AT3G05590	ribosomal protein L18
	AT5G27850	Ribosomal protein L18e/L15 superfamily protein
Poplar	POPTR_0002s20400	RPL18 (RIBOSOMAL PROTEIN L18); structural constituent of ribosome
	POPTR_0005s02330	RPL18 (RIBOSOMAL PROTEIN L18); structural constituent of ribosome
	POPTR_0013s01520	RPL18 (RIBOSOMAL PROTEIN L18); structural constituent of ribosome
	POPTR_0014s12210	RPL18 (RIBOSOMAL PROTEIN L18); structural constituent of ribosome
Grapevine	GSVIVG00028036001	60S ribosomal protein L18
	GSVIVG00031168001	60S ribosomal protein L18
	GSVIVG00035120001	60S ribosomal protein L18
Sorghum	Sb01g035860	Ribosomal protein L18
	Sb02g042750	Ribosomal protein L18
Maize	GRMZM2G322413	Ribosomal protein L18
Brachypodium	Bradi1g17970	Ribosomal protein L18
	Bradi1g62930	Ribosomal protein L18

**Species**	**Orthologous genes**	**Putative function**
Rice	LOC_Os03g21940	60S ribosomal protein L19-3, putative, expressed
	LOC_Os03g38260	60S ribosomal protein L19-3, putative, expressed
Arabidopsis	AT1G02780	Ribosomal protein L19e family protein
	AT3G16780	Ribosomal protein L19e family protein
	AT4G02230	Ribosomal protein L19e family protein
Poplar	POPTR_0004s07620	60S ribosomal protein L19 (RPL19B)
	POPTR_0012s03450	60S ribosomal protein L19 (RPL19B)
	POPTR_0015s05500	60S ribosomal protein L19 (RPL19B)
	POPTR_0017s01890	60S ribosomal protein L19 (RPL19B)
Grapevine	GSVIVG00035096001	Ribosomal protein L19
Sorghum	Sb03g025960	Ribosomal protein L19
	Sb04g003890	Ribosomal protein L19
Maize	GRMZM2G116135	60S ribosomal protein L19-3
	GRMZM5G887054	60S ribosomal protein L19-3
Brachypodium	Bradi1g17920	Ribosomal protein L19
	Bradi1g63010	Ribosomal protein L19

Additional File 2 cont.: Orthologous Proteins from Different Plant Species

Species	Orthologous genes	Putative function
Rice	LOC_Os03g21530	AARP2CN domain containing protein, expressed
Arabidopsis	AT1G06720	P-loop containing nucleoside triphosphate hydrolases superfamily protein
Poplar	POPTR_0017s04900	unknown protein
Grapevine	GSVIVG00020936001	Ribosome biogenesis protein bms1
Sorghum	Sb01g036270	Ribosome biogenesis protein bms1
Maize	GRMZM2G399320	Ribosome biogenesis protein bms1
Brachypodium	Bradi1g63330	Ribosome biogenesis protein BMS1 homolog

Species	Orthologous genes	Putative function
Rice	LOC_Os03g18840	NUC189 domain containing protein, expressed
Arabidopsis	AT5G11240	transducin family protein / WD-40 repeat family protein
Poplar	POPTR_0010s00760	transducin family protein / WD-40 repeat family protein
Grapevine	GSVIVG00035079001	Nucleotide binding protein
Sorghum	Sb01g037980	Nucleotide binding protein
Maize	GRMZM2G095043	transducin family protein / WD-40 repeat family protein
Brachypodium	Bradi1g64930	Nucleotide binding protein

Species	Orthologous genes	Putative function
Rice	LOC_Os03g18570	40S ribosomal protein S7, putative, expressed
	LOC_Os03g18580	40S ribosomal protein S7, putative
	LOC_Os05g27940	40S ribosomal protein S7, putative, expressed
Arabidopsis	AT1G48830	Ribosomal protein S7e family protein
	AT3G02560	Ribosomal protein S7e family protein
	AT5G16130	Ribosomal protein S7e family protein
Poplar	POPTR_0004s09830	40S ribosomal protein S7 (RPS7B)
	POPTR_0005s15580	40S ribosomal protein S7 (RPS7B)
	POPTR_0006s08820	40S ribosomal protein S7 (RPS7B)
	POPTR_0016s10560	40S ribosomal protein S7 (RPS7B)
Grapevine	GSVIVG00008307001	40S ribosomal protein S7
	GSVIVG00031018001	40S ribosomal protein S7
Sorghum	Sb01g038170	40S ribosomal protein S7
	Sb09g016170	40S ribosomal protein S7
Maize	GRMZM2G030016	40S ribosomal protein S7
	GRMZM2G053652	40S ribosomal protein S7
Brachypodium	Bradi1g65150	Ribosomal protein S7

Species	Orthologous genes	Putative function
Rice	LOC_Os03g18510	expressed protein
Arabidopsis	AT2G40290	Eukaryotic translation initiation factor 2 subunit 1
Poplar	POPTR_0008s07250	eukaryotic translation initiation factor 2 subunit 1, putative / eIF-2A, putative / eIF-2-alpha, putative
	POPTR_0010s19230	eukaryotic translation initiation factor 2 subunit 1, putative / eIF-2A, putative / eIF-2-alpha, putative

Additional File 2 cont.: Orthologous Proteins from Different Plant Species

Grapevine	GSVIVG00024676001	Eukaryotic translation initiation factor
Sorghum	Sb01g038230	Eukaryotic translation initiation factor 2 alpha subunit
Maize	GRMZM2G058138	Eukaryotic translation initiation factor 2 alpha subunit
	GRMZM2G083262	Eukaryotic translation initiation factor 2 alpha subunit
Brachypodium	Bradi1g16700	Eukaryotic translation initiation factor 2 alpha
	Bradi1g65210	Eukaryotic translation initiation factor 2 alpha

**Species**	**Orthologous genes**	**Putative function**
Rice	LOC_Os03g18410	DNA-directed RNA polymerase subunit, putative, expressed
Arabidopsis	AT1G60620	RNA polymerase I subunit 43
	AT1G60850	DNA-directed RNA polymerase family protein
Poplar	POPTR_0001s37820	ATRPAC43; DNA binding / DNA-directed RNA polymerase/ protein dimerization
Grapevine	GSVIVG00014713001	DNA-directed RNA polymerase I/III subunits
Sorghum	Sb04g006420	RNA polymerase Rpb3/Rpb11 dimerisation domain containing protein
Maize	GRMZM2G025356	RNA polymerase Rpb3/Rpb11 dimerisation domain containing protein
Brachypodium	Bradi1g15810	RNA polymerase Rpb3/Rpb11 dimerisation domain containing protein

**Species**	**Orthologous genes**	**Putative function**
Rice	LOC_Os01g31800	Core histone H2A/H2B/H3/H4 domain containing protein, putative, expressed
	LOC_Os03g17100	Core histone H2A/H2B/H3/H4 domain containing protein, putative, expressed

**Species**	**Orthologous genes**	**Putative function**
Rice	LOC_Os03g15900	SH3 domain containing protein, expressed
Arabidopsis	AT2G07360	SH3 domain-containing protein
Poplar	POPTR_0018s14630	SH3 domain-containing protein
Grapevine	GSVIVG00022289001	SH3 domain containing protein
Sorghum	Sb01g040090	SH3 domain containing protein
Maize	GRMZM2G012319	SH3 domain containing protein
Brachypodium	Bradi1g67140	SH3 domain containing protein

**Species**	**Orthologous genes**	**Putative function**
Rice	LOC_Os03g14530	S10/S20 domain containing ribosomal protein, putative, expressed
	LOC_Os06g04290	S10/S20 domain containing ribosomal protein, putative, expressed
	LOC_Os10g08930	S10/S20 domain containing ribosomal protein, putative, expressed
Arabidopsis	AT3G45030	Ribosomal protein S10p/S20e family protein
	AT3G47370	Ribosomal protein S10p/S20e family protein
	AT5G62300	Ribosomal protein S10p/S20e family protein
Poplar	POPTR_0012s14470	40S ribosomal protein S20 (RPS20C)
	POPTR_0015s14510	40S ribosomal protein S20 (RPS20C)
Grapevine	GSVIVG00018553001	Ribosomal protein S10, eukaryotic and archaeal form
Sorghum	Sb01g041040	40S ribosomal protein S20
	Sb10g002240	40S ribosomal protein S20

Additional File 2 cont.: Orthologous Proteins from Different Plant Species

Maize	GRMZM2G067303	40S ribosomal protein S20
	GRMZM2G092296	40S ribosomal protein S20
Brachypodium	Bradi1g51650	40S ribosomal protein S20

Species	Orthologous genes	Putative function
Rice	LOC_Os03g13800	ribosomal protein L7Ae, putative, expressed
	LOC_Os10g03540	ribosomal protein L7Ae, putative, expressed
Arabidopsis	AT4G12600	Ribosomal protein L7Ae/L30e/S12e/Gadd45 family protein
	AT4G22380	Ribosomal protein L7Ae/L30e/S12e/Gadd45 family protein
	AT5G20160	Ribosomal protein L7Ae/L30e/S12e/Gadd45 family protein
Poplar	POPTR_0013s12110	ribosomal protein L7Ae/L30e/S12e/Gadd45 family protein
	POPTR_0019s11700	ribosomal protein L7Ae/L30e/S12e/Gadd45 family protein
Grapevine	GSVIVG00015520001	Ribosomal protein l7ae
	GSVIVG00016438001	Ribosomal protein l7ae
Sorghum	Sb01g041420	NHP2 1
	Sb01g047920	NHP2 1
Maize	GRMZM2G140799	NHP2 1
	GRMZM5G868062	NHP2 1
Brachypodium	Bradi3g21230	Ribosomal protein l7ae

Species	Orthologous genes	Putative function
Rice	LOC_Os03g17000	NAD dependent epimerase/dehydratase family domain containing protein, expressed
Arabidopsis	AT1G53500	NAD-dependent epimerase/dehydratase family protein
	AT1G78570	rhamnose biosynthesis 1
	AT3G14790	rhamnose biosynthesis 3
Poplar	POPTR_0001s39210	RHM1 (RHAMNOSE BIOSYNTHESIS 1); UDP-L-rhamnose synthase/ UDP-glucose 4,6-dehydratase/ catalytic
	POPTR_0006s28750	RHM1 (RHAMNOSE BIOSYNTHESIS 1); UDP-L-rhamnose synthase/ UDP-glucose 4,6-dehydratase/ catalytic
	POPTR_0011s10480	RHM1 (RHAMNOSE BIOSYNTHESIS 1); UDP-L-rhamnose synthase/ UDP-glucose 4,6-dehydratase/ catalytic
Grapevine	GSVIVG00033176001	rhamnose biosynthetic enzyme 1
Sorghum	Sb01g039220	Rhamnose synthase
	Sb01g039340	rhamnose biosynthetic enzyme 1
	Sb09g008220	RHM1
Maize	GRMZM2G031311	RHM1
	GRMZM2G044281	RHM1/ROL1
	GRMZM2G166767	RHM1/ROL1
Brachypodium	Bradi3g08850	RHM1

Species	Orthologous genes	Putative function
Rice	LOC_Os02g18550	40S ribosomal protein S3a, putative, expressed
	LOC_Os03g10340	40S ribosomal protein S3a, putative, expressed
	LOC_Os12g21798	40S ribosomal protein S3a, putative, expressed
Arabidopsis	AT3G04840	Ribosomal protein S3Ae

Additional File 2 cont.: Orthologous Proteins from Different Plant Species

	AT4G34670	Ribosomal protein S3Ae
Poplar	POPTR_0005s05280	40S ribosomal protein S3A (RPS3aB)
	POPTR_0008s15610	40S ribosomal protein S3A (RPS3aB)
	POPTR_0010s09330	40S ribosomal protein S3A (RPS3aB)
Grapevine	GSVIVG00018024001	40S ribosomal protein S3a-1
Sorghum	Sb02g038365	40S ribosomal protein S3a
	Sb02g038370	40S ribosomal protein S3a
Maize	GRMZM2G030915	40S ribosomal protein S3a
	GRMZM2G145258	40S ribosomal protein S3a
Brachypodium	Bradi1g71200	40S ribosomal protein S3a
	Bradi1g78170	40S ribosomal protein S3a
	Bradi3g01210	40S ribosomal protein S3a

**Species**	**Orthologous genes**	**Putative function**
Rice	LOC_Os03g08810	expressed protein
Arabidopsis	AT5G62440	Protein of unknown function (DUF3223)
Poplar	POPTR_0005s03610	unknown protein
	POPTR_0013s02280	unknown protein
	POPTR_0013s02340	unknown protein
Grapevine	GSVIVG00007999001	EMB514
Sorghum	Sb01g044760	EMB514
Maize	GRMZM2G091362	EMB514
Brachypodium	Bradi1g72100	EMB514

**Species**	**Orthologous genes**	**Putative function**
Rice	LOC_Os03g08440	ribosomal protein S2, putative, expressed
	LOC_Os07g42450	ribosomal protein S2, putative, expressed
Arabidopsis	AT1G72370	40s ribosomal protein SA
Poplar	POPTR_0001s16430	P40; structural constituent of ribosome
	POPTR_0003s06880	RPSAb (40S ribosomal protein SA B); structural constituent of ribosome
	POPTR_0012s11830	RPSAb (40S ribosomal protein SA B); structural constituent of ribosome
	POPTR_0015s12460	P40; structural constituent of ribosome
Grapevine	GSVIVG00016695001	40S ribosomal protein SA
Sorghum	Sb02g039260	40S ribosomal protein SA
	Sb02g039270	40S ribosomal protein SA
	Sb05g027730	40S ribosomal protein SA
Maize	GRMZM2G092719	40S ribosomal protein SA
	GRMZM2G099657	40S ribosomal protein SA
	GRMZM2G126821	40S ribosomal protein SA
	GRMZM2G145308	40S ribosomal protein SA
	GRMZM2G159237	40S ribosomal protein SA
Brachypodium	Bradi1g20880	40S ribosomal protein SA
	Bradi1g23780	40S ribosomal protein SA

Additional File 2 cont.: Orthologous Proteins from Different Plant Species

	Bradi1g72480	40S ribosomal protein

Species	Orthologous genes	Putative function
Rice	LOC_Os03g06670	Core histone H2A/H2B/H3/H4 domain containing protein, putative, expressed
	LOC_Os03g53190	Core histone H2A/H2B/H3/H4 domain containing protein, putative, expressed
	LOC_Os10g28230	Core histone H2A/H2B/H3/H4 domain containing protein, putative, expressed
Arabidopsis	AT1G52740	histone H2A protein 9
	AT3G54560	histone H2A 11
Poplar	POPTR_0002s04720	HTA11; DNA binding
	POPTR_0005s23810	HTA11; DNA binding
	POPTR_0006s26540	HTA9 (HISTONE H2A PROTEIN 9); DNA binding
	POPTR_0006s26550	HTA9 (HISTONE H2A PROTEIN 9); DNA binding
	POPTR_0018s01310	HTA9 (HISTONE H2A PROTEIN 9); DNA binding
	POPTR_0018s01320	HTA9 (HISTONE H2A PROTEIN 9); DNA binding
Grapevine	GSVIVG00003139001	Histone H2A
	GSVIVG00017218001	Histone H2A
	GSVIVG00035768001	Histone H2A
Sorghum	Sb04g025140	Histone H2A
Maize	GRMZM2G050833	Histone H2A
	GRMZM2G056231	Histone H2A
	GRMZM2G149775	Histone H2A
Brachypodium	Bradi1g09060	histone H2A variant 3
	Bradi3g26880	histone H2A variant 2

Species	Orthologous genes	Putative function
Rice	LOC_Os03g05980	40S ribosomal protein S9-2, putative, expressed
	LOC_Os11g38959	40S ribosomal protein S9-2, putative, expressed
Arabidopsis	AT5G39850	Ribosomal protein S4
Poplar	POPTR_0006s22630	40S ribosomal protein S9 (RPS9C)
	POPTR_0007s09750	40S ribosomal protein S9 (RPS9C)
	POPTR_0011s09670	40S ribosomal protein S9 (RPS9C)
	POPTR_0016s07730	40S ribosomal protein S9 (RPS9C)
	POPTR_0016s07770	40S ribosomal protein S9 (RPS9C)
	POPTR_0018s07520	40S ribosomal protein S9 (RPS9C)
Grapevine	GSVIVG00008028001	40S ribosomal protein S9
	GSVIVG00011384001	40S ribosomal protein S9
	GSVIVG00020020001	40S ribosomal protein S9
Sorghum	Sb02g040080	40S ribosomal protein S9
	Sb02g040120	40S ribosomal protein S9
	Sb08g015270	40S ribosomal protein S9
Maize	GRMZM2G108348	40S ribosomal protein S9
	GRMZM5G832108	40S ribosomal protein S9

Brachypodium	Bradi1g20580	40S ribosomal protein S9
	Bradi1g61090	40S ribosomal protein S9
	Bradi4g07120	40S ribosomal protein S9
	Bradi4g14160	40S ribosomal protein S9

**Species**	**Orthologous genes**	**Putative function**
Rice	LOC_Os03g05730	cell division control protein 48 homolog E, putative, expressed
	LOC_Os10g30580	cell division control protein 48 homolog E, putative, expressed
Arabidopsis	AT3G09840	cell division cycle 48
	AT3G53230	"ATPase, AAA-type, CDC48 protein"
	AT5G03340	"ATPase, AAA-type, CDC48 protein"
Poplar	POPTR_0006s12740	cell division cycle protein 48, putative / CDC48, putative
	POPTR_0012s09000	cell division cycle protein 48, putative / CDC48, putative
	POPTR_0015s09220	cell division cycle protein 48, putative / CDC48, putative
	POPTR_0016s09280	cell division cycle protein 48, putative / CDC48, putative
	POPTR_0017s14340	cell division cycle protein 48, putative / CDC48, putative
Grapevine	GSVIVG00007689001	Cell division cycle protein 48 homolog
	GSVIVG00025723001	Transitional endoplasmic reticulum ATPase
	GSVIVG00031517001	similar to Cell division cycle protein 48 homolog
Sorghum	Sb01g020910	Transitional endoplasmic reticulum ATPase
	Sb01g046840	Transitional endoplasmic reticulum ATPase
	Sb01g047410	Cell division cycle protein 48 homolog
	Sb01g047440	Cell division cycle protein 48 homolog
Maize	AC233949.1_FG004	Cell division cycle protein 48
	GRMZM2G036765	Cell division cycle protein 48
	GRMZM2G063060	Cell division cycle protein 48
Brachypodium	Bradi1g74920	Transitional endoplasmic reticulum ATPase
	Bradi1g75570	Cell division cycle protein 48 homolog

**Species**	**Orthologous genes**	**Putative function**
Rice	LOC_Os03g05720	WD domain, G-beta repeat domain containing protein, expressed
Arabidopsis	AT3G21540	transducin family protein / WD-40 repeat family protein
Poplar	POPTR_0006s12290	transducin family protein / WD-40 repeat family protein
Grapevine	GSVIVG00033690001	WD-repeat protein
Sorghum	Sb01g046850	WD-repeat protein
Maize	AC209858.4_FG002	WD-repeat protein
Brachypodium	Bradi1g74940	WD-repeat protein

**Species**	**Orthologous genes**	**Putative function**
Rice	LOC_Os03g01970	THO complex subunit 1, putative, expressed
Arabidopsis	AT5G09860	nuclear matrix protein-related
Poplar	POPTR_0001s06900	nuclear matrix protein-related

Additional File 2 cont.: Orthologous Proteins from Different Plant Species

	POPTR_0003s19340	nuclear matrix protein-related
Grapevine	GSVIVG00036611001	Nuclear matrix protein
	GSVIVG00036631001	Nuclear matrix protein
Sorghum	Sb01g049910	Nuclear matrix protein
Maize	GRMZM2G006673	Nuclear matrix protein
Brachypodium	Bradi1g77890	Nuclear matrix protein

**Species**	**Orthologous genes**	**Putative function**
Rice	LOC_Os03g01530	tubulin/FtsZ domain containing protein, putative, expressed
Arabidopsis	AT5G23860	tubulin beta 8
Poplar	POPTR_0001s09180	TUB8; structural constituent of cytoskeleton
	POPTR_0001s25410	TUB2; GTP binding / GTPase/ structural molecule
	POPTR_0003s12540	TUB8; structural constituent of cytoskeleton
	POPTR_0006s09610	TUB8; structural constituent of cytoskeleton
	POPTR_0009s04500	TUB8; structural constituent of cytoskeleton
Grapevine	GSVIVG00028941001	Tubulin beta-1 chain
Sorghum	Sb01g050310	Tubulin beta-1 chain
Maize	GRMZM2G164696	Tubulin beta-1 chain
Brachypodium	Bradi1g78210	Tubulin beta-2 chain

**Species**	**Orthologous genes**	**Putative function**
Rice	LOC_Os02g57590	rRNA 2-O-methyltransferase fibrillarin 2, putative, expressed
	LOC_Os05g08360	rRNA 2-O-methyltransferase fibrillarin 2, putative, expressed
Arabidopsis	AT4G25630	fibrillarin 2
	AT5G52470	fibrillarin 1
Poplar	POPTR_0012s12860	FIB2 (FIBRILLARIN 2); snoRNA binding
	POPTR_0015s12790	FIB1 (FIBRILLARIN 1); snoRNA binding
Grapevine	GSVIVG00028887001	fibrillarin homolog
Sorghum	Sb04g037640	Fibrillarin-2
Maize	GRMZM2G363678	Fibrillarin-2
Brachypodium	Bradi1g32010	Fibrillarin-2
	Bradi3g55810	Fibrillarin-2

**Species**	**Orthologous genes**	**Putative function**
Rice	LOC_Os02g56960	ribosomal protein, putative, expressed
	LOC_Os03g04590	ribosomal protein, putative, expressed
	LOC_Os10g32920	ribosomal protein, putative, expressed
Arabidopsis	AT1G04480	Ribosomal protein L14p/L23e family protein
	AT2G33370	Ribosomal protein L14p/L23e family protein
	AT3G04400	Ribosomal protein L14p/L23e family protein
Poplar	POPTR_0002s25930	60S ribosomal protein L23 (RPL23A)
	POPTR_0008s17110	60S ribosomal protein L23 (RPL23B)

	POPTR_0010s07690	emb2171 (embryo defective 2171); structural constituent of ribosome
	POPTR_1243s00200	60S ribosomal protein L23 (RPL23B)
Grapevine	GSVIVG00027905001	60S ribosomal protein L23
	GSVIVG00028407001	60S ribosomal protein L23
Sorghum	Sb01g019770	60S ribosomal protein L23
	Sb01g047680	60S ribosomal protein L23
	Sb04g036970	60S ribosomal protein L23
Maize	GRMZM2G078396	60S ribosomal protein L23
	GRMZM2G102230	60S ribosomal protein L23
	GRMZM2G150058	60S ribosomal protein L23
	GRMZM2G171181	60S ribosomal protein L23
Brachypodium	Bradi3g28300	60S ribosomal protein L23
	Bradi3g55140	60S ribosomal protein L23

**Species**	**Orthologous genes**	**Putative function**
Rice	LOC_Os02g56014	40S ribosomal protein S30, putative, expressed
	LOC_Os06g07580	expressed protein
Arabidopsis	AT2G19750	Ribosomal protein S30 family protein
	AT4G29390	Ribosomal protein S30 family protein
	AT5G56670	Ribosomal protein S30 family protein
Poplar	POPTR_0012s08850	40S ribosomal protein S30 (RPS30B)
	POPTR_0015s09620	40S ribosomal protein S30 (RPS30C)
Grapevine	GSVIVG00016902001	40S ribosomal protein S30
Sorghum	Sb04g036360	40S ribosomal protein S30
	Sb10g004940	40S ribosomal protein S30
Maize	GRMZM2G096690	40S ribosomal protein S30
	GRMZM5G805526	40S ribosomal protein S30
Brachypodium	Bradi1g48060	40S ribosomal protein S30

**Species**	**Orthologous genes**	**Putative function**
Rice	LOC_Os02g55010	expressed protein
	LOC_Os11g07470	expressed protein
Arabidopsis	AT3G01780	ARM repeat superfamily protein
Poplar	POPTR_0001s34190	TPLATE; binding
Grapevine	GSVIVG00001825001	Armadillo-like helical domain-containing protein
Sorghum	Sb05g004840	Armadillo-like helical domain-containing protein
Maize	GRMZM2G162286	Conserved gene of unknown function
	GRMZM2G472770	Conserved gene of unknown function
Brachypodium	Bradi4g24200	Armadillo-like helical domain-containing protein

**Species**	**Orthologous genes**	**Putative function**
Rice	LOC_Os02g54340	26S protease regulatory subunit 7, putative, expressed

Additional File 2 cont.: Orthologous Proteins from Different Plant Species

Arabidopsis	AT1G53750	regulatory particle triple-A 1A
Poplar	POPTR_0001s16180	RPT1A (REGULATORY PARTICLE TRIPLE-A 1A); ATPase
	POPTR_0006s23310	RPT1A (REGULATORY PARTICLE TRIPLE-A 1A); ATPase
	POPTR_0018s04900	RPT1A (REGULATORY PARTICLE TRIPLE-A 1A); ATPase
	POPTR_0563s00200	RPT1A (REGULATORY PARTICLE TRIPLE-A 1A); ATPase
Grapevine	GSVIVG00016655001	26S protease regulatory subunit 7
Sorghum	Sb03g030500	26S protease regulatory subunit 7
	Sb04g035230	26S protease regulatory subunit 7
Maize	GRMZM2G110185	26S protease regulatory subunit 7
	GRMZM2G181359	26S protease regulatory subunit 7
Brachypodium	Bradi1g46760	26S protease regulatory subunit 7

**Species**	**Orthologous genes**	**Putative function**
Rice	LOC_Os02g52950	expressed protein
Sorghum	Sb04g034360	Conserved gene of unknown function
	Sb04g034370	Conserved gene of unknown function
Maize	GRMZM2G174240	DUF573 domain containing protein
Brachypodium	Bradi3g57800	storekeeper protein

**Species**	**Orthologous genes**	**Putative function**
Rice	LOC_Os02g52250	SKIP/SNW domain containing protein, expressed
Arabidopsis	AT1G77180	chromatin protein family
Poplar	POPTR_0018s09310	chromatin protein family
Grapevine	GSVIVG00015321001	Nuclear protein skip
Sorghum	Sb04g033860	Pre-mRNA-splicing factor prp45
Maize	GRMZM2G478709	Pre-mRNA-splicing factor prp45
Brachypodium	Bradi3g58310	Pre-mRNA-splicing factor prp45

**Species**	**Orthologous genes**	**Putative function**
Rice	LOC_Os02g50880	OsDegp3 - Putative Deg protease homologue, expressed
Arabidopsis	AT5G40200	DegP protease 9
Poplar	POPTR_0012s07930	DegP9 (DegP protease 9); catalytic/ protein binding / serine-type endopeptidase/ serine-type peptidase
	POPTR_0015s08440	DegP9 (DegP protease 9); catalytic/ protein binding / serine-type endopeptidase/ serine-type peptidase
Grapevine	GSVIVG00007803001	Serine endopeptidase degp2
	GSVIVG00032512001	Serine endopeptidase degp2
Sorghum	Sb04g028270	DegP2 protease
Maize	AC212112.4_FG002	DegP2 protease
Brachypodium	Bradi3g59540	DegP2 protease

**Species**	**Orthologous genes**	**Putative function**
Rice	LOC_Os02g49270	NOL1/NOP2/sun family protein, putative, expressed
	LOC_Os09g37860	NOL1/NOP2/sun family protein, putative, expressed

Additional File 2 cont.: Orthologous Proteins from Different Plant Species

Arabidopsis	AT4G26600	S-adenosyl-L-methionine-dependent methyltransferases superfamily protein
	AT5G55920	S-adenosyl-L-methionine-dependent methyltransferases superfamily protein
Poplar	POPTR_0001s37830	nucleolar protein, putative
Grapevine	GSVIVG00014717001	Proliferating-cell nucleolar antigen p120
Sorghum	Sb04g029465	proliferating cell nuclear protein P120
Maize	GRMZM2G005256	proliferating cell nuclear protein P120
Brachypodium	Bradi3g56470	proliferating cell nuclear protein P120

Species	Orthologous genes	Putative function
Rice	LOC_Os11g38900	histone-lysine N-methyltransferase, H3 lysine-9 specific SUVH1, putative, expressed
Arabidopsis	AT5G04940	SU(VAR)3-9 homolog 1
Poplar	POPTR_0001s07390	SUVH3 (SU(VAR)3-9 HOMOLOG 3); histone methyltransferase
	POPTR_0003s18740	SUVH3 (SU(VAR)3-9 HOMOLOG 3); histone methyltransferase
Sorghum	Sb02g006620	SET1
	Sb06g001340	SET1
Maize	AC233961.1_FG001	SET1
Brachypodium	Bradi1g53840	SET1

Species	Orthologous genes	Putative function
Rice	LOC_Os02g47140	L11 domain containing ribosomal protein, putative, expressed
	LOC_Os04g50990	L11 domain containing ribosomal protein, putative, expressed
Arabidopsis	AT2G37190	Ribosomal protein L11 family protein
	AT3G53430	Ribosomal protein L11 family protein
	AT5G60670	Ribosomal protein L11 family protein
Poplar	POPTR_0006s07680	60S ribosomal protein L12 (RPL12B)
	POPTR_0018s14210	60S ribosomal protein L12 (RPL12B)
	POPTR_0018s14220	60S ribosomal protein L12 (RPL12B)
	POPTR_0516s00210	60S ribosomal protein L12 (RPL12B)
Grapevine	GSVIVG00025683001	60S ribosomal protein L12
Sorghum	Sb04g030890	60S ribosomal protein L12
	Sb06g014530	60S ribosomal protein L12
	Sb06g027330	60S ribosomal protein L12
Maize	AC196489.3_FG002	60S ribosomal protein L12
	GRMZM2G121075	60S ribosomal protein L12
	GRMZM2G149649	60S ribosomal protein L12
Brachypodium	Bradi3g52460	60S ribosomal protein L12
	Bradi5g20330	60S ribosomal protein L12

Species	Orthologous genes	Putative function
Rice	LOC_Os02g43930	chaperone protein dnaJ, putative, expressed
Poplar	POPTR_0002s14200	ATJ2; protein binding
Grapevine	GSVIVG00018226001	DnaJ homolog protein

Sorghum	Sb04g032970	DnaJ family heat shock protein
Maize	GRMZM2G118731	DnaJ family heat shock protein
Brachypodium	Bradi3g50610	DnaJ family heat shock protein

**Species**	**Orthologous genes**	**Putative function**
Rice	LOC_Os02g40514	h/ACA ribonucleoprotein complex subunit 3, putative, expressed
Arabidopsis	AT2G20490	nucleolar RNA-binding Nop10p family protein
Poplar	POPTR_0002s03780	NOP10; RNA binding
Grapevine	GSVIVG00033693001	Nucleolar RNA-binding Nop10p
Sorghum	Sb04g026200	Nucleolar RNA-binding Nop10p
	Sb06g029530	Nucleolar RNA-binding Nop10p
Maize	GRMZM2G153227	H/ACA ribonucleoprotein complex subunit 3

**Species**	**Orthologous genes**	**Putative function**
Rice	LOC_Os02g39140	helix-loop-helix DNA-binding domain containing protein, expressed
Arabidopsis	AT2G43140	basic helix-loop-helix (bHLH) DNA-binding superfamily protein
Sorghum	Sb04g025260	Helix-loop-helix DNA-binding domain containing protein
Maize	GRMZM2G137426	Helix-loop-helix DNA-binding domain containing protein
Brachypodium	Bradi3g48070	Transcription factor bHLH128

**Species**	**Orthologous genes**	**Putative function**
Rice	LOC_Os02g38210	elongation factor Tu, putative, expressed
Arabidopsis	AT4G20360	RAB GTPase homolog E1B
Poplar	POPTR_0001s08770	ATRABE1B (ARABIDOPSIS RAB GTPASE HOMOLOG E1B); GTP binding / GTPase/ translation elongation factor
	POPTR_0003s12160	ATRABE1B (ARABIDOPSIS RAB GTPASE HOMOLOG E1B); GTP binding / GTPase/ translation elongation factor
Sorghum	Sb04g024850	Elongation factor Tu
Maize	GRMZM2G106061	Elongation factor Tu
	GRMZM2G407996	Elongation factor Tu
Brachypodium	Bradi3g47690	Elongation factor Tu

**Species**	**Orthologous genes**	**Putative function**
Rice	LOC_Os02g37862	60S ribosomal protein L6, putative, expressed
	LOC_Os04g39700	60S ribosomal protein L6, putative, expressed
Arabidopsis	AT1G18540	Ribosomal protein L6 family protein
	AT1G74050	Ribosomal protein L6 family protein
	AT1G74060	Ribosomal protein L6 family protein
Poplar	POPTR_0001s27830	60S ribosomal protein L6 (RPL6C)
	POPTR_0009s07020	60S ribosomal protein L6 (RPL6C)
Grapevine	GSVIVG00032041001	60S ribosomal protein L6
	GSVIVG00033532001	60S ribosomal protein L6
Sorghum	Sb04g024500	60S ribosomal protein L6
	Sb06g019800	60S ribosomal protein L6

Maize	GRMZM2G003384	60S ribosomal protein L6
	GRMZM2G008748	60S ribosomal protein L6
	GRMZM2G024647	60S ribosomal protein L6
Brachypodium	Bradi3g08900	60S ribosomal protein L6
	Bradi3g47410	60S ribosomal protein L6

**Species**	**Orthologous genes**	**Putative function**
Rice	LOC_Os02g37430	LSM domain containing protein, expressed
	LOC_Os04g39444	LSM domain containing protein, expressed
Arabidopsis	AT3G07590	Small nuclear ribonucleoprotein family protein
Poplar	POPTR_0002s05570	small nuclear ribonucleoprotein D1, putative / snRNP core protein D1, putative / Sm protein D1, putative
	POPTR_0005s22940	small nuclear ribonucleoprotein D1, putative / snRNP core protein D1, putative / Sm protein D1, putative
Grapevine	GSVIVG00011117001	Small nuclear ribonucleoprotein sm d1
Sorghum	Sb04g024330	Small nuclear ribonucleoprotein Sm D1
	Sb06g019630	Small nuclear ribonucleoprotein Sm D1
Maize	GRMZM2G027571	Small nuclear ribonucleoprotein Sm D1
	GRMZM2G416061	Small nuclear ribonucleoprotein Sm D1
Brachypodium	Bradi3g47250	Small nuclear ribonucleoprotein Sm D1
	Bradi5g13020	Small nuclear ribonucleoprotein Sm D1

**Species**	**Orthologous genes**	**Putative function**
Rice	LOC_Os02g33140	ribosomal protein, putative, expressed
Arabidopsis	AT2G36160	Ribosomal protein S11 family protein
	AT3G11510	Ribosomal protein S11 family protein
	AT3G52580	Ribosomal protein S11 family protein
Poplar	POPTR_0001s22620	40S ribosomal protein S14 (RPS14C)
	POPTR_0004s13690	40S ribosomal protein S14 (RPS14C)
	POPTR_0009s02450	40S ribosomal protein S14 (RPS14C)
	POPTR_0011s02660	40S ribosomal protein S14 (RPS14C)
Grapevine	GSVIVG00033877001	Ribosomal protein S14
	GSVIVG00036096001	Ribosomal protein S14
Sorghum	Sb06g018900	40S ribosomal protein S14
	Sb10g025270	40S ribosomal protein S14
	Sb10g025340	40S ribosomal protein S14
Maize	GRMZM2G069762	40S ribosomal protein S14
	GRMZM2G094074	40S ribosomal protein S14
Brachypodium	Bradi3g04790	Ribosomal protein S14
	Bradi5g09460	40S ribosomal protein S14

**Species**	**Orthologous genes**	**Putative function**
Rice	LOC_Os01g52470	elongation factor, putative, expressed
	LOC_Os01g53900	elongation factor, putative, expressed

Additional File 2 cont.: Orthologous Proteins from Different Plant Species

	LOC_Os02g32030	elongation factor, putative, expressed
	LOC_Os04g02820	elongation factor, putative, expressed
Arabidopsis	AT1G56070	Ribosomal protein S5/Elongation factor G/III/V family protein
	AT3G12915	Ribosomal protein S5/Elongation factor G/III/V family protein
Poplar	POPTR_0005s10090	LOS1; copper ion binding / translation elongation factor/ translation factor, nucleic acid binding
	POPTR_0007s08390	LOS1; copper ion binding / translation elongation factor/ translation factor, nucleic acid binding
	POPTR_0007s08400	LOS1; copper ion binding / translation elongation factor/ translation factor, nucleic acid binding
Sorghum	Sb01g002040	Elongation factor 2
	Sb03g033210	Elongation factor 2
	Sb03g034200	Elongation factor 2
Maize	AC203173.3_FG004	Elongation factor 2
	GRMZM2G040369	Elongation factor 2
	GRMZM2G095851	Elongation factor 2
	GRMZM2G113250	Elongation factor 2
Brachypodium	Bradi2g45070	Elongation factor 2
	Bradi3g44160	Elongation factor 2
	Bradi3g44480	Elongation factor 2

**Species**	**Orthologous genes**	**Putative function**
Rice	LOC_Os02g28810	ribosomal protein, putative, expressed
	LOC_Os04g28180	ribosomal protein, putative, expressed
Arabidopsis	AT5G20290	Ribosomal protein S8e family protein
	AT5G59240	Ribosomal protein S8e family protein
Poplar	POPTR_0001s26950	40S ribosomal protein S8 (RPS8B)
	POPTR_0009s06180	40S ribosomal protein S8 (RPS8B)
	POPTR_0017s14180	40S ribosomal protein S8 (RPS8B)
Grapevine	GSVIVG0003156600 1	Ribosomal protein S8
Sorghum	Sb04g028530	Ribosomal protein S8
	Sb06g004770	Ribosomal protein S8
Maize	GRMZM2G030228	40S ribosomal protein S8
	GRMZM2G051848	40S ribosomal protein S8
	GRMZM2G063700	40S ribosomal protein S8
	GRMZM2G336875	40S ribosomal protein S8
	GRMZM2G360677	40S ribosomal protein S8
Brachypodium	Bradi1g51360	Ribosomal protein S8
	Bradi3g43560	Ribosomal protein S8

**Species**	**Orthologous genes**	**Putative function**
Rice	LOC_Os01g64090	L1P family of ribosomal proteins domain containing protein, expressed
	LOC_Os02g21660	L1P family of ribosomal proteins domain containing protein, expressed
	LOC_Os08g44380	L1P family of ribosomal proteins domain containing protein, expressed
Arabidopsis	AT1G08360	Ribosomal protein L1p/L10e family
	AT2G27530	Ribosomal protein L1p/L10e family

	AT5G22440	Ribosomal protein L1p/L10e family
Poplar	POPTR_0004s21290	60S ribosomal protein L10A (RPL10aA)
	POPTR_0004s21300	PGY1 (PIGGYBACK1); RNA binding / structural constituent of ribosome
	POPTR_0007s11880	PGY1 (PIGGYBACK1); RNA binding / structural constituent of ribosome
	POPTR_0007s11960	PGY1 (PIGGYBACK1); RNA binding / structural constituent of ribosome
Grapevine	GSVIVG00033841001	Ribosomal protein L1
	GSVIVG00036128001	similar to ribosomal protein L10a
Sorghum	Sb03g040550	Ribosomal protein L1
	Sb07g024200	Ribosomal protein L1
	Sb07g024210	Ribosomal protein L1
Maize	GRMZM2G023748	Ribosomal protein
	GRMZM2G144387	Ribosomal protein
Brachypodium	Bradi4g38510	Ribosomal protein L1

Species	Orthologous genes	Putative function
Rice	LOC_Os02g18660	expressed protein
Sorghum	Sb04g011230	storekeeper protein
Maize	GRMZM2G021015	Conserved gene of unknown function
	GRMZM2G125239	Dere/GG16231-like protein
Brachypodium	Bradi3g20610	storekeeper protein

Species	Orthologous genes	Putative function
Rice	LOC_Os02g18550	40S ribosomal protein S3a, putative, expressed
	LOC_Os03g10340	40S ribosomal protein S3a, putative, expressed
	LOC_Os12g21798	40S ribosomal protein S3a, putative, expressed
Arabidopsis	AT3G04840	Ribosomal protein S3Ae
	AT4G34670	Ribosomal protein S3Ae
Poplar	POPTR_0005s05280	40S ribosomal protein S3A (RPS3aB)
	POPTR_0008s15610	40S ribosomal protein S3A (RPS3aB)
	POPTR_0010s09330	40S ribosomal protein S3A (RPS3aB)
Grapevine	GSVIVG00018024001	40S ribosomal protein S3a-1
Sorghum	Sb02g038365	40S ribosomal protein S3a
	Sb02g038370	40S ribosomal protein S3a
Maize	GRMZM2G030915	40S ribosomal protein S3a
	GRMZM2G145258	40S ribosomal protein S3a
Brachypodium	Bradi1g71200	40S ribosomal protein S3a
	Bradi1g78170	40S ribosomal protein S3a
	Bradi3g01210	40S ribosomal protein S3a

Species	Orthologous genes	Putative function
Rice	LOC_Os02g18380	60S ribosomal protein L27-3, putative, expressed
	LOC_Os10g41470	60S ribosomal protein L27-3, putative, expressed
Arabidopsis	AT3G22230	Ribosomal L27e protein family

	AT4G15000	Ribosomal L27e protein family
Poplar	POPTR_0001s35630	60S ribosomal protein L27 (RPL27C)
	POPTR_0006s02230	60S ribosomal protein L27 (RPL27C)
	POPTR_0016s02040	60S ribosomal protein L27 (RPL27C)
Grapevine	GSVIVG00012080001	60S ribosomal protein L27
	GSVIVG00020410001	60S ribosomal protein L27
Sorghum	Sb06g029070	60S ribosomal protein L27
	Sb10g027330	60S ribosomal protein L27
Maize	GRMZM2G016250	60S ribosomal protein L27
	GRMZM2G302712	60S ribosomal protein L27
	GRMZM2G326066	60S ribosomal protein L27
	GRMZM2G366077	60S ribosomal protein L27
Brachypodium	Bradi1g30210	60S ribosomal protein L27

Species	Orthologous genes	Putative function
Rice	LOC_Os02g16640	proline-rich protein HaeIII subfamily 1 precursor, putative, expressed
Arabidopsis	AT4G03120	C2H2 and C2HC zinc fingers superfamily protein
Poplar	POPTR_0003s05710	proline-rich family protein
Grapevine	GSVIVG00011011001	similar to putative C-type U1 snRNP
Sorghum	Sb04g009880	similar to putative C-type U1 snRNP
	Sb04g028260	similar to putative C-type U1 snRNP
Maize	GRMZM2G413193	similar to putative C-type U1 snRNP
Brachypodium	Bradi3g09870	similar to putative C-type U1 snRNP

Species	Orthologous genes	Putative function
Rice	LOC_Os02g13990	U2 small nuclear ribonucleoprotein A, putative, expressed
Arabidopsis	AT1G09760	U2 small nuclear ribonucleoprotein A
Poplar	POPTR_0001s12570	U2A (U2 small nuclear ribonucleoprotein A); protein binding
	POPTR_0381s00210	U2A (U2 small nuclear ribonucleoprotein A); protein binding
Grapevine	GSVIVG00001138001	U2 small nuclear ribonucleoprotein A
Sorghum	Sb01g001040	U2 small nuclear ribonucleoprotein A
Maize	GRMZM2G021742	U2 small nuclear ribonucleoprotein A
Brachypodium	Bradi1g01530	U2 small nuclear ribonucleoprotein A

Species	Orthologous genes	Putative function
Rice	LOC_Os01g52490	40S ribosomal protein S24, putative, expressed
	LOC_Os02g13530	40S ribosomal protein S24, putative, expressed
	LOC_Os06g36160	40S ribosomal protein S24, putative, expressed
Arabidopsis	AT3G04920	Ribosomal protein S24e family protein
	AT5G28060	Ribosomal protein S24e family protein
Poplar	POPTR_0005s05120	40S ribosomal protein S24 (RPS24B)
	POPTR_0008s15190	40S ribosomal protein S24 (RPS24B)

Additional File 2 cont.: Orthologous Proteins from Different Plant Species

	POPTR_0010s09820	40S ribosomal protein S24 (RPS24B)
	POPTR_0030s00470	40S ribosomal protein S24 (RPS24B)
Grapevine	GSVIVG00001167001	40S ribosomal protein S24
Sorghum	Sb03g033230	40S ribosomal protein S24
	Sb04g008130	40S ribosomal protein S24
	Sb10g021950	40S ribosomal protein S24
Maize	GRMZM2G029685	40S ribosomal protein S24
	GRMZM2G091383	40S ribosomal protein S24
	GRMZM2G171426	40S ribosomal protein S24
Brachypodium	Bradi1g38010	40S ribosomal protein S24
	Bradi3g08680	40S ribosomal protein S24

**Species**	**Orthologous genes**	**Putative function**
Rice	LOC_Os02g10640	26S protease regulatory subunit, putative, expressed
	LOC_Os06g40560	26S protease regulatory subunit S10B, putative, expressed
Arabidopsis	AT1G45000	AAA-type ATPase family protein
	AT5G43010	regulatory particle triple-A ATPase 4A
Poplar	POPTR_0002s03280	26S proteasome regulatory complex subunit p42D, putative
	POPTR_0004s16600	26S proteasome regulatory complex subunit p42D, putative
	POPTR_0005s25320	26S proteasome regulatory complex subunit p42D, putative
	POPTR_0009s12330	RPT4A; ATPase
Grapevine	GSVIVG00009774001	26S protease regulatory subunit S10b
	GSVIVG00023828001	26S protease regulatory subunit S10b
Sorghum	Sb04g006830	26S protease regulatory subunit S10B
Maize	GRMZM2G165817	26S protease regulatory subunit S10B
Brachypodium	Bradi1g36400	26S protease regulatory subunit S10B
	Bradi3g07370	26S protease regulatory subunit S10B

**Species**	**Orthologous genes**	**Putative function**
Rice	LOC_Os02g10080	zinc finger C-x8-C-x5-C-x3-H type family protein, expressed
	LOC_Os06g41384	zinc finger C-x8-C-x5-C-x3-H type family protein, expressed
Arabidopsis	AT3G12130	KH domain-containing protein / zinc finger (CCCH type) family protein
	AT5G06770	KH domain-containing protein / zinc finger (CCCH type) family protein
Poplar	POPTR_0001s05070	KH domain-containing protein / zinc finger (CCCH type) family protein
	POPTR_0003s21780	KH domain-containing protein / zinc finger (CCCH type) family protein
	POPTR_0016s04590	KH domain-containing protein / zinc finger (CCCH type) family protein
	POPTR_0016s04690	KH domain-containing protein / zinc finger (CCCH type) family protein
Grapevine	GSVIVG00001369001	Zinc finger CCCH domain-containing protein 52
	GSVIVG00025360001	Zinc finger CCCH domain-containing protein 36
	GSVIVG00033508001	Zinc finger CCCH domain-containing protein 36
Sorghum	Sb04g006450	Zinc finger CCCH domain-containing protein 14
	Sb10g024330	Zinc finger C-x8-C-x5-C-x3-H type family protein

Additional File 2 cont.: Orthologous Proteins from Different Plant Species

Maize	GRMZM2G056920	Zinc finger CCCH domain-containing protein 44
	GRMZM2G110402	Zinc finger C-x8-C-x5-C-x3-H type family protein
	GRMZM2G151689	Zinc finger C-x8-C-x5-C-x3-H type family protein
Brachypodium	Bradi1g35920	Zinc finger C-x8-C-x5-C-x3-H type family protein
	Bradi3g06940	Zinc finger CCCH domain-containing protein 14

**Species**	**Orthologous genes**	**Putative function**
Rice	LOC_Os02g08370	ubiquitin carboxyl-terminal hydrolase, family 1, putative, expressed
Sorghum	Sb01g042110	Ubiquitin carboxyl-terminal hydrolase isozyme L5
	Sb07g023880	Ubiquitin carboxyl-terminal hydrolase isozyme L5
Maize	GRMZM2G109977	Ubiquitin carboxyl-terminal hydrolase isozyme L5
Brachypodium	Bradi3g06000	Ubiquitin carboxyl-terminal hydrolase isozyme L5

**Species**	**Orthologous genes**	**Putative function**
Rice	LOC_Os02g07890	60S ribosomal protein L27a-3, putative, expressed
	LOC_Os03g29460	60S ribosomal protein, putative, expressed
Arabidopsis	AT1G23290	Ribosomal protein L18e/L15 superfamily protein
	AT1G70600	Ribosomal protein L18e/L15 superfamily protein
Poplar	POPTR_0006s21680	structural constituent of ribosome
	POPTR_0008s19110	structural constituent of ribosome
	POPTR_0010s05590	structural constituent of ribosome
	POPTR_0016s06950	structural constituent of ribosome
Grapevine	GSVIVG00007667001	60S ribosomal protein L27a-3
	GSVIVG00032405001	60S ribosomal protein L27A
Sorghum	Sb02g039090	60S ribosomal protein L27a-3
Maize	GRMZM2G030731	60S ribosomal protein L27a-3
Brachypodium	Bradi1g21630	60S ribosomal protein L27a-3
	Bradi1g60160	60S ribosomal protein L27a-2

**Species**	**Orthologous genes**	**Putative function**
Rice	LOC_Os02g07260	phosphoglycerate kinase protein, putative, expressed
	LOC_Os06g45710	phosphoglycerate kinase protein, putative, expressed
Arabidopsis	AT1G79550	phosphoglycerate kinase
Poplar	POPTR_0008s08400	PGK (PHOSPHOGLYCERATE KINASE); phosphoglycerate kinase
	POPTR_0010s17870	PGK (PHOSPHOGLYCERATE KINASE); phosphoglycerate kinase
Sorghum	Sb04g004690	Phosphoglycerate kinase
	Sb10g026710	Phosphoglycerate kinase
Maize	GRMZM2G382914	Phosphoglycerate kinase
Brachypodium	Bradi3g05220	Phosphoglycerate kinase, cytosolic

**Species**	**Orthologous genes**	**Putative function**
Rice	LOC_Os02g06584	zinc finger C-x8-C-x5-C-x3-H type family protein, expressed
Arabidopsis	AT3G21810	Zinc finger C-x8-C-x5-C-x3-H type family protein

Additional File 2 cont.: Orthologous Proteins from Different Plant Species

Poplar	POPTR_0007s02620	zinc finger (CCCH-type) family protein
Grapevine	GSVIVG00030622001	Zinc finger C-x8-C-x5-C-x3-H type family protein
Sorghum	Sb04g004246	Zinc finger C-x8-C-x5-C-x3-H type family protein
Maize	GRMZM2G119640	Zinc finger C-x8-C-x5-C-x3-H type family protein
Brachypodium	Bradi3g04650	Zinc finger CCCH domain-containing protein 13

Species	Orthologous genes	Putative function
Rice	LOC_Os02g06370	whirly transcription factor domain containing protein, expressed
Arabidopsis	AT1G71260	WHIRLY 2
Poplar	POPTR_0003s04700	ATWHY2 (A. THALIANA WHIRLY 2); DNA binding
Grapevine	GSVIVG00037137001	DNA binding protein
Sorghum	Sb04g004060	DNA binding protein
Maize	GRMZM2G012262	DNA binding protein
Brachypodium	Bradi3g04450	DNA binding protein

Species	Orthologous genes	Putative function
Rice	LOC_Os02g04480	cleavage and polyadenylation specificity factor, putative, expressed
Arabidopsis	AT3G55200	Cleavage and polyadenylation specificity factor (CPSF) A subunit protein
	AT3G55220	Cleavage and polyadenylation specificity factor (CPSF) A subunit protein
Poplar	POPTR_0008s04890	splicing factor, putative
	POPTR_0010s21890	splicing factor, putative
Grapevine	GSVIVG00005970001	Spliceosomal protein sap
Sorghum	Sb04g003000	splicing factor 3b, subunit 3, 130kDa
Maize	GRMZM2G096972	splicing factor 3b, subunit 3, 130kDa
Brachypodium	Bradi3g03320	splicing factor 3b, subunit 3, 130kDa

Species	Orthologous genes	Putative function
Rice	LOC_Os02g04050	chromosome segregation protein, putative, expressed
	LOC_Os02g04080	chromosome segregation protein sudA, putative
Arabidopsis	AT2G27170	Structural maintenance of chromosomes (SMC) family protein
Poplar	POPTR_0009s15710	TTN7 (TITAN7); ATP binding / protein binding
Grapevine	GSVIVG00033181001	Condensin complex components subunit
Sorghum	Sb09g030220	SMC3 protein
Maize	GRMZM2G456570	SMC3 protein
Brachypodium	Bradi1g17900	SMC3 protein

Species	Orthologous genes	Putative function
Rice	LOC_Os02g04040	RecF/RecN/SMC N terminal domain containing protein, expressed
Sorghum	Sb09g030210	SMC3 protein

Species	Orthologous genes	Putative function
Rice	LOC_Os02g02410	DnaK family protein, putative, expressed

Additional File 2 cont.: Orthologous Proteins from Different Plant Species

Arabidopsis	AT5G28540	heat shock protein 70 (Hsp 70) family protein
	AT5G42020	Heat shock protein 70 (Hsp 70) family protein
Poplar	POPTR_0001s11010	BIP2; ATP binding
	POPTR_0003s14360	BIP2; ATP binding
	POPTR_0012s02470	BIP2; ATP binding
Grapevine	GSVIVG00019607001	Heat shock protein 70
	GSVIVG00038580001	luminal-binding protein
Sorghum	Sb04g001140	Luminal-binding protein 2
Maize	GRMZM2G114793	Luminal-binding protein 2
	GRMZM2G415007	Luminal-binding protein 3
Brachypodium	Bradi3g01480	Dnak-type molecular chaperone Bip

**Species**	**Orthologous genes**	**Putative function**
Rice	LOC_Os01g25610	40S ribosomal protein S4, putative, expressed
	LOC_Os02g01560	40S ribosomal protein S4, putative, expressed
Arabidopsis	AT2G17360	Ribosomal protein S4 (RPS4A) family protein
	AT5G07090	Ribosomal protein S4 (RPS4A) family protein
	AT5G58420	Ribosomal protein S4 (RPS4A) family protein
Poplar	POPTR_0006s10460	40S ribosomal protein S4 (RPS4B)
	POPTR_0013s01230	40S ribosomal protein S4 (RPS4B)
	POPTR_0015s05120	40S ribosomal protein S4 (RPS4B)
	POPTR_0015s09110	40S ribosomal protein S4 (RPS4B)
	POPTR_0016s13210	40S ribosomal protein S4 (RPS4D)
Grapevine	GSVIVG00024668001	similar to 40S ribosomal S4 protein
	GSVIVG00032756001	similar to 40S ribosomal S4 protein
Sorghum	Sb01g001850	40S ribosomal protein S4
	Sb03g014380	40S ribosomal protein S4
Maize	GRMZM2G125271	40S ribosomal protein S4
Brachypodium	Bradi2g35620	Ribosomal protein

**Species**	**Orthologous genes**	**Putative function**
Rice	LOC_Os02g01332	ribosomal protein L6, putative, expressed
	LOC_Os09g31180	ribosomal protein L6, putative, expressed
Arabidopsis	AT1G33120	Ribosomal protein L6 family
	AT1G33140	Ribosomal protein L6 family
	AT4G10450	Ribosomal protein L6 family
Poplar	POPTR_0001s45810	PGY2 (PIGGYBACK2); structural constituent of ribosome
	POPTR_0001s45820	PGY2 (PIGGYBACK2); structural constituent of ribosome
	POPTR_0011s15080	PGY2 (PIGGYBACK2); structural constituent of ribosome
	POPTR_0011s15170	PGY2 (PIGGYBACK2); structural constituent of ribosome
Grapevine	GSVIVG00016795001	60S ribosomal protein L9
	GSVIVG00038670001	60S ribosomal protein L9

Additional File 2 cont.: Orthologous Proteins from Different Plant Species

Sorghum	Sb10g000700	60S ribosomal protein L9
Maize	GRMZM2G084739	60S ribosomal protein L9
	GRMZM2G385287	60S ribosomal protein L9
Brachypodium	Bradi1g21850	60S ribosomal protein L9
	Bradi1g52040	60S ribosomal protein L9
	Bradi3g00640	60S ribosomal protein L9

**Species**	**Orthologous genes**	**Putative function**
Rice	LOC_Os02g01250	LSM domain containing protein, expressed
	LOC_Os03g13760	LSM domain containing protein, expressed
Arabidopsis	AT1G20580	Small nuclear ribonucleoprotein family protein
Poplar	POPTR_0002s01120	small nuclear ribonucleoprotein, putative / snRNP, putative / Sm protein, putative
	POPTR_0005s27250	small nuclear ribonucleoprotein, putative / snRNP, putative / Sm protein, putative
Grapevine	GSVIVG00013364001	Small nuclear ribonucleoprotein
	GSVIVG00023894001	Small nuclear ribonucleoprotein
Sorghum	Sb01g041430	Small nuclear ribonucleoprotein Sm D3
Maize	GRMZM2G115176	Small nuclear ribonucleoprotein Sm D3
	GRMZM2G115925	Small nuclear ribonucleoprotein Sm D3
	GRMZM5G884325	Small nuclear ribonucleoprotein Sm D3
Brachypodium	Bradi3g00420	Small nuclear ribonucleoprotein Sm D3

**Species**	**Orthologous genes**	**Putative function**
Rice	LOC_Os01g69970	WD domain, G-beta repeat domain containing protein, expressed
Arabidopsis	AT4G18900	Transducin/WD40 repeat-like superfamily protein
	AT4G18905	Transducin/WD40 repeat-like superfamily protein
Poplar	POPTR_0007s13400	transducin family protein / WD-40 repeat family protein
	POPTR_0337s00210	transducin family protein / WD-40 repeat family protein
Grapevine	GSVIVG00018880001	WD-repeat protein
Sorghum	Sb03g044310	Periodic tryptophan protein 1
Maize	GRMZM2G081013	Periodic tryptophan protein 1
Brachypodium	Bradi2g59230	Periodic tryptophan protein 1

**Species**	**Orthologous genes**	**Putative function**
Rice	LOC_Os01g68950	ubiquitin family domain containing protein, expressed
Arabidopsis	AT4G26840	small ubiquitin-like modifier 1
Poplar	POPTR_0002s21680	SUMO2 (SMALL UBIQUITIN-LIKE MODIFIER 2); protein binding / protein tag
	POPTR_0002s21690	SUMO2 (SMALL UBIQUITIN-LIKE MODIFIER 2); protein binding / protein tag
	POPTR_0014s15650	SUMO2 (SMALL UBIQUITIN-LIKE MODIFIER 2); protein binding / protein tag
	POPTR_0014s18990	SUMO2 (SMALL UBIQUITIN-LIKE MODIFIER 2); protein binding / protein tag
Grapevine	GSVIVG00003307001	Ubiquitin SMT3
	GSVIVG00030502001	SUMO protein
Sorghum	Sb03g043870	SUMO1b protein

Additional File 2 cont.: Orthologous Proteins from Different Plant Species

Maize	GRMZM2G053898	SUMO1b protein
	GRMZM2G082390	SUMO1b protein
Brachypodium	Bradi2g58830	Ubiquitin SMT3

**Species**	**Orthologous genes**	**Putative function**
Rice	LOC_Os01g67134	ribosomal L18p/L5e family protein, putative, expressed
Arabidopsis	AT3G25520	ribosomal protein L5
	AT5G39740	ribosomal protein L5 B
Poplar	POPTR_0014s17230	60S ribosomal protein L5 (RPL5B)
	POPTR_0019s13040	60S ribosomal protein L5 (RPL5B)
Grapevine	GSVIVG00038188001	Ribosomal protein L5
Sorghum	Sb01g048270	60S ribosomal protein L5-1
Maize	GRMZM2G090738	60S ribosomal protein L5-1
	GRMZM2G163081	60S ribosomal protein L5-1
	GRMZM5G815894	60S ribosomal protein L5-1
Brachypodium	Bradi1g13630	60S ribosomal protein L5-1
	Bradi4g13640	60S ribosomal protein L5-1
	Bradi5g09500	60S ribosomal protein L5-1

**Species**	**Orthologous genes**	**Putative function**
Rice	LOC_Os01g64640	histone H3, putative, expressed
	LOC_Os04g34240	histone H3, putative, expressed
	LOC_Os05g36280	histone H3, putative, expressed
	LOC_Os06g06460	histone H3, putative, expressed
	LOC_Os06g06510	histone H3, putative, expressed
	LOC_Os11g05730	histone H3, putative, expressed
Arabidopsis	AT1G09200	Histone superfamily protein
	AT3G27360	Histone superfamily protein
	AT5G10390	Histone superfamily protein
	AT5G10400	Histone superfamily protein
	AT5G65360	Histone superfamily protein
Poplar	POPTR_0001s05450	histone H3
	POPTR_0001s05470	histone H3
	POPTR_0002s03030	histone H3
	POPTR_0003s22120	histone H3
	POPTR_0003s22240	histone H3
	POPTR_0005s25530	histone H3
	POPTR_0014s09260	histone H3
Sorghum	Sb03g005550	Histone H3.2
	Sb04g022160	Histone H3.2
	Sb06g016330	Histone H3.2
	Sb06g016850	Histone H3.2
	Sb09g021650	Histone H3.2

Additional File 2 cont.: Orthologous Proteins from Different Plant Species

	Sb10g004100	Histone H3.2
	Sb10g004110	Histone H3.2
Maize	GRMZM2G130079	Histone H3.2
	GRMZM2G179005	Histone H3.2
	GRMZM2G355773	Histone H3.2
	GRMZM2G376957	Histone H3.2
	GRMZM2G401581	Histone H3.2
	GRMZM2G418258	Histone H3.2
	GRMZM2G447984	histone cluster 1, H3f
	GRMZM2G451254	Histone H3.2
	GRMZM2G475899	Histone H3
	GRMZM5G864735	Histone H3.2
Brachypodium	Bradi1g48660	Histone H3.2
	Bradi1g50820	Histone H3.2
	Bradi2g18410	Histone H3.2
	Bradi2g24080	Histone H3.2
	Bradi2g27720	Histone H3.2
	Bradi3g45290	Histone H3.2
	Bradi4g07840	Histone H3.2

**Species**	**Orthologous genes**	**Putative function**
Rice	LOC_Os01g64090	L1P family of ribosomal proteins domain containing protein, expressed
	LOC_Os02g21660	L1P family of ribosomal proteins domain containing protein, expressed
	LOC_Os08g44380	L1P family of ribosomal proteins domain containing protein, expressed
Arabidopsis	AT1G08360	Ribosomal protein L1p/L10e family
	AT2G27530	Ribosomal protein L1p/L10e family
	AT5G22440	Ribosomal protein L1p/L10e family
Poplar	POPTR_0004s21290	60S ribosomal protein L10A (RPL10aA)
	POPTR_0004s21300	PGY1 (PIGGYBACK1); RNA binding / structural constituent of ribosome
	POPTR_0007s11880	PGY1 (PIGGYBACK1); RNA binding / structural constituent of ribosome
	POPTR_0007s11960	PGY1 (PIGGYBACK1); RNA binding / structural constituent of ribosome
Grapevine	GSVIVG00033841001	Ribosomal protein L1
	GSVIVG00036128001	similar to ribosomal protein L10a
Sorghum	Sb03g040550	Ribosomal protein L1
	Sb07g024200	Ribosomal protein L1
	Sb07g024210	Ribosomal protein L1
Maize	GRMZM2G023748	Ribosomal protein
	GRMZM2G144387	Ribosomal protein
Brachypodium	Bradi4g38510	Ribosomal protein L1

**Species**	**Orthologous genes**	**Putative function**
Rice	LOC_Os01g05610	Core histone H2A/H2B/H3/H4 domain containing protein, putative, expressed
	LOC_Os01g05630	Core histone H2A/H2B/H3/H4 domain containing protein, putative, expressed

Additional File 2 cont.: Orthologous Proteins from Different Plant Species

	LOC_Os01g05900	Core histone H2A/H2B/H3/H4 domain containing protein, putative
	LOC_Os01g05970	OsFBO1 - F-box and other domain containing protein, expressed
	LOC_Os01g06010	Core histone H2A/H2B/H3/H4 domain containing protein, putative, expressed
	LOC_Os01g62230	Core histone H2A/H2B/H3/H4 domain containing protein, putative, expressed
	LOC_Os05g49860	Core histone H2A/H2B/H3/H4 domain containing protein, putative, expressed
	LOC_Os08g38300	Core histone H2A/H2B/H3/H4 domain containing protein, putative, expressed
Arabidopsis	AT1G07790	Histone superfamily protein
	AT2G28720	Histone superfamily protein
	AT3G45980	Histone superfamily protein
	AT3G46030	Histone superfamily protein
	AT5G22880	histone B2
	AT5G59910	Histone superfamily protein
Poplar	POPTR_0008s02990	HTB1; DNA binding
	POPTR_0008s03040	histone H2B, putative
	POPTR_0008s03050	histone H2B, putative
	POPTR_0008s03060	HTB9; DNA binding
	POPTR_0010s23720	histone H2B, putative
	POPTR_0010s23730	histone H2B, putative
	POPTR_0010s23770	HTB9; DNA binding
Grapevine	GSVIVG00025021001	Histone H2B
	GSVIVG00025023001	Histone H2B
	GSVIVG00025025001	Histone H2B
Sorghum	Sb02g025410	Histone H2B
	Sb02g041800	Histone H2B
	Sb03g005720	Histone H2B
	Sb03g005730	Histone H2B
	Sb03g007700	Histone H2B
	Sb03g026260	Histone H2B
	Sb03g039310	Histone H2B
	Sb04g030340	Histone H2B
	Sb07g022370	Histone H2B
	Sb07g028760	Histone H2B
	Sb09g022610	Histone H2B
Maize	GRMZM2G071959	Histone H2B.1
	GRMZM2G112912	Histone H2B
	GRMZM2G119071	Histone H2B.2
	GRMZM2G141432	Histone H2B
	GRMZM2G163939	Histone H2B
	GRMZM2G304575	Histone H2B
	GRMZM2G306258	Histone H2B.4
	GRMZM2G342515	Histone H2B.5
	GRMZM2G401147	Histone H2B
	GRMZM2G472696	Histone H2B

Additional File 2 cont.: Orthologous Proteins from Different Plant Species

Brachypodium	Bradi1g08860	Histone H2B.1
	Bradi1g47980	Histone H2B
	Bradi1g56060	Histone H2B.1
	Bradi2g00510	Histone H2B
	Bradi2g00530	Histone H2B
	Bradi2g23230	Histone H2B
	Bradi2g27710	Histone H2B.1
	Bradi2g27760	Histone H2B
	Bradi2g54540	Histone H2B.11
	Bradi3g54520	Histone H2B

**Species**	**Orthologous genes**	**Putative function**
Rice	LOC_Os01g62040	ruvB-like, putative, expressed
	LOC_Os07g08170	ruvB-like, putative, expressed
Arabidopsis	AT5G22330	P-loop containing nucleoside triphosphate hydrolases superfamily protein
Poplar	POPTR_0004s20930	RIN1 (RESISTANCE TO PSEUDOMONAS SYRINGAE PV MACULICOLA INTERACTOR 1); protein binding
Grapevine	GSVIVG0002518100<br>1	DNA helicase
Sorghum	Sb03g039200	RuvB 1,49-kDa TATA box-binding protein-interacting protein
	Sb04g002430	RuvB 1,49-kDa TATA box-binding protein-interacting protein
Maize	GRMZM2G032267	RuvB 1,49-kDa TATA box-binding protein-interacting protein
Brachypodium	Bradi2g54390	RuvB 1,49-kDa TATA box-binding protein-interacting protein

**Species**	**Orthologous genes**	**Putative function**
Rice	LOC_Os01g61920	Core histone H2A/H2B/H3/H4 domain containing protein, putative, expressed
	LOC_Os02g45940	Core histone H2A/H2B/H3/H4 domain containing protein, putative, expressed
	LOC_Os03g02780	Core histone H2A/H2B/H3/H4 domain containing protein, putative, expressed
	LOC_Os04g49420	Core histone H2A/H2B/H3/H4 domain containing protein, putative, expressed
	LOC_Os05g38740	Core histone H2A/H2B/H3/H4 domain containing protein, putative, expressed
	LOC_Os05g39050	Core histone H2A/H2B/H3/H4 domain containing protein, putative, expressed
	LOC_Os07g36500	Core histone H2A/H2B/H3/H4 domain containing protein, putative, expressed
	LOC_Os09g26340	Core histone H2A/H2B/H3/H4 domain containing protein, putative, expressed
	LOC_Os09g38020	Core histone H2A/H2B/H3/H4 domain containing protein, putative, expressed
	LOC_Os10g39410	Core histone H2A/H2B/H3/H4 domain containing protein, putative, expressed
Arabidopsis	AT1G07660	Histone superfamily protein
	AT1G07820	Histone superfamily protein
	AT2G28740	histone H4
	AT3G45930	Histone superfamily protein
	AT3G46320	Histone superfamily protein
	AT3G53730	Histone superfamily protein
	AT5G59690	Histone superfamily protein
	AT5G59970	Histone superfamily protein
Poplar	POPTR_0005s11740	histone H4
	POPTR_0005s11770	histone H4
	POPTR_0006s18360	histone H4

Additional File 2 cont.: Orthologous Proteins from Different Plant Species

	POPTR_0007s14000	histone H4
	POPTR_0007s14020	histone H4
	POPTR_0007s14090	histone H4
	POPTR_0008s04720	histone H4
	POPTR_0010s22080	histone H4
	POPTR_0010s22090	histone H4
	POPTR_0018s10040	histone H4
	POPTR_0018s10070	histone H4
	POPTR_0018s10080	histone H4
	POPTR_0168s00200	histone H4
Grapevine	GSVIVG00016576001	Histone H4
Sorghum	Sb01g030460	Histone H4
	Sb01g049250	Histone H4
	Sb02g025440	Histone H4
	Sb02g032240	Histone H4
	Sb03g004840	Histone H4
	Sb03g004870	Histone H4
	Sb03g004890	Histone H4
	Sb03g039090	Histone H4
	Sb04g031620	Histone H4
	Sb06g026490	Histone H4
	Sb09g022920	Histone H4
Maize	AC196961.2_FG003	Histone H4
	AC212565.3_FG001	similar to germinal histone H4 gene
	AC233865.1_FG001	Histone H4
	GRMZM2G016232	Histone H4
	GRMZM2G063896	Histone H4
	GRMZM2G072855	Histone H4
	GRMZM2G073275	Histone H4
	GRMZM2G084195	Histone H4
	GRMZM2G143780	Histone H4
	GRMZM2G149178	Histone H4
	GRMZM2G181153	Histone H4
	GRMZM2G332838	similar to germinal histone H4 gene
	GRMZM2G349651	Histone H4
	GRMZM2G421279	Histone H4
	GRMZM2G479684	Histone H4
Brachypodium	Bradi1g05980	Histone H4
	Bradi1g06000	Histone H4
	Bradi1g68190	Histone H4
	Bradi1g77230	Histone H4
	Bradi2g22790	Histone H4
	Bradi2g22990	Histone H4
	Bradi2g23010	Histone H4

	Bradi3g51930	Histone H4
	Bradi4g06040	Histone H4
	Bradi4g30960	Histone H4
	Bradi4g37340	Histone H4
	Bradi5g19350	Histone H4
	Bradi5g19360	Histone H4

**Species**	**Orthologous genes**	**Putative function**
Rice	LOC_Os01g59990	ribosomal protein L24, putative, expressed
	LOC_Os05g40820	ribosomal protein L24, putative, expressed
	LOC_Os07g12250	ribosomal protein L24, putative, expressed
Arabidopsis	AT2G36620	ribosomal protein L24
	AT3G53020	Ribosomal protein L24e family protein
Poplar	POPTR_0003s12330	RPL24A (ribosomal protein L24); structural constituent of ribosome
	POPTR_0004s08370	RPL24A (ribosomal protein L24); structural constituent of ribosome
	POPTR_0012s13380	RPL24A (ribosomal protein L24); structural constituent of ribosome
	POPTR_0015s13330	RPL24A (ribosomal protein L24); structural constituent of ribosome
Grapevine	GSVIVG00010601001	60S ribosomal protein L24
	GSVIVG00033743001	60S ribosomal protein L24
	GSVIVG00034676001	60S ribosomal protein L24
Sorghum	Sb01g015240	60S ribosomal protein L24
	Sb09g023800	60S ribosomal protein L24
Maize	GRMZM2G074898	60S ribosomal protein L24
	GRMZM2G110328	60S ribosomal protein L24
	GRMZM2G142640	60S ribosomal protein L24
Brachypodium	Bradi2g53220	60S ribosomal protein L24

**Species**	**Orthologous genes**	**Putative function**
Rice	LOC_Os01g59500	U3 small nucleolar RNA-associated protein 11, putative, expressed
Arabidopsis	AT3G60360	embryo sac development arrest 14
Poplar	POPTR_0016s02490	EDA14 (EMBRYO SAC DEVELOPMENT ARREST 14)
Grapevine	GSVIVG00029836001	U3 small nucleolar RNA-associated protein
Sorghum	Sb03g037600	U3 small nucleolar RNA-associated protein 11
Maize	GRMZM2G121785	U3 small nucleolar RNA-associated protein 11
Brachypodium	Bradi2g52910	U3 small nucleolar RNA-associated protein 11

**Species**	**Orthologous genes**	**Putative function**
Rice	LOC_Os01g54870	60S ribosomal protein L18a, putative, expressed
Brachypodium	Bradi2g50020	60S ribosomal protein L18a

**Species**	**Orthologous genes**	**Putative function**
Rice	LOC_Os01g52470	elongation factor, putative, expressed

Additional File 2 cont.: Orthologous Proteins from Different Plant Species

	LOC_Os01g53900	elongation factor, putative, expressed
	LOC_Os02g32030	elongation factor, putative, expressed
	LOC_Os04g02820	elongation factor, putative, expressed
Arabidopsis	AT1G56070	Ribosomal protein S5/Elongation factor G/III/V family protein
	AT3G12915	Ribosomal protein S5/Elongation factor G/III/V family protein
Poplar	POPTR_0005s10090	LOS1; copper ion binding / translation elongation factor/ translation factor, nucleic acid binding
	POPTR_0007s08390	LOS1; copper ion binding / translation elongation factor/ translation factor, nucleic acid binding
	POPTR_0007s08400	LOS1; copper ion binding / translation elongation factor/ translation factor, nucleic acid binding
Sorghum	Sb01g002040	Elongation factor 2
	Sb03g033210	Elongation factor 2
	Sb03g034200	Elongation factor 2
Maize	AC203173.3_FG004	Elongation factor 2
	GRMZM2G040369	Elongation factor 2
	GRMZM2G095851	Elongation factor 2
	GRMZM2G113250	Elongation factor 2
Brachypodium	Bradi2g45070	Elongation factor 2
	Bradi3g44160	Elongation factor 2
	Bradi3g44480	Elongation factor 2

**Species**	**Orthologous genes**	**Putative function**
Rice	LOC_Os01g52490	40S ribosomal protein S24, putative, expressed
	LOC_Os02g13530	40S ribosomal protein S24, putative, expressed
	LOC_Os06g36160	40S ribosomal protein S24, putative, expressed
Arabidopsis	AT3G04920	Ribosomal protein S24e family protein
	AT5G28060	Ribosomal protein S24e family protein
Poplar	POPTR_0005s05120	40S ribosomal protein S24 (RPS24B)
	POPTR_0008s15190	40S ribosomal protein S24 (RPS24B)
	POPTR_0010s09820	40S ribosomal protein S24 (RPS24B)
	POPTR_0030s00470	40S ribosomal protein S24 (RPS24B)
Grapevine	GSVIVG0000116700 1	40S ribosomal protein S24
Sorghum	Sb03g033230	40S ribosomal protein S24
	Sb04g008130	40S ribosomal protein S24
	Sb10g021950	40S ribosomal protein S24
Maize	GRMZM2G029685	40S ribosomal protein S24
	GRMZM2G091383	40S ribosomal protein S24
	GRMZM2G171426	40S ribosomal protein S24
Brachypodium	Bradi1g38010	40S ribosomal protein S24
	Bradi3g08680	40S ribosomal protein S24

**Species**	**Orthologous genes**	**Putative function**
Rice	LOC_Os01g51300	WD domain, G-beta repeat domain containing protein, expressed
Arabidopsis	AT2G19520	Transducin family protein / WD-40 repeat family protein
Poplar	POPTR_0006s15630	FVE; metal ion binding

Additional File 2 cont.: Orthologous Proteins from Different Plant Species

	POPTR_0018s06840	FVE; metal ion binding
Grapevine	GSVIVG00015200001	WD-repeat protein
	GSVIVG00016852001	WD-repeat protein
Sorghum	Sb03g032630	WD-40 repeat protein MSI4
Maize	GRMZM2G137965	WD-40 repeat protein MSI4
	GRMZM2G316113	Nucleosome/chromatin assembly factor C
Brachypodium	Bradi2g47940	WD-40 repeat protein MSI4

**Species**	**Orthologous genes**	**Putative function**
Rice	LOC_Os01g49290	WD repeat-containing protein, putative, expressed
Arabidopsis	AT1G18080	Transducin/WD40 repeat-like superfamily protein
	AT1G48630	receptor for activated C kinase 1B
	AT3G18130	receptor for activated C kinase 1C
Poplar	POPTR_0012s04700	ATARCA; nucleotide binding
	POPTR_0015s04250	ATARCA; nucleotide binding
Sorghum	Sb03g031520	Guanine nucleotide-binding protein subunit beta
	Sb09g027690	Guanine nucleotide-binding protein beta subunit
Maize	GRMZM2G038032	Guanine nucleotide-binding protein beta subunit
	GRMZM2G040477	Guanine nucleotide-binding protein beta subunit
Brachypodium	Bradi2g17350	Guanine nucleotide-binding protein beta subunit
	Bradi2g46840	Guanine nucleotide-binding protein subunit beta

**Species**	**Orthologous genes**	**Putative function**
Rice	LOC_Os01g47660	60S ribosomal protein L18a, putative, expressed
	LOC_Os05g49030	60S ribosomal protein L18a, putative, expressed
Arabidopsis	AT2G34480	Ribosomal protein L18ae/LX family protein
Poplar	POPTR_0002s05870	60S ribosomal protein L18A (RPL18aB)
	POPTR_0011s06730	60S ribosomal protein L18A (RPL18aB)
	POPTR_1064s00200	60S ribosomal protein L18A (RPL18aB)
Grapevine	GSVIVG00000485001	60S ribosomal protein L18a
	GSVIVG00021430001	similar to RPL18AA (60S RIBOSOMAL PROTEIN L18A-1)
Sorghum	Sb03g030600	60S ribosomal protein L18a
	Sb05g007540	60S ribosomal protein L18a
	Sb09g028590	60S ribosomal protein L18a
Maize	AC230013.2_FG007	60S ribosomal protein L18a
	GRMZM2G113720	60S ribosomal protein L18a
Brachypodium	Bradi2g16430	60S ribosomal protein L18a
	Bradi2g46010	60S ribosomal protein L18a

**Species**	**Orthologous genes**	**Putative function**
Rice	LOC_Os01g46060	NUC189 domain containing protein, expressed
	LOC_Os12g43830	NUC189 domain containing protein, expressed

Additional File 2 cont.: Orthologous Proteins from Different Plant Species

Arabidopsis	AT1G15420	NA
Poplar	POPTR_0001s17320	unknown protein
	POPTR_0003s05960	unknown protein
Grapevine	GSVIVG00017246001	Nucleotide binding protein
Sorghum	Sb09g027390	similar to WD repeat domain 43
Maize	GRMZM2G041472	similar to WD repeat domain 43
Brachypodium	Bradi4g00800	similar to WD repeat domain 43

**Species**	**Orthologous genes**	**Putative function**
Rice	LOC_Os01g42820	RNA recognition motif containing protein, putative, expressed
Arabidopsis	AT3G15010	RNA-binding (RRM/RBD/RNP motifs) family protein
Poplar	POPTR_0001s38640	RNA recognition motif (RRM)-containing protein
Sorghum	Sb03g027820	Heterogeneous nuclear ribonucleoprotein A3 2
Maize	GRMZM5G803433	Heterogeneous nuclear ribonucleoprotein A3 2
Brachypodium	Bradi2g43460	Heterogeneous nuclear ribonucleoprotein A3 2

**Species**	**Orthologous genes**	**Putative function**
Rice	LOC_Os01g34200	AATF, putative, expressed
Arabidopsis	AT5G61330	rRNA processing protein-related
Poplar	POPTR_0019s11490	rRNA processing protein-related
Grapevine	GSVIVG00018246001	apoptosis antagonizing transcription factor
Sorghum	Sb03g009240	Protein AATF
Maize	GRMZM2G031239	Protein AATF
Brachypodium	Bradi2g03790	Protein AATF

**Species**	**Orthologous genes**	**Putative function**
Rice	LOC_Os01g33050	ribosomal protein L24, putative, expressed
	LOC_Os07g19190	ribosomal protein L24, putative, expressed
Arabidopsis	AT2G44860	Ribosomal protein L24e family protein
Poplar	POPTR_0004s19930	60S ribosomal protein L24, putative
	POPTR_0009s15070	60S ribosomal protein L24, putative
Grapevine	GSVIVG00031394001	60S ribosomal protein L24
	GSVIVG00033788001	60S ribosomal protein L24
Sorghum	Sb09g028470	60S ribosomal protein L24
Brachypodium	Bradi2g03070	60S ribosomal protein L24
	Bradi4g01560	60S ribosomal protein L24

**Species**	**Orthologous genes**	**Putative function**
Rice	LOC_Os01g33030	Brix domain containing protein, putative, expressed
Arabidopsis	AT3G23620	Ribosomal RNA processing Brix domain protein
Poplar	POPTR_0015s10010	brix domain-containing protein
Grapevine	GSVIVG00033782001	Brix domain containing protein

Additional File 2 cont.: Orthologous Proteins from Different Plant Species

Sorghum	Sb09g024910	Brix domain-containing protein 1
Maize	GRMZM2G069518	Brix domain-containing protein 1
Brachypodium	Bradi2g57720	Ribosome production factor 2 homolog
	Bradi4g31410	Ribosome production factor 2 homolog

**Species**	**Orthologous genes**	**Putative function**
Rice	LOC_Os01g27730	GTPase of unknown function domain containing protein, putative, expressed
Arabidopsis	AT3G07050	GTP-binding family protein
Poplar	POPTR_0002s24220	GTP-binding family protein
Grapevine	GSVIVG00028584001	GTP-binding protein-plant
Sorghum	Sb01g015410	Guanine nucleotide-binding protein-like 3
Maize	GRMZM2G140602	Guanine nucleotide-binding protein-like 3
	GRMZM2G403149	Guanine nucleotide-binding protein-like 3
Brachypodium	Bradi1g31710	Guanine nucleotide-binding protein-like 3
	Bradi1g76700	Guanine nucleotide-binding protein-like 3

**Species**	**Orthologous genes**	**Putative function**
Rice	LOC_Os01g25610	40S ribosomal protein S4, putative, expressed
	LOC_Os02g01560	40S ribosomal protein S4, putative, expressed
Arabidopsis	AT2G17360	Ribosomal protein S4 (RPS4A) family protein
	AT5G07090	Ribosomal protein S4 (RPS4A) family protein
	AT5G58420	Ribosomal protein S4 (RPS4A) family protein
Poplar	POPTR_0006s10460	40S ribosomal protein S4 (RPS4B)
	POPTR_0013s01230	40S ribosomal protein S4 (RPS4B)
	POPTR_0015s05120	40S ribosomal protein S4 (RPS4B)
	POPTR_0015s09110	40S ribosomal protein S4 (RPS4B)
	POPTR_0016s13210	40S ribosomal protein S4 (RPS4D)
Grapevine	GSVIVG00024668001	similar to 40S ribosomal S4 protein
	GSVIVG00032756001	similar to 40S ribosomal S4 protein
Sorghum	Sb01g001850	40S ribosomal protein S4
	Sb03g014380	40S ribosomal protein S4
Maize	GRMZM2G125271	40S ribosomal protein S4
Brachypodium	Bradi2g35620	Ribosomal protein

**Species**	**Orthologous genes**	**Putative function**
Rice	LOC_Os01g24690	60S ribosomal protein L23A, putative, expressed
	LOC_Os04g42270	60S ribosomal protein L23A, putative, expressed
Arabidopsis	AT2G39460	ribosomal protein L23AA
	AT3G55280	ribosomal protein L23AB
Poplar	POPTR_0006s22980	RPL23AB (RIBOSOMAL PROTEIN L23AB); RNA binding / nucleotide binding / structural constituent of ribosome
	POPTR_0008s05000	RPL23AB (RIBOSOMAL PROTEIN L23AB); RNA binding / nucleotide binding / structural constituent of ribosome
	POPTR_0009s03210	RPL23AB (RIBOSOMAL PROTEIN L23AB); RNA binding / nucleotide binding / structural constituent of ribosome

Additional File 2 cont.: Orthologous Proteins from Different Plant Species

	POPTR_0010s21740	RPL23AB (RIBOSOMAL PROTEIN L23AB); RNA binding / nucleotide binding / structural constituent of ribosome
	POPTR_0016s08070	RPL23AB (RIBOSOMAL PROTEIN L23AB); RNA binding / nucleotide binding / structural constituent of ribosome
Grapevine	GSVIVG00025041001	60S ribosomal protein L23a
Sorghum	Sb06g021660	60S ribosomal protein L23a
Maize	GRMZM2G083253	60S ribosomal protein L23a
	GRMZM2G166659	60S ribosomal protein L23a
Brachypodium	Bradi1g06220	60S ribosomal protein L23a
	Bradi5g14750	60S ribosomal protein L23a

**Species**	**Orthologous genes**	**Putative function**
Rice	LOC_Os01g21940	WD domain, G-beta repeat domain containing protein, expressed
Arabidopsis	AT1G73720	transducin family protein / WD-40 repeat family protein
Poplar	POPTR_0011s16030	transducin family protein / WD-40 repeat family protein
Grapevine	GSVIVG00007685001	WD-40 repeat protein
Sorghum	Sb03g013120	WD-40 repeat protein
Maize	GRMZM2G037683	WD-40 repeat protein
Brachypodium	Bradi2g12110	WD-40 repeat protein

**Species**	**Orthologous genes**	**Putative function**
Rice	LOC_Os01g21590	homeodomain, putative, expressed
Arabidopsis	AT3G10040	sequence-specific DNA binding transcription factors
Poplar	POPTR_0006s11870	transcription factor
	POPTR_0016s11010	transcription factor
Grapevine	GSVIVG00033746001	Homeodomain
Sorghum	Sb03g013050	Homeodomain
Maize	GRMZM2G305362	Homeodomain
	GRMZM5G818655	Homeodomain
Brachypodium	Bradi2g12030	Transcription factor

**Species**	**Orthologous genes**	**Putative function**
Rice	LOC_Os01g16870	argonaute, putative, expressed
	LOC_Os04g06770	argonaute, putative, expressed
Arabidopsis	AT2G27040	Argonaute family protein
	AT2G32940	Argonaute family protein
	AT5G21030	PAZ domain-containing protein / piwi domain-containing protein
	AT5G21150	Argonaute family protein
Poplar	POPTR_0001s22710	AGO4 (ARGONAUTE 4); nucleic acid binding
	POPTR_0006s02680	AGO4 (ARGONAUTE 4); nucleic acid binding
	POPTR_0008s01100	AGO4 (ARGONAUTE 4); nucleic acid binding
	POPTR_0014s15760	AGO6 (ARGONAUTE 6); nucleic acid binding
	POPTR_0016s02480	AGO4 (ARGONAUTE 4); nucleic acid binding
Grapevine	GSVIVG00001941001	AGO4-2

Additional File 2 cont.: Orthologous Proteins from Different Plant Species

	GSVIVG00025868001	Argonaute protein group
	GSVIVG00030512001	Argonaute protein group
	GSVIVG00037488001	Eukaryotic translation initiation factor 2c
Sorghum	Sb03g011020	Protein argonaute 4A
	Sb09g030910	Protein argonaute 4B
Maize	GRMZM2G141818	Protein argonaute 4B
	GRMZM2G347402	Protein argonaute 16
	GRMZM2G589579	Protein argonaute 4A
Brachypodium	Bradi2g10370	Protein argonaute 4A
	Bradi2g14150	Protein argonaute 4B
	Bradi4g08590	Protein argonaute 16

Species	Orthologous genes	Putative function
Rice	LOC_Os01g16290	DNA gyrase subunit B, chloroplast/mitochondrial precursor, putative, expressed
Arabidopsis	AT3G10270	DNA GYRASE B1
	AT5G04130	DNA GYRASE B2
Poplar	POPTR_0006s03770	DNA topoisomerase, ATP-hydrolyzing, putative / DNA topoisomerase II, putative / DNA gyrase, putative
	POPTR_0016s03560	DNA topoisomerase, ATP-hydrolyzing, putative / DNA topoisomerase II, putative / DNA gyrase, putative
Grapevine	GSVIVG00033639001	DNA gyrase subunit B, chloroplastic/mitochondrial
Sorghum	Sb03g010630	DNA gyrase subunit B, chloroplastic/mitochondrial
Maize	GRMZM2G111014	DNA gyrase subunit B, chloroplastic/mitochondrial
Brachypodium	Bradi2g10040	DNA gyrase subunit B, chloroplastic/mitochondrial

Species	Orthologous genes	Putative function
Rice	LOC_Os01g16220	Sad1 / UNC-like C-terminal domain containing protein, putative, expressed
	LOC_Os05g18770	Sad1 / UNC-like C-terminal domain containing protein, putative, expressed
Arabidopsis	AT3G10730	SAD1/UNC-84 domain protein 2
	AT5G04990	SAD1/UNC-84 domain protein 1
Poplar	POPTR_0008s01140	sad1/unc-84 protein-related
	POPTR_0010s25420	sad1/unc-84 protein-related
Grapevine	GSVIVG00001935001	Sad1/unc-84 2
Sorghum	Sb04g005160	Sad1-unc84
Maize	GRMZM2G109818	Sad1-unc84
	GRMZM2G440614	Sad1/unc-84 2
Brachypodium	Bradi2g09990	Sad1/unc-84 2
	Bradi2g36230	Sad1-unc84

Species	Orthologous genes	Putative function
Rice	LOC_Os01g16010	BCAS2 protein, putative, expressed
Arabidopsis	AT3G18165	"modifier of snc1,4"
Poplar	POPTR_0015s04220	MOS4 (Modifier of snc1,4)
Grapevine	GSVIVG00008385001	Breast carcinoma amplified sequence

Additional File 2 cont.: Orthologous Proteins from Different Plant Species

Sorghum	Sb03g010330	BCAS2 protein
Maize	GRMZM2G419085	BCAS2 protein
Brachypodium	Bradi2g09770	BCAS2 protein

**Species**	**Orthologous genes**	**Putative function**
Rice	LOC_Os01g14950	importin subunit alpha, putative, expressed
	LOC_Os05g06350	importin subunit alpha, putative, expressed
Arabidopsis	AT3G06720	importin alpha isoform 1
	AT4G16143	importin alpha isoform 2
Poplar	POPTR_0005s02030	IMPA-1 (IMPORTIN ALPHA ISOFORM 1); binding / protein transporter
	POPTR_0008s22930	IMPA-1 (IMPORTIN ALPHA ISOFORM 1); binding / protein transporter
	POPTR_0013s01220	IMPA-1 (IMPORTIN ALPHA ISOFORM 1); binding / protein transporter
Grapevine	GSVIVG00031205001	Importin alpha
	GSVIVG00035047001	Impa2
Sorghum	Sb03g009700	Importin alpha-1b subunit
	Sb09g004320	Importin subunit alpha-1b
Maize	GRMZM2G009845	Importin subunit alpha-1b
	GRMZM2G088088	Importin subunit alpha-1b
	GRMZM2G091119	Importin alpha-1b subunit
Brachypodium	Bradi2g08960	Importin subunit alpha-1a
	Bradi2g35050	Importin subunit alpha-1b

**Species**	**Orthologous genes**	**Putative function**
Rice	LOC_Os01g13730	WD domain, G-beta repeat domain containing protein, expressed
Arabidopsis	AT4G28450	nucleotide binding;protein binding
Poplar	POPTR_0017s04650	nucleotide binding / protein binding
Grapevine	GSVIVG00020601001	U3 small nucleolar RNA (U3 snorna) associated protein
Sorghum	Sb01g000780	Protein SOF1
	Sb03g000410	Protein SOF1
Maize	GRMZM2G590033	Protein SOF1
Brachypodium	Bradi5g21640	Protein SOF1

**Species**	**Orthologous genes**	**Putative function**
Rice	LOC_Os06g51220	HMG1/2, putative, expressed
Arabidopsis	AT1G20693	high mobility group B2
	AT1G20696	high mobility group B3
Poplar	POPTR_0002s01000	HMGB3 (HIGH MOBILITY GROUP B 3); DNA binding / chromatin binding / structural constituent of chromatin / transcription factor
	POPTR_0005s10440	HMGB2 (HIGH MOBILITY GROUP B 2); DNA binding / chromatin binding / structural constituent of chromatin / transcription factor
	POPTR_0005s27390	HMGB3 (HIGH MOBILITY GROUP B 3); DNA binding / chromatin binding / structural constituent of chromatin / transcription factor
Grapevine	GSVIVG00013385001	HMG 1 protein
	GSVIVG00026642001	High mobility group protein

Additional File 2 cont.: Orthologous Proteins from Different Plant Species

Sorghum	Sb10g030910	DNA-binding protein MNB1B
Maize	GRMZM5G834758	DNA-binding protein MNB1B
Brachypodium	Bradi1g29730	HMG protein

**Species**	**Orthologous genes**	**Putative function**
Rice	LOC_Os01g08970	SSRP1-like FACT complex subunit, putative, expressed
	LOC_Os05g08970	SSRP1-like FACT complex subunit, putative, expressed
Arabidopsis	AT3G28730	high mobility group
Poplar	POPTR_0004s12290	ATHMG (ARABIDOPSIS THALIANA HIGH MOBILITY GROUP); transcription factor
	POPTR_0017s11460	ATHMG (ARABIDOPSIS THALIANA HIGH MOBILITY GROUP); transcription factor
Grapevine	GSVIVG00011349001	Structure-specific recognition protein
Sorghum	Sb03g003450	FACT complex subunit SSRP1
	Sb09g005650	FACT complex subunit SSRP1-B
Maize	GRMZM2G032252	Structure-specific recognition protein 1
	GRMZM5G842484	FACT complex subunit SSRP1-B
Brachypodium	Bradi2g33580	FACT complex subunit SSRP1-B

**Species**	**Orthologous genes**	**Putative function**
Rice	LOC_Os01g08770	WD domain, G-beta repeat domain containing protein, expressed
Arabidopsis	AT3G10530	Transducin/WD40 repeat-like superfamily protein
Poplar	POPTR_0010s23880	transducin family protein / WD-40 repeat family protein
Grapevine	GSVIVG00034665001	WD-repeat protein
Sorghum	Sb04g021020	WD-repeat protein
	Sb10g030850	WD-repeat protein
Maize	GRMZM2G096051	WD-repeat protein
Brachypodium	Bradi1g04230	WD-repeat protein
	Bradi1g14400	WD-repeat protein

**Species**	**Orthologous genes**	**Putative function**
Rice	LOC_Os01g07810	protein of unknown function domain containing protein, expressed
Sorghum	Sb03g004340	Conserved gene of unknown function
Maize	GRMZM2G096240	Nucleic acid binding protein
	GRMZM2G098335	Nucleic acid binding protein
Brachypodium	Bradi2g04560	Conserved gene of unknown function

**Species**	**Orthologous genes**	**Putative function**
Rice	LOC_Os01g06290	splicing factor, arginine/serine-rich, putative, expressed
Sorghum	Sb03g005500	Splicing factor
Brachypodium	Bradi2g00370	Splicing factor

**Species**	**Orthologous genes**	**Putative function**
Rice	LOC_Os01g05610	Core histone H2A/H2B/H3/H4 domain containing protein, putative, expressed
	LOC_Os01g05630	Core histone H2A/H2B/H3/H4 domain containing protein, putative, expressed

Additional File 2 cont.: Orthologous Proteins from Different Plant Species

	LOC_Os01g05900	Core histone H2A/H2B/H3/H4 domain containing protein, putative
	LOC_Os01g05970	OsFBO1 - F-box and other domain containing protein, expressed
	LOC_Os01g06010	Core histone H2A/H2B/H3/H4 domain containing protein, putative, expressed
	LOC_Os01g62230	Core histone H2A/H2B/H3/H4 domain containing protein, putative, expressed
	LOC_Os05g49860	Core histone H2A/H2B/H3/H4 domain containing protein, putative, expressed
	LOC_Os08g38300	Core histone H2A/H2B/H3/H4 domain containing protein, putative, expressed
Arabidopsis	AT1G07790	Histone superfamily protein
	AT2G28720	Histone superfamily protein
	AT3G45980	Histone superfamily protein
	AT3G46030	Histone superfamily protein
	AT5G22880	histone B2
	AT5G59910	Histone superfamily protein
Poplar	POPTR_0008s02990	HTB1; DNA binding
	POPTR_0008s03040	histone H2B, putative
	POPTR_0008s03050	histone H2B, putative
	POPTR_0008s03060	HTB9; DNA binding
	POPTR_0010s23720	histone H2B, putative
	POPTR_0010s23730	histone H2B, putative
	POPTR_0010s23770	HTB9; DNA binding
Grapevine	GSVIVG00025021001	Histone H2B
	GSVIVG00025023001	Histone H2B
	GSVIVG00025025001	Histone H2B
Sorghum	Sb02g025410	Histone H2B
	Sb02g041800	Histone H2B
	Sb03g005720	Histone H2B
	Sb03g005730	Histone H2B
	Sb03g007700	Histone H2B
	Sb03g026260	Histone H2B
	Sb03g039310	Histone H2B
	Sb04g030340	Histone H2B
	Sb07g022370	Histone H2B
	Sb07g028760	Histone H2B
	Sb09g022610	Histone H2B
Maize	GRMZM2G071959	Histone H2B.1
	GRMZM2G112912	Histone H2B
	GRMZM2G119071	Histone H2B.2
	GRMZM2G141432	Histone H2B
	GRMZM2G163939	Histone H2B
	GRMZM2G304575	Histone H2B
	GRMZM2G306258	Histone H2B.4
	GRMZM2G342515	Histone H2B.5
	GRMZM2G401147	Histone H2B
	GRMZM2G472696	Histone H2B

Additional File 2 cont.: Orthologous Proteins from Different Plant Species

Brachypodium	Bradi1g08860	Histone H2B.1
	Bradi1g47980	Histone H2B
	Bradi1g56060	Histone H2B.1
	Bradi2g00510	Histone H2B
	Bradi2g00530	Histone H2B
	Bradi2g23230	Histone H2B
	Bradi2g27710	Histone H2B.1
	Bradi2g27760	Histone H2B
	Bradi2g54540	Histone H2B.11
	Bradi3g54520	Histone H2B

**Species**	**Orthologous genes**	**Putative function**
Rice	LOC_Os01g05610	Core histone H2A/H2B/H3/H4 domain containing protein, putative, expressed
	LOC_Os01g05630	Core histone H2A/H2B/H3/H4 domain containing protein, putative, expressed
	LOC_Os01g05900	Core histone H2A/H2B/H3/H4 domain containing protein, putative
	LOC_Os01g05970	OsFBO1 - F-box and other domain containing protein, expressed
	LOC_Os01g06010	Core histone H2A/H2B/H3/H4 domain containing protein, putative, expressed
	LOC_Os01g62230	Core histone H2A/H2B/H3/H4 domain containing protein, putative, expressed
	LOC_Os05g49860	Core histone H2A/H2B/H3/H4 domain containing protein, putative, expressed
	LOC_Os08g38300	Core histone H2A/H2B/H3/H4 domain containing protein, putative, expressed
Arabidopsis	AT1G07790	Histone superfamily protein
	AT2G28720	Histone superfamily protein
	AT3G45980	Histone superfamily protein
	AT3G46030	Histone superfamily protein
	AT5G22880	histone B2
	AT5G59910	Histone superfamily protein
Poplar	POPTR_0008s02990	HTB1; DNA binding
	POPTR_0008s03040	histone H2B, putative
	POPTR_0008s03050	histone H2B, putative
	POPTR_0008s03060	HTB9; DNA binding
	POPTR_0010s23720	histone H2B, putative
	POPTR_0010s23730	histone H2B, putative
	POPTR_0010s23770	HTB9; DNA binding
Grapevine	GSVIVG00025021001	Histone H2B
	GSVIVG00025023001	Histone H2B
	GSVIVG00025025001	Histone H2B
Sorghum	Sb02g025410	Histone H2B
	Sb02g041800	Histone H2B
	Sb03g005720	Histone H2B
	Sb03g005730	Histone H2B
	Sb03g007700	Histone H2B
	Sb03g026260	Histone H2B
	Sb03g039310	Histone H2B

Additional File 2 cont.: Orthologous Proteins from Different Plant Species

	Sb04g030340	Histone H2B
	Sb07g022370	Histone H2B
	Sb07g028760	Histone H2B
	Sb09g022610	Histone H2B
Maize	GRMZM2G071959	Histone H2B.1
	GRMZM2G112912	Histone H2B
	GRMZM2G119071	Histone H2B.2
	GRMZM2G141432	Histone H2B
	GRMZM2G163939	Histone H2B
	GRMZM2G304575	Histone H2B
	GRMZM2G306258	Histone H2B.4
	GRMZM2G342515	Histone H2B.5
	GRMZM2G401147	Histone H2B
	GRMZM2G472696	Histone H2B
Brachypodium	Bradi1g08860	Histone H2B.1
	Bradi1g47980	Histone H2B
	Bradi1g56060	Histone H2B.1
	Bradi2g00510	Histone H2B
	Bradi2g00530	Histone H2B
	Bradi2g23230	Histone H2B
	Bradi2g27710	Histone H2B.1
	Bradi2g27760	Histone H2B
	Bradi2g54540	Histone H2B.11
	Bradi3g54520	Histone H2B

Species	Orthologous genes	Putative function
Rice	LOC_Os01g05610	Core histone H2A/H2B/H3/H4 domain containing protein, putative, expressed
	LOC_Os01g05630	Core histone H2A/H2B/H3/H4 domain containing protein, putative, expressed
	LOC_Os01g05900	Core histone H2A/H2B/H3/H4 domain containing protein, putative
	LOC_Os01g05970	OsFBO1 - F-box and other domain containing protein, expressed
	LOC_Os01g06010	Core histone H2A/H2B/H3/H4 domain containing protein, putative, expressed
	LOC_Os01g62230	Core histone H2A/H2B/H3/H4 domain containing protein, putative, expressed
	LOC_Os05g49860	Core histone H2A/H2B/H3/H4 domain containing protein, putative, expressed
	LOC_Os08g38300	Core histone H2A/H2B/H3/H4 domain containing protein, putative, expressed
Arabidopsis	AT1G07790	Histone superfamily protein
	AT2G28720	Histone superfamily protein
	AT3G45980	Histone superfamily protein
	AT3G46030	Histone superfamily protein
	AT5G22880	histone B2
	AT5G59910	Histone superfamily protein
Poplar	POPTR_0008s02990	HTB1; DNA binding
	POPTR_0008s03040	histone H2B, putative
	POPTR_0008s03050	histone H2B, putative

Additional File 2 cont.: Orthologous Proteins from Different Plant Species

	POPTR_0008s03060	HTB9; DNA binding
	POPTR_0010s23720	histone H2B, putative
	POPTR_0010s23730	histone H2B, putative
	POPTR_0010s23770	HTB9; DNA binding
Grapevine	GSVIVG00025021001	Histone H2B
	GSVIVG00025023001	Histone H2B
	GSVIVG00025025001	Histone H2B
Sorghum	Sb02g025410	Histone H2B
	Sb02g041800	Histone H2B
	Sb03g005720	Histone H2B
	Sb03g005730	Histone H2B
	Sb03g007700	Histone H2B
	Sb03g026260	Histone H2B
	Sb03g039310	Histone H2B
	Sb04g030340	Histone H2B
	Sb07g022370	Histone H2B
	Sb07g028760	Histone H2B
	Sb09g022610	Histone H2B
Maize	GRMZM2G071959	Histone H2B.1
	GRMZM2G112912	Histone H2B
	GRMZM2G119071	Histone H2B.2
	GRMZM2G141432	Histone H2B
	GRMZM2G163939	Histone H2B
	GRMZM2G304575	Histone H2B
	GRMZM2G306258	Histone H2B.4
	GRMZM2G342515	Histone H2B.5
	GRMZM2G401147	Histone H2B
	GRMZM2G472696	Histone H2B
Brachypodium	Bradi1g08860	Histone H2B.1
	Bradi1g47980	Histone H2B
	Bradi1g56060	Histone H2B.1
	Bradi2g00510	Histone H2B
	Bradi2g00530	Histone H2B
	Bradi2g23230	Histone H2B
	Bradi2g27710	Histone H2B.1
	Bradi2g27760	Histone H2B
	Bradi2g54540	Histone H2B.11
	Bradi3g54520	Histone H2B

**Species**	**Orthologous genes**	**Putative function**
Rice	LOC_Os01g05610	Core histone H2A/H2B/H3/H4 domain containing protein, putative, expressed
	LOC_Os01g05630	Core histone H2A/H2B/H3/H4 domain containing protein, putative, expressed
	LOC_Os01g05900	Core histone H2A/H2B/H3/H4 domain containing protein, putative

Additional File 2 cont.: Orthologous Proteins from Different Plant Species

	LOC_Os01g05970	OsFBO1 - F-box and other domain containing protein, expressed
	LOC_Os01g06010	Core histone H2A/H2B/H3/H4 domain containing protein, putative, expressed
	LOC_Os01g62230	Core histone H2A/H2B/H3/H4 domain containing protein, putative, expressed
	LOC_Os05g49860	Core histone H2A/H2B/H3/H4 domain containing protein, putative, expressed
	LOC_Os08g38300	Core histone H2A/H2B/H3/H4 domain containing protein, putative, expressed
Arabidopsis	AT1G07790	Histone superfamily protein
	AT2G28720	Histone superfamily protein
	AT3G45980	Histone superfamily protein
	AT3G46030	Histone superfamily protein
	AT5G22880	histone B2
	AT5G59910	Histone superfamily protein
Poplar	POPTR_0008s02990	HTB1; DNA binding
	POPTR_0008s03040	histone H2B, putative
	POPTR_0008s03050	histone H2B, putative
	POPTR_0008s03060	HTB9; DNA binding
	POPTR_0010s23720	histone H2B, putative
	POPTR_0010s23730	histone H2B, putative
	POPTR_0010s23770	HTB9; DNA binding
Grapevine	GSVIVG00025021001	Histone H2B
	GSVIVG00025023001	Histone H2B
	GSVIVG00025025001	Histone H2B
Sorghum	Sb02g025410	Histone H2B
	Sb02g041800	Histone H2B
	Sb03g005720	Histone H2B
	Sb03g005730	Histone H2B
	Sb03g007700	Histone H2B
	Sb03g026260	Histone H2B
	Sb03g039310	Histone H2B
	Sb04g030340	Histone H2B
	Sb07g022370	Histone H2B
	Sb07g028760	Histone H2B
	Sb09g022610	Histone H2B
Maize	GRMZM2G071959	Histone H2B.1
	GRMZM2G112912	Histone H2B
	GRMZM2G119071	Histone H2B.2
	GRMZM2G141432	Histone H2B
	GRMZM2G163939	Histone H2B
	GRMZM2G304575	Histone H2B
	GRMZM2G306258	Histone H2B.4
	GRMZM2G342515	Histone H2B.5
	GRMZM2G401147	Histone H2B
	GRMZM2G472696	Histone H2B
Brachypodium	Bradi1g08860	Histone H2B.1

Additional File 2 cont.: Orthologous Proteins from Different Plant Species

	Bradi1g47980	Histone H2B
	Bradi1g56060	Histone H2B.1
	Bradi2g00510	Histone H2B
	Bradi2g00530	Histone H2B
	Bradi2g23230	Histone H2B
	Bradi2g27710	Histone H2B.1
	Bradi2g27760	Histone H2B
	Bradi2g54540	Histone H2B.11
	Bradi3g54520	Histone H2B

**Species**	**Orthologous genes**	**Putative function**
Rice	LOC_Os01g05610	Core histone H2A/H2B/H3/H4 domain containing protein, putative, expressed
	LOC_Os01g05630	Core histone H2A/H2B/H3/H4 domain containing protein, putative, expressed
	LOC_Os01g05900	Core histone H2A/H2B/H3/H4 domain containing protein, putative
	LOC_Os01g05970	OsFBO1 - F-box and other domain containing protein, expressed
	LOC_Os01g06010	Core histone H2A/H2B/H3/H4 domain containing protein, putative, expressed
	LOC_Os01g62230	Core histone H2A/H2B/H3/H4 domain containing protein, putative, expressed
	LOC_Os05g49860	Core histone H2A/H2B/H3/H4 domain containing protein, putative, expressed
	LOC_Os08g38300	Core histone H2A/H2B/H3/H4 domain containing protein, putative, expressed
Arabidopsis	AT1G07790	Histone superfamily protein
	AT2G28720	Histone superfamily protein
	AT3G45980	Histone superfamily protein
	AT3G46030	Histone superfamily protein
	AT5G22880	histone B2
	AT5G59910	Histone superfamily protein
Poplar	POPTR_0008s02990	HTB1; DNA binding
	POPTR_0008s03040	histone H2B, putative
	POPTR_0008s03050	histone H2B, putative
	POPTR_0008s03060	HTB9; DNA binding
	POPTR_0010s23720	histone H2B, putative
	POPTR_0010s23730	histone H2B, putative
	POPTR_0010s23770	HTB9; DNA binding
Grapevine	GSVIVG00025021001	Histone H2B
	GSVIVG00025023001	Histone H2B
	GSVIVG00025025001	Histone H2B
Sorghum	Sb02g025410	Histone H2B
	Sb02g041800	Histone H2B
	Sb03g005720	Histone H2B
	Sb03g005730	Histone H2B
	Sb03g007700	Histone H2B
	Sb03g026260	Histone H2B
	Sb03g039310	Histone H2B
	Sb04g030340	Histone H2B

Additional File 2 cont.: Orthologous Proteins from Different Plant Species

	Sb07g022370	Histone H2B
	Sb07g028760	Histone H2B
	Sb09g022610	Histone H2B
Maize	GRMZM2G071959	Histone H2B.1
	GRMZM2G112912	Histone H2B
	GRMZM2G119071	Histone H2B.2
	GRMZM2G141432	Histone H2B
	GRMZM2G163939	Histone H2B
	GRMZM2G304575	Histone H2B
	GRMZM2G306258	Histone H2B.4
	GRMZM2G342515	Histone H2B.5
	GRMZM2G401147	Histone H2B
	GRMZM2G472696	Histone H2B
Brachypodium	Bradi1g08860	Histone H2B.1
	Bradi1g47980	Histone H2B
	Bradi1g56060	Histone H2B.1
	Bradi2g00510	Histone H2B
	Bradi2g00530	Histone H2B
	Bradi2g23230	Histone H2B
	Bradi2g27710	Histone H2B.1
	Bradi2g27760	Histone H2B
	Bradi2g54540	Histone H2B.11
	Bradi3g54520	Histone H2B

**Species**	**Orthologous genes**	**Putative function**
Rice	LOC_Os01g01510	sas10/Utp3 family protein, expressed
Arabidopsis	AT2G43650	Sas10/U3 ribonucleoprotein (Utp) family protein
Poplar	POPTR_0013s12950	EMB2777 (EMBRYO DEFECTIVE 2777)
Grapevine	GSVIVG00038137001	Something about silencing protein sas10
Sorghum	Sb07g028350	Sas10/Utp3 family protein
Maize	GRMZM2G080746	Sas10/U3 ribonucleoprotein family protein
Brachypodium	Bradi2g13340	Sas10/Utp3 family protein

**Species**	**Orthologous genes**	**Putative function**
Rice	LOC_Os01g01060	40S ribosomal protein S5, putative, expressed
Arabidopsis	AT2G37270	ribosomal protein 5B
	AT3G11940	ribosomal protein 5A
Poplar	POPTR_0006s21210	ATRPS5B (RIBOSOMAL PROTEIN 5B); structural constituent of ribosome
	POPTR_0016s06310	ATRPS5B (RIBOSOMAL PROTEIN 5B); structural constituent of ribosome
Grapevine	GSVIVG00026328001	40S ribosomal protein S5
	GSVIVG00033419001	40S ribosomal protein S5
Sorghum	Sb03g009210	40S ribosomal protein S5
Maize	GRMZM2G078985	40S ribosomal protein S5

Additional File 2 cont.: Orthologous Proteins from Different Plant Species

	GRMZM2G156673	40S ribosomal protein S5
Brachypodium	Bradi2g03760	40S ribosomal protein S5
	Bradi4g41050	40S ribosomal protein S5

**Species**	**Orthologous genes**	**Putative function**
Rice	LOC_Os07g41190	WD domain, G-beta repeat domain containing protein, expressed
Arabidopsis	AT5G14050	Transducin/WD40 repeat-like superfamily protein
Poplar	POPTR_0005s24010	transducin family protein / WD-40 repeat family protein
Grapevine	GSVIVG00000726001	Nucleotide binding protein
Sorghum	Sb03g003690	WD-repeat protein 50
	Sb10g029900	WD-repeat protein 50
Maize	GRMZM2G099758	WD-repeat protein 50
Brachypodium	Bradi2g18710	WD-repeat protein 50

**Species**	**Orthologous genes**	**Putative function**
Rice	LOC_Os06g40950	DNA-directed RNA polymerase I subunit RPA1, putative, expressed
Arabidopsis	AT3G57660	nuclear RNA polymerase A1
Poplar	POPTR_0006s05420	NRPA1; DNA binding / DNA-directed RNA polymerase/ zinc ion binding
Grapevine	GSVIVG00033327001	DNA-directed RNA polymerase
Sorghum	Sb09g027223	DNA-directed RNA polymerase
	Sb09g027230	DNA-directed RNA polymerase
Maize	GRMZM2G028132	DNA-directed RNA polymerase
	GRMZM2G043456	DNA-directed RNA polymerase
Brachypodium	Bradi1g02620	DNA-directed RNA polymerase
