# Supplementary material for: Nuclear proteome response to cell wall removal in rice (Oryza sativa)
Source: Proteome Sci. 2013 Jun 19;11:26. doi: 10.1186/1477-5956-11-26 (PMC3695858; doi:10.1186/1477-5956-11-26)
Supplement: Additional file 3 — As Pfam Domain Assignment for Proteins Identified with two or more peptides. [file 1477-5956-11-26-S3.pdf]

Additional File 3: Pfam Domain Assignment for Proteins Identified with two or more peptides

Model	Acc	Name	Type	Bit Score	E-value	PS	PE	MS	ME
LOC_Os01g01060	PF00177.14	Ribosomal_S7	Domain	141.6	9.90E-42	46	200	2	148
LOC_Os01g01510	PF09368.3	Sas10_Utp3_C	Domain	93.1	6.90E-27	569	643	2	76
LOC_Os01g05610	PF00125.17	Histone	Domain	77.7	4.20E-22	62	129	3	74
LOC_Os01g05630	PF00125.17	Histone	Domain	77.7	4.20E-22	62	129	3	74
LOC_Os01g05900	PF00125.17	Histone	Domain	77.7	4.20E-22	62	129	3	74
LOC_Os01g05970	PF00125.17	Histone	Domain	82.2	1.70E-23	62	129	3	74
LOC_Os01g06010	PF00125.17	Histone	Domain	79.6	1.10E-22	64	131	3	74
LOC_Os01g06290	PF00076.15	RRM_1	Domain	50.3	1.00E-13	20	80	1	68
LOC_Os01g06290	PF00098.16	zf-CCHC	Domain	26.7	2.60E-06	110	125	2	17
LOC_Os01g06290	PF00098.16	zf-CCHC	Domain	22.9	4.20E-05	132	148	2	18
LOC_Os01g06290	PF00076.15	RRM_1	Domain	50.3	1.00E-13	20	80	1	68
LOC_Os01g06290	PF00076.15	RRM_1	Domain	22.5	5.00E-05	3	32	39	68
LOC_Os01g06290	PF00098.16	zf-CCHC	Domain	23.2	3.40E-05	84	100	2	18
LOC_Os01g06290	PF00098.16	zf-CCHC	Domain	27	2.10E-06	62	77	2	17
LOC_Os01g06290	PF00076.15	RRM_1	Domain	51.4	4.80E-14	20	80	1	68
LOC_Os01g06290	PF00098.16	zf-CCHC	Domain	27.5	1.50E-06	110	125	2	17
LOC_Os01g06290	PF00098.16	zf-CCHC	Domain	23.7	2.40E-05	132	148	2	18
LOC_Os01g08770	PF08149.4	BING4CT	Domain	116.1	2.80E-34	356	435	2	80
LOC_Os01g08970	PF00505.12	HMG_box	Domain	70.3	1.10E-19	556	624	1	69
LOC_Os01g08970	PF00505.12	HMG_box	Domain	70.6	8.50E-20	450	518	1	69
LOC_Os01g16290	PF00204.18	DNA_gyraseB	Domain	184.1	9.60E-55	313	482	1	172
LOC_Os01g16290	PF02518.19	HATPase_c	Domain	67.7	4.90E-19	116	261	3	109
LOC_Os01g16290	PF02518.19	HATPase_c	Domain	68.1	3.70E-19	116	261	3	109
LOC_Os01g16290	PF00204.18	DNA_gyraseB	Domain	184.5	7.10E-55	313	482	1	172
LOC_Os01g16870	PF08699.3	DUF1785	Domain	73.4	5.50E-21	221	272	2	52
LOC_Os01g25610	PF08071.5	RS4NT	Domain	70	8.30E-20	3	40	1	38
LOC_Os01g33030	PF04427.11	Brix	Domain	120.3	5.90E-35	33	232	1	190
LOC_Os01g34200	PF08164.5	TRAUB	Domain	76.8	8.00E-22	334	409	1	83
LOC_Os01g36860	PF00270.22	DEAD	Domain	168.1	8.60E-50	174	343	1	166
LOC_Os01g36860	PF00397.19	WW	Domain	29.7	3.20E-07	19	49	1	30
LOC_Os01g36860	PF00270.22	DEAD	Domain	168.6	6.10E-50	33	202	1	166
LOC_Os01g36860	PF00270.22	DEAD	Domain	125.1	1.40E-36	3	134	36	166
LOC_Os01g36890	PF00270.22	DEAD	Domain	123.3	5.20E-36	2	153	16	164
LOC_Os01g42820	PF00076.15	RRM_1	Domain	61.5	3.20E-17	78	144	1	67
LOC_Os01g42820	PF00076.15	RRM_1	Domain	35.6	4.10E-09	179	236	1	57
LOC_Os01g45190	PF00270.22	DEAD	Domain	144.4	1.70E-42	56	218	2	164
LOC_Os01g45190	PF00270.22	DEAD	Domain	76.5	1.30E-21	8	100	73	164
LOC_Os01g47660	PF01775.10	Ribosomal_L18ae	Domain	192.4	1.70E-57	7	128	2	123
LOC_Os01g53900	PF03144.18	GTP_EFTU_D2	Domain	53.2	1.80E-14	388	464	2	74
LOC_Os01g53900	PF00679.17	EFG_C	Domain	67.6	5.20E-19	722	808	1	86
LOC_Os01g53900	PF03764.11	EFG_IV	Domain	118.3	1.00E-34	605	720	5	120
LOC_Os01g53900	PF00009.20	GTP_EFTU	Domain	197.7	8.80E-59	17	338	1	189
LOC_Os01g54870	PF01775.10	Ribosomal_L18ae	Domain	193.2	9.20E-58	7	128	2	123
LOC_Os01g61920	PF00125.17	Histone	Domain	52.8	2.60E-14	28	94	4	75
LOC_Os01g62230	PF00125.17	Histone	Domain	81.1	3.70E-23	48	115	3	74

Additional File 3 cont.: Pfam Domain Assignment for Proteins Identified with two or more peptides

LOC_Os01g64090	PF00687.14	Ribosomal_L1	Domain	73.1	1.50E-20	148	233	118	208
LOC_Os01g64090	PF00687.14	Ribosomal_L1	Domain	97.7	4.30E-28	6	133	6	125
LOC_Os01g64640	PF00125.17	Histone	Domain	112.2	7.40E-33	58	132	1	75
LOC_Os01g68320	PF00270.22	DEAD	Domain	160.9	1.40E-47	275	445	1	166
LOC_Os01g68320	PF00270.22	DEAD	Domain	161.3	1.00E-47	275	445	1	166
LOC_Os02g01250	PF01423.15	LSM	Domain	52.9	1.40E-14	11	74	2	67
LOC_Os02g01332	PF00347.16	Ribosomal_L6	Domain	53.1	2.60E-14	100	179	1	77
LOC_Os02g01332	PF00347.16	Ribosomal_L6	Domain	46.2	3.70E-12	12	88	1	77
LOC_Os02g01560	PF08071.5	RS4NT	Domain	70	8.30E-20	3	40	1	38
LOC_Os02g01740	PF00270.22	DEAD	Domain	90.5	6.00E-26	520	695	2	165
LOC_Os02g01740	PF00270.22	DEAD	Domain	84.4	4.70E-24	1364	1533	2	164
LOC_Os02g04050	PF06470.6	SMC_hinge	Domain	98.7	1.60E-28	518	630	2	120
LOC_Os02g05330	PF00270.22	DEAD	Domain	145.6	7.30E-43	66	227	2	162
LOC_Os02g06370	PF08536.4	Whirly	Domain	194.3	4.70E-58	52	189	2	139
LOC_Os02g07260	PF00162.12	PGK	Domain	547.8	7.50E-165	10	391	2	384
LOC_Os02g07260	PF00162.12	PGK	Domain	467.7	1.50E-140	10	337	2	334
LOC_Os02g08370	PF01088.14	Peptidase_C12	Domain	218.8	3.50E-65	2	204	1	215
LOC_Os02g10080	PF00013.22	KH_1	Domain	44	9.90E-12	172	234	1	57
LOC_Os02g10080	PF00013.22	KH_1	Domain	44.6	6.30E-12	103	165	1	57
LOC_Os02g16640	PF06220.5	zf-U1	Domain	76.8	4.90E-22	1	38	1	38
LOC_Os02g21660	PF00687.14	Ribosomal_L1	Domain	176.8	2.70E-52	7	211	7	208
LOC_Os02g21660	PF00687.14	Ribosomal_L1	Domain	160.1	3.50E-47	6	200	6	197
LOC_Os02g21660	PF00687.14	Ribosomal_L1	Domain	98.9	1.90E-28	6	133	6	125
LOC_Os02g32030	PF00009.20	GTP_EFTU	Domain	200.4	1.30E-59	18	342	2	189
LOC_Os02g32030	PF03144.18	GTP_EFTU_D2	Domain	49.9	1.90E-13	393	468	3	74
LOC_Os02g32030	PF03764.11	EFG_IV	Domain	126.2	3.60E-37	609	722	5	120
LOC_Os02g32030	PF00679.17	EFG_C	Domain	72.9	1.10E-20	724	811	1	87
LOC_Os02g36974	PF00244.13	14-3-3	Domain	384.7	8.30E-116	9	245	1	235
LOC_Os02g36974	PF00244.13	14-3-3	Domain	309.1	1.10E-92	9	204	1	194
LOC_Os02g36974	PF00244.13	14-3-3	Domain	251.6	4.00E-75	25	176	84	235
LOC_Os02g37430	PF01423.15	LSM	Domain	61.6	2.70E-17	6	70	2	68
LOC_Os02g37862	PF03868.8	Ribosomal_L6e_N	Domain	59.2	2.00E-16	4	48	13	58
LOC_Os02g38210	PF03143.10	GTP_EFTU_D3	Domain	131.4	9.90E-39	366	465	1	100
LOC_Os02g38210	PF03144.18	GTP_EFTU_D2	Domain	65.3	3.10E-18	293	362	1	74
LOC_Os02g38210	PF00009.20	GTP_EFTU	Domain	208.2	5.40E-62	68	269	1	188
LOC_Os02g39140	PF00010.19	HLH	Domain	39.7	2.30E-10	236	281	6	54
LOC_Os02g43930	PF00226.24	DnaJ	Domain	87	4.00E-25	14	71	2	64
LOC_Os02g47140	PF00298.12	Ribosomal_L11	Domain	50.9	9.90E-14	75	144	2	69
LOC_Os02g47140	PF03946.7	Ribosomal_L11_N	Domain	59	1.80E-16	15	70	4	59
LOC_Os02g47140	PF03946.7	Ribosomal_L11_N	Domain	59	1.80E-16	15	70	4	59
LOC_Os02g47140	PF00298.12	Ribosomal_L11	Domain	50.9	9.90E-14	75	144	2	69
LOC_Os02g50880	PF00089.19	Trypsin	Domain	59	3.70E-16	120	294	11	216
LOC_Os02g56960	PF00238.12	Ribosomal_L14	Domain	117.6	2.00E-34	21	140	3	122
LOC_Os02g57590	PF01269.10	Fibrillarin	Domain	378.3	5.90E-114	71	297	2	228
LOC_Os02g57590	PF01269.10	Fibrillarin	Domain	268	3.30E-80	71	227	2	158

Additional File 3 cont.: Pfam Domain Assignment for Proteins Identified with two or more peptides

LOC_Os03g01530	PF00091.18	Tubulin	Domain	239	3.70E-71	3	222	1	214
LOC_Os03g01530	PF03953.10	Tubulin_C	Domain	164	1.20E-48	261	382	1	124
LOC_Os03g05730	PF02359.11	CDC48_N	Domain	84.2	3.40E-24	32	114	1	86
LOC_Os03g05730	PF02933.10	CDC48_2	Domain	36.1	2.90E-09	133	195	2	61
LOC_Os03g06670	PF00125.17	Histone	Domain	81.8	2.30E-23	33	107	1	75
LOC_Os03g08010	PF00009.20	GTP_EFTU	Domain	188.6	5.60E-56	5	222	1	185
LOC_Os03g08010	PF03143.10	GTP_EFTU_D3	Domain	112.7	6.60E-33	322	429	2	100
LOC_Os03g08010	PF03144.18	GTP_EFTU_D2	Domain	62.5	2.40E-17	248	314	1	73
LOC_Os03g08020	PF03143.10	GTP_EFTU_D3	Domain	112.7	6.60E-33	322	429	2	100
LOC_Os03g08020	PF03144.18	GTP_EFTU_D2	Domain	62.5	2.40E-17	248	314	1	73
LOC_Os03g08020	PF00009.20	GTP_EFTU	Domain	188.6	5.60E-56	5	222	1	185
LOC_Os03g08050	PF03144.18	GTP_EFTU_D2	Domain	62.5	2.40E-17	248	314	1	73
LOC_Os03g08050	PF00009.20	GTP_EFTU	Domain	188.6	5.60E-56	5	222	1	185
LOC_Os03g08050	PF03143.10	GTP_EFTU_D3	Domain	112.7	6.60E-33	322	429	2	100
LOC_Os03g13800	PF01248.19	Ribosomal_L7Ae	Domain	91.4	1.50E-26	22	111	3	92
LOC_Os03g15900	PF00018.21	SH3_1	Domain	28.5	5.60E-07	986	1027	1	43
LOC_Os03g17000	PF04321.10	RmlD_sub_bind	Domain	51.8	4.00E-14	389	564	2	171
LOC_Os03g17000	PF04321.10	RmlD_sub_bind	Domain	42.5	2.80E-11	389	524	2	130
LOC_Os03g17084	PF00125.17	Histone	Domain	78.1	3.10E-22	61	128	3	74
LOC_Os03g17100	PF00125.17	Histone	Domain	86.1	1.10E-24	31	104	1	75
LOC_Os03g18410	PF01000.19	RNA_pol_A_bac	Domain	79.7	1.40E-22	117	257	3	111
LOC_Os03g18410	PF01193.17	RNA_pol_L	Domain	62.2	1.80E-17	84	374	1	84
LOC_Os03g18510	PF00575.16	S1	Domain	44	1.40E-11	17	88	5	74
LOC_Os03g21530	PF08142.5	AARP2CN	Domain	93.8	2.60E-27	235	321	1	84
LOC_Os03g21530	PF00009.20	GTP_EFTU	Domain	24.5	1.20E-05	126	226	66	171
LOC_Os03g22730	PF08156.6	NOP5NT	Domain	78.9	1.70E-22	1	66	1	67
LOC_Os03g22730	PF08060.6	NOSIC	Domain	101.4	1.30E-29	159	211	1	53
LOC_Os03g22740	PF08156.6	NOP5NT	Domain	78.9	1.70E-22	1	66	1	67
LOC_Os03g22740	PF08060.6	NOSIC	Domain	101.4	1.30E-29	159	211	1	53
LOC_Os03g22880	PF08156.6	NOP5NT	Domain	68.1	4.00E-19	3	69	1	67
LOC_Os03g22880	PF08060.6	NOSIC	Domain	81.8	1.70E-23	170	221	1	52
LOC_Os03g25450	PF08068.5	DKCLD	Domain	103.8	2.80E-30	66	124	1	59
LOC_Os03g31134	PF00098.16	zf-CCHC	Domain	26.2	3.60E-06	456	473	1	18
LOC_Os03g31134	PF00665.19	rve	Domain	108.7	1.50E-31	703	819	3	118
LOC_Os03g36930	PF00270.22	DEAD	Domain	147	2.60E-43	56	218	2	164
LOC_Os03g38000	PF07650.10	KH_2	Domain	31.5	9.30E-08	45	102	1	59
LOC_Os03g38000	PF00189.13	Ribosomal_S3_C	Domain	66.4	1.50E-18	105	188	2	85
LOC_Os03g38000	PF07650.10	KH_2	Domain	31.8	7.60E-08	22	79	1	59
LOC_Os03g38000	PF00189.13	Ribosomal_S3_C	Domain	66.8	1.20E-18	82	165	2	85
LOC_Os03g42110	PF01118.17	Semialdhyde_dh	Domain	102.1	1.80E-29	74	211	1	121
LOC_Os03g42110	PF02774.11	Semialdhyde_dhC	Domain	59.3	3.30E-16	228	386	1	184
LOC_Os03g46770	PF00076.15	RRM_1	Domain	82.1	1.20E-23	11	80	2	70
LOC_Os03g47800	PF00098.16	zf-CCHC	Domain	26.9	2.30E-06	115	131	2	18
LOC_Os03g47800	PF00076.15	RRM_1	Domain	68.6	2.00E-19	9	78	1	69
LOC_Os03g50090	PF00270.22	DEAD	Domain	168.3	7.70E-50	340	530	2	167

Additional File 3 cont.: Pfam Domain Assignment for Proteins Identified with two or more peptides

LOC_Os03g50290	PF00244.13	14-3-3	Domain	378.8	5.60E-114	8	244	1	235
LOC_Os03g50480	PF02878.9	PGM_PMM_I	Domain	116.4	4.90E-34	17	164	2	137
LOC_Os03g50480	PF02879.9	PGM_PMM_II	Domain	50.6	1.50E-13	200	308	2	101
LOC_Os03g50480	PF02880.9	PGM_PMM_III	Domain	90.7	4.70E-26	316	439	1	111
LOC_Os03g50480	PF02878.9	PGM_PMM_I	Domain	57.7	6.40E-16	2	87	64	137
LOC_Os03g50480	PF02880.9	PGM_PMM_III	Domain	91	3.70E-26	239	362	1	111
LOC_Os03g50480	PF02879.9	PGM_PMM_II	Domain	51	1.20E-13	123	231	2	101
LOC_Os03g51200	PF00125.17	Histone	Domain	91.6	2.00E-26	21	94	1	75
LOC_Os03g53190	PF00125.17	Histone	Domain	83.9	4.90E-24	32	106	1	75
LOC_Os03g53220	PF00270.22	DEAD	Domain	78.2	3.60E-22	1333	1500	2	161
LOC_Os03g53220	PF00270.22	DEAD	Domain	59.1	2.80E-16	513	627	2	111
LOC_Os03g55070	PF03721.7	UDPG_MGDP_dh_N	Domain	217.8	5.60E-65	3	185	2	180
LOC_Os03g55070	PF00984.12	UDPG_MGDP_dh	Domain	114.6	1.40E-33	209	306	1	96
LOC_Os03g55070	PF03720.8	UDPG_MGDP_dh_C	Domain	128.1	1.10E-37	328	452	1	106
LOC_Os03g58430	PF00203.14	Ribosomal_S19	Domain	98.5	9.50E-29	93	158	17	81
LOC_Os03g58530	PF01426.11	BAH	Domain	77.6	4.70E-22	24	138	3	117
LOC_Os03g58530	PF00628.22	PHD	Domain	42.2	3.70E-11	143	191	1	50
LOC_Os03g58530	PF00628.22	PHD	Domain	42.6	2.80E-11	111	159	1	50
LOC_Os03g58530	PF01426.11	BAH	Domain	22.6	5.30E-05	61	106	69	117
LOC_Os03g58810	PF00270.22	DEAD	Domain	167.9	9.70E-50	120	290	1	166
LOC_Os03g59310	PF00333.13	Ribosomal_S5	Domain	100	3.40E-29	85	150	2	67
LOC_Os03g59310	PF03719.8	Ribosomal_S5_C	Domain	81.6	1.40E-23	167	233	1	66
LOC_Os03g61990	PF00076.15	RRM_1	Domain	80.2	5.00E-23	10	78	2	69
LOC_Os03g61990	PF00098.16	zf-CCHC	Domain	34.2	1.10E-08	128	143	3	18
LOC_Os04g01740	PF02518.19	HATPase_c	Domain	37.7	1.10E-09	37	187	3	109
LOC_Os04g02820	PF00009.20	GTP_EFTU	Domain	200.4	1.30E-59	18	342	2	189
LOC_Os04g02820	PF03764.11	EFG_IV	Domain	126.2	3.60E-37	609	722	5	120
LOC_Os04g02820	PF00679.17	EFG_C	Domain	73	1.10E-20	724	811	1	87
LOC_Os04g02820	PF03144.18	GTP_EFTU_D2	Domain	49.9	1.90E-13	393	468	3	74
LOC_Os04g02820	PF00009.20	GTP_EFTU	Domain	201.2	7.40E-60	18	342	2	189
LOC_Os04g02820	PF03144.18	GTP_EFTU_D2	Domain	50.4	1.40E-13	393	468	3	74
LOC_Os04g25550	PF08512.5	Rtt106	Domain	34.4	1.30E-08	841	992	14	138
LOC_Os04g25550	PF00557.17	Peptidase_M24	Domain	101.9	2.90E-29	197	406	2	188
LOC_Os04g28090	PF00249.24	Myb_DNA-binding	Domain	44.7	7.90E-12	8	53	2	48
LOC_Os04g28090	PF00249.24	Myb_DNA-binding	Domain	32.7	4.50E-08	60	103	2	48
LOC_Os04g31320	PF02201.11	SWIB	Domain	71.1	3.60E-20	242	314	3	75
LOC_Os04g36890	PF00254.21	FKBP_C	Domain	95.7	9.70E-28	433	522	3	96
LOC_Os04g36890	PF00254.21	FKBP_C	Domain	96.6	5.00E-28	262	351	3	96
LOC_Os04g38870	PF00244.13	14-3-3	Domain	383.8	1.60E-115	9	245	1	235
LOC_Os04g39444	PF01423.15	LSM	Domain	62.6	1.30E-17	6	70	2	68
LOC_Os04g41040	PF01191.12	RNA_pol_Rpb5_C	Domain	132.9	1.70E-39	132	205	1	74
LOC_Os04g41040	PF03871.7	RNA_pol_Rpb5_N	Domain	99.9	6.20E-29	1	90	1	93
LOC_Os04g41040	PF03871.7	RNA_pol_Rpb5_N	Domain	100.6	3.70E-29	1	90	1	93
LOC_Os04g41040	PF01191.12	RNA_pol_Rpb5_C	Domain	43	2.00E-11	132	165	1	34
LOC_Os04g46920	PF00098.16	zf-CCHC	Domain	21.7	0.0001	149	164	3	18

Additional File 3 cont.: Pfam Domain Assignment for Proteins Identified with two or more peptides

LOC_Os04g46920	PF00098.16	zf-CCHC	Domain	10	0.49	96	111	2	17
LOC_Os04g46920	PF00098.16	zf-CCHC	Domain	15.4	0.01	71	87	2	18
LOC_Os04g46920	PF00098.16	zf-CCHC	Domain	19.3	0.00058	122	137	3	18
LOC_Os04g50660	PF08625.4	Utp13	Domain	146.1	4.00E-43	683	816	2	140
LOC_Os04g50990	PF03946.7	Ribosomal_L11_N	Domain	59	1.80E-16	15	70	4	59
LOC_Os04g50990	PF00298.12	Ribosomal_L11	Domain	50.8	1.00E-13	75	144	2	69
LOC_Os04g51630	PF00327.13	Ribosomal_L30	Domain	73.1	7.80E-21	85	136	1	52
LOC_Os04g51630	PF08079.5	Ribosomal_L30_N	Domain	80.2	6.40E-23	13	82	1	70
LOC_Os04g51630	PF00327.13	Ribosomal_L30	Domain	73.9	4.20E-21	13	64	1	52
LOC_Os04g52200	PF00076.15	RRM_1	Domain	55	3.70E-15	25	93	1	69
LOC_Os04g52200	PF00076.15	RRM_1	Domain	46.1	2.10E-12	376	440	5	69
LOC_Os04g52200	PF00076.15	RRM_1	Domain	68	3.10E-19	783	850	1	69
LOC_Os04g52200	PF00076.15	RRM_1	Domain	46.2	2.00E-12	684	758	1	69
LOC_Os04g52200	PF00076.15	RRM_1	Domain	29.4	3.50E-07	564	627	1	69
LOC_Os04g52960	PF00076.15	RRM_1	Domain	65.3	2.20E-18	451	519	1	70
LOC_Os04g52960	PF00076.15	RRM_1	Domain	42.1	3.80E-11	567	623	13	69
LOC_Os04g57010	PF00013.22	KH_1	Domain	47.2	9.80E-13	177	239	1	57
LOC_Os04g57010	PF00013.22	KH_1	Domain	48.1	5.40E-13	88	150	1	57
LOC_Os05g02300	PF00125.17	Histone	Domain	84.7	2.80E-24	28	101	1	75
LOC_Os05g04850	PF00076.15	RRM_1	Domain	58.1	3.90E-16	103	168	1	65
LOC_Os05g04850	PF00076.15	RRM_1	Domain	25.4	6.00E-06	103	142	1	45
LOC_Os05g06770	PF00240.16	ubiquitin	Domain	116.4	1.90E-34	6	74	1	69
LOC_Os05g06770	PF01599.12	Ribosomal_S27	Domain	87.4	3.10E-25	101	148	1	47
LOC_Os05g08360	PF01269.10	Fibrillarin	Domain	375.6	4.10E-113	74	300	2	228
LOC_Os05g08360	PF01269.10	Fibrillarin	Domain	303.4	4.70E-91	74	251	2	179
LOC_Os05g11710	PF00281.12	Ribosomal_L5	Domain	73.2	8.40E-21	9	62	1	56
LOC_Os05g11710	PF00673.14	Ribosomal_L5_C	Domain	77.5	3.80E-22	66	165	1	95
LOC_Os05g28280	PF00557.17	Peptidase_M24	Domain	100.6	6.90E-29	23	226	2	182
LOC_Os05g30530	PF08071.5	RS4NT	Domain	70.9	4.30E-20	3	40	1	38
LOC_Os05g38640	PF00125.17	Histone	Domain	85	2.30E-24	29	102	1	75
LOC_Os05g41172	PF02182.10	YDG_SRA	Domain	183.9	8.80E-55	213	367	2	155
LOC_Os05g48820	PF00046.22	Homeobox	Domain	59.7	1.20E-16	59	113	3	57
LOC_Os05g49030	PF01775.10	Ribosomal_L18ae	Domain	192.4	1.70E-57	7	128	2	123
LOC_Os05g49230	PF07780.5	Spb1_C	Domain	207.9	9.70E-62	587	790	3	216
LOC_Os05g49860	PF00125.17	Histone	Domain	79.7	1.00E-22	61	128	3	74
LOC_Os05g49890	PF00071.15	Ras	Domain	170.9	1.00E-50	15	171	1	160
LOC_Os05g49890	PF00071.15	Ras	Domain	171.4	7.20E-51	15	171	1	160
LOC_Os05g49890	PF00071.15	Ras	Domain	112.8	7.90E-33	15	118	1	104
LOC_Os05g51850	PF00538.12	Linker_histone	Domain	33.2	3.10E-08	21	79	2	62
LOC_Os05g51850	PF00538.12	Linker_histone	Domain	33.3	2.90E-08	21	79	2	62
LOC_Os06g03780	PF08159.5	NUC153	Domain	48.4	3.80E-13	501	528	1	28
LOC_Os06g05350	PF08536.4	Whirly	Domain	191.8	2.70E-57	97	234	1	138
LOC_Os06g06460	PF00125.17	Histone	Domain	112.2	7.40E-33	58	132	1	75
LOC_Os06g06480	PF00125.17	Histone	Domain	110.3	3.00E-32	172	246	1	75
LOC_Os06g06880	PF00149.21	Metallophos	Domain	155.1	1.10E-45	55	248	2	199

Additional File 3 cont.: Pfam Domain Assignment for Proteins Identified with two or more peptides

LOC_Os06g09570	PF08155.4	NOGCT	Domain	99	6.70E-29	411	465	1	55
LOC_Os06g12780	PF00089.19	Trypsin	Domain	58.8	4.30E-16	181	357	11	218
LOC_Os06g14470	PF00076.15	RRM_1	Domain	51.1	5.90E-14	112	180	1	69
LOC_Os06g16290	PF01248.19	Ribosomal_L7Ae	Domain	87	3.40E-25	33	117	4	88
LOC_Os06g40600	PF03144.18	GTP_EFTU_D2	Domain	46.7	2.00E-12	516	590	4	74
LOC_Os06g40600	PF00009.20	GTP_EFTU	Domain	153.1	4.40E-45	148	342	3	177
LOC_Os06g40600	PF00679.17	EFG_C	Domain	75.2	2.20E-21	850	938	1	88
LOC_Os06g40600	PF03764.11	EFG_IV	Domain	90.8	3.40E-26	733	848	11	120
LOC_Os06g40950	PF04998.10	RNA_pol_Rpb1_5	Domain	67.6	7.80E-19	114	293	129	273
LOC_Os06g41384	PF00013.22	KH_1	Domain	44.8	5.50E-12	168	230	1	57
LOC_Os06g43690	PF04192.5	Utp21	Domain	217.3	1.10E-64	668	900	7	235
LOC_Os06g45710	PF00162.12	PGK	Domain	547.4	1.00E-164	9	390	1	384
LOC_Os06g48750	PF00270.22	DEAD	Domain	145.6	7.20E-43	66	227	2	162
LOC_Os06g48750	PF00270.22	DEAD	Domain	146	5.50E-43	21	182	2	162
LOC_Os06g51220	PF00505.12	HMG_box	Domain	85.1	2.60E-24	42	111	1	69
LOC_Os07g01490	PF00225.16	Kinesin	Domain	349.4	1.10E-104	55	376	2	332
LOC_Os07g01490	PF00225.16	Kinesin	Domain	166.3	5.50E-49	54	258	1	215
LOC_Os07g01920	PF08155.4	NOGCT	Domain	99	6.70E-29	411	465	1	55
LOC_Os07g03240	PF00076.15	RRM_1	Domain	64.2	4.70E-18	22	78	1	56
LOC_Os07g03240	PF00076.15	RRM_1	Domain	61.7	2.80E-17	146	213	1	68
LOC_Os07g05580	PF01248.19	Ribosomal_L7Ae	Domain	103.4	2.60E-30	24	117	2	94
LOC_Os07g06980	PF00850.12	Hist_deacetyl	Domain	309.4	2.10E-92	223	514	9	305
LOC_Os07g07220	PF01423.15	LSM	Domain	58.3	3.00E-16	11	83	5	67
LOC_Os07g08660	PF00203.14	Ribosomal_S19	Domain	118.7	4.60E-35	56	137	1	81
LOC_Os07g08880	PF00628.22	PHD	Domain	41.2	7.50E-11	142	190	1	50
LOC_Os07g08880	PF01426.11	BAH	Domain	83.8	5.80E-24	23	137	3	117
LOC_Os07g08880	PF01426.11	BAH	Domain	83.8	5.70E-24	23	137	3	117
LOC_Os07g08880	PF00628.22	PHD	Domain	41.2	7.40E-11	142	190	1	50
LOC_Os07g08880	PF01426.11	BAH	Domain	84.9	2.60E-24	23	137	3	117
LOC_Os07g08880	PF01426.11	BAH	Domain	85.6	1.50E-24	23	137	3	117
LOC_Os07g08960	PF00098.16	zf-CCHC	Domain	27.5	1.50E-06	127	141	3	17
LOC_Os07g08960	PF00076.15	RRM_1	Domain	73.6	5.60E-21	9	78	1	69
LOC_Os07g10350	PF00575.16	S1	Domain	24.3	1.80E-05	232	297	3	66
LOC_Os07g10350	PF00575.16	S1	Domain	52.4	3.30E-14	428	500	2	73
LOC_Os07g10660	PF03719.8	Ribosomal_S5_C	Domain	82	1.00E-23	170	236	1	66
LOC_Os07g10660	PF00333.13	Ribosomal_S5	Domain	98.4	1.10E-28	88	153	2	67
LOC_Os07g12650	PF01248.19	Ribosomal_L7Ae	Domain	91.1	1.80E-26	63	146	2	85
LOC_Os07g12910	PF00628.22	PHD	Domain	39.4	2.80E-10	190	238	2	50
LOC_Os07g20580	PF00270.22	DEAD	Domain	157.7	1.40E-46	117	288	2	166
LOC_Os07g25440	PF08145.5	BOP1NT	Domain	347.4	4.60E-104	78	334	1	260
LOC_Os07g25440	PF08145.5	BOP1NT	Domain	347.5	4.20E-104	53	309	1	260
LOC_Os07g33340	PF00270.22	DEAD	Domain	153.4	2.90E-45	105	275	2	167
LOC_Os07g36130	PF00125.17	Histone	Domain	92.8	8.50E-27	19	92	1	75
LOC_Os07g36140	PF00125.17	Histone	Domain	92.8	8.50E-27	19	92	1	75
LOC_Os07g36500	PF00125.17	Histone	Domain	52.8	2.60E-14	28	94	4	75

LOC_Os07g40930	PF08154.5	NLE	Domain	69	1.90E-19	12	80	1	65
LOC_Os07g41740	PF00628.22	PHD	Domain	39.1	3.50E-10	212	259	2	49
LOC_Os07g41750	PF00189.13	Ribosomal_S3_C	Domain	60.8	8.50E-17	107	190	2	85
LOC_Os07g41750	PF07650.10	KH_2	Domain	33.4	2.40E-08	47	104	1	59
LOC_Os07g41750	PF00189.13	Ribosomal_S3_C	Domain	61.1	6.60E-17	82	165	2	85
LOC_Os07g41750	PF07650.10	KH_2	Domain	33.7	1.90E-08	22	79	1	59
LOC_Os07g41750	PF00189.13	Ribosomal_S3_C	Domain	61.5	5.20E-17	11	92	4	85
LOC_Os07g43980	PF00270.22	DEAD	Domain	146.7	3.40E-43	101	268	1	165
LOC_Os07g44190	PF08068.5	DKCLD	Domain	103.3	3.90E-30	62	120	1	59
LOC_Os07g46720	PF08060.6	NOSIC	Domain	84.9	1.80E-24	170	220	1	51
LOC_Os07g46720	PF08156.6	NOP5NT	Domain	67.2	7.80E-19	3	69	1	67
LOC_Os07g46720	PF08060.6	NOSIC	Domain	84.9	1.80E-24	170	220	1	51
LOC_Os08g02400	PF08069.5	Ribosomal_S13_N	Domain	102.9	4.60E-30	1	60	1	60
LOC_Os08g02400	PF00312.15	Ribosomal_S15	Domain	82.9	7.80E-24	66	148	2	80
LOC_Os08g02410	PF00312.15	Ribosomal_S15	Domain	82.9	7.80E-24	66	148	2	80
LOC_Os08g02410	PF08069.5	Ribosomal_S13_N	Domain	103.6	2.70E-30	1	60	1	60
LOC_Os08g03520	PF00313.15	CSD	Domain	91.1	2.10E-26	5	71	1	66
LOC_Os08g03520	PF00098.16	zf-CCHC	Domain	33.4	2.00E-08	135	151	1	17
LOC_Os08g03520	PF00098.16	zf-CCHC	Domain	35.5	4.30E-09	178	194	2	18
LOC_Os08g05840	PF01028.13	Topoisom_I	Domain	294.3	3.40E-88	586	815	1	234
LOC_Os08g05880	PF04427.11	Brix	Domain	160.6	2.60E-47	88	262	2	191
LOC_Os08g09350	PF00076.15	RRM_1	Domain	46.8	1.30E-12	413	485	1	69
LOC_Os08g09350	PF00076.15	RRM_1	Domain	63.5	7.70E-18	313	381	1	70
LOC_Os08g13690	PF00327.13	Ribosomal_L30	Domain	73	7.90E-21	86	137	1	52
LOC_Os08g13690	PF08079.5	Ribosomal_L30_N	Domain	86.4	7.60E-25	14	84	1	71
LOC_Os08g23710	PF01248.19	Ribosomal_L7Ae	Domain	87.3	2.70E-25	115	204	3	91
LOC_Os08g29650	PF00076.15	RRM_1	Domain	69.9	8.10E-20	93	162	1	69
LOC_Os08g31240	PF00557.17	Peptidase_M24	Domain	99.2	1.90E-28	166	346	23	187
LOC_Os08g31810	PF00076.15	RRM_1	Domain	55.5	2.50E-15	56	125	1	69
LOC_Os08g32090	PF08147.5	DBP10CT	Domain	56.8	1.10E-15	646	706	1	65
LOC_Os08g32090	PF00270.22	DEAD	Domain	162	6.30E-48	73	240	1	165
LOC_Os08g33100	PF00125.17	Histone	Domain	92.8	8.30E-27	19	92	1	75
LOC_Os08g33120	PF00076.15	RRM_1	Domain	49.7	1.70E-13	92	160	1	69
LOC_Os08g33120	PF00076.15	RRM_1	Domain	50.1	1.20E-13	44	112	1	69
LOC_Os08g33120	PF00076.15	RRM_1	Domain	50.4	1.00E-13	44	112	1	69
LOC_Os08g33120	PF00076.15	RRM_1	Domain	51.2	5.40E-14	44	112	1	69
LOC_Os08g33370	PF00244.13	14-3-3	Domain	382	5.70E-115	3	239	1	235
LOC_Os08g37490	PF00244.13	14-3-3	Domain	370.3	2.10E-111	10	249	1	235
LOC_Os08g38300	PF00125.17	Histone	Domain	79.5	1.20E-22	59	126	3	74
LOC_Os08g38410	PF00076.15	RRM_1	Domain	59.9	1.10E-16	113	175	1	62
LOC_Os08g38410	PF00076.15	RRM_1	Domain	58.6	2.70E-16	25	92	1	69
LOC_Os08g38410	PF00076.15	RRM_1	Domain	60.1	8.90E-17	59	121	1	62
LOC_Os08g38410	PF00076.15	RRM_1	Domain	59	2.00E-16	25	92	1	69
LOC_Os08g38410	PF00076.15	RRM_1	Domain	60.5	7.10E-17	113	176	1	63
LOC_Os08g39140	PF02518.19	HATPase_c	Domain	40.6	1.30E-10	30	180	3	109

Additional File 3 cont.: Pfam Domain Assignment for Proteins Identified with two or more peptides

LOC_Os08g39140	PF02518.19	HATPase_c	Domain	40.8	1.20E-10	30	180	3	109
LOC_Os08g39140	PF02518.19	HATPase_c	Domain	41.5	7.10E-11	30	180	3	109
LOC_Os08g41810	PF00237.12	Ribosomal_L22	Domain	99.1	9.60E-29	38	165	10	105
LOC_Os08g42920	PF08079.5	Ribosomal_L30_N	Domain	52.2	3.60E-14	16	82	1	69
LOC_Os08g42920	PF00327.13	Ribosomal_L30	Domain	45.7	2.70E-12	90	140	2	52
LOC_Os08g44380	PF00687.14	Ribosomal_L1	Domain	184.3	1.40E-54	8	212	4	209
LOC_Os08g44450	PF00687.14	Ribosomal_L1	Domain	183	3.50E-54	9	212	5	209
LOC_Os08g44450	PF00687.14	Ribosomal_L1	Domain	110.4	5.80E-32	2	129	83	209
LOC_Os09g02284	PF01000.19	RNA_pol_A_bac	Domain	105.4	1.40E-30	57	196	1	111
LOC_Os09g02284	PF01193.17	RNA_pol_L	Domain	63.5	7.40E-18	27	310	2	83
LOC_Os09g08430	PF00237.12	Ribosomal_L22	Domain	117.2	2.20E-34	17	153	1	105
LOC_Os09g10770	PF02518.19	HATPase_c	Domain	32	6.20E-08	77	243	5	97
LOC_Os09g10770	PF09239.4	Topo-VIb_trans	Domain	180.8	1.00E-53	425	584	1	160
LOC_Os09g23730	PF00538.12	Linker_histone	Domain	47.7	9.50E-13	22	84	2	65
LOC_Os09g30412	PF02518.19	HATPase_c	Domain	39.1	3.80E-10	30	180	3	109
LOC_Os09g30418	PF02518.19	HATPase_c	Domain	38.7	5.00E-10	30	180	3	109
LOC_Os09g31180	PF00347.16	Ribosomal_L6	Domain	53.1	2.60E-14	98	177	1	77
LOC_Os09g31180	PF00347.16	Ribosomal_L6	Domain	53.7	1.70E-14	12	86	1	77
LOC_Os09g31180	PF00347.16	Ribosomal_L6	Domain	54	1.30E-14	12	86	1	77
LOC_Os09g31180	PF00347.16	Ribosomal_L6	Domain	26.5	5.40E-06	110	160	30	77
LOC_Os10g28230	PF00125.17	Histone	Domain	80.6	5.20E-23	34	108	1	75
LOC_Os10g30580	PF02933.10	CDC48_2	Domain	36.7	1.80E-09	133	195	2	61
LOC_Os10g30580	PF02359.11	CDC48_N	Domain	89	1.10E-25	32	115	1	87
LOC_Os10g32880	PF08606.4	Prp19	Domain	109.5	3.90E-32	35	102	2	69
LOC_Os10g35290	PF00562.21	RNA_pol_Rpb2_6	Domain	367.9	3.80E-110	543	907	1	385
LOC_Os10g35290	PF04560.13	RNA_pol_Rpb2_7	Domain	74.3	5.30E-21	910	1020	1	81
LOC_Os10g35290	PF06883.5	RNA_pol_Rpa2_4	Domain	52.6	2.40E-14	451	487	20	58
LOC_Os10g35290	PF04565.9	RNA_pol_Rpb2_3	Domain	85.9	8.90E-25	327	391	3	68
LOC_Os10g35290	PF04561.7	RNA_pol_Rpb2_2	Domain	33.9	1.60E-08	37	210	16	188
LOC_Os11g29190	PF00177.14	Ribosomal_S7	Domain	115.1	1.50E-33	45	182	2	133
LOC_Os11g29190	PF00177.14	Ribosomal_S7	Domain	141.5	1.10E-41	45	199	2	148
LOC_Os11g29190	PF00177.14	Ribosomal_S7	Domain	125.1	1.30E-36	3	128	31	148
LOC_Os11g29190	PF00177.14	Ribosomal_S7	Domain	38.7	5.30E-10	45	105	2	59
LOC_Os11g29190	PF00177.14	Ribosomal_S7	Domain	34.6	9.90E-09	45	101	2	55
LOC_Os11g34450	PF00244.13	14-3-3	Domain	374.6	1.00E-112	8	248	1	235
LOC_Os11g36390	PF08519.5	RFC1	Domain	162.1	6.40E-48	735	898	1	155
LOC_Os11g38900	PF02182.10	YDG_SRA	Domain	174.5	6.60E-52	357	506	2	155
LOC_Os11g43900	PF00838.10	TCTP	Domain	221.7	4.20E-66	1	165	1	165
LOC_Os11g43900	PF00838.10	TCTP	Domain	165.4	8.40E-49	1	129	1	129
LOC_Os12g25120	PF00125.17	Histone	Domain	92.8	8.50E-27	19	92	1	75
LOC_Os12g25690	PF03720.8	UDPG_MGDP_dh_C	Domain	127.5	1.60E-37	328	451	1	105
LOC_Os12g25690	PF03721.7	UDPG_MGDP_dh_N	Domain	216.7	1.30E-64	3	185	2	180
LOC_Os12g25690	PF00984.12	UDPG_MGDP_dh	Domain	113	4.30E-33	209	306	1	96
LOC_Os12g34510	PF00125.17	Histone	Domain	91.6	2.00E-26	21	94	1	75
LOC_Os12g38000	PF03947.11	Ribosomal_L2_C	Domain	138.6	7.60E-41	97	230	2	129

Additional File 3 cont.: Pfam Domain Assignment for Proteins Identified with two or more peptides

LOC_Os12g38000	PF00181.16	Ribosomal_L2	Domain	60	1.30E-16	13	90	3	77
LOC_Os12g41715	PF00270.22	DEAD	Domain	162.8	3.60E-48	122	290	1	166
LOC_Os12g41930	PF05022.5	SRP40_C	Domain	102	1.40E-29	369	442	2	72
LOC_Os12g44390	PF06470.6	SMC_hinge	Domain	34.8	1.00E-08	524	569	2	46
LOC_Os01g01510	PF04000.8	Sas10_Utp3	Family	53.5	1.60E-14	229	308	9	85
LOC_Os01g01510	PF04000.8	Sas10_Utp3	Family	53.6	1.50E-14	229	308	9	85
LOC_Os01g07810	PF01918.14	Alba	Family	43.5	1.40E-11	43	105	1	70
LOC_Os01g08970	PF03531.7	SSrecog	Family	335.2	9.30E-101	218	435	1	216
LOC_Os01g08970	PF03531.7	SSrecog	Family	335.7	6.30E-101	112	329	1	216
LOC_Os01g13730	PF04158.7	Sof1	Family	102.4	7.70E-30	361	447	1	87
LOC_Os01g14950	PF01749.13	IBB	Family	84.8	2.60E-24	5	95	2	97
LOC_Os01g16010	PF05700.4	BCAS2	Family	284.6	3.70E-85	31	238	7	215
LOC_Os01g16220	PF07738.6	Sad1_UNC	Family	125.8	7.50E-37	306	443	6	134
LOC_Os01g16290	PF00986.14	DNA_gyraseB_C	Family	93.2	4.70E-27	654	716	3	65
LOC_Os01g16870	PF02170.15	PAZ	Family	109.5	6.20E-32	274	410	2	133
LOC_Os01g16870	PF02171.10	Piwi	Family	342.7	1.10E-102	558	865	2	304
LOC_Os01g16870	PF02170.15	PAZ	Family	109.5	6.20E-32	274	410	2	133
LOC_Os01g16870	PF02171.10	Piwi	Family	342.7	1.10E-102	558	865	2	304
LOC_Os01g16870	PF02170.15	PAZ	Family	109.5	6.20E-32	274	410	2	133
LOC_Os01g24690	PF03939.6	Ribosomal_L23eN	Family	69.5	1.40E-19	13	63	3	53
LOC_Os01g24690	PF00276.13	Ribosomal_L23	Family	67.3	6.50E-19	70	148	1	91
LOC_Os01g24690	PF00276.13	Ribosomal_L23	Family	54.9	5.00E-15	70	125	1	56
LOC_Os01g24690	PF00276.13	Ribosomal_L23	Family	67.7	4.90E-19	55	133	1	91
LOC_Os01g24690	PF03939.6	Ribosomal_L23eN	Family	66.7	1.10E-18	2	48	7	53
LOC_Os01g25610	PF00900.13	Ribosomal_S4e	Family	104.6	1.20E-30	94	170	1	77
LOC_Os01g25610	PF01479.18	S4	Family	28.6	5.40E-07	50	90	8	48
LOC_Os01g25610	PF00467.22	KOW	Family	28.8	4.40E-07	177	211	1	32
LOC_Os01g27730	PF08701.4	GN3L_Grn1	Family	68.4	2.90E-19	165	241	1	76
LOC_Os01g27730	PF01926.16	MMR_HSR1	Family	42.9	2.60E-11	429	490	1	68
LOC_Os01g33050	PF01246.13	Ribosomal_L24e	Family	107.2	2.10E-31	1	67	1	67
LOC_Os01g36860	PF00271.24	Helicase_C	Family	92.9	6.00E-27	418	489	7	78
LOC_Os01g36860	PF00271.24	Helicase_C	Family	93.3	4.60E-27	277	348	7	78
LOC_Os01g36860	PF00271.24	Helicase_C	Family	93.5	3.90E-27	209	280	7	78
LOC_Os01g36890	PF00271.24	Helicase_C	Family	93.4	4.30E-27	224	300	2	78
LOC_Os01g45190	PF00271.24	Helicase_C	Family	92.2	1.00E-26	290	365	3	78
LOC_Os01g45190	PF00271.24	Helicase_C	Family	93	5.50E-27	172	247	3	78
LOC_Os01g46060	PF04003.5	Utp12	Family	58.8	3.20E-16	445	547	3	108
LOC_Os01g51300	PF12265.1	CAF1C_H4-bd	Family	69.4	1.40E-19	11	80	1	73
LOC_Os01g52490	PF01282.12	Ribosomal_S24e	Family	125.5	4.20E-37	28	110	1	83
LOC_Os01g59500	PF03998.6	Utp11	Family	208.6	9.60E-62	10	229	1	242
LOC_Os01g59990	PF01246.13	Ribosomal_L24e	Family	123.1	2.30E-36	3	73	1	71
LOC_Os01g62040	PF06068.6	TIP49	Family	629.5	1.40E-189	14	414	1	397
LOC_Os01g62040	PF06068.6	TIP49	Family	521	1.30E-156	14	339	1	322
LOC_Os01g62040	PF06068.6	TIP49	Family	245.2	7.40E-73	14	186	1	172
LOC_Os01g67134	PF00861.15	Ribosomal_L18p	Family	136.9	2.70E-40	27	170	1	119

Additional File 3 cont.: Pfam Domain Assignment for Proteins Identified with two or more peptides

LOC_Os01g68320	PF00271.24	Helicase_C	Family	88	2.20E-25	515	591	2	78
LOC_Os01g68320	PF00271.24	Helicase_C	Family	30.8	1.50E-07	515	560	2	47
LOC_Os01g68950	PF11976.1	Rad60-SLD	Family	88.2	1.60E-25	21	91	1	72
LOC_Os02g01560	PF00900.13	Ribosomal_S4e	Family	103.9	2.00E-30	94	170	1	77
LOC_Os02g01560	PF00467.22	KOW	Family	28.8	4.40E-07	177	211	1	32
LOC_Os02g01560	PF01479.18	S4	Family	28.6	5.40E-07	50	90	8	48
LOC_Os02g01740	PF02889.9	Sec63	Family	321.8	3.30E-96	1849	2168	1	311
LOC_Os02g01740	PF00271.24	Helicase_C	Family	31.9	6.80E-08	816	894	9	77
LOC_Os02g01740	PF02889.9	Sec63	Family	360.4	6.10E-108	1017	1319	2	310
LOC_Os02g02410	PF00012.13	HSP70	Family	894.3	2.00E-269	35	642	1	602
LOC_Os02g04040	PF02463.12	SMC_N	Family	94.7	3.60E-27	144	469	135	217
LOC_Os02g04050	PF02463.12	SMC_N	Family	112.9	9.30E-33	2	133	1	135
LOC_Os02g04480	PF03178.8	CPSF_A	Family	328.5	3.00E-98	492	811	2	319
LOC_Os02g05330	PF00271.24	Helicase_C	Family	88.5	1.50E-25	300	375	3	78
LOC_Os02g06584	PF00642.17	zf-CCCH	Family	22.8	4.00E-05	11	34	1	26
LOC_Os02g06584	PF00642.17	zf-CCCH	Family	23.4	2.70E-05	11	34	1	26
LOC_Os02g06584	PF00642.17	zf-CCCH	Family	23.5	2.50E-05	11	34	1	26
LOC_Os02g06700	PF00411.12	Ribosomal_S11	Family	110.6	3.10E-32	28	129	1	93
LOC_Os02g06700	PF00411.12	Ribosomal_S11	Family	140.9	1.20E-41	28	146	1	110
LOC_Os02g07890	PF00828.12	Ribosomal_L18e	Family	115.3	1.70E-33	20	142	1	128
LOC_Os02g07890	PF00828.12	Ribosomal_L18e	Family	43.7	2.50E-11	58	105	82	128
LOC_Os02g07890	PF00828.12	Ribosomal_L18e	Family	30.2	3.70E-07	20	56	1	41
LOC_Os02g10080	PF00642.17	zf-CCCH	Family	35.6	3.90E-09	266	291	1	27
LOC_Os02g10080	PF00642.17	zf-CCCH	Family	24.6	1.10E-05	101	126	2	27
LOC_Os02g10080	PF00642.17	zf-CCCH	Family	25.1	7.70E-06	32	57	2	27
LOC_Os02g10080	PF00642.17	zf-CCCH	Family	36.1	2.80E-09	197	222	1	27
LOC_Os02g10640	PF00004.22	AAA	Family	143.2	3.80E-42	177	310	1	130
LOC_Os02g13530	PF01282.12	Ribosomal_S24e	Family	125.7	3.60E-37	28	110	1	83
LOC_Os02g18380	PF01777.11	Ribosomal_L27e	Family	106	6.30E-31	52	137	1	85
LOC_Os02g18380	PF00467.22	KOW	Family	24.4	1.10E-05	7	38	1	30
LOC_Os02g18550	PF01015.11	Ribosomal_S3Ae	Family	275.5	1.40E-82	12	222	2	195
LOC_Os02g18660	PF04504.7	DUF573	Family	42.3	5.50E-11	203	292	7	98
LOC_Os02g28810	PF01201.15	Ribosomal_S8e	Family	174.7	7.00E-52	1	216	1	132
LOC_Os02g33140	PF00411.12	Ribosomal_S11	Family	140.9	1.20E-41	29	147	1	110
LOC_Os02g37862	PF01159.12	Ribosomal_L6e	Family	140.7	1.50E-41	112	219	1	108
LOC_Os02g40514	PF04135.5	Nop10p	Family	74.7	2.60E-21	3	53	1	53
LOC_Os02g43930	PF01556.11	DnaJ_C	Family	89.1	1.20E-25	251	344	1	92
LOC_Os02g43930	PF00684.12	DnaJ_CXXCXGXG	Family	65.1	2.90E-18	135	214	1	79
LOC_Os02g49270	PF01189.10	Nol1_Nop2_Fmu	Family	293.8	9.90E-88	253	539	2	283
LOC_Os02g52250	PF02731.8	SKIP_SNW	Family	221.1	3.60E-66	191	350	2	158
LOC_Os02g52950	PF04504.7	DUF573	Family	43.4	2.50E-11	94	182	3	97
LOC_Os02g54340	PF00004.22	AAA	Family	134.7	1.70E-39	205	337	1	129
LOC_Os02g54340	PF00004.22	AAA	Family	134.8	1.50E-39	205	337	1	129
LOC_Os02g56014	PF04758.7	Ribosomal_S30	Family	105.6	6.00E-31	3	60	1	58
LOC_Os03g01970	PF11957.1	efThoc1	Family	170.9	2.40E-50	125	246	1	122

Additional File 3 cont.: Pfam Domain Assignment for Proteins Identified with two or more peptides

LOC_Os03g01970	PF11957.1	efThoc1	Family	223.3	2.90E-66	248	511	163	462
LOC_Os03g05720	PF04003.5	Utp12	Family	77	7.20E-22	789	892	2	108
LOC_Os03g05730	PF00004.22	AAA	Family	158	1.00E-46	247	376	1	129
LOC_Os03g05730	PF00004.22	AAA	Family	158.7	6.40E-47	520	653	1	130
LOC_Os03g05980	PF00163.12	Ribosomal_S4	Family	90.7	4.60E-26	7	107	1	93
LOC_Os03g05980	PF01479.18	S4	Family	52.6	1.70E-14	109	153	1	45
LOC_Os03g05980	PF00163.12	Ribosomal_S4	Family	91.5	2.60E-26	7	107	1	93
LOC_Os03g05980	PF01479.18	S4	Family	29	4.00E-07	109	136	1	28
LOC_Os03g08440	PF00318.13	Ribosomal_S2	Family	137.3	3.10E-40	22	189	1	210
LOC_Os03g08440	PF00318.13	Ribosomal_S2	Family	137.8	2.20E-40	22	189	1	210
LOC_Os03g08810	PF11523.1	DUF3223	Family	83.7	6.10E-24	106	179	2	76
LOC_Os03g10340	PF01015.11	Ribosomal_S3Ae	Family	278.4	1.70E-83	12	222	2	195
LOC_Os03g14530	PF00338.15	Ribosomal_S10	Family	103.4	3.40E-30	28	124	1	97
LOC_Os03g17000	PF01370.14	Epimerase	Family	191	1.70E-56	9	251	1	238
LOC_Os03g18510	PF07541.5	EIF_2_alpha	Family	157.1	9.60E-47	126	256	2	114
LOC_Os03g18570	PF01251.11	Ribosomal_S7e	Family	273.5	5.30E-82	6	190	1	188
LOC_Os03g18580	PF01251.11	Ribosomal_S7e	Family	272.8	8.90E-82	6	190	1	188
LOC_Os03g18840	PF04003.5	Utp12	Family	56.1	2.20E-15	490	585	11	110
LOC_Os03g21530	PF04950.5	DUF663	Family	262.6	2.70E-78	698	1016	6	297
LOC_Os03g21940	PF01280.13	Ribosomal_L19e	Family	218.5	2.30E-65	3	149	2	148
LOC_Os03g22180	PF00828.12	Ribosomal_L18e	Family	79.8	1.70E-22	6	122	5	129
LOC_Os03g22320	PF04615.6	Utp14	Family	635	1.20E-190	124	843	2	734
LOC_Os03g22460	PF01249.11	Ribosomal_S21e	Family	115.8	4.70E-34	1	62	1	62
LOC_Os03g22730	PF01798.11	Nop	Family	202	2.20E-60	252	399	1	149
LOC_Os03g22740	PF01798.11	Nop	Family	202	2.20E-60	252	399	1	149
LOC_Os03g22880	PF01798.11	Nop	Family	205.5	1.80E-61	266	412	1	148
LOC_Os03g25450	PF01509.11	TruB_N	Family	71.7	5.40E-20	128	244	1	149
LOC_Os03g25450	PF01472.13	PUA	Family	74.3	3.70E-21	315	387	1	73
LOC_Os03g26630	PF02037.20	SAP	Family	36.8	1.50E-09	13	42	2	31
LOC_Os03g27260	PF01092.12	Ribosomal_S6e	Family	188.6	2.30E-56	1	129	1	127
LOC_Os03g28410	PF00318.13	Ribosomal_S2	Family	40.4	1.40E-10	94	137	143	186
LOC_Os03g28410	PF00318.13	Ribosomal_S2	Family	45.9	3.00E-12	20	89	28	98
LOC_Os03g31090	PF01090.12	Ribosomal_S19e	Family	196.2	1.20E-58	8	141	2	135
LOC_Os03g31134	PF01090.12	Ribosomal_S19e	Family	190.3	7.50E-57	8	144	2	138
LOC_Os03g31134	PF07727.7	RVT_2	Family	330.8	2.60E-99	1036	1280	1	245
LOC_Os03g36930	PF00271.24	Helicase_C	Family	92.2	1.00E-26	290	365	3	78
LOC_Os03g37970	PF01294.11	Ribosomal_L13e	Family	309.8	3.20E-93	6	185	1	179
LOC_Os03g46490	PF01249.11	Ribosomal_S21e	Family	133.9	1.10E-39	1	77	1	77
LOC_Os03g49210	PF06732.4	Pescadillo_N	Family	366.8	5.20E-110	10	266	3	262
LOC_Os03g49210	PF00533.19	BRCT	Family	41.5	8.70E-11	340	414	4	78
LOC_Os03g50090	PF00271.24	Helicase_C	Family	95.8	7.60E-28	597	673	2	78
LOC_Os03g50480	PF00408.13	PGM_PMM_IV	Family	41.6	7.40E-11	492	546	22	60
LOC_Os03g50480	PF00408.13	PGM_PMM_IV	Family	41.8	6.00E-11	415	469	22	60
LOC_Os03g53220	PF02889.9	Sec63	Family	349.4	1.30E-104	986	1290	2	312
LOC_Os03g53220	PF02889.9	Sec63	Family	313.9	8.70E-94	1819	2135	2	311

Additional File 3 cont.: Pfam Domain Assignment for Proteins Identified with two or more peptides

LOC_Os03g53220	PF00271.24	Helicase_C	Family	26.7	2.80E-06	786	863	10	77
LOC_Os03g54890	PF00572.11	Ribosomal_L13	Family	96.4	9.70E-28	12	125	2	126
LOC_Os03g54890	PF00572.11	Ribosomal_L13	Family	96.9	6.80E-28	12	125	2	126
LOC_Os03g55150	PF01287.13	eIF-5a	Family	103.5	3.30E-30	99	168	1	69
LOC_Os03g55150	PF00467.22	KOW	Family	21.4	0.0001	30	50	1	26
LOC_Os03g55150	PF01287.13	eIF-5a	Family	103.7	2.70E-30	86	155	1	69
LOC_Os03g55150	PF01287.13	eIF-5a	Family	22.6	5.60E-05	86	103	1	18
LOC_Os03g55150	PF00467.22	KOW	Family	22.1	6.20E-05	30	50	1	26
LOC_Os03g58204	PF00573.15	Ribosomal_L4	Family	146.7	3.90E-43	26	266	2	191
LOC_Os03g58810	PF00271.24	Helicase_C	Family	74.6	3.20E-21	367	442	2	77
LOC_Os03g61640	PF12171.1	zf-C2H2_jaz	Family	28.6	8.30E-07	82	107	2	27
LOC_Os03g63670	PF07842.5	GCFC	Family	125.7	1.80E-36	651	856	1	271
LOC_Os04g01740	PF00183.11	HSP90	Family	853.3	4.50E-257	192	703	1	531
LOC_Os04g25550	PF08644.4	SPT16	Family	177.5	1.20E-52	543	694	1	152
LOC_Os04g28090	PF11831.1	DUF3351	Family	58.2	5.30E-16	263	358	2	107
LOC_Os04g28090	PF11831.1	DUF3351	Family	458	1.80E-137	322	730	2	379
LOC_Os04g28180	PF01201.15	Ribosomal_S8e	Family	174.5	7.90E-52	1	199	1	132
LOC_Os04g42140	PF02854.12	MIF4G	Family	197.5	1.30E-58	208	435	1	209
LOC_Os04g42140	PF02847.10	MA3	Family	72.1	2.20E-20	629	739	2	112
LOC_Os04g42270	PF03939.6	Ribosomal_L23eN	Family	76.4	9.50E-22	13	63	3	53
LOC_Os04g42270	PF00276.13	Ribosomal_L23	Family	67.3	6.50E-19	70	148	1	91
LOC_Os04g48060	PF08542.4	Rep_fac_C	Family	96.3	6.30E-28	237	325	1	90
LOC_Os04g48060	PF00004.22	AAA	Family	54.7	8.70E-15	53	171	1	127
LOC_Os04g49580	PF03914.10	CBF	Family	157.7	1.30E-46	329	491	1	164
LOC_Os04g49580	PF03914.10	CBF	Family	32	5.80E-08	329	377	1	51
LOC_Os04g49580	PF03914.10	CBF	Family	38.7	5.00E-10	386	433	117	164
LOC_Os04g56350	PF05997.5	Nop52	Family	207.3	1.40E-61	13	223	2	216
LOC_Os04g57010	PF00642.17	zf-CCCH	Family	38.3	5.80E-10	276	301	1	27
LOC_Os04g57010	PF00642.17	zf-CCCH	Family	25.8	4.90E-06	105	130	2	27
LOC_Os04g57010	PF00642.17	zf-CCCH	Family	26.4	3.10E-06	16	41	2	27
LOC_Os04g57010	PF00642.17	zf-CCCH	Family	38.9	3.70E-10	187	212	1	27
LOC_Os04g58830	PF04939.5	RRS1	Family	173.6	1.70E-51	14	179	1	164
LOC_Os05g01450	PF01398.14	Mov34	Family	62.5	2.20E-17	17	122	4	114
LOC_Os05g06310	PF00828.12	Ribosomal_L18e	Family	82.7	2.20E-23	6	123	5	129
LOC_Os05g06350	PF01749.13	IBB	Family	86.4	8.30E-25	5	101	2	97
LOC_Os05g07700	PF00252.11	Ribosomal_L16	Family	113.9	3.40E-33	5	166	1	132
LOC_Os05g07700	PF00252.11	Ribosomal_L16	Family	114.2	2.70E-33	5	166	1	132
LOC_Os05g07700	PF00252.11	Ribosomal_L16	Family	115.1	1.40E-33	5	166	1	132
LOC_Os05g09620	PF08514.4	STAG	Family	136.4	2.60E-40	143	251	5	116
LOC_Os05g09620	PF08514.4	STAG	Family	136.7	2.20E-40	5	113	5	116
LOC_Os05g10620	PF02365.8	NAM	Family	109.9	7.60E-32	57	211	1	128
LOC_Os05g10620	PF02365.8	NAM	Family	109.9	7.50E-32	48	202	1	128
LOC_Os05g22920	PF06862.5	DUF1253	Family	506.6	3.40E-152	234	663	1	442
LOC_Os05g22920	PF06862.5	DUF1253	Family	456.4	5.60E-137	154	569	1	442
LOC_Os05g22920	PF06862.5	DUF1253	Family	135.8	1.00E-39	234	367	1	142

Additional File 3 cont.: Pfam Domain Assignment for Proteins Identified with two or more peptides

LOC_Os05g27940	PF01251.11	Ribosomal_S7e	Family	268.9	1.40E-80	6	190	1	188
LOC_Os05g30530	PF01479.18	S4	Family	27.3	1.40E-06	47	90	6	48
LOC_Os05g30530	PF00900.13	Ribosomal_S4e	Family	109.2	4.50E-32	94	170	1	77
LOC_Os05g30530	PF00467.22	KOW	Family	28.7	4.80E-07	177	211	1	32
LOC_Os05g30880	PF04935.5	SURF6	Family	81.2	4.90E-23	194	371	11	209
LOC_Os05g40820	PF01246.13	Ribosomal_L24e	Family	123.1	2.30E-36	3	73	1	71
LOC_Os05g41172	PF00856.21	SET	Family	95.6	2.30E-27	513	646	1	158
LOC_Os05g44320	PF04003.5	Utp12	Family	108.2	1.40E-31	778	883	1	108
LOC_Os05g48820	PF02791.10	DDT	Family	80.5	4.30E-23	618	677	1	61
LOC_Os05g49230	PF11861.1	DUF3381	Family	150	2.80E-44	231	383	2	158
LOC_Os05g49230	PF01728.12	FtsJ	Family	178	1.30E-52	22	201	1	181
LOC_Os05g51180	PF04774.8	HABP4_PAI-RBP1	Family	95.6	1.70E-27	161	273	1	105
LOC_Os05g51180	PF09598.3	Stm1_N	Family	37	3.20E-09	5	72	1	65
LOC_Os06g01700	PF04889.5	Cwf_Cwc_15	Family	262.1	4.00E-78	1	230	1	239
LOC_Os06g02510	PF01294.11	Ribosomal_L13e	Family	310.5	2.00E-93	6	185	1	179
LOC_Os06g02510	PF01294.11	Ribosomal_L13e	Family	250.9	3.90E-75	6	137	1	132
LOC_Os06g02510	PF01294.11	Ribosomal_L13e	Family	150.2	3.20E-44	6	85	1	80
LOC_Os06g05660	PF00956.11	NAP	Family	277.3	6.30E-83	59	306	1	243
LOC_Os06g05660	PF00956.11	NAP	Family	277.8	4.40E-83	55	297	1	243
LOC_Os06g05660	PF00956.11	NAP	Family	277.8	4.40E-83	59	301	1	243
LOC_Os06g05660	PF00956.11	NAP	Family	277.9	4.10E-83	55	297	1	243
LOC_Os06g05660	PF00956.11	NAP	Family	278	3.80E-83	55	297	1	243
LOC_Os06g07580	PF04758.7	Ribosomal_S30	Family	105.6	6.00E-31	3	60	1	58
LOC_Os06g08770	PF06068.6	TIP49	Family	592.1	3.20E-178	18	420	1	396
LOC_Os06g09570	PF06858.7	NOG1	Family	93.2	5.10E-27	235	292	1	58
LOC_Os06g10430	PF06972.4	DUF1296	Family	109.7	3.90E-32	21	77	4	60
LOC_Os06g10710	PF04504.7	DUF573	Family	45.3	6.40E-12	96	183	6	98
LOC_Os06g30320	PF07540.4	NOC3p	Family	109.3	6.70E-32	198	290	2	95
LOC_Os06g30320	PF03914.10	CBF	Family	91.4	3.10E-26	561	714	2	165
LOC_Os06g36160	PF01282.12	Ribosomal_S24e	Family	125.7	3.60E-37	28	110	1	83
LOC_Os06g40560	PF00004.22	AAA	Family	146	5.30E-43	178	311	1	130
LOC_Os06g41384	PF00642.17	zf-CCCH	Family	21.7	9.00E-05	34	58	2	26
LOC_Os06g41384	PF00642.17	zf-CCCH	Family	37	1.50E-09	260	285	1	27
LOC_Os06g46890	PF00642.17	zf-CCCH	Family	22.6	4.70E-05	14	37	1	26
LOC_Os06g48230	PF03870.8	RNA_pol_Rpb8	Family	173.7	1.70E-51	52	190	1	138
LOC_Os06g48230	PF03870.8	RNA_pol_Rpb8	Family	125.2	1.50E-36	52	156	1	106
LOC_Os06g48350	PF01873.10	eIF-5_eIF-2B	Family	140.5	1.60E-41	4	129	5	123
LOC_Os06g48350	PF02020.11	W2	Family	73.3	8.60E-21	371	450	2	84
LOC_Os06g48355	PF01873.10	eIF-5_eIF-2B	Family	140.5	1.60E-41	4	129	5	123
LOC_Os06g48355	PF02020.11	W2	Family	73.3	8.60E-21	371	450	2	84
LOC_Os06g48750	PF00271.24	Helicase_C	Family	87.7	2.60E-25	301	375	4	78
LOC_Os06g48750	PF00271.24	Helicase_C	Family	88	2.10E-25	256	330	4	78
LOC_Os07g01920	PF06858.7	NOG1	Family	93.2	5.10E-27	235	292	1	58
LOC_Os07g08330	PF00573.15	Ribosomal_L4	Family	155.1	1.10E-45	28	267	3	191
LOC_Os07g10350	PF05843.7	Suf	Family	20.7	0.00021	636	739	74	153

Additional File 3 cont.: Pfam Domain Assignment for Proteins Identified with two or more peptides

LOC_Os07g10350	PF05843.7	Suf	Family	36.2	4.10E-09	783	879	41	138
LOC_Os07g12250	PF01246.13	Ribosomal_L24e	Family	121.6	6.90E-36	3	73	1	71
LOC_Os07g12320	PF09384.3	UTP15_C	Family	137.8	1.70E-40	381	525	1	146
LOC_Os07g12910	PF12165.1	DUF3594	Family	236	6.60E-71	12	139	1	130
LOC_Os07g12910	PF12165.1	DUF3594	Family	208.5	2.00E-62	12	123	1	114
LOC_Os07g12910	PF12165.1	DUF3594	Family	209	1.40E-62	12	120	1	111
LOC_Os07g19190	PF01246.13	Ribosomal_L24e	Family	106	5.00E-31	1	67	1	67
LOC_Os07g20580	PF00271.24	Helicase_C	Family	86	9.10E-25	362	437	3	78
LOC_Os07g33340	PF00271.24	Helicase_C	Family	68	3.80E-19	349	420	5	77
LOC_Os07g41260	PF01535.13	PPR	Family	19.4	0.00054	293	322	2	31
LOC_Os07g41260	PF01535.13	PPR	Family	15.8	0.0076	217	245	3	31
LOC_Os07g41260	PF01535.13	PPR	Family	9.7	0.69	400	429	2	31
LOC_Os07g41260	PF01535.13	PPR	Family	10.3	0.42	370	393	7	30
LOC_Os07g41740	PF12165.1	DUF3594	Family	270.4	1.50E-81	8	140	1	133
LOC_Os07g42170	PF00828.12	Ribosomal_L18e	Family	114.5	3.20E-33	20	144	1	128
LOC_Os07g42450	PF00318.13	Ribosomal_S2	Family	136.3	6.00E-40	22	189	1	210
LOC_Os07g42950	PF01092.12	Ribosomal_S6e	Family	187.1	6.60E-56	1	129	1	127
LOC_Os07g43980	PF00271.24	Helicase_C	Family	85.5	1.20E-24	340	415	2	77
LOC_Os07g44190	PF01509.11	TruB_N	Family	70.8	1.00E-19	124	240	1	149
LOC_Os07g44190	PF01472.13	PUA	Family	74	4.60E-21	311	383	1	73
LOC_Os07g46370	PF07304.4	SRA1	Family	29.2	5.10E-07	987	1122	5	138
LOC_Os07g46720	PF01798.11	Nop	Family	42.5	3.00E-11	266	308	1	43
LOC_Os07g47420	PF03657.6	UPF0113	Family	30.3	2.20E-07	50	183	40	162
LOC_Os07g49150	PF00004.22	AAA	Family	138.3	1.30E-40	230	363	1	130
LOC_Os08g03640	PF00428.12	Ribosomal_60s	Family	70.3	1.00E-19	234	318	1	88
LOC_Os08g03640	PF00466.13	Ribosomal_L10	Family	78.8	1.80E-22	7	108	2	98
LOC_Os08g04280	PF00022.12	Actin	Family	411.4	1.80E-123	4	409	1	347
LOC_Os08g04280	PF00022.12	Actin	Family	488.8	5.60E-147	4	442	1	380
LOC_Os08g05840	PF02919.8	Topoisom_I_N	Family	332.1	8.40E-100	372	584	1	215
LOC_Os08g06040	PF01199.11	Ribosomal_L34e	Family	126.3	3.20E-37	1	96	1	94
LOC_Os08g21840	PF00828.12	Ribosomal_L18e	Family	66.5	2.30E-18	99	224	3	128
LOC_Os08g31240	PF08644.4	SPT16	Family	173.5	1.90E-51	484	635	1	152
LOC_Os08g32090	PF00271.24	Helicase_C	Family	61.1	5.10E-17	312	387	3	78
LOC_Os08g33920	PF01199.11	Ribosomal_L34e	Family	126.3	3.20E-37	1	96	1	94
LOC_Os08g36450	PF04504.7	DUF573	Family	99.8	6.30E-29	188	287	2	98
LOC_Os08g39140	PF00183.11	HSP90	Family	882.5	6.40E-266	185	699	1	531
LOC_Os08g39140	PF00183.11	HSP90	Family	786.3	9.40E-237	185	628	1	454
LOC_Os08g39140	PF00183.11	HSP90	Family	883.1	4.10E-266	100	614	1	531
LOC_Os08g39140	PF00183.11	HSP90	Family	520.1	4.10E-156	185	482	1	308
LOC_Os08g41300	PF01655.11	Ribosomal_L32e	Family	163.2	1.30E-48	15	123	2	110
LOC_Os08g44480	PF03297.8	Ribosomal_S25	Family	157.7	6.10E-47	1	106	1	105
LOC_Os08g44480	PF03297.8	Ribosomal_S25	Family	103.9	3.10E-30	1	75	1	74
LOC_Os09g02810	PF03914.10	CBF	Family	185	5.30E-55	518	735	2	165
LOC_Os09g15770	PF02020.11	W2	Family	73.3	8.30E-21	371	450	2	84
LOC_Os09g15770	PF01873.10	eIF-5_eIF-2B	Family	140.1	2.20E-41	4	129	5	123

Additional File 3 cont.: Pfam Domain Assignment for Proteins Identified with two or more peptides

LOC_Os09g15775	PF01873.10	eIF-5_eIF-2B	Family	140.1	2.20E-41	4	129	5	123
LOC_Os09g15775	PF02020.11	W2	Family	73.3	8.30E-21	371	450	2	84
LOC_Os09g24820	PF04770.5	ZF-HD_dimer	Family	102.6	6.70E-30	39	94	4	57
LOC_Os09g27850	PF04504.7	DUF573	Family	104.2	2.80E-30	191	288	2	98
LOC_Os09g30412	PF00183.11	HSP90	Family	883.8	2.70E-266	185	699	1	531
LOC_Os09g30418	PF00183.11	HSP90	Family	880.2	3.30E-265	185	698	1	530
LOC_Os09g30418	PF10714.2	LEA_6	Family	114.1	1.40E-33	730	830	1	79
LOC_Os09g32500	PF01655.11	Ribosomal_L32e	Family	162.8	1.70E-48	15	123	2	110
LOC_Os09g32520	PF01655.11	Ribosomal_L32e	Family	161	6.40E-48	15	123	2	110
LOC_Os09g32532	PF01655.11	Ribosomal_L32e	Family	162.5	2.20E-48	15	123	2	110
LOC_Os09g36160	PF05142.5	DUF702	Family	228.2	3.80E-68	92	242	3	153
LOC_Os09g37860	PF01189.10	Nol1_Nop2_Fmu	Family	294	8.40E-88	258	544	2	283
LOC_Os09g39540	PF03297.8	Ribosomal_S25	Family	155.5	2.80E-46	1	106	1	105
LOC_Os09g39540	PF03297.8	Ribosomal_S25	Family	129.4	3.70E-38	11	91	25	105
LOC_Os10g27190	PF00833.11	Ribosomal_S17e	Family	197.8	3.00E-59	1	121	1	121
LOC_Os10g30580	PF00004.22	AAA	Family	158.7	6.40E-47	520	653	1	130
LOC_Os10g30580	PF00004.22	AAA	Family	158.4	7.80E-47	247	376	1	129
LOC_Os10g30580	PF00004.22	AAA	Family	30.4	2.80E-07	1	40	91	130
LOC_Os10g35280	PF03715.6	Noc2	Family	292.6	2.00E-87	273	590	2	298
LOC_Os10g41470	PF00467.22	KOW	Family	24.6	9.70E-06	7	38	1	30
LOC_Os10g41470	PF01777.11	Ribosomal_L27e	Family	106.3	4.90E-31	52	136	1	85
LOC_Os11g01420	PF00466.13	Ribosomal_L10	Family	69.5	1.40E-19	22	122	4	99
LOC_Os11g04070	PF00428.12	Ribosomal_60s	Family	71.9	3.40E-20	234	319	1	88
LOC_Os11g04070	PF00466.13	Ribosomal_L10	Family	78.8	1.80E-22	7	108	2	98
LOC_Os11g05562	PF03297.8	Ribosomal_S25	Family	155.7	2.50E-46	1	106	1	105
LOC_Os11g06750	PF00297.15	Ribosomal_L3	Family	405.8	5.90E-122	50	343	1	263
LOC_Os11g11390	PF00252.11	Ribosomal_L16	Family	115.3	1.30E-33	5	166	1	132
LOC_Os11g36390	PF00004.22	AAA	Family	49.8	3.00E-13	462	574	1	109
LOC_Os11g36390	PF00533.19	BRCT	Family	56.3	2.00E-15	259	334	3	78
LOC_Os11g37080	PF04410.7	Gar1	Family	134.5	1.60E-39	32	175	3	143
LOC_Os11g38900	PF00856.21	SET	Family	89.8	1.40E-25	652	781	1	158
LOC_Os11g38959	PF01479.18	S4	Family	52.6	1.70E-14	109	153	1	45
LOC_Os11g38959	PF00163.12	Ribosomal_S4	Family	90.7	4.60E-26	7	107	1	93
LOC_Os11g40090	PF06870.5	RNA_pol_I_A49	Family	149	1.10E-43	131	482	8	384
LOC_Os11g40090	PF06870.5	RNA_pol_I_A49	Family	154.4	2.40E-45	131	493	8	384
LOC_Os12g01430	PF00466.13	Ribosomal_L10	Family	69	2.00E-19	22	122	4	99
LOC_Os12g01430	PF00466.13	Ribosomal_L10	Family	41.7	6.40E-11	1	74	31	99
LOC_Os12g03880	PF00466.13	Ribosomal_L10	Family	78.8	1.80E-22	7	108	2	98
LOC_Os12g03880	PF00428.12	Ribosomal_60s	Family	68.3	4.50E-19	234	319	1	88
LOC_Os12g06910	PF03813.7	Nrap	Family	447.2	7.20E-134	472	1027	418	968
LOC_Os12g06910	PF03813.7	Nrap	Family	77.5	3.50E-22	319	471	231	381
LOC_Os12g06910	PF03813.7	Nrap	Family	102	1.40E-29	148	290	24	173
LOC_Os12g06910	PF03813.7	Nrap	Family	77.9	2.70E-22	319	471	231	381
LOC_Os12g06910	PF03813.7	Nrap	Family	102.3	1.10E-29	148	290	24	173
LOC_Os12g06910	PF03813.7	Nrap	Family	396.7	1.30E-118	469	871	415	824

Additional File 3 cont.: Pfam Domain Assignment for Proteins Identified with two or more peptides

LOC_Os12g07010	PF00297.15	Ribosomal_L3	Family	405.3	8.00E-122	50	343	1	263
LOC_Os12g14070	PF00012.13	HSP70	Family	875.6	8.90E-264	63	657	1	601
LOC_Os12g21798	PF01015.11	Ribosomal_S3Ae	Family	275.5	1.30E-82	12	222	2	195
LOC_Os12g38180	PF00012.13	HSP70	Family	175.9	6.50E-52	50	209	213	378
LOC_Os12g41715	PF00271.24	Helicase_C	Family	87.2	3.60E-25	365	436	7	78
LOC_Os12g44390	PF02463.12	SMC_N	Family	97.4	5.20E-28	16	145	2	135
LOC_Os05g41172	PF05033.9	Pre-SET	Motif	72.5	2.10E-20	395	494	2	103
LOC_Os11g38900	PF05033.9	Pre-SET	Motif	71.8	3.50E-20	533	633	1	103
LOC_Os01g08770	PF00400.25	WD40	Repeat	33.7	1.70E-08	272	309	2	39
LOC_Os01g13730	PF00400.25	WD40	Repeat	23.6	2.50E-05	62	95	7	39
LOC_Os01g13730	PF00400.25	WD40	Repeat	33.5	2.00E-08	101	137	3	39
LOC_Os01g13730	PF00400.25	WD40	Repeat	17.5	0.0021	194	232	2	39
LOC_Os01g13730	PF00400.25	WD40	Repeat	31.3	9.30E-08	281	316	3	38
LOC_Os01g13730	PF00400.25	WD40	Repeat	22.2	7.10E-05	335	360	14	39
LOC_Os01g14950	PF00514.16	Arm	Repeat	36.3	2.50E-09	274	312	2	40
LOC_Os01g14950	PF00514.16	Arm	Repeat	42.1	3.60E-11	105	145	2	41
LOC_Os01g14950	PF00514.16	Arm	Repeat	27.2	1.70E-06	402	440	2	40
LOC_Os01g14950	PF00514.16	Arm	Repeat	48.2	4.20E-13	147	186	1	40
LOC_Os01g14950	PF00514.16	Arm	Repeat	30.4	1.70E-07	189	230	1	41
LOC_Os01g14950	PF00514.16	Arm	Repeat	33.9	1.40E-08	243	271	13	41
LOC_Os01g14950	PF00514.16	Arm	Repeat	40.1	1.60E-10	315	356	1	41
LOC_Os01g14950	PF00514.16	Arm	Repeat	44.5	6.20E-12	358	397	1	40
LOC_Os01g21940	PF00400.25	WD40	Repeat	26.4	3.40E-06	222	246	15	39
LOC_Os01g21940	PF00400.25	WD40	Repeat	32.9	2.90E-08	266	295	9	38
LOC_Os01g21940	PF00400.25	WD40	Repeat	12.2	0.1	482	516	5	39
LOC_Os01g21940	PF00400.25	WD40	Repeat	41.8	4.60E-11	344	381	2	39
LOC_Os01g21940	PF00400.25	WD40	Repeat	24.3	1.50E-05	300	337	1	37
LOC_Os01g46060	PF00400.25	WD40	Repeat	11.3	0.2	101	127	9	35
LOC_Os01g49290	PF00400.25	WD40	Repeat	14.8	0.016	242	277	2	39
LOC_Os01g49290	PF00400.25	WD40	Repeat	45	4.60E-12	162	195	8	39
LOC_Os01g49290	PF00400.25	WD40	Repeat	23.1	3.80E-05	13	47	6	39
LOC_Os01g49290	PF00400.25	WD40	Repeat	17.9	0.0016	301	326	14	39
LOC_Os01g49290	PF00400.25	WD40	Repeat	32.6	3.60E-08	67	103	3	39
LOC_Os01g49290	PF00400.25	WD40	Repeat	39	3.60E-10	110	145	4	39
LOC_Os01g49290	PF00400.25	WD40	Repeat	33.3	2.20E-08	203	237	5	39
LOC_Os01g51300	PF00400.25	WD40	Repeat	30.9	1.30E-07	287	323	4	39
LOC_Os01g51300	PF00400.25	WD40	Repeat	21.5	0.00012	243	278	5	39
LOC_Os01g51300	PF00400.25	WD40	Repeat	11.3	0.19	181	203	17	39
LOC_Os01g51300	PF00400.25	WD40	Repeat	30.4	1.80E-07	335	372	3	39
LOC_Os01g51300	PF00400.25	WD40	Repeat	30.5	1.70E-07	326	363	3	39
LOC_Os01g51300	PF00400.25	WD40	Repeat	30.9	1.20E-07	278	314	4	39
LOC_Os01g51300	PF00400.25	WD40	Repeat	21.5	0.00011	234	269	5	39
LOC_Os01g51300	PF00400.25	WD40	Repeat	11.2	0.21	172	194	17	39
LOC_Os01g69970	PF00400.25	WD40	Repeat	33.3	2.20E-08	258	293	5	39
LOC_Os01g69970	PF00400.25	WD40	Repeat	15.8	0.0073	437	474	3	39

Additional File 3 cont.: Pfam Domain Assignment for Proteins Identified with two or more peptides

LOC_Os01g69970	PF00400.25	WD40	Repeat	43.6	1.30E-11	391	429	2	39
LOC_Os01g69970	PF00400.25	WD40	Repeat	23.5	2.80E-05	297	332	1	35
LOC_Os03g05720	PF00400.25	WD40	Repeat	16.1	0.0061	65	96	13	39
LOC_Os03g05720	PF00400.25	WD40	Repeat	30.3	2.00E-07	100	138	1	39
LOC_Os03g05720	PF00400.25	WD40	Repeat	12.1	0.11	534	561	10	38
LOC_Os03g05720	PF00400.25	WD40	Repeat	26.5	3.00E-06	608	646	1	39
LOC_Os03g05720	PF00400.25	WD40	Repeat	35.2	5.40E-09	146	180	5	39
LOC_Os03g05720	PF00400.25	WD40	Repeat	28.4	7.90E-07	186	221	3	38
LOC_Os03g05720	PF00400.25	WD40	Repeat	15.7	0.0082	390	421	7	39
LOC_Os03g05720	PF00400.25	WD40	Repeat	33.1	2.70E-08	651	688	2	39
LOC_Os03g05720	PF00400.25	WD40	Repeat	17.3	0.0025	468	509	4	39
LOC_Os03g05720	PF00400.25	WD40	Repeat	31.9	6.30E-08	571	603	6	38
LOC_Os03g18840	PF00400.25	WD40	Repeat	17.1	0.0029	193	230	2	38
LOC_Os03g18840	PF00400.25	WD40	Repeat	13.2	0.05	14	35	18	39
LOC_Os03g18840	PF00400.25	WD40	Repeat	14.9	0.014	115	144	6	35
LOC_Os03g18840	PF00400.25	WD40	Repeat	10.1	0.45	154	181	3	30
LOC_Os03g42770	PF00400.25	WD40	Repeat	12.2	0.1	158	188	9	39
LOC_Os03g42770	PF00400.25	WD40	Repeat	32.1	5.40E-08	252	289	2	39
LOC_Os03g42770	PF00400.25	WD40	Repeat	24.2	1.60E-05	220	247	12	39
LOC_Os03g42770	PF00400.25	WD40	Repeat	9.8	0.57	415	441	12	38
LOC_Os03g42770	PF00400.25	WD40	Repeat	17.7	0.0019	345	371	12	39
LOC_Os03g42770	PF00400.25	WD40	Repeat	8.7	1.2	300	330	8	39
LOC_Os03g52470	PF00400.25	WD40	Repeat	23.2	3.40E-05	211	235	15	39
LOC_Os03g52470	PF00400.25	WD40	Repeat	16.4	0.0049	289	325	2	38
LOC_Os03g52470	PF00400.25	WD40	Repeat	11.1	0.22	65	95	11	39
LOC_Os04g50660	PF00400.25	WD40	Repeat	17.5	0.0021	624	660	2	38
LOC_Os04g50660	PF00400.25	WD40	Repeat	21.3	0.00013	75	105	9	39
LOC_Os04g50660	PF00400.25	WD40	Repeat	37.3	1.20E-09	110	147	2	39
LOC_Os04g50660	PF00400.25	WD40	Repeat	31.3	9.60E-08	152	191	2	39
LOC_Os04g50660	PF00400.25	WD40	Repeat	36.3	2.60E-09	196	233	2	39
LOC_Os04g50660	PF00400.25	WD40	Repeat	25.8	5.10E-06	400	440	2	39
LOC_Os04g50660	PF00400.25	WD40	Repeat	33.7	1.70E-08	445	483	2	39
LOC_Os04g50660	PF00400.25	WD40	Repeat	30.1	2.30E-07	498	535	2	39
LOC_Os04g50660	PF00400.25	WD40	Repeat	33.6	1.80E-08	543	576	5	38
LOC_Os04g50660	PF00400.25	WD40	Repeat	33.4	2.10E-08	581	618	1	38
LOC_Os04g56720	PF00415.11	RCC1	Repeat	25	1.30E-05	198	256	6	51
LOC_Os04g56720	PF00415.11	RCC1	Repeat	24.6	1.70E-05	364	410	5	50
LOC_Os04g56720	PF00415.11	RCC1	Repeat	35.7	5.70E-09	259	306	1	45
LOC_Os04g56720	PF00415.11	RCC1	Repeat	22.3	8.60E-05	140	186	1	49
LOC_Os04g56720	PF00415.11	RCC1	Repeat	59.1	2.90E-16	88	137	1	51
LOC_Os05g06350	PF00514.16	Arm	Repeat	26.8	2.40E-06	408	446	2	40
LOC_Os05g06350	PF00514.16	Arm	Repeat	45.1	4.20E-12	364	403	1	40
LOC_Os05g06350	PF00514.16	Arm	Repeat	40.5	1.10E-10	322	362	2	41
LOC_Os05g06350	PF00514.16	Arm	Repeat	34.1	1.20E-08	280	318	2	40
LOC_Os05g06350	PF00514.16	Arm	Repeat	25.9	4.50E-06	249	277	13	41

Additional File 3 cont.: Pfam Domain Assignment for Proteins Identified with two or more peptides

LOC_Os05g06350	PF00514.16	Arm	Repeat	30.5	1.60E-07	195	236	1	41
LOC_Os05g06350	PF00514.16	Arm	Repeat	51	5.60E-14	154	192	2	40
LOC_Os05g06350	PF00514.16	Arm	Repeat	35.2	5.40E-09	111	151	2	41
LOC_Os05g16660	PF00400.25	WD40	Repeat	12.3	0.091	285	312	8	32
LOC_Os05g16660	PF00400.25	WD40	Repeat	14.2	0.024	7	37	8	38
LOC_Os05g16660	PF00400.25	WD40	Repeat	14.3	0.023	7	37	8	38
LOC_Os05g16660	PF00400.25	WD40	Repeat	19.3	0.00056	140	174	5	39
LOC_Os05g16660	PF00400.25	WD40	Repeat	19.4	0.00054	140	174	5	39
LOC_Os05g16660	PF00400.25	WD40	Repeat	24.3	1.50E-05	188	219	8	39
LOC_Os05g16660	PF00400.25	WD40	Repeat	24.4	1.40E-05	188	219	8	39
LOC_Os05g16660	PF00400.25	WD40	Repeat	25.7	5.70E-06	285	314	8	37
LOC_Os05g16660	PF00400.25	WD40	Repeat	36.8	1.70E-09	44	80	3	39
LOC_Os05g16660	PF00400.25	WD40	Repeat	36.9	1.60E-09	44	80	3	39
LOC_Os05g44320	PF00400.25	WD40	Repeat	14.3	0.022	135	168	4	37
LOC_Os05g44320	PF00400.25	WD40	Repeat	14.5	0.019	135	168	4	37
LOC_Os05g44320	PF00400.25	WD40	Repeat	18	0.0015	546	575	10	39
LOC_Os05g44320	PF00400.25	WD40	Repeat	18.2	0.0013	546	575	10	39
LOC_Os05g44320	PF00400.25	WD40	Repeat	30.9	1.30E-07	411	447	3	39
LOC_Os05g44320	PF00400.25	WD40	Repeat	31.1	1.10E-07	411	447	3	39
LOC_Os05g44320	PF00400.25	WD40	Repeat	34	1.40E-08	497	533	3	39
LOC_Os05g44320	PF00400.25	WD40	Repeat	34.2	1.20E-08	497	533	3	39
LOC_Os05g44320	PF00400.25	WD40	Repeat	36.8	1.80E-09	373	404	7	38
LOC_Os05g44320	PF00400.25	WD40	Repeat	37	1.50E-09	373	404	7	38
LOC_Os06g03780	PF00400.25	WD40	Repeat	11.2	0.2	277	307	12	39
LOC_Os06g43690	PF00400.25	WD40	Repeat	16.6	0.0041	310	342	6	38
LOC_Os06g43690	PF00400.25	WD40	Repeat	13.1	0.051	226	254	12	39
LOC_Os06g43690	PF00400.25	WD40	Repeat	15	0.013	266	296	8	38
LOC_Os06g43690	PF00400.25	WD40	Repeat	12.9	0.061	468	503	4	39
LOC_Os06g43690	PF00400.25	WD40	Repeat	44.1	8.90E-12	550	586	3	39
LOC_Os06g43690	PF00400.25	WD40	Repeat	19.3	0.00058	173	208	2	37
LOC_Os07g12320	PF00400.25	WD40	Repeat	19.5	0.0005	223	251	10	39
LOC_Os07g12320	PF00400.25	WD40	Repeat	8.9	1.1	258	298	3	39
LOC_Os07g12320	PF00400.25	WD40	Repeat	18.7	0.00091	179	210	9	39
LOC_Os07g25440	PF00400.25	WD40	Repeat	22.9	4.20E-05	587	621	4	38
LOC_Os07g25440	PF00400.25	WD40	Repeat	39.4	2.70E-10	336	373	2	39
LOC_Os07g25440	PF00400.25	WD40	Repeat	13.8	0.031	544	579	4	39
LOC_Os07g25440	PF00400.25	WD40	Repeat	17.6	0.0019	650	676	12	38
LOC_Os07g25440	PF00400.25	WD40	Repeat	13.9	0.029	519	554	4	39
LOC_Os07g25440	PF00400.25	WD40	Repeat	17.7	0.0019	625	651	12	38
LOC_Os07g25440	PF00400.25	WD40	Repeat	23	4.00E-05	562	596	4	38
LOC_Os07g25440	PF00400.25	WD40	Repeat	39.4	2.60E-10	311	348	2	39
LOC_Os07g32350	PF00400.25	WD40	Repeat	20.7	0.00021	304	332	11	39
LOC_Os07g40930	PF00400.25	WD40	Repeat	28.8	6.10E-07	355	390	5	39
LOC_Os07g40930	PF00400.25	WD40	Repeat	40.9	9.00E-11	199	233	5	39
LOC_Os07g40930	PF00400.25	WD40	Repeat	25.9	4.70E-06	267	302	3	39

Additional File 3 cont.: Pfam Domain Assignment for Proteins Identified with two or more peptides

LOC_Os07g40930	PF00400.25	WD40	Repeat	17.2	0.0027	110	137	10	38
LOC_Os07g40930	PF00400.25	WD40	Repeat	9.2	0.9	319	344	15	39
LOC_Os07g40930	PF00400.25	WD40	Repeat	27.7	1.30E-06	142	184	2	39
LOC_Os07g40930	PF00400.25	WD40	Repeat	10.4	0.37	397	429	4	37
LOC_Os07g41190	PF00400.25	WD40	Repeat	13.4	0.042	255	282	11	38
LOC_Os07g41190	PF00400.25	WD40	Repeat	9.7	0.61	433	455	16	38
LOC_Os07g41190	PF00400.25	WD40	Repeat	21.7	9.90E-05	381	413	6	39
LOC_Os07g46370	PF00400.25	WD40	Repeat	24.3	1.50E-05	257	294	3	39
LOC_Os07g46370	PF00400.25	WD40	Repeat	11.2	0.21	175	203	12	39
LOC_Os07g46370	PF00400.25	WD40	Repeat	14.8	0.015	117	153	4	39
LOC_Os08g21660	PF00400.25	WD40	Repeat	11	0.24	285	301	8	24
LOC_Os08g21660	PF00400.25	WD40	Repeat	14.2	0.024	7	37	8	38
LOC_Os08g21660	PF00400.25	WD40	Repeat	14.3	0.022	7	37	8	38
LOC_Os08g21660	PF00400.25	WD40	Repeat	19.8	0.00042	139	174	4	39
LOC_Os08g21660	PF00400.25	WD40	Repeat	24.3	1.50E-05	188	219	8	39
LOC_Os08g21660	PF00400.25	WD40	Repeat	24.4	1.50E-05	188	219	8	39
LOC_Os08g21660	PF00400.25	WD40	Repeat	25.7	5.70E-06	285	314	8	37
LOC_Os08g21660	PF00400.25	WD40	Repeat	37.7	9.10E-10	44	80	3	39
LOC_Os08g21660	PF00400.25	WD40	Repeat	37.8	8.70E-10	44	80	3	39
LOC_Os09g24260	PF00400.25	WD40	Repeat	18.8	0.00081	291	324	6	39
LOC_Os10g32880	PF00400.25	WD40	Repeat	41.8	4.60E-11	224	260	2	38
LOC_Os10g32880	PF00400.25	WD40	Repeat	15.1	0.012	452	480	10	38
LOC_Os10g32880	PF00400.25	WD40	Repeat	10.6	0.32	326	350	15	39
LOC_Os10g32880	PF00400.25	WD40	Repeat	28.4	7.70E-07	269	306	2	39
LOC_Os10g32880	PF00400.25	WD40	Repeat	27.2	1.80E-06	357	391	4	39
LOC_Os10g32880	PF00400.25	WD40	Repeat	9.4	0.77	193	219	13	39
LOC_Os11g43890	PF00400.25	WD40	Repeat	39.8	2.00E-10	239	273	5	39
LOC_Os11g43890	PF00400.25	WD40	Repeat	30.3	2.00E-07	193	231	2	39
LOC_Os11g43890	PF00400.25	WD40	Repeat	32.4	4.30E-08	160	188	11	39
LOC_Os11g43890	PF00400.25	WD40	Repeat	19.4	0.00055	96	134	2	39
LOC_Os11g43890	PF00400.25	WD40	Repeat	11	0.24	60	91	8	39
LOC_Os11g43890	PF00400.25	WD40	Repeat	20.8	0.0002	7	46	4	38
LOC_Os11g43890	PF00400.25	WD40	Repeat	22.4	6.00E-05	280	321	4	39
LOC_Os12g41620	PF00400.25	WD40	Repeat	8.1	2	476	502	12	38
LOC_Os12g41620	PF00400.25	WD40	Repeat	18.2	0.0013	40	84	3	39
LOC_Os12g41620	PF00400.25	WD40	Repeat	15.5	0.0091	203	232	9	38
LOC_Os12g41620	PF00400.25	WD40	Repeat	17.7	0.0018	267	300	5	38
LOC_Os12g42150	PF00400.25	WD40	Repeat	18.5	0.0011	38	73	4	39
LOC_Os12g42150	PF00400.25	WD40	Repeat	23.9	2.00E-05	259	297	8	39
