# Supplementary material for: Nuclear proteome response to cell wall removal in rice (Oryza sativa)
Source: Proteome Sci. 2013 Jun 19;11:26. doi: 10.1186/1477-5956-11-26 (PMC3695858; doi:10.1186/1477-5956-11-26)
Supplement: Additional file 4 — As List of Primers Used in the Study. [file 1477-5956-11-26-S4.pdf]

Additional File 4. List of Primers Used in Study

Primer Name	Sequence
LOC_Os01g08970f	AGAAGTTGCCAAGATGGGTAT
LOC_Os01g08970r	TGTAGAATGAGGGTGGGAGG
LOC_Os01g13730f	GGTTTGGCGACATCTACTGA
LOC_Os01g13730r	GTAGACCGCTGATGGCTGA
LOC_Os01g16870f	GCTGCGGTCGTTAGTTCTC
LOC_Os01g16870r	GCTCCTTGTGGCTTAAATAGTC
LOC_Os01g27730f	AAGCAATATCCCACAAAGCA
LOC_Os01g27730r	AGGAAGACCAACAATACCCAC
LOC_Os01g33030f	AGCAATCTCCCCTACTACTGTCT
LOC_Os01g33030r	TGTCGCCTAACTACTAGATCCATA
LOC_Os02g04040f	CAGCCCCATTCTATCTGTTTG
LOC_Os02g04040r	TCTGGTCGGAATGTCGTTG
LOC_Os02g49270f	AACAGCCCATTCAGTCAAAA
LOC_Os02g49270r	TGTCCTTAGTCACCCCATCA
LOC_Os03g01970f	AACGACCTAAGAGGGAAGACC
LOC_Os03g01970r	GAATCCATTGGCACAGCAC
LOC_Os03g36930f	GATGAGATGTTGGGCAGAGG
LOC_Os03g36930r	TGGCAGAGTTGCGGAGAT
Ubiquitin-F	AACCAGCTGAGGCCCAAGA
Ubiquitin-R	ACGATTGATTTAACCAGTCCATGA
